# Integrated Analysis of Environment, Cattle and Human Serological Data: Risks and Mechanisms of Transmission of Rift Valley Fever in Madagascar

**DOI:** 10.1371/journal.pntd.0004827

**Published:** 2016-07-14

**Authors:** Marie-Marie Olive, Véronique Chevalier, Vladimir Grosbois, Annelise Tran, Soa-Fy Andriamandimby, Benoit Durand, Jean-Pierre Ravalohery, Seta Andriamamonjy, Fanjasoa Rakotomanana, Christophe Rogier, Jean-Michel Heraud

**Affiliations:** 1 CIRAD, Animal and Integrated Risk Management (AGIRs) Unit, Montpellier, France; 2 Institut Pasteur de Madagascar, Virology Unit, Antananarivo, Madagascar; 3 Paris-Est University, ANSES, Laboratory for Animal Health, Epidemiology Unit, Maisons-Alfort, France; 4 Institut Pasteur de Madagascar, Epidemiology Unit, Antananarivo, Madagascar; 5 Institut Pasteur de Madagascar, Direction, Antananarivo, Madagascar; University of California, Davis, UNITED STATES

## Abstract

**Background:**

Rift Valley fever (RVF) is a vector-borne disease affecting ruminants and humans. Madagascar was heavily affected by RVF in 2008–2009, with evidence of a large and heterogeneous spread of the disease. The identification of at-risk environments is essential to optimize the available resources by targeting RVF surveillance in Madagascar. Herein, the objectives of our study were: (i) to identify the environmental factors and areas favorable to RVF transmission to both cattle and human and (ii) to identify human behaviors favoring human infections in Malagasy contexts.

**Methodology/Principal Findings:**

First, we characterized the environments of Malagasy communes using a Multiple Factor Analysis (MFA). Then, we analyzed cattle and human serological data collected at national level using Generalized Linear Mixed Models, with the individual serological status (cattle or human) as the response, and MFA factors, as well as other potential risk factors (cattle density, human behavior) as explanatory variables. Cattle and human seroprevalence rates were positively associated to humid environments (p<0.001). Areas with high cattle density were at risk (p<0.01; OR = 2.6). Furthermore, our analysis showed that frequent contact with raw milk contributed to explain human infection (OR = 1.6). Finally, our study highlighted the eastern-coast, western and north-western parts as high-risk areas for RVF transmission in cattle.

**Conclusions/Significance:**

Our integrated approach analyzing environmental, cattle and human datasets allow us to bring new insight on RVF transmission patterns in Madagascar. The association between cattle seroprevalence, humid environments and high cattle density suggests that concomitant vectorial and direct transmissions are critical to maintain RVF enzootic transmission. Additionally, in the at-risk humid environment of the western, north-western and the eastern-coast areas, suitable to Culex and Anopheles mosquitoes, vectorial transmission probably occurs in both cattle and human. The relative contribution of vectorial or direct transmissions could be further assessed by mathematic modelling.

## Author Summary

Rift Valley fever virus (RVFV) is a pathogen that causes a vector-borne tropical disease. The disease affects ruminants and humans and severely impacts the health and economy of affected countries. Madagascar was heavily affected by Rift Valley fever (RVF) in 2008–2009, with evidence of a large and heterogeneous spread of the disease. Our study aims at identifying environmental and human-related risk factors for RVFV transmission. First, we characterized Malagasy environments according to their putative influence on RVFV mosquito density and population dynamics. Then we statistically analyzed cattle and human serological data collected at a national level with the individual serological status as response, and Malagasy environments previously characterized by climatic and landscape variables as well as other potential risk factors as explanatory variables. Our results identified humid environments of the western, north-western and eastern parts of the island as risky areas. The identification of at-risk environments is essential to focus veterinary surveillance and control of RVFV.

## Introduction

Rift Valley fever virus (RVFV) is an arthropod-borne zoonotic virus belonging to the *Bunyaviridae* family and affecting ruminants and humans. Infection causes abortion in pregnant ruminants and acute deaths in newborns [1,2]. In the majority of human cases, infection is asymptomatic or causes mild symptoms such as fever, headaches and muscle pains [2]. However severe cases occur, characterized by retinitis, encephalitis, or hemorrhagic forms that may lead to death [2]. Ruminants are infected through vector bites and probably also by direct contact with infected tissues or fluids, such as blood or abortion products [2,3]. Humans are mainly infected through direct contact with infectious tissues or fluids of ruminants but vectorial transmission has been suspected in Central African Republic (RCA) and Gabon [4,5]. Virus circulation has been reported in several eco-climatic areas: arid in Western Africa and Arabic Peninsula [1,6], sub-humid in Eastern Africa [7,8], wet forests in central Africa [5], dam and irrigated agricultural land under hot climatic conditions in Egypt, Mauritania and Sudan [9–11] and recently humid highlands in Madagascar [3,12]. The respective roles of direct and vectorial transmissions remain unevaluated in both human and cattle and probably vary among these eco-climatic areas.

Madagascar experienced two major Rift Valley fever (RVF) outbreaks: 1990–91 in the eastern-coast and central highlands and 2008–09 in the south, the north and the highlands [13–15]. The last outbreaks occurred in two epidemic waves during the two successive rainy seasons of 2007–08 and 2008–09. Following the first wave, passive surveillance and emergency response were developed. Sentinel surveillance in herds were set up with field veterinarians [16]. This sentinel surveillance allowed the early detection of the second wave of outbreak in cattle at the end of 2008 and thus the implementation of local control measures to prevent the spreading of RVF outside the region [15,16]. At the end of the epidemic, about 700 suspected human cases were recorded from which 26 were fatal. About 400 human and cattle samples were received for laboratory analyses and RVF infection was confirmed or considered as probable in 86 human and 46 ruminant samples [15]. Following the 2008–09 epidemics, studies showed a wide and heterogeneous spread of RVFV infection both in human and cattle [15,17] suggesting that some areas were more favorable than others to transmission [17]. Madagascar has a large variety of eco-climatic patterns, including semi-arid in the south, tropical in the west and on the eastern-coast, and temperate in the central highlands [18]. Apart from the highlands [3,12,19], RVF epidemiology is poorly understood in this country [15,17]. Since 2007, a human syndromic-based surveillance system has been developed which has allowed the detection of the first case of RVF in humans in 2008 [15]. Besides, retrospective investigations suggested that RVFV circulated among livestock since December 2007 [15], revealing a dearth in veterinary surveillance. The main difficulty to implement veterinary surveillance in Madagascar is the lack of basic means to collect and communicate veterinary information [20]. Thus, the identification of at-risk environments is essential to optimize the available resources by targeting RVF surveillance. In addition, there is a need to provide insight into the role of the two transmission routes and better adapt available control measures.

Herein, the objectives of our study were: (i) to identify the environmental factors and areas favorable to RVFV transmission to both cattle and human and (ii) to identify human behaviors favoring human infections in Malagasy contexts.

## Materials and Methods

To achieve these goals, we characterized the environments of Malagasy communes using a Multiple Factor Analysis (MFA). Then we analyzed cattle and human serological data using a Generalized Linear Mixed Models (GLMMs), with the individual serological status (cattle or human) as the response, and MFA factors, as well as potential other risk factors (covariates), as explanatory variables.

### Cattle and human datasets

**Fig 1:**
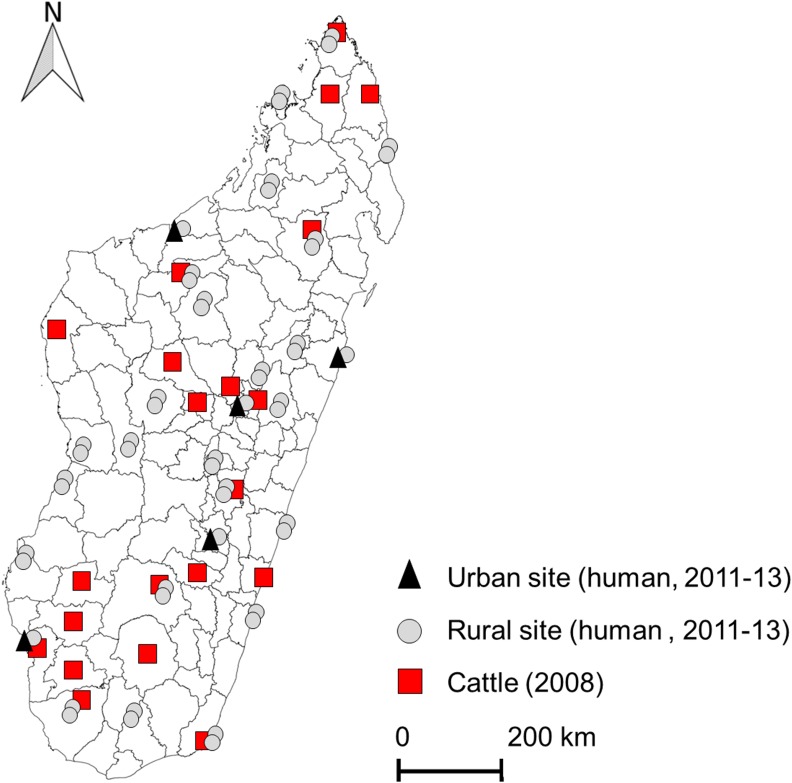
Cattle and human sampling sites [17]. Animal and human sera were analyzed using commercial ELISA kits (Biological Diagnostic Supplies Ltd., BDSL) to detect anti-RVFV immunoglobulin (Ig) G [17,22,23]. Cattle and human data were aggregated at the commune level (n = 1,578).

The cattle dataset contained results of a national cross-sectional serological survey performed in August 2008 on 3,450 ruminants [17]. Only cattle with known breeding location were included in the study (n = 1,432; Fig 1; [17]). The human dataset contained data from a national cross-sectional serological survey conducted from November 2011 to April 2012 and from October 2012 to May 2013 in 56 sites (cities or villages). Six percent of these sera were used in a previous study [21]. In each of these sites, 30 adults were randomly chosen and sampled on a voluntary basis (Fig 1). Potential contacts with ruminants or fresh ruminant fluids (secretion, blood, milk) and socio-professional categories—butcher, farmer, health worker, worker in contact with environment (water, forest) and others (teacher, student, administrative worker, retired)—were documented through a dedicated questionnaire.

### Ethics statement

The cattle study was performed in collaboration with the Malagasy Veterinary Services and animals were sampled by qualified veterinarians [17]. The human study protocol was approved by the Malagasy competent authorities, the Malagasy Ethic National Committee (authorization N°066/MSAMP/CE, 26th July 2011). After reading of the informed consent letter, written and oral consent was obtained from volunteering individuals. Participants were sampled by qualified investigators and the data were analyzed anonymously.

### Covariates

The following covariates were selected according to their putative influence on mosquito density and population dynamics or on the risk of contact with ruminants:

- Cattle density. This variable has previously been identified as a risk factor for RVF transmission [7,24].
- Surface covered by water bodies and landscape classes (such as forest, shrub, and agricultural areas). Density and population dynamics of vectors are influenced by environmental factors such as climate, the presence of water bodies and other landscape features [1,25]. The presence of temporary water bodies and floodplains are known as risk factors for RVF in semi-arid areas in eastern Africa, the Arabian Peninsula and Western Africa [1]. Artificial water bodies such as dam and irrigated rice fields are also known to be associated with high abundance of RVFV vectors in western Africa [1]. Furthermore, RVFV transmission occurred in forested or shrubby areas [5,8,26,27]. A recent study details the mosquito species and their habitat in Madagascar [28]. Briefly, RVF potential mosquito vectors belong to the genera *Aedes*, *Anopheles*, *Culex*, *Eretmapodites* and *Mansonia* [28]. The breeding areas of the *Aedes* genus are mostly associated with temporary water bodies such as flooded area, temporary pond, puddles, rice field [28]. *Culex* and *Anopheles* mosquitoes breeding areas are diversified and could be temporary (rice field, swamps) or permanent (lakes, pond). Stagnant and permanent water bodies are the habitat of *Eretmapodites* and *Mansonia* respectively [28].
- Rainfall, Normalized Difference Vegetation Index (NDVI) and temperatures. The risk of RVFV infection in Eastern and Southern Africa has been shown to vary as a function of rainfall, NDVI and temperatures [29,30].
- Human related factors: habitat, gender, profession, contact with ruminant and ruminant products [31].

Values of covariates were computed at the commune level (except for human behaviors which were at the individual level).

#### Cattle density

For each of the 1,578 communes considered, cattle density was estimated using the new global distribution maps for cattle produced by the Food and Agriculture Organization of the United Nations (FAO; http://www.fao.org/Ag/againfo/resources/en/glw/GLW_dens.html; [32]).

#### Water bodies and landscape classes

A landscape map of Madagascar was obtained from Globcover project [33]. The GlobCover 2009 landscape product is a 300-m global landscape map produced from an automated classification of Medium Resolution Imaging Spectrometer (MERIS) time series. The global landscape map included 22 landscape classes defined with the United Nations (UN) Land Cover Classification System (LCCS). Among these 22 classes, we identified 5 relevant LCCS categories: “Cultivated Terrestrial Areas and Managed Lands” (so-called Crops), “Woody/ Trees”, “Shrubs”, “Herbaceous”, “Artificial Surfaces (so-called Urbanization)”. To reflect the availability of potential breeding habitats of RVF vectors in Madagascar such as artificial, irrigated, permanent and temporary water bodies, we needed to combine different data sources extracted from several GIS databases. The first one described inland permanent water point, such as lake, and was available from DIVA-GIS (http://www.diva-gis.org/). Marshland data representing temporary water bodies were obtained from Geographical Information Systems at the Royal Botanic Gardens, Kew [34]. Wetland locations representing temporary water bodies were extracted from the International Panel on Climate Change (IPCC; [35]). Irrigated area locations came from Global Map of Irrigation Areas (GMIA) from AQUASTAT-FAO [36].

#### Climatic variables: Precipitation, temperatures and NDVI

To depict the climatic conditions at each commune of Madagascar, day and night Land Surface Temperature (LST) and NDVI were retrieved from Moderate Resolution Imaging Spectroradiometer (MODIS; http://iridl.ldeo.columbia.edu/). For the period 2001 to 2010, day and night LST were extracted from MODIS data produced every 8 days at 1 km spatial resolution (MODIS MOD11A2 product: Land Surface Temperature and Emissivity). For the same period, NDVI data were obtained from MODIS data produced every 16 days at 250 m spatial resolution (MODIS MOD13A1: Vegetation Indices). Rainfall data were retrieved from the Tropical Rainfall Measuring Mission (TRMM; http://pmm.nasa.gov/trmm/mission-end). These data were produced at 25 km spatial resolution. Finally, for each commune and the same period, we computed the annual mean of day and night LST, NDVI and precipitation. Seasonality of NDVI and precipitation was also considered by computing the difference between the cumulated value over 3 months of the rainy season (November, December and January) and the cumulated value over 3 months of the dry season (June, July and August).

#### Human related factors

Human density was computed for each commune using data generated from Landscan 2007 Global Population Grid from Oak Ridge National Laboratory & the US Department of Defense (OCHA, 2007). Based on our field knowledge, the communes with more than 5,000 persons per square kilometer were considered as “urban”. Other communes were classified as “rural”. Human behaviors were documented through a dedicated questionnaire.

For each of the 1,578 communes considered, the percentage of surface covered by each landscape class (vegetation and water bodies), as well as the values of climatic, NDVI and cattle density covariates were computed with the Quantum GIS software [37]. Malagasy commune administrative boundaries and data come from the layers data merged by the Office for the Coordination of Humanitarian Affairs (OCHA) and based on data obtained from the Malagasy National Disaster Management Office in 2011.

### Multiple Factor Analysis

Synthetic variables characterizing the environment of communes were computed using a MFA combining the previously mentioned climatic and landscape variables [38,39]. By performing a factor analysis inside each variable category and then between categories, MFA produces a quantitative summary of the initial set of variables taking the form of a set of linear combination of variables, referred to as factors [39]. The climatic category included the annual means of day and night LST, the annual mean and seasonality of precipitation. The landscape category included the percentage of the surface of the commune covered by each landscape category and the annual mean and seasonality of NDVI. The value of each factor was computed for each of the 1,578 Malagasy communes. Correlation between MFA factor values and cattle density distribution was assessed using Pearson product-moment correlation coefficient test.

### Statistical analysis

As a first step univariate analyses of association between suspected risk factors and cattle or human RVFV serological status were undertaken using Chi square tests for categorical factors and generalized linear models for quantitative factors. Risk factors with significance level ≤0.20 were then included as explanatory variables in GLMMs, with cattle or human individual serological status as the binomial response. In these models, it was assumed that the relationships between serological prevalence and quantitative factors were linear on the logit scale. To account for interdependency of serological status of individuals sampled in the same locality, the smallest administrative unit—the commune for the cattle model and the city/village for human model- were included in the models as a random effect. Multicollinearity among explanatory variables was assessed using Variance Inflation Factors (VIF) and correlation tests. Collinear factors were not included in a same model. The selection of the best models was based on the Akaike Information Criterion (AIC). When needed, a multi-model inference approach was used to estimate model-averaged fixed effects (mafe) and the relative importance (RI) of each explanatory variable [40]. Within the set of models tested, only those with an AIC within 2 units difference from the best model were considered [40].

Internal validity of sets of models was evaluated using the Receiver Operating Characteristic (ROC) curve method [41].

In addition, we calculated the 10-fold cross-validation prediction. Because, it is not possible to perform 10-fold cross-validation on GLMM, this procedure was applied to Generalized Linear Models that were similar to the selected GLMM except that did not include the site of sampling as random effect. Firstly, the cattle seroprevalence dataset was split randomly into 10 parts. Then, the model was fitted to 90% of the data and used to predict the serological status of the remaining 10% individuals as validation step. The procedure was performed 10 times, each time with 1 of the 10 parts as validation step. [42].

Finally, parameter estimations derived from the best cattle model were used to predict and map cattle seroprevalence at the commune scale for the whole island.

Data analyses were performed using R software version 3.0.1 [43–49].

## Results

### Environmental characterization of Malagasy communes

**Fig 2:**
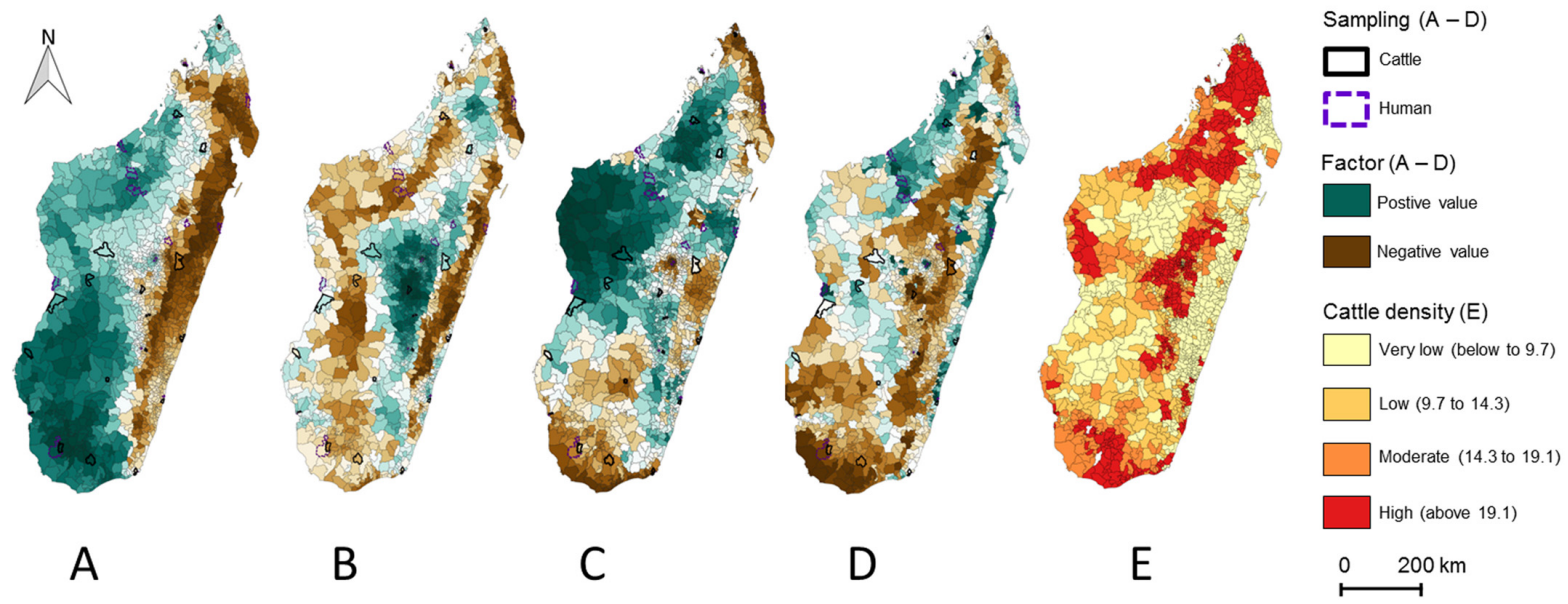
Geographical representation of the MFA factor values and cattle density of the 1,578 Malagasy communes. (A) Factor 1, (B) Factor 2, (C) Factor 3, (D) Factor 4, (E) cattle density categories. For each factor, green colors represent positive values and brown negative values. The darkest colors represent the highest values. Cattle were sampled in communes surrounded in black and human were enrolled in communes surrounded in purple.

Four MFA factors contributing to 60% of the total variance were selected. Table 1 shows the correlation between each quantitative covariate included in the MFA and each of these four factors:

- Factor 1 separated areas based on seasonality in primary productivity (photosynthetic activity measured by NDVI), vegetation, land use and temperature. Large positive values described ecosystems with high seasonal primary productivity dominated by herbaceous vegetation and with low surfaces of crops under dry and hot climatic conditions (Fig 2A in green). Large negative values described ecosystems with low seasonal primary productivity including crops under wet and less hot climatic conditions (Fig 2A in brown). The communes with the largest positive values for Factor1 are located in the south-western part of Madagascar (Fig 2A in green) while the communes with the largest negative values for Factor1 are located on the north-eastern part (Fig 2A in brown);
- Factor 2 separated areas based on seasonality in primary productivity, vegetation, land use and temperature. Large positive values described ecosystems with high seasonal primary productivity including ligneous vegetation and irrigated areas (rice fields) under climatic conditions characterized by low night temperatures (Fig 2B in green). Large negative values described ecosystems with low seasonal primary productivity including crops under climatic conditions characterized by warm night temperatures (Fig 2B in brown). The communes with the largest positive values of Factor 2 are located in the central highlands (Fig 2B in green). The communes with the largest negative values are mostly located in the eastern part of the island (Fig 2B in brown);
- Factor 3 was a rainfall seasonality index. The highest values of Factors3 (highly seasonal rainfall) are observed in the north-western part of the island (Fig 2C in green);
- Factor 4 represented a humid areas (marshlands, wetland and irrigated lands) index. The highest values are mostly located on the eastern-coast and the north-western part of the island (Fig 2D in green).

**Table 1:** Correlation between each quantitative covariate included in the MFA and each factor (Factor 1, Factor 2, Factor 3 and Factor 4).

Covariate	Factor 1	Factor 2	Factor 3	Factor 4
**Mean LST-day**	0.92	-0.19	0.11	/
**Mean LST-night**	0.50	-0.66	0.14	0.26
**Mean precipitation**	-0.70	/	0.32	0.31
**Seasonality of precipitation**	0.17	-0.15	0.82	0.09
**Mean NDVI**	-0.83	-0.34	/	/
**NDVI seasonality**	0.63	0.45	0.08	0.08
**Herbaceous**	0.84	-0.12	-0.24	0.11
**Shrubs**	0.11	0.40	0.30	-0.17
**Wood–Trees**	-0.33	0.56	0.37	-0.19
**Urbanization**	/	0.14	-0.30	0.27
**Crops**	-0.62	-0.61	-0.24	0.10
**Irrigated area**	/	0.66	-0.08	0.37
**Wetlands**	/	0.24	-0.39	0.46
**Water bodies**	/	/	0.07	0.22
**Marshlands**	/	0.07	0.18	0.71

/: The correlation coefficients were not significantly different from zero and so not included in the results

Considering each of the 1,578 communes of Madagascar, MFA factors values ranged from -1.9 to 3.3 (Factor 1), -1.9 to 2.8 (Factor 2), -5.1 to 2.7 (Factor 3) and -1.1 to 7.3 (Factor 4).

### Description of data and univariate statistical analysis (Table 2)

A total of 1,432 individuals from the initial cattle dataset, sampled in 26 communes belonging to 22 Malagasy districts were included in the study (Fig 1). The number of animals sampled per commune ranged from 1 to 110. Cattle ages ranged from 1 to 12 years (mean age 4.5 years). The overall seropositivity rate was 19.3% (CI 95% [17.3–21.8]). Age was categorized in 4 groups: 1–2, 3–4, 5–6 and more than 7 years old. Cattle density was classified as follows according to quartiles: below 9.7; 9.7 to 14.3; 14.3 to 19.1 and more than 19.1 per square kilometer. MFA factor values of the 26 sampling communes ranged from -1.7 to 2.6 (Factor 1), -0.9 to 1.5 (Factor 2), -1.5 to 2.3 (Factor 3) and -1.1 to 0.6 (Factor 4). Age category, cattle density category, Factor 1, Factor 3 and Factor 4 were statistically associated with cattle seroprevalence (p≤ 0.20). A total of 1,679 people were sampled, 91% (n = 1,529) living in rural areas and 9% (n = 150) living in urban areas (Fig 1). Age of volunteers ranged from 18 to 99 years (mean age 37.6 years) with a ratio of 1.03 (male/female). The overall seropositivity rate was 9.5% (95% CI [8.1–11.0]). Age was categorized in 4 groups: 18 to 26, 26 to 37, 37 to 46 and more than 46 years old. Cattle density of the related communes was classified as following: below 6.3; 6.3 to 11.7; 11.7 to 22.0 and more than 22.0 per square kilometer. A total of 267 individuals declared no contact with live animals or animal product and were categorized as “other profession”. Among them, 24 individuals were seropositive (9.0% 95% CI [5.8–13.1]). MFA factor values of the 48 communes ranged from -1.86 to 3.29 (Factor 1), -1.87 to 2.77 (Factor 2), -5.08 to 1.75 (Factor 3) and -0.75 to 4.51 (Factor 4). Habitat, gender, contact with ruminants, contact with raw milk, profession, age category, cattle density category, Factor 2, Factor 3 and Factor 4 were statistically associated with human seroprevalence (p≤ 0.20).

**Table 2:** Descriptive and univariate analyses for cattle and human seroprevalences.

Characteristics			Positive	Total	Seroprevalence [95% CI]	Chi2
**Cattle**	Age	1 to 2	46	353	13.0 [9.7–17.0]	p<0.001
		3 to 4	69	422	16.4 [13.0–20.2]	
		5 to 6	72	361	19.9 [15.9–24.4]	
		> 7	90	296	30.4 [25.2–36.0]	
	Cattle density per sq. km	< 9.7	69	359	19.2 [15.3–23.7]	p < 0.001
		9.7–14.3	55	357	15.4 [11.8–19.6]	
		14.3–19.1	37	362	10.2 [7.3–13.8]	
		> 19.1	116	354	32.8 [27.9–37.9]	
	Factor 1		/	/	/	p < 0.01
	Factor 2		/	/	/	p >0.20
	Factor 3		/	/	/	p < 0.10
	Factor 4		/	/	/	p < 0.10
	Total cattle		277	1432	15.9 [14.0–17.8]	/
**Human**	Habitat	Urban	9	150	6.0 [2.8–11.1]	p < 0.20
		Rural	150	1,529	9.8 [8.4–11.4]	
	Gender	F	50	851	5.9 [4.4–7.7]	p < 0.001
		M	109	828	13.2 [10.9–15.7]	
	Contact with ruminant	No	103	1,209	8.5 [7.0–10.2]	p < 0.05
		Yes	56	470	11.9 [9.1–15.2]	
	Contact with raw milk	No	140	1,576	8.9 [7.5–10.4]	p < 0.005
		Yes	19	103	18.4 [11.5–27.3]	
	Contact with fresh ruminant fluids	No	158	1,675	9.4 [8.1–10.9]	NA
		Yes	1	5	20 [0.1–71.6]	
	Profession	Butcher	1	6	16.7 [0.0–64.4]	p < 0.005
		Farmers	95	755	12.6 [10.3–15.2]	
		Health	1	19	5.3 [0.0–26.0]	
		Contact with environment	9	52	17.3 [8.2–30.3]	
		Others	53	847	6.3 [4.7–8.1]	
	Age	18 to 26	30	455	6.6 [4.5–9.3]	p < 0.05
		26 to 37	35	423	8.3 [5.8–11.3]	
		37 to 46	40	361	11.1 [8.0–14.8]	
		> 46	54	440	12.3 [9.4–15.7]	
	Cattle density per sq. km	< 6.3	51	450	11.3 [8.6–14.6]	p < 0.05
		6.3–11.7	51	420	12.1 [9.2–15.7]	
		11.7–22.0	28	389	7.2 [4.8–10.2]	
		> 22.0	29	420	6.9 [4.7–9.8]	
	Factor 1		/	/	/	p >0.20
	Factor 2		/	/	/	p < 0. 01
	Factor 3		/	/	/	p < 0. 2
	Factor 4		/	/	/	p < 0.10
	Total human		159	1,679	9.5 [8.1–11.0]	/

### Multivariate analysis

Both cattle and human seroprevalences increased gradually with age categories (Table 2). It was thus assumed that the relationship between age and seroprevalence was linear: age was thus included as a quantitative variable in multivariate models. Since the variation in cattle or human seroprevalence along cattle density categories was not clearly gradual, cattle density was included as a categorical variable in both cattle and human multivariate models. Cattle density was correlated with Factor 1, Factor 2 and Factor 3 and thus was included separately from Factor 1, Factor 2 and Factor 3 in both cattle and human multivariate models. The multicollinearity test did not detect any correlation between human related factors (VIF < 1.5).

For cattle, the single selected model (weight 0.99; S1 Table) included age, cattle density and Factor 4 as explanatory variables (S1 Table and Table 3). Factor 4 and age had a significant positive effect on seroprevalence (estimation of fixed effect at 1.73 and 0.17 respectively; p<0.001 for both explanatory variables; Table 3). Areas with high cattle density (> 19.1 per sq. km) were at risk (p<0.01; OR = 2.6 95% CI [1.3–5.4]; Table 3). According to AIC, seven models were considered as suitable for describing seroprevalence in humans and thus were analyzed using a multi-model inference approach (S1 Table). These models included age, Factor 2, Factor 3, Factor 4, gender, habitat, contact with raw milk, contact with fresh ruminant product, with live ruminant as explanatory variables (S1 Table and Table 4). Age, gender (male; OR = 2.3 95% CI [1.6–3.3]) and Factor 4 had a significant positive effect on seroprevalence (p<0.001, p<0.05 and p<0.05 respectively; Table 4). Factor 2 had a significant negative effect on seroprevalence (p<0.05) whereas Factor 3 had a minor importance in this set of models (RI = 0.27; Table 4). Contact with raw milk had a moderate effect on individual seroprevalence (OR = 1.6 95% CI [1.0–3.5]) whereas direct contacts ruminants and/or with fresh ruminant fluids, and habitat had a low impact on seroprevalence (RI = 0.12 or less; Table 4).

**Table 3:** Results from the best cattle model.

Variable		Estimate	95% CI	p-value
**Intercept**	/	-2.34	[-3.02–-1.72]	/
**Age**	/	0.17	[0.10–0.23]	p < 0.001
**Cattle density per sq. km**	< 6.3	Reference	/	/
** **	6.3–11.7	-0.24	[-1.01–0.54]	NS
** **	11.7–22.0	-0.66	[-1.61–0.24]	NS
** **	> 22.0	0.97	[0.30–1.69]	p < 0.01
**Factor 4**	/	1.73	[0.96–2.55]	p < 0.001

NS = not significant

**Table 4:** Results from the multi-model inference approach for human dataset analysis.

Variables	model-averaged fixed effects (mafe)	95% CI	p-value	Relative importance (RI)	Number of models
Age	0.02	[0.01–0.03]	0.001	1	7
Factor 2	-0.41	[-0.74–-0.09]	0.05	1	7
Factor 3	0.17	[-0.08–0.41]	NS	0.27	2
Factor 4	0.34	[0.08–0.61]	0.05	1	7
Gender	0.83	[0.52–1.14]	0.001	1	7
Contact with raw milk	0.60	[0.05–1.15]	NS	0.75	5
Contact with fresh ruminant fluids	1.04	[-1.26–3.36]	NS	0.12	1
Cattle density categories	/	/	NS	/	0
Profession	/	/	NS	/	0
Contact with ruminant	-0.07	[-0.44–0.29]	NS	0.10	1
Habitat	-0.42	[-1.42–0.57]	NS	0.12	1

NS = not significant

Internal validity of both cattle and human sets of models were satisfactory with an Area Under the Curve (AUC) of 0.82 (95% CI [0. 79–0.84]) and 0.80 (95% CI [0.77–0.84]) for cattle and human models respectively. The 10-fold cross-validation estimated an individual prediction error of about 14%.

**Fig 3:**
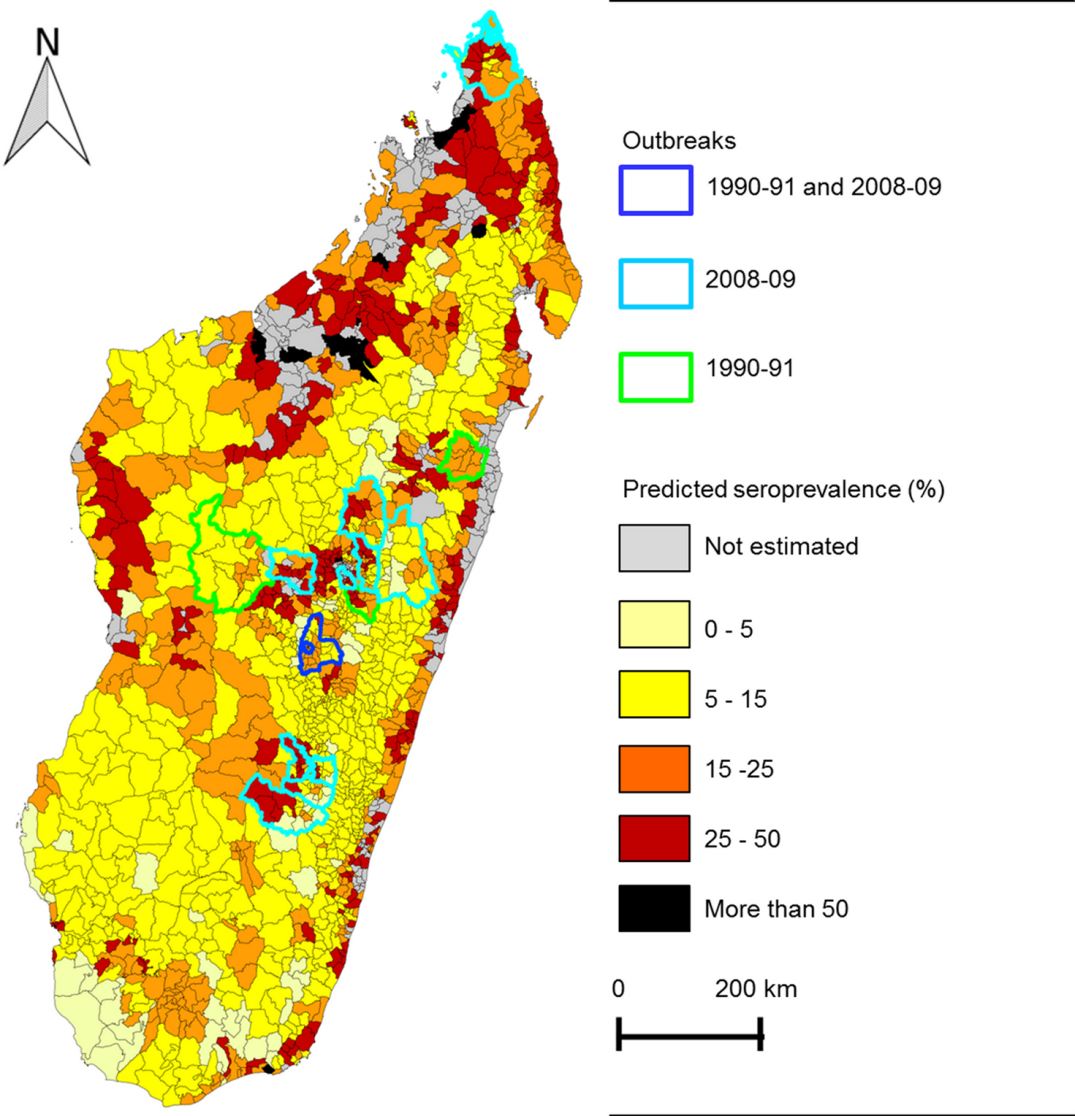
Predicted cattle seroprevalence in Madagascar and areas affected by RVF outbreaks in ruminant during 1990–1991 and 2008-2009. The cattle seroprevalence (SP) was predicted per commune and according to the best cattle model (Factor 4, cattle density and fixed age 5 years old).

Cattle seroprevalence was predicted according to Factor 4, cattle density categories and for a fixed cattle age of 5 years. To avoid biased estimations resulting from extrapolations, the prediction of seroprevalence was restricted to communes included in the range of the Factor 4 values corresponding to communes where cattle were sampled (i.e [-1.1–0.6]; n = 1,368). The prediction map highlights the western, north-western part and eastern-coast of Madagascar as high-risk areas for RVF transmission (Fig 3). Nineteen percent of the communes affected by outbreaks in ruminants during the 1990–91 and 2008–09 epizootics are located in areas with a predicted seroprevalence higher than 25%. Yet, 24% of the communes affected by these epizootics are located in low risk areas (predicted seroprevalence lower than 10%). Observed and predicted seroprevalence at the district level are compared in the S1 Appendix.

## Discussion

Following the 2008–09 epidemics, studies showed that RVFV spread widely but heterogeneously over Madagascar in both cattle and human populations [15,17]. This could be explained by the presence of ecosystems that are more or less suitable to the RVF candidate vector genera in Madagascar, including mosquitoes in the *Aedes*, *Anopheles*, *Culex*, *Eretmapodites* and *Mansonia* genera [25, 28]. Indeed, vector density and population dynamics are influenced by environmental factors such as climate and landscape features [1,25]. However, to date, environmental factors linked to the transmission of RVFV have never been investigated in Madagascar. To characterize Malagasy environments, we used MFA methods to generate environmental indicators that combined climatic, NDVI and landscape variables selected according to their putative influence on mosquito vector populations. Our MFA classification is in accordance with the known Malagasy ecosystems [18].

The risk of transmission and respective roles of direct and vectorial transmission are probably different among eco-climatic areas. In the case of direct transmission the force of infection is expected to depend on the number of potentially infectious contacts that a susceptible individual experiences over a time unit. This contact rate is expected to depend, among others, on cattle density. A positive association between cattle density and IgG seroprevalence rate in cattle and/or humans would thus suggest a direct transmission of RVFV. In the case of direct cattle to human transmission, the force of infection in the human population is also expected to depend on the frequency of human behaviors resulting in exposure to ruminant fluids or products. By contrast, in the case of vectorial transmission, due to the so-called “dilution effect” and for a fixed vector density, increased cattle density would decrease the probability for a susceptible individual to be bitten by an infectious vector over a time unit [50,51]. Therefore, a negative association between cattle density and IgG seroprevalence rates in cattle and/or in humans would rather suggest a vectorial transmission. The force of infection is also expected to increase with vector density. In Madagascar the density of vectors mostly depends on climatic and landscape factors [1,25,28]. High cattle densities are not systematically associated with high vector densities, as the main RVF vectors breed rather in large water bodies [25] than in artificial containers created from livestock-related activities. Thus, a positive association between cattle and/or human seroprevalence, local environmental and climatic conditions favorable to mosquitoes is expected under the hypothesis of vectorial transmission.

According to our analysis, cattle seroprevalence increased with age suggesting an enzootic circulation. Cattle seropositivity was positively associated to humid environment (large surface of permanent wetlands, marshlands and irrigated lands) each of these factors being favorable to *Culex* and *Anopheles* mosquitoes [25]. Actually, during the 2008–09 epidemics, 3 mosquito species were found to be naturally infected by RVFV: *Anopheles coustani*, *An*. *squamosus* and *Culex antennatus* [52]. *Cx*. *antennatus* is considered a RVFV vector and both *Anopheles* species as candidate vectors [25]. Cattle seroprevalence was also positively associated with cattle density suggesting the existence of a direct transmission between cattle, as suggested by Nicolas et al [3,19]. However, in our study cattle density and environmental factors were not independent (correlation with Factor 1, Factor 2 and Factor 3). Because of such associations it was impossible to disentangle the influence of cattle density from the influence of environmental conditions and thus to thoroughly assess the relative importance of vectorial and direct transmission. The prediction map of cattle seroprevalence highlighted the eastern-coast, western and north-western parts as high-risk areas. Surprisingly, some districts affected by RVFV outbreaks are located in the predicted low risk area [13–15]. The last outbreaks were mostly reported in the highlands, which are highly connected by road to the capital city, Antananarivo. Outbreaks occurring in isolated areas may have been missed explaining why a low proportion of outbreaks were located in predicted at-risk areas. On the other hand, enzootic transmission could have maintained a sufficient level of immunity in cattle in the high risk area restraining the outbreak magnitude in these regions. RVF could have been introduced in low risk areas through cattle trade and because of the low level of cattle immunity in these zones, trigger outbreaks. Nevertheless, as RVF cases were suspected to be under-reported, it was not possible to assess the relationship between the prediction of the herd immunity and the case notifications. Using satellite measurements (sea surface temperatures, rainfall and NDVI) and human cases as model output, Anyamba et al. [29] identified mainly the east-coast and some small areas of northern and north-western parts as at-risk for 2008–2009 RVF outbreaks in Madagascar. Considering that, during the 2008–09 outbreaks, several human cases occurred from the contact with infected fresh meat from traded ruminants [15], all the human infections could not be attributed to local infection [15]. Moreover, the detection of human cases depends on the intensity of the local circulation between ruminants and vectors, the probability of human exposure, the presence of clinical signs and the declaration to health services. Then, the human case data were probably not an optimum indicator of spatial distribution of RVF cases as suggested by Anyamba et al. [29]. Our prediction map is based on cattle for which the infection could be attributed to a local infection and identifies larger at-risk areas on western part of Madagascar than Anyamba et al. [29]. The discrepancy between results of Anyamba et al. study [29] and our study may be due the methodological differences: environmental variables included in both models and human clinical cases as model output on one side, bovine serological results on the other hand.

The estimated overall human seroprevalence was 9.5% (IC95% [8.2–11.0]). This seroprevalence is higher than adult seroprevalence observed in the island of Mayotte (2011) and Tanzania (2007–08) [24,53] but lower than adult seroprevalence in Kenya or Saudi Arabia [54,55]. Additionally, this seroprevalence is higher than the seroprevalence estimated for Madagascar in Gray et al [21]. The difference in the sampling area could explain this difference. Indeed, sera from the study of Gray et al. [21] were mainly sampled in south where RVF seroprevalence in human is low. Because of the different eco-epidemiological contexts and survey settings it is difficult to compare our results with the studies performed in Mayotte, Tanzanian, Kenya and Saudi Arabia [24, 53–55]. Human RVF seropositivity increased with age, suggesting an endemic transmission in human populations. As observed in cattle, human seropositivity was positively associated with the presence of temporary and artificial water points. In addition, 24 seropositive individuals declared no contact with ruminant or ruminant products, and the 3 mosquito species considered as potential vectors in Madagascar are zoo-anthropophilic feeders [25,52]: these results strongly suggest the existence of a vectorial transmission from ruminant to humans. Our analysis showed that frequent contact with raw milk contributed to explain human infection as previously suspected in Kenya [31]. Direct contact with fresh blood was not identified as human related risk factor whereas this way is suspected to be the main route of human infection in other studies [31]. In our sample, the number of people in contact with fresh blood was very low resulting in a low statistical power. However, this way of transmission has still to be considered, especially in the areas unfavorable to mosquitoes where direct contact could explain human infections [15].

Our integrated approach analyzing environmental, cattle and human datasets allow us to bring new insight on RVF transmission patterns in Madagascar. The association between cattle seroprevalence, humid environments and high cattle density suggests that concomitant vectorial and direct transmissions are critical to maintain RVFV enzootic transmission.

Even if the 2008–09 outbreaks are suspected to be associated with infected domestic animals imported from east Africa [56], our study confirms that enzootic and endemic circulations occur in Madagascar as suggested before [3,12,21].

The identification of at-risk environments is essential to focus veterinary surveillance and control of RVFV. Because of the variety of ecosystems and socio-cultural practices in Madagascar, it is likely that some areas are more favorable to direct transmission [3,19], while others are more favorable to vectorial transmission or to both transmission pathways. In the at-risk humid environment of the western, north-western and the eastern-coast areas, suitable for *Culex* and *Anopheles* mosquitoes, vectorial transmission probably occur in both cattle and human. In the future, mathematical modeling may be used to decipher the relative contribution of each transmission pathway in both human and ruminants, integrate the role of animal trade in disease spread in the Malagasy context, and thus propose adapted surveillance and control measures.

## Supporting Information

S1 Table Comparison of the values and weight of AIC for the cattle and human models. (DOCX)

S1 Appendix Scatterplot of observed versus predicted seroprevalences at the district level. Seroprevalence has been predicted for each age category in each communes sampled. For each district the sampling has been reconstructed taking into account the communes sampled and the number of animals sampled in each commune. Grey points correspond to districts where less than 5 animals were sampled.
(DOCX)
