# Correction: Integrated Analysis of Environment, Cattle and Human Serological Data: Risks and Mechanisms of Transmission of Rift Valley Fever in Madagascar

**DOI:** 10.1371/journal.pntd.0004976

**Published:** 2016-08-24

**Authors:** Marie-Marie Olive, Véronique Chevalier, Vladimir Grosbois, Annelise Tran, Soa-Fy Andriamandimby, Benoit Durand, Jean-Pierre Ravalohery, Seta Andriamamonjy, Fanjasoa Rakotomanana, Christophe Rogier, Jean-Michel Heraud

S1 File is omitted from the list of Supporting Information. It can be viewed below.

## Supporting Information

S1 File Supplementary dataset. This file includes supplementary data.
(DOCX)
