# Supplementary material for: Integrated Analysis of Environment, Cattle and Human Serological Data: Risks and Mechanisms of Transmission of Rift Valley Fever in Madagascar
Source: PLoS Negl Trop Dis. 2016 Jul 14;10(7):e0004827. doi: 10.1371/journal.pntd.0004827 (PMC4945045; doi:10.1371/journal.pntd.0004827)
Supplement: S1 Table — (DOCX) [file pntd.0004827.s001.docx]

**S1 Table: Comparison of the values and weight of AIC for the cattle and human models.**

| **Model** | **Variable included** | **AIC** | **weight** |
| --- | --- | --- | --- |
| **Cattle** | Age + Factor 4 + cattle density categories | 1203.2 | 0.99 |
|  | Age + Factor 1 + Factor 4 | 1214.6 | 0.00 |
|  | Age + Factor 4 | 1217.6 | 0.00 |
|  | Age + cattle density categories | 1218.5 | 0.00 |
|  | Age + Factor 3 + Factor 4 | 1218.6 | 0.00 |
|  | Age + Factor 1 + Factor 3 | 1227.7 | 0.00 |
|  | Age | 1230.0 | 0.00 |
| **Human** | Age + Factor 2 + Factor 4 +contact-raw milk + gender | 969.7 | 0.25 |
|  | Age + Factor 2 + Factor 3 + Factor 4 + contact-raw milk + gender | 970.4 | 0.17 |
|  | Age + Factor 2 + Factor 4 + gender | 970.7 | 0.15 |
|  | Age + Factor 2 + Factor 4 + contact-raw milk + gender + contact-fresh ruminant fluids | 971.2 | 0.12 |
|  | Age + Factor 2 + Factor 4 + contact-raw milk + gender + habitat | 971.2 | 0.12 |
|  | Age + Factor 2 + Factor 3 + Factor 4 + gender | 971.6 | 0.10 |
|  | Age + Factor 2 + Factor 4 contact-raw milk + gender + contact-ruminant | 971.6 | 0.10 |
