# Supplementary material for: Integrated Analysis of Environment, Cattle and Human Serological Data: Risks and Mechanisms of Transmission of Rift Valley Fever in Madagascar
Source: PLoS Negl Trop Dis. 2016 Jul 14;10(7):e0004827. doi: 10.1371/journal.pntd.0004827 (PMC4945045; doi:10.1371/journal.pntd.0004827)
Supplement: S1 Appendix — Seroprevalence has been predicted for each age category in each communes sampled. For each district the sampling has been reconstructed taking into account the communes sampled and the number of animals sampled in each commune. Grey points correspond to districts where less than 5 animals were sampled. (DOCX) [file pntd.0004827.s002.docx]

**S1 Appendix: Scatterplot of observed versus predicted seroprevalences at the district level**

Seroprevalence has been predicted for each age category in each communes sampled. For each district the sampling has been reconstructed taking into account the communes sampled and the number of animals sampled in each commune. Grey points correspond to districts where less than 5 animals were sampled.
