# Supplementary material for: Correction: Integrated Analysis of Environment, Cattle and Human Serological Data: Risks and Mechanisms of Transmission of Rift Valley Fever in Madagascar
Source: PLoS Negl Trop Dis. 2016 Aug 24;10(8):e0004976. doi: 10.1371/journal.pntd.0004976 (PMC4996515; doi:10.1371/journal.pntd.0004976)
Supplement: S1 File — This file includes supplementary data. (DOCX) [file pntd.0004976.s001.docx]

| ID | PCODE | site2 | sp | gender | age | IgG | contact_ruminant | contact_milk | contact_fresh_fluid | profession | fact1 | fact2 | fact3 | fact4 | cattle_density | habitat |
| --- | --- | --- | --- | --- | --- | --- | --- | --- | --- | --- | --- | --- | --- | --- | --- | --- |
| I-0030-CDA | MDG11101001 | CDAU | human | M | 28 | 0 | yes | no | no | contact_env | 1.057 | 2.770 | -5.079 | 4.512 | 11.6666666666666 | urban |
| I-0001-CDA | MDG11101001 | CDAU | human | M | 19 | 0 | no | no | no | other | 1.057 | 2.770 | -5.079 | 4.512 | 11.6666666666666 | urban |
| I-0029-CDA | MDG11101001 | CDAU | human | F | 24 | 0 | yes | no | no | other | 1.057 | 2.770 | -5.079 | 4.512 | 11.6666666666666 | urban |
| I-0028-CDA | MDG11101001 | CDAU | human | F | 43 | 0 | no | no | no | other | 1.057 | 2.770 | -5.079 | 4.512 | 11.6666666666666 | urban |
| I-0027-CDA | MDG11101001 | CDAU | human | M | 20 | 0 | no | no | no | other | 1.057 | 2.770 | -5.079 | 4.512 | 11.6666666666666 | urban |
| I-0026-CDA | MDG11101001 | CDAU | human | F | 36 | 0 | no | no | no | other | 1.057 | 2.770 | -5.079 | 4.512 | 11.6666666666666 | urban |
| I-0025-CDA | MDG11101001 | CDAU | human | F | 43 | 0 | no | no | no | other | 1.057 | 2.770 | -5.079 | 4.512 | 11.6666666666666 | urban |
| I-0024-CDA | MDG11101001 | CDAU | human | F | 24 | 0 | no | no | no | other | 1.057 | 2.770 | -5.079 | 4.512 | 11.6666666666666 | urban |
| I-0023-CDA | MDG11101001 | CDAU | human | F | 20 | 0 | no | no | no | other | 1.057 | 2.770 | -5.079 | 4.512 | 11.6666666666666 | urban |
| I-0022-CDA | MDG11101001 | CDAU | human | M | 40 | 1 | no | no | no | other | 1.057 | 2.770 | -5.079 | 4.512 | 11.6666666666666 | urban |
| I-0021-CDA | MDG11101001 | CDAU | human | F | 36 | 0 | no | no | no | other | 1.057 | 2.770 | -5.079 | 4.512 | 11.6666666666666 | urban |
| I-0020-CDA | MDG11101001 | CDAU | human | M | 23 | 0 | no | no | no | other | 1.057 | 2.770 | -5.079 | 4.512 | 11.6666666666666 | urban |
| I-0019-CDA | MDG11101001 | CDAU | human | M | 23 | 0 | no | no | no | other | 1.057 | 2.770 | -5.079 | 4.512 | 11.6666666666666 | urban |
| I-0018-CDA | MDG11101001 | CDAU | human | M | 30 | 0 | no | no | no | other | 1.057 | 2.770 | -5.079 | 4.512 | 11.6666666666666 | urban |
| I-0017-CDA | MDG11101001 | CDAU | human | F | 27 | 0 | no | no | no | other | 1.057 | 2.770 | -5.079 | 4.512 | 11.6666666666666 | urban |
| I-0006-CDA | MDG11101001 | CDAU | human | M | 80 | 1 | no | no | no | other | 1.057 | 2.770 | -5.079 | 4.512 | 11.6666666666666 | urban |
| I-0003-CDA | MDG11101001 | CDAU | human | M | 31 | 0 | no | no | no | other | 1.057 | 2.770 | -5.079 | 4.512 | 11.6666666666666 | urban |
| I-0016-CDA | MDG11101001 | CDAU | human | F | 66 | 0 | no | no | no | other | 1.057 | 2.770 | -5.079 | 4.512 | 11.6666666666666 | urban |
| I-0005-CDA | MDG11101001 | CDAU | human | M | 66 | 0 | no | no | no | other | 1.057 | 2.770 | -5.079 | 4.512 | 11.6666666666666 | urban |
| I-0002-CDA | MDG11101001 | CDAU | human | F | 24 | 0 | no | no | no | other | 1.057 | 2.770 | -5.079 | 4.512 | 11.6666666666666 | urban |
| I-0007-CDA | MDG11101001 | CDAU | human | F | 55 | 0 | no | no | no | other | 1.057 | 2.770 | -5.079 | 4.512 | 11.6666666666666 | urban |
| I-0008-CDA | MDG11101001 | CDAU | human | F | 34 | 0 | no | no | no | other | 1.057 | 2.770 | -5.079 | 4.512 | 11.6666666666666 | urban |
| I-0009-CDA | MDG11101001 | CDAU | human | F | 24 | 0 | yes | no | no | other | 1.057 | 2.770 | -5.079 | 4.512 | 11.6666666666666 | urban |
| I-0010-CDA | MDG11101001 | CDAU | human | M | 38 | 1 | no | no | no | other | 1.057 | 2.770 | -5.079 | 4.512 | 11.6666666666666 | urban |
| I-0011-CDA | MDG11101001 | CDAU | human | F | 56 | 0 | no | no | no | other | 1.057 | 2.770 | -5.079 | 4.512 | 11.6666666666666 | urban |
| I-0012-CDA | MDG11101001 | CDAU | human | F | 20 | 0 | no | no | no | other | 1.057 | 2.770 | -5.079 | 4.512 | 11.6666666666666 | urban |
| I-0013-CDA | MDG11101001 | CDAU | human | F | 27 | 0 | no | no | no | other | 1.057 | 2.770 | -5.079 | 4.512 | 11.6666666666666 | urban |
| I-0014-CDA | MDG11101001 | CDAU | human | M | 48 | 0 | no | no | no | other | 1.057 | 2.770 | -5.079 | 4.512 | 11.6666666666666 | urban |
| I-0015-CDA | MDG11101001 | CDAU | human | M | 84 | 0 | no | no | no | other | 1.057 | 2.770 | -5.079 | 4.512 | 11.6666666666666 | urban |
| I-0004-CDA | MDG11101001 | CDAU | human | F | 34 | 0 | no | no | no | other | 1.057 | 2.770 | -5.079 | 4.512 | 11.6666666666666 | urban |
| I-0045-AJB | MDG11107019 | AJBR | human | M | 75 | 0 | yes | no | no | farmer | -0.776 | 0.631 | 0.426 | -0.617 | 23.4166666666666 | rural |
| I-0044-AJB | MDG11107019 | AJBR | human | M | 22 | 0 | yes | no | no | farmer | -0.776 | 0.631 | 0.426 | -0.617 | 23.4166666666666 | rural |
| I-0043-AJB | MDG11107019 | AJBR | human | M | 58 | 0 | yes | no | no | farmer | -0.776 | 0.631 | 0.426 | -0.617 | 23.4166666666666 | rural |
| I-0042-AJB | MDG11107019 | AJBR | human | M | 26 | 0 | yes | no | no | farmer | -0.776 | 0.631 | 0.426 | -0.617 | 23.4166666666666 | rural |
| I-0041-AJB | MDG11107019 | AJBR | human | M | 50 | 0 | yes | no | no | farmer | -0.776 | 0.631 | 0.426 | -0.617 | 23.4166666666666 | rural |
| I-0040-AJB | MDG11107019 | AJBR | human | F | 46 | 1 | no | no | no | farmer | -0.776 | 0.631 | 0.426 | -0.617 | 23.4166666666666 | rural |
| I-0039-AJB | MDG11107019 | AJBR | human | M | 27 | 0 | yes | no | no | farmer | -0.776 | 0.631 | 0.426 | -0.617 | 23.4166666666666 | rural |
| I-0038-AJB | MDG11107019 | AJBR | human | F | 28 | 0 | no | no | no | farmer | -0.776 | 0.631 | 0.426 | -0.617 | 23.4166666666666 | rural |
| I-0037-AJB | MDG11107019 | AJBR | human | M | 29 | 0 | yes | no | no | farmer | -0.776 | 0.631 | 0.426 | -0.617 | 23.4166666666666 | rural |
| I-0036-AJB | MDG11107019 | AJBR | human | F | 29 | 0 | no | no | no | farmer | -0.776 | 0.631 | 0.426 | -0.617 | 23.4166666666666 | rural |
| I-0035-AJB | MDG11107019 | AJBR | human | M | 33 | 0 | yes | no | no | farmer | -0.776 | 0.631 | 0.426 | -0.617 | 23.4166666666666 | rural |
| I-0046-AJB | MDG11107019 | AJBR | human | F | 42 | 0 | yes | no | no | farmer | -0.776 | 0.631 | 0.426 | -0.617 | 23.4166666666666 | rural |
| I-0033-AJB | MDG11107019 | AJBR | human | M | 25 | 0 | yes | no | no | farmer | -0.776 | 0.631 | 0.426 | -0.617 | 23.4166666666666 | rural |
| I-0055-AJB | MDG11107019 | AJBR | human | F | 38 | 0 | no | no | no | farmer | -0.776 | 0.631 | 0.426 | -0.617 | 23.4166666666666 | rural |
| I-0034-AJB | MDG11107019 | AJBR | human | F | 54 | 0 | no | no | no | farmer | -0.776 | 0.631 | 0.426 | -0.617 | 23.4166666666666 | rural |
| I-0047-AJB | MDG11107019 | AJBR | human | M | 21 | 0 | yes | no | no | farmer | -0.776 | 0.631 | 0.426 | -0.617 | 23.4166666666666 | rural |
| I-0048-AJB | MDG11107019 | AJBR | human | M | 43 | 1 | yes | no | no | farmer | -0.776 | 0.631 | 0.426 | -0.617 | 23.4166666666666 | rural |
| I-0049-AJB | MDG11107019 | AJBR | human | F | 42 | 0 | yes | no | no | farmer | -0.776 | 0.631 | 0.426 | -0.617 | 23.4166666666666 | rural |
| I-0050-AJB | MDG11107019 | AJBR | human | M | 18 | 0 | yes | no | no | farmer | -0.776 | 0.631 | 0.426 | -0.617 | 23.4166666666666 | rural |
| I-0051-AJB | MDG11107019 | AJBR | human | M | 82 | 1 | yes | no | no | farmer | -0.776 | 0.631 | 0.426 | -0.617 | 23.4166666666666 | rural |
| I-0052-AJB | MDG11107019 | AJBR | human | F | 48 | 1 | yes | no | no | farmer | -0.776 | 0.631 | 0.426 | -0.617 | 23.4166666666666 | rural |
| I-0060-AJB | MDG11107019 | AJBR | human | F | 37 | 0 | no | no | no | farmer | -0.776 | 0.631 | 0.426 | -0.617 | 23.4166666666666 | rural |
| I-0054-AJB | MDG11107019 | AJBR | human | M | 42 | 1 | no | no | no | farmer | -0.776 | 0.631 | 0.426 | -0.617 | 23.4166666666666 | rural |
| I-0056-AJB | MDG11107019 | AJBR | human | M | 42 | 0 | yes | no | no | farmer | -0.776 | 0.631 | 0.426 | -0.617 | 23.4166666666666 | rural |
| I-0057-AJB | MDG11107019 | AJBR | human | F | 36 | 0 | yes | no | no | farmer | -0.776 | 0.631 | 0.426 | -0.617 | 23.4166666666666 | rural |
| I-0058-AJB | MDG11107019 | AJBR | human | F | 24 | 0 | no | no | no | farmer | -0.776 | 0.631 | 0.426 | -0.617 | 23.4166666666666 | rural |
| I-0059-AJB | MDG11107019 | AJBR | human | M | 53 | 1 | yes | no | no | farmer | -0.776 | 0.631 | 0.426 | -0.617 | 23.4166666666666 | rural |
| I-0032-AJB | MDG11107019 | AJBR | human | M | 52 | 1 | yes | no | no | farmer | -0.776 | 0.631 | 0.426 | -0.617 | 23.4166666666666 | rural |
| I-0022-AJB | MDG11107019 | AJBU | human | F | 39 | 0 | no | no | no | other | -0.776 | 0.631 | 0.426 | -0.617 | 23.4166666666666 | rural |
| I-0053-AJB | MDG11107019 | AJBR | human | M | 28 | 1 | no | no | no | farmer | -0.776 | 0.631 | 0.426 | -0.617 | 23.4166666666666 | rural |
| I-0007-AJB | MDG11107019 | AJBU | human | F | 25 | 0 | no | no | no | other | -0.776 | 0.631 | 0.426 | -0.617 | 23.4166666666666 | rural |
| I-0014-AJB | MDG11107019 | AJBU | human | F | 50 | 0 | yes | no | no | farmer | -0.776 | 0.631 | 0.426 | -0.617 | 23.4166666666666 | rural |
| I-0013-AJB | MDG11107019 | AJBU | human | F | 49 | 0 | no | no | no | farmer | -0.776 | 0.631 | 0.426 | -0.617 | 23.4166666666666 | rural |

| ID | PCODE | site2 | sp | gender | age | IgG | contact_ruminant | contact_milk | contact_fresh_fluid | profession | fact1 | fact2 | fact3 | fact4 | cattle_density | habitat |
| --- | --- | --- | --- | --- | --- | --- | --- | --- | --- | --- | --- | --- | --- | --- | --- | --- |
| I-0012-AJB | MDG11107019 | AJBU | human | F | 20 | 0 | no | no | no | other | -0.776 | 0.631 | 0.426 | -0.617 | 23.4166666666666 | rural |
| I-0011-AJB | MDG11107019 | AJBU | human | F | 30 | 0 | no | no | no | other | -0.776 | 0.631 | 0.426 | -0.617 | 23.4166666666666 | rural |
| I-0031-AJB | MDG11107019 | AJBR | human | F | 60 | 1 | yes | no | no | farmer | -0.776 | 0.631 | 0.426 | -0.617 | 23.4166666666666 | rural |
| I-0015-AJB | MDG11107019 | AJBU | human | M | 19 | 0 | no | no | no | other | -0.776 | 0.631 | 0.426 | -0.617 | 23.4166666666666 | rural |
| I-0024-AJB | MDG11107019 | AJBU | human | F | 80 | 0 | no | no | no | other | -0.776 | 0.631 | 0.426 | -0.617 | 23.4166666666666 | rural |
| I-0010-AJB | MDG11107019 | AJBU | human | F | 77 | 0 | no | no | no | farmer | -0.776 | 0.631 | 0.426 | -0.617 | 23.4166666666666 | rural |
| I-0006-AJB | MDG11107019 | AJBU | human | M | 23 | 0 | no | no | no | other | -0.776 | 0.631 | 0.426 | -0.617 | 23.4166666666666 | rural |
| I-0005-AJB | MDG11107019 | AJBU | human | F | 27 | 0 | no | no | no | farmer | -0.776 | 0.631 | 0.426 | -0.617 | 23.4166666666666 | rural |
| I-0004-AJB | MDG11107019 | AJBU | human | M | 22 | 0 | no | no | no | other | -0.776 | 0.631 | 0.426 | -0.617 | 23.4166666666666 | rural |
| I-0003-AJB | MDG11107019 | AJBU | human | M | 23 | 0 | no | no | no | other | -0.776 | 0.631 | 0.426 | -0.617 | 23.4166666666666 | rural |
| I-0002-AJB | MDG11107019 | AJBU | human | F | 39 | 0 | no | no | no | other | -0.776 | 0.631 | 0.426 | -0.617 | 23.4166666666666 | rural |
| I-0001-AJB | MDG11107019 | AJBU | human | M | 57 | 0 | no | no | no | other | -0.776 | 0.631 | 0.426 | -0.617 | 23.4166666666666 | rural |
| I-0009-AJB | MDG11107019 | AJBU | human | F | 25 | 0 | no | no | no | farmer | -0.776 | 0.631 | 0.426 | -0.617 | 23.4166666666666 | rural |
| I-0029-AJB | MDG11107019 | AJBU | human | M | 40 | 0 | no | no | no | farmer | -0.776 | 0.631 | 0.426 | -0.617 | 23.4166666666666 | rural |
| I-0008-AJB | MDG11107019 | AJBU | human | F | 53 | 0 | no | no | no | other | -0.776 | 0.631 | 0.426 | -0.617 | 23.4166666666666 | rural |
| I-0030-AJB | MDG11107019 | AJBU | human | F | 24 | 0 | no | no | no | other | -0.776 | 0.631 | 0.426 | -0.617 | 23.4166666666666 | rural |
| I-0016-AJB | MDG11107019 | AJBU | human | M | 18 | 0 | yes | no | no | other | -0.776 | 0.631 | 0.426 | -0.617 | 23.4166666666666 | rural |
| I-0028-AJB | MDG11107019 | AJBU | human | F | 36 | 0 | no | no | no | other | -0.776 | 0.631 | 0.426 | -0.617 | 23.4166666666666 | rural |
| I-0027-AJB | MDG11107019 | AJBU | human | F | 39 | 0 | no | no | no | other | -0.776 | 0.631 | 0.426 | -0.617 | 23.4166666666666 | rural |
| I-0026-AJB | MDG11107019 | AJBU | human | M | 64 | 0 | no | no | no | other | -0.776 | 0.631 | 0.426 | -0.617 | 23.4166666666666 | rural |
| I-0025-AJB | MDG11107019 | AJBU | human | F | 62 | 0 | no | no | no | other | -0.776 | 0.631 | 0.426 | -0.617 | 23.4166666666666 | rural |
| I-0023-AJB | MDG11107019 | AJBU | human | M | 35 | 0 | no | no | no | other | -0.776 | 0.631 | 0.426 | -0.617 | 23.4166666666666 | rural |
| I-0021-AJB | MDG11107019 | AJBU | human | F | 23 | 0 | no | no | no | farmer | -0.776 | 0.631 | 0.426 | -0.617 | 23.4166666666666 | rural |
| I-0020-AJB | MDG11107019 | AJBU | human | F | 42 | 0 | yes | no | no | farmer | -0.776 | 0.631 | 0.426 | -0.617 | 23.4166666666666 | rural |
| I-0019-AJB | MDG11107019 | AJBU | human | M | 19 | 0 | yes | no | no | farmer | -0.776 | 0.631 | 0.426 | -0.617 | 23.4166666666666 | rural |
| I-0018-AJB | MDG11107019 | AJBU | human | M | 19 | 1 | yes | no | no | other | -0.776 | 0.631 | 0.426 | -0.617 | 23.4166666666666 | rural |
| I-0017-AJB | MDG11107019 | AJBU | human | M | 19 | 0 | yes | no | no | other | -0.776 | 0.631 | 0.426 | -0.617 | 23.4166666666666 | rural |
| I-0028-ATB | MDG12108003 | ATBU | human | F | 66 | 0 | no | no | no | farmer | -0.019 | 1.609 | 0.329 | -0.422 | 48 | rural |
| I-0018-ATB | MDG12108003 | ATBU | human | M | 64 | 0 | no | no | no | other | -0.019 | 1.609 | 0.329 | -0.422 | 48 | rural |
| I-0019-ATB | MDG12108003 | ATBU | human | M | 31 | 0 | no | no | no | other | -0.019 | 1.609 | 0.329 | -0.422 | 48 | rural |
| I-0020-ATB | MDG12108003 | ATBU | human | F | 54 | 0 | no | no | no | farmer | -0.019 | 1.609 | 0.329 | -0.422 | 48 | rural |
| I-0021-ATB | MDG12108003 | ATBU | human | F | 63 | 0 | no | no | no | farmer | -0.019 | 1.609 | 0.329 | -0.422 | 48 | rural |
| I-0022-ATB | MDG12108003 | ATBU | human | F | 58 | 0 | yes | no | no | other | -0.019 | 1.609 | 0.329 | -0.422 | 48 | rural |
| I-0023-ATB | MDG12108003 | ATBU | human | M | 54 | 0 | yes | no | no | farmer | -0.019 | 1.609 | 0.329 | -0.422 | 48 | rural |
| I-0024-ATB | MDG12108003 | ATBU | human | F | 36 | 0 | no | no | no | other | -0.019 | 1.609 | 0.329 | -0.422 | 48 | rural |
| I-0025-ATB | MDG12108003 | ATBU | human | M | 21 | 0 | no | no | no | other | -0.019 | 1.609 | 0.329 | -0.422 | 48 | rural |
| I-0030-ATB | MDG12108003 | ATBU | human | M | 72 | 0 | no | no | no | farmer | -0.019 | 1.609 | 0.329 | -0.422 | 48 | rural |
| I-0027-ATB | MDG12108003 | ATBU | human | M | 61 | 0 | no | no | no | other | -0.019 | 1.609 | 0.329 | -0.422 | 48 | rural |
| I-0029-ATB | MDG12108003 | ATBU | human | F | 46 | 0 | no | no | no | other | -0.019 | 1.609 | 0.329 | -0.422 | 48 | rural |
| I-0016-ATB | MDG12108003 | ATBU | human | F | 63 | 0 | no | no | no | other | -0.019 | 1.609 | 0.329 | -0.422 | 48 | rural |
| I-0015-ATB | MDG12108003 | ATBU | human | M | 56 | 0 | no | no | no | other | -0.019 | 1.609 | 0.329 | -0.422 | 48 | rural |
| I-0026-ATB | MDG12108003 | ATBU | human | F | 20 | 0 | no | no | no | other | -0.019 | 1.609 | 0.329 | -0.422 | 48 | rural |
| I-0006-ATB | MDG12108003 | ATBU | human | M | 39 | 0 | no | no | no | other | -0.019 | 1.609 | 0.329 | -0.422 | 48 | rural |
| I-0001-ATB | MDG12108003 | ATBU | human | M | 39 | 0 | no | no | no | other | -0.019 | 1.609 | 0.329 | -0.422 | 48 | rural |
| I-0017-ATB | MDG12108003 | ATBU | human | M | 37 | 0 | no | no | no | other | -0.019 | 1.609 | 0.329 | -0.422 | 48 | rural |
| I-0003-ATB | MDG12108003 | ATBU | human | F | 22 | 0 | no | no | no | other | -0.019 | 1.609 | 0.329 | -0.422 | 48 | rural |
| I-0005-ATB | MDG12108003 | ATBU | human | F | 42 | 0 | no | no | no | other | -0.019 | 1.609 | 0.329 | -0.422 | 48 | rural |
| I-0002-ATB | MDG12108003 | ATBU | human | F | 48 | 0 | no | no | no | other | -0.019 | 1.609 | 0.329 | -0.422 | 48 | rural |
| I-0007-ATB | MDG12108003 | ATBU | human | M | 53 | 0 | no | no | no | other | -0.019 | 1.609 | 0.329 | -0.422 | 48 | rural |
| I-0008-ATB | MDG12108003 | ATBU | human | F | 31 | 0 | no | no | no | other | -0.019 | 1.609 | 0.329 | -0.422 | 48 | rural |
| I-0009-ATB | MDG12108003 | ATBU | human | F | 37 | 0 | no | no | no | other | -0.019 | 1.609 | 0.329 | -0.422 | 48 | rural |
| I-0010-ATB | MDG12108003 | ATBU | human | M | 44 | 0 | no | no | no | other | -0.019 | 1.609 | 0.329 | -0.422 | 48 | rural |
| I-0011-ATB | MDG12108003 | ATBU | human | F | 35 | 0 | no | no | no | other | -0.019 | 1.609 | 0.329 | -0.422 | 48 | rural |
| I-0012-ATB | MDG12108003 | ATBU | human | F | 53 | 0 | no | no | no | other | -0.019 | 1.609 | 0.329 | -0.422 | 48 | rural |
| I-0013-ATB | MDG12108003 | ATBU | human | M | 46 | 0 | no | no | no | other | -0.019 | 1.609 | 0.329 | -0.422 | 48 | rural |
| I-0014-ATB | MDG12108003 | ATBU | human | M | 69 | 0 | no | no | no | other | -0.019 | 1.609 | 0.329 | -0.422 | 48 | rural |
| I-0004-ATB | MDG12108003 | ATBU | human | F | 28 | 0 | no | no | no | other | -0.019 | 1.609 | 0.329 | -0.422 | 48 | rural |
| I-0045-TDD | MDG14111030 | TDDR | human | F | 18 | 0 | yes | no | no | farmer | 0.546 | 0.577 | 1.140 | -0.033 | 15.7333333333333 | rural |
| I-0044-TDD | MDG14111030 | TDDR | human | F | 61 | 0 | no | no | no | farmer | 0.546 | 0.577 | 1.140 | -0.033 | 15.7333333333333 | rural |
| I-0043-TDD | MDG14111030 | TDDR | human | M | 32 | 0 | yes | no | no | farmer | 0.546 | 0.577 | 1.140 | -0.033 | 15.7333333333333 | rural |
| I-0042-TDD | MDG14111030 | TDDR | human | M | 30 | 0 | yes | no | no | farmer | 0.546 | 0.577 | 1.140 | -0.033 | 15.7333333333333 | rural |
| I-0041-TDD | MDG14111030 | TDDR | human | F | 37 | 0 | no | no | no | farmer | 0.546 | 0.577 | 1.140 | -0.033 | 15.7333333333333 | rural |
| I-0040-TDD | MDG14111030 | TDDR | human | F | 30 | 0 | no | no | no | farmer | 0.546 | 0.577 | 1.140 | -0.033 | 15.7333333333333 | rural |

| ID | PCODE | site2 | sp | gender | age | IgG | contact_ruminant | contact_milk | contact_fresh_fluid | profession | fact1 | fact2 | fact3 | fact4 | cattle_density | habitat |
| --- | --- | --- | --- | --- | --- | --- | --- | --- | --- | --- | --- | --- | --- | --- | --- | --- |
| I-0039-TDD | MDG14111030 | TDDR | human | F | 53 | 0 | no | no | no | farmer | 0.546 | 0.577 | 1.140 | -0.033 | 15.7333333333333 | rural |
| I-0038-TDD | MDG14111030 | TDDR | human | M | 25 | 0 | yes | no | no | farmer | 0.546 | 0.577 | 1.140 | -0.033 | 15.7333333333333 | rural |
| I-0037-TDD | MDG14111030 | TDDR | human | F | 44 | 0 | yes | no | no | farmer | 0.546 | 0.577 | 1.140 | -0.033 | 15.7333333333333 | rural |
| I-0036-TDD | MDG14111030 | TDDR | human | M | 60 | 0 | yes | no | no | farmer | 0.546 | 0.577 | 1.140 | -0.033 | 15.7333333333333 | rural |
| I-0046-TDD | MDG14111030 | TDDR | human | M | 25 | 0 | no | no | no | farmer | 0.546 | 0.577 | 1.140 | -0.033 | 15.7333333333333 | rural |
| I-0034-TDD | MDG14111030 | TDDR | human | M | 58 | 1 | yes | yes | no | farmer | 0.546 | 0.577 | 1.140 | -0.033 | 15.7333333333333 | rural |
| I-0056-TDD | MDG14111030 | TDDR | human | F | 23 | 0 | no | yes | no | other | 0.546 | 0.577 | 1.140 | -0.033 | 15.7333333333333 | rural |
| I-0033-TDD | MDG14111030 | TDDR | human | M | 31 | 0 | yes | no | no | farmer | 0.546 | 0.577 | 1.140 | -0.033 | 15.7333333333333 | rural |
| I-0035-TDD | MDG14111030 | TDDR | human | M | 27 | 0 | no | no | no | farmer | 0.546 | 0.577 | 1.140 | -0.033 | 15.7333333333333 | rural |
| I-0047-TDD | MDG14111030 | TDDR | human | M | 61 | 0 | no | no | no | farmer | 0.546 | 0.577 | 1.140 | -0.033 | 15.7333333333333 | rural |
| I-0048-TDD | MDG14111030 | TDDR | human | M | 25 | 0 | yes | no | no | farmer | 0.546 | 0.577 | 1.140 | -0.033 | 15.7333333333333 | rural |
| I-0049-TDD | MDG14111030 | TDDR | human | F | 55 | 0 | no | no | no | farmer | 0.546 | 0.577 | 1.140 | -0.033 | 15.7333333333333 | rural |
| I-0050-TDD | MDG14111030 | TDDR | human | F | 34 | 0 | no | no | no | farmer | 0.546 | 0.577 | 1.140 | -0.033 | 15.7333333333333 | rural |
| I-0051-TDD | MDG14111030 | TDDR | human | M | 42 | 0 | no | no | no | farmer | 0.546 | 0.577 | 1.140 | -0.033 | 15.7333333333333 | rural |
| I-0052-TDD | MDG14111030 | TDDR | human | M | 24 | 0 | no | no | no | farmer | 0.546 | 0.577 | 1.140 | -0.033 | 15.7333333333333 | rural |
| I-0058-TDD | MDG14111030 | TDDR | human | F | 19 | 0 | yes | no | no | farmer | 0.546 | 0.577 | 1.140 | -0.033 | 15.7333333333333 | rural |
| I-0059-TDD | MDG14111030 | TDDR | human | M | 65 | 0 | no | no | no | farmer | 0.546 | 0.577 | 1.140 | -0.033 | 15.7333333333333 | rural |
| I-0060-TDD | MDG14111030 | TDDR | human | M | 25 | 0 | no | no | no | farmer | 0.546 | 0.577 | 1.140 | -0.033 | 15.7333333333333 | rural |
| I-0061-TDD | MDG14111030 | TDDU | human | F | 57 | 0 | no | no | no | other | 0.546 | 0.577 | 1.140 | -0.033 | 15.7333333333333 | rural |
| I-0062-TDD | MDG14111030 | TDDR | human | M | 56 | 1 | no | no | no | farmer | 0.546 | 0.577 | 1.140 | -0.033 | 15.7333333333333 | rural |
| I-0032-TDD | MDG14111030 | TDDR | human | M | 34 | 0 | no | no | no | farmer | 0.546 | 0.577 | 1.140 | -0.033 | 15.7333333333333 | rural |
| I-0055-TDD | MDG14111030 | TDDR | human | M | 22 | 0 | yes | yes | no | farmer | 0.546 | 0.577 | 1.140 | -0.033 | 15.7333333333333 | rural |
| I-0053-TDD | MDG14111030 | TDDR | human | F | 24 | 0 | no | no | no | farmer | 0.546 | 0.577 | 1.140 | -0.033 | 15.7333333333333 | rural |
| I-0057-TDD | MDG14111030 | TDDR | human | M | 38 | 0 | no | no | no | farmer | 0.546 | 0.577 | 1.140 | -0.033 | 15.7333333333333 | rural |
| I-0014-TDD | MDG14111030 | TDDU | human | M | 58 | 0 | no | no | no | other | 0.546 | 0.577 | 1.140 | -0.033 | 15.7333333333333 | rural |
| I-0013-TDD | MDG14111030 | TDDU | human | M | 26 | 0 | no | no | no | other | 0.546 | 0.577 | 1.140 | -0.033 | 15.7333333333333 | rural |
| I-0012-TDD | MDG14111030 | TDDU | human | M | 20 | 0 | no | yes | no | other | 0.546 | 0.577 | 1.140 | -0.033 | 15.7333333333333 | rural |
| I-0011-TDD | MDG14111030 | TDDU | human | M | 19 | 0 | no | no | no | other | 0.546 | 0.577 | 1.140 | -0.033 | 15.7333333333333 | rural |
| I-0010-TDD | MDG14111030 | TDDU | human | M | 27 | 0 | no | no | no | other | 0.546 | 0.577 | 1.140 | -0.033 | 15.7333333333333 | rural |
| I-0015-TDD | MDG14111030 | TDDU | human | F | 56 | 0 | no | no | no | other | 0.546 | 0.577 | 1.140 | -0.033 | 15.7333333333333 | rural |
| I-0007-TDD | MDG14111030 | TDDU | human | F | 37 | 0 | no | no | no | other | 0.546 | 0.577 | 1.140 | -0.033 | 15.7333333333333 | rural |
| I-0009-TDD | MDG14111030 | TDDU | human | M | 22 | 0 | no | no | no | other | 0.546 | 0.577 | 1.140 | -0.033 | 15.7333333333333 | rural |
| I-0006-TDD | MDG14111030 | TDDU | human | M | 33 | 0 | no | no | no | other | 0.546 | 0.577 | 1.140 | -0.033 | 15.7333333333333 | rural |
| I-0005-TDD | MDG14111030 | TDDU | human | M | 28 | 0 | yes | no | no | farmer | 0.546 | 0.577 | 1.140 | -0.033 | 15.7333333333333 | rural |
| I-0004-TDD | MDG14111030 | TDDU | human | M | 55 | 0 | no | no | no | farmer | 0.546 | 0.577 | 1.140 | -0.033 | 15.7333333333333 | rural |
| I-0003-TDD | MDG14111030 | TDDU | human | F | 22 | 0 | no | no | no | other | 0.546 | 0.577 | 1.140 | -0.033 | 15.7333333333333 | rural |
| I-0002-TDD | MDG14111030 | TDDU | human | M | 29 | 0 | no | no | no | other | 0.546 | 0.577 | 1.140 | -0.033 | 15.7333333333333 | rural |
| I-0031-TDD | MDG14111030 | TDDR | human | M | 36 | 1 | no | no | no | farmer | 0.546 | 0.577 | 1.140 | -0.033 | 15.7333333333333 | rural |
| I-0008-TDD | MDG14111030 | TDDU | human | F | 63 | 0 | no | no | no | other | 0.546 | 0.577 | 1.140 | -0.033 | 15.7333333333333 | rural |
| I-0028-TDD | MDG14111030 | TDDU | human | M | 24 | 0 | yes | no | yes | other | 0.546 | 0.577 | 1.140 | -0.033 | 15.7333333333333 | rural |
| I-0001-TDD | MDG14111030 | TDDU | human | M | 62 | 0 | no | no | no | other | 0.546 | 0.577 | 1.140 | -0.033 | 15.7333333333333 | rural |
| I-0029-TDD | MDG14111030 | TDDU | human | M | 43 | 0 | yes | no | no | farmer | 0.546 | 0.577 | 1.140 | -0.033 | 15.7333333333333 | rural |
| I-0016-TDD | MDG14111030 | TDDU | human | M | 23 | 0 | no | no | no | farmer | 0.546 | 0.577 | 1.140 | -0.033 | 15.7333333333333 | rural |
| I-0027-TDD | MDG14111030 | TDDU | human | F | 34 | 0 | no | no | no | other | 0.546 | 0.577 | 1.140 | -0.033 | 15.7333333333333 | rural |
| I-0026-TDD | MDG14111030 | TDDU | human | F | 25 | 0 | no | no | no | other | 0.546 | 0.577 | 1.140 | -0.033 | 15.7333333333333 | rural |
| I-0025-TDD | MDG14111030 | TDDU | human | F | 41 | 0 | no | no | no | farmer | 0.546 | 0.577 | 1.140 | -0.033 | 15.7333333333333 | rural |
| I-0024-TDD | MDG14111030 | TDDU | human | M | 39 | 0 | no | no | no | other | 0.546 | 0.577 | 1.140 | -0.033 | 15.7333333333333 | rural |
| I-0023-TDD | MDG14111030 | TDDU | human | F | 25 | 0 | no | no | no | other | 0.546 | 0.577 | 1.140 | -0.033 | 15.7333333333333 | rural |
| I-0022-TDD | MDG14111030 | TDDU | human | M | 44 | 0 | no | no | no | other | 0.546 | 0.577 | 1.140 | -0.033 | 15.7333333333333 | rural |
| I-0021-TDD | MDG14111030 | TDDU | human | F | 45 | 1 | no | no | no | other | 0.546 | 0.577 | 1.140 | -0.033 | 15.7333333333333 | rural |
| I-0020-TDD | MDG14111030 | TDDU | human | F | 27 | 0 | no | no | no | other | 0.546 | 0.577 | 1.140 | -0.033 | 15.7333333333333 | rural |
| I-0018-TDD | MDG14111030 | TDDU | human | M | 20 | 0 | no | no | no | other | 0.546 | 0.577 | 1.140 | -0.033 | 15.7333333333333 | rural |
| I-0017-TDD | MDG14111030 | TDDU | human | M | 22 | 0 | no | no | no | other | 0.546 | 0.577 | 1.140 | -0.033 | 15.7333333333333 | rural |
| I-0030-TDD | MDG14111030 | TDDU | human | M | 67 | 0 | yes | no | no | farmer | 0.546 | 0.577 | 1.140 | -0.033 | 15.7333333333333 | rural |
| I-0058-CDA | MDG11117250 | CDAR | human | F | 22 | 0 | no | no | no | farmer | 1.061 | 1.123 | -1.948 | 1.185 | 25.5 | rural |
| I-0048-CDA | MDG11117250 | CDAR | human | F | 52 | 0 | no | no | no | farmer | 1.061 | 1.123 | -1.948 | 1.185 | 25.5 | rural |
| I-0049-CDA | MDG11117250 | CDAR | human | F | 73 | 0 | yes | no | no | farmer | 1.061 | 1.123 | -1.948 | 1.185 | 25.5 | rural |
| I-0050-CDA | MDG11117250 | CDAR | human | M | 76 | 0 | yes | no | no | farmer | 1.061 | 1.123 | -1.948 | 1.185 | 25.5 | rural |
| I-0051-CDA | MDG11117250 | CDAR | human | M | 19 | 0 | yes | no | no | farmer | 1.061 | 1.123 | -1.948 | 1.185 | 25.5 | rural |
| I-0052-CDA | MDG11117250 | CDAR | human | F | 32 | 0 | yes | no | no | farmer | 1.061 | 1.123 | -1.948 | 1.185 | 25.5 | rural |
| I-0053-CDA | MDG11117250 | CDAR | human | M | 32 | 0 | yes | no | no | farmer | 1.061 | 1.123 | -1.948 | 1.185 | 25.5 | rural |
| I-0054-CDA | MDG11117250 | CDAR | human | M | 29 | 0 | no | no | no | other | 1.061 | 1.123 | -1.948 | 1.185 | 25.5 | rural |
| I-0055-CDA | MDG11117250 | CDAR | human | F | 35 | 0 | no | no | no | other | 1.061 | 1.123 | -1.948 | 1.185 | 25.5 | rural |

| ID | PCODE | site2 | sp | gender | age | IgG | contact_ruminant | contact_milk | contact_fresh_fluid | profession | fact1 | fact2 | fact3 | fact4 | cattle_density | habitat |
| --- | --- | --- | --- | --- | --- | --- | --- | --- | --- | --- | --- | --- | --- | --- | --- | --- |
| I-0060-CDA | MDG11117250 | CDAR | human | F | 51 | 0 | yes | no | no | farmer | 1.061 | 1.123 | -1.948 | 1.185 | 25.5 | rural |
| I-0057-CDA | MDG11117250 | CDAR | human | M | 29 | 0 | yes | no | no | farmer | 1.061 | 1.123 | -1.948 | 1.185 | 25.5 | rural |
| I-0046-CDA | MDG11117250 | CDAR | human | F | 34 | 0 | no | no | no | farmer | 1.061 | 1.123 | -1.948 | 1.185 | 25.5 | rural |
| I-0059-CDA | MDG11117250 | CDAR | human | F | 48 | 0 | no | no | no | farmer | 1.061 | 1.123 | -1.948 | 1.185 | 25.5 | rural |
| I-0045-CDA | MDG11117250 | CDAR | human | F | 41 | 0 | no | no | no | farmer | 1.061 | 1.123 | -1.948 | 1.185 | 25.5 | rural |
| I-0056-CDA | MDG11117250 | CDAR | human | F | 26 | 0 | no | no | no | other | 1.061 | 1.123 | -1.948 | 1.185 | 25.5 | rural |
| I-0032-CDA | MDG11117250 | CDAR | human | F | 33 | 0 | no | no | no | other | 1.061 | 1.123 | -1.948 | 1.185 | 25.5 | rural |
| I-0047-CDA | MDG11117250 | CDAR | human | F | 22 | 0 | no | no | no | other | 1.061 | 1.123 | -1.948 | 1.185 | 25.5 | rural |
| I-0033-CDA | MDG11117250 | CDAR | human | F | 49 | 0 | no | no | no | other | 1.061 | 1.123 | -1.948 | 1.185 | 25.5 | rural |
| I-0034-CDA | MDG11117250 | CDAR | human | M | 64 | 0 | no | no | no | other | 1.061 | 1.123 | -1.948 | 1.185 | 25.5 | rural |
| I-0035-CDA | MDG11117250 | CDAR | human | F | 37 | 0 | no | no | no | contact_env | 1.061 | 1.123 | -1.948 | 1.185 | 25.5 | rural |
| I-0036-CDA | MDG11117250 | CDAR | human | F | 23 | 0 | yes | no | no | other | 1.061 | 1.123 | -1.948 | 1.185 | 25.5 | rural |
| I-0037-CDA | MDG11117250 | CDAR | human | F | 59 | 0 | yes | no | no | farmer | 1.061 | 1.123 | -1.948 | 1.185 | 25.5 | rural |
| I-0038-CDA | MDG11117250 | CDAR | human | M | 19 | 0 | no | no | no | other | 1.061 | 1.123 | -1.948 | 1.185 | 25.5 | rural |
| I-0040-CDA | MDG11117250 | CDAR | human | F | 27 | 0 | no | no | no | farmer | 1.061 | 1.123 | -1.948 | 1.185 | 25.5 | rural |
| I-0041-CDA | MDG11117250 | CDAR | human | M | 31 | 0 | no | no | no | contact_env | 1.061 | 1.123 | -1.948 | 1.185 | 25.5 | rural |
| I-0042-CDA | MDG11117250 | CDAR | human | F | 31 | 0 | no | no | no | contact_env | 1.061 | 1.123 | -1.948 | 1.185 | 25.5 | rural |
| I-0043-CDA | MDG11117250 | CDAR | human | F | 48 | 0 | no | no | no | other | 1.061 | 1.123 | -1.948 | 1.185 | 25.5 | rural |
| I-0044-CDA | MDG11117250 | CDAR | human | M | 45 | 0 | no | no | no | farmer | 1.061 | 1.123 | -1.948 | 1.185 | 25.5 | rural |
| I-0031-CDA | MDG11117250 | CDAR | human | F | 20 | 0 | no | no | no | other | 1.061 | 1.123 | -1.948 | 1.185 | 25.5 | rural |
| I-0039-CDA | MDG11117250 | CDAR | human | M | 55 | 0 | no | no | no | other | 1.061 | 1.123 | -1.948 | 1.185 | 25.5 | rural |
| I-0054-ATB | MDG12118110 | ATBR | human | F | 57 | 0 | yes | no | no | farmer | -0.371 | 1.853 | 0.170 | -0.577 | 45.0833333333333 | rural |
| I-0049-ATB | MDG12118110 | ATBR | human | F | 31 | 0 | yes | no | no | farmer | -0.371 | 1.853 | 0.170 | -0.577 | 45.0833333333333 | rural |
| I-0050-ATB | MDG12118110 | ATBR | human | F | 21 | 0 | yes | no | no | farmer | -0.371 | 1.853 | 0.170 | -0.577 | 45.0833333333333 | rural |
| I-0051-ATB | MDG12118110 | ATBR | human | M | 22 | 1 | no | no | no | other | -0.371 | 1.853 | 0.170 | -0.577 | 45.0833333333333 | rural |
| I-0052-ATB | MDG12118110 | ATBR | human | F | 27 | 0 | no | no | no | farmer | -0.371 | 1.853 | 0.170 | -0.577 | 45.0833333333333 | rural |
| I-0048-ATB | MDG12118110 | ATBR | human | M | 33 | 0 | yes | no | no | farmer | -0.371 | 1.853 | 0.170 | -0.577 | 45.0833333333333 | rural |
| I-0053-ATB | MDG12118110 | ATBR | human | M | 27 | 0 | no | no | no | farmer | -0.371 | 1.853 | 0.170 | -0.577 | 45.0833333333333 | rural |
| I-0055-ATB | MDG12118110 | ATBR | human | M | 62 | 0 | yes | no | no | farmer | -0.371 | 1.853 | 0.170 | -0.577 | 45.0833333333333 | rural |
| I-0056-ATB | MDG12118110 | ATBR | human | M | 41 | 0 | yes | no | no | farmer | -0.371 | 1.853 | 0.170 | -0.577 | 45.0833333333333 | rural |
| I-0057-ATB | MDG12118110 | ATBR | human | F | 37 | 0 | yes | no | no | other | -0.371 | 1.853 | 0.170 | -0.577 | 45.0833333333333 | rural |
| I-0058-ATB | MDG12118110 | ATBR | human | F | 62 | 0 | yes | no | no | farmer | -0.371 | 1.853 | 0.170 | -0.577 | 45.0833333333333 | rural |
| I-0059-ATB | MDG12118110 | ATBR | human | M | 54 | 0 | no | no | no | farmer | -0.371 | 1.853 | 0.170 | -0.577 | 45.0833333333333 | rural |
| I-0038-ATB | MDG12118110 | ATBR | human | M | 24 | 0 | no | no | no | farmer | -0.371 | 1.853 | 0.170 | -0.577 | 45.0833333333333 | rural |
| I-0047-ATB | MDG12118110 | ATBR | human | M | 27 | 0 | yes | no | no | farmer | -0.371 | 1.853 | 0.170 | -0.577 | 45.0833333333333 | rural |
| I-0060-ATB | MDG12118110 | ATBR | human | F | 51 | 0 | yes | no | no | farmer | -0.371 | 1.853 | 0.170 | -0.577 | 45.0833333333333 | rural |
| I-0036-ATB | MDG12118110 | ATBR | human | F | 41 | 0 | yes | no | no | farmer | -0.371 | 1.853 | 0.170 | -0.577 | 45.0833333333333 | rural |
| I-0040-ATB | MDG12118110 | ATBR | human | M | 65 | 0 | yes | no | no | farmer | -0.371 | 1.853 | 0.170 | -0.577 | 45.0833333333333 | rural |
| I-0032-ATB | MDG12118110 | ATBR | human | M | 23 | 0 | no | no | no | farmer | -0.371 | 1.853 | 0.170 | -0.577 | 45.0833333333333 | rural |
| I-0033-ATB | MDG12118110 | ATBR | human | M | 38 | 0 | no | no | no | farmer | -0.371 | 1.853 | 0.170 | -0.577 | 45.0833333333333 | rural |
| I-0046-ATB | MDG12118110 | ATBR | human | M | 22 | 0 | no | no | no | farmer | -0.371 | 1.853 | 0.170 | -0.577 | 45.0833333333333 | rural |
| I-0035-ATB | MDG12118110 | ATBR | human | M | 49 | 0 | yes | no | no | other | -0.371 | 1.853 | 0.170 | -0.577 | 45.0833333333333 | rural |
| I-0031-ATB | MDG12118110 | ATBR | human | M | 40 | 0 | no | no | no | farmer | -0.371 | 1.853 | 0.170 | -0.577 | 45.0833333333333 | rural |
| I-0037-ATB | MDG12118110 | ATBR | human | M | 39 | 0 | no | no | no | farmer | -0.371 | 1.853 | 0.170 | -0.577 | 45.0833333333333 | rural |
| I-0039-ATB | MDG12118110 | ATBR | human | M | 59 | 0 | no | no | no | farmer | -0.371 | 1.853 | 0.170 | -0.577 | 45.0833333333333 | rural |
| I-0041-ATB | MDG12118110 | ATBR | human | M | 42 | 0 | yes | no | no | farmer | -0.371 | 1.853 | 0.170 | -0.577 | 45.0833333333333 | rural |
| I-0042-ATB | MDG12118110 | ATBR | human | F | 43 | 0 | yes | no | no | farmer | -0.371 | 1.853 | 0.170 | -0.577 | 45.0833333333333 | rural |
| I-0043-ATB | MDG12118110 | ATBR | human | M | 30 | 0 | no | yes | no | other | -0.371 | 1.853 | 0.170 | -0.577 | 45.0833333333333 | rural |
| I-0044-ATB | MDG12118110 | ATBR | human | F | 39 | 0 | no | no | no | farmer | -0.371 | 1.853 | 0.170 | -0.577 | 45.0833333333333 | rural |
| I-0045-ATB | MDG12118110 | ATBR | human | F | 19 | 0 | no | no | no | farmer | -0.371 | 1.853 | 0.170 | -0.577 | 45.0833333333333 | rural |
| I-0034-ATB | MDG12118110 | ATBR | human | M | 21 | 0 | yes | no | no | farmer | -0.371 | 1.853 | 0.170 | -0.577 | 45.0833333333333 | rural |
| I-0028-FIA | MDG21201001 | FIAU | human | F | 31 | 0 | no | no | no | other | 0.038 | 0.806 | 0.057 | -0.081 | 13.5 | urban |
| I-0018-FIA | MDG21201001 | FIAU | human | M | 48 | 0 | no | no | no | other | 0.038 | 0.806 | 0.057 | -0.081 | 13.5 | urban |
| I-0019-FIA | MDG21201001 | FIAU | human | F | 26 | 0 | no | no | no | other | 0.038 | 0.806 | 0.057 | -0.081 | 13.5 | urban |
| I-0020-FIA | MDG21201001 | FIAU | human | F | 47 | 0 | no | no | no | other | 0.038 | 0.806 | 0.057 | -0.081 | 13.5 | urban |
| I-0021-FIA | MDG21201001 | FIAU | human | M | 29 | 0 | no | no | no | other | 0.038 | 0.806 | 0.057 | -0.081 | 13.5 | urban |
| I-0022-FIA | MDG21201001 | FIAU | human | F | 34 | 1 | no | no | no | other | 0.038 | 0.806 | 0.057 | -0.081 | 13.5 | urban |
| I-0023-FIA | MDG21201001 | FIAU | human | M | 38 | 0 | no | no | no | other | 0.038 | 0.806 | 0.057 | -0.081 | 13.5 | urban |
| I-0024-FIA | MDG21201001 | FIAU | human | F | 50 | 0 | no | no | no | other | 0.038 | 0.806 | 0.057 | -0.081 | 13.5 | urban |
| I-0025-FIA | MDG21201001 | FIAU | human | F | 21 | 0 | no | no | no | other | 0.038 | 0.806 | 0.057 | -0.081 | 13.5 | urban |
| I-0027-FIA | MDG21201001 | FIAU | human | M | 67 | 0 | no | no | no | other | 0.038 | 0.806 | 0.057 | -0.081 | 13.5 | urban |
| I-0017-FIA | MDG21201001 | FIAU | human | M | 19 | 0 | yes | no | no | other | 0.038 | 0.806 | 0.057 | -0.081 | 13.5 | urban |
| I-0029-FIA | MDG21201001 | FIAU | human | M | 25 | 0 | no | no | no | other | 0.038 | 0.806 | 0.057 | -0.081 | 13.5 | urban |

| ID | PCODE | site2 | sp | gender | age | IgG | contact_ruminant | contact_milk | contact_fresh_fluid | profession | fact1 | fact2 | fact3 | fact4 | cattle_density | habitat |
| --- | --- | --- | --- | --- | --- | --- | --- | --- | --- | --- | --- | --- | --- | --- | --- | --- |
| I-0030-FIA | MDG21201001 | FIAU | human | M | 35 | 0 | no | no | no | contact_env | 0.038 | 0.806 | 0.057 | -0.081 | 13.5 | urban |
| I-0006-FIA | MDG21201001 | FIAU | human | M | 42 | 0 | no | no | no | farmer | 0.038 | 0.806 | 0.057 | -0.081 | 13.5 | urban |
| I-0026-FIA | MDG21201001 | FIAU | human | M | 31 | 0 | no | no | no | other | 0.038 | 0.806 | 0.057 | -0.081 | 13.5 | urban |
| I-0002-FIA | MDG21201001 | FIAU | human | F | 45 | 0 | no | no | no | other | 0.038 | 0.806 | 0.057 | -0.081 | 13.5 | urban |
| I-0008-FIA | MDG21201001 | FIAU | human | M | 72 | 0 | yes | no | no | other | 0.038 | 0.806 | 0.057 | -0.081 | 13.5 | urban |
| I-0001-FIA | MDG21201001 | FIAU | human | M | 25 | 0 | no | no | no | other | 0.038 | 0.806 | 0.057 | -0.081 | 13.5 | urban |
| I-0016-FIA | MDG21201001 | FIAU | human | F | 35 | 0 | no | no | no | other | 0.038 | 0.806 | 0.057 | -0.081 | 13.5 | urban |
| I-0003-FIA | MDG21201001 | FIAU | human | M | 19 | 0 | no | no | no | other | 0.038 | 0.806 | 0.057 | -0.081 | 13.5 | urban |
| I-0004-FIA | MDG21201001 | FIAU | human | F | 37 | 0 | no | no | no | other | 0.038 | 0.806 | 0.057 | -0.081 | 13.5 | urban |
| I-0005-FIA | MDG21201001 | FIAU | human | M | 26 | 0 | no | no | no | other | 0.038 | 0.806 | 0.057 | -0.081 | 13.5 | urban |
| I-0007-FIA | MDG21201001 | FIAU | human | F | 66 | 0 | no | no | no | other | 0.038 | 0.806 | 0.057 | -0.081 | 13.5 | urban |
| I-0009-FIA | MDG21201001 | FIAU | human | M | 38 | 0 | no | yes | no | farmer | 0.038 | 0.806 | 0.057 | -0.081 | 13.5 | urban |
| I-0010-FIA | MDG21201001 | FIAU | human | M | 46 | 0 | no | no | no | farmer | 0.038 | 0.806 | 0.057 | -0.081 | 13.5 | urban |
| I-0011-FIA | MDG21201001 | FIAU | human | M | 58 | 0 | no | no | no | other | 0.038 | 0.806 | 0.057 | -0.081 | 13.5 | urban |
| I-0012-FIA | MDG21201001 | FIAU | human | F | 46 | 0 | no | no | no | other | 0.038 | 0.806 | 0.057 | -0.081 | 13.5 | urban |
| I-0013-FIA | MDG21201001 | FIAU | human | F | 57 | 0 | no | no | no | other | 0.038 | 0.806 | 0.057 | -0.081 | 13.5 | urban |
| I-0014-FIA | MDG21201001 | FIAU | human | F | 31 | 0 | no | no | no | other | 0.038 | 0.806 | 0.057 | -0.081 | 13.5 | urban |
| I-0015-FIA | MDG21201001 | FIAU | human | M | 31 | 0 | no | no | no | other | 0.038 | 0.806 | 0.057 | -0.081 | 13.5 | urban |
| I-0022-BOS | MDG22203010 | BOSU | human | M | 26 | 0 | yes | no | no | farmer | -0.394 | 1.047 | -0.105 | -0.112 | 4.5 | rural |
| I-0018-BOS | MDG22203010 | BOSU | human | F | 33 | 0 | no | no | no | other | -0.394 | 1.047 | -0.105 | -0.112 | 4.5 | rural |
| I-0019-BOS | MDG22203010 | BOSU | human | F | 49 | 0 | no | no | no | other | -0.394 | 1.047 | -0.105 | -0.112 | 4.5 | rural |
| I-0020-BOS | MDG22203010 | BOSU | human | F | 51 | 0 | no | no | no | other | -0.394 | 1.047 | -0.105 | -0.112 | 4.5 | rural |
| I-0021-BOS | MDG22203010 | BOSU | human | M | 61 | 0 | no | no | no | contact_env | -0.394 | 1.047 | -0.105 | -0.112 | 4.5 | rural |
| I-0016-BOS | MDG22203010 | BOSU | human | M | 26 | 0 | no | no | no | health | -0.394 | 1.047 | -0.105 | -0.112 | 4.5 | rural |
| I-0023-BOS | MDG22203010 | BOSU | human | F | 27 | 0 | no | no | no | other | -0.394 | 1.047 | -0.105 | -0.112 | 4.5 | rural |
| I-0024-BOS | MDG22203010 | BOSU | human | F | 36 | 1 | no | no | no | other | -0.394 | 1.047 | -0.105 | -0.112 | 4.5 | rural |
| I-0025-BOS | MDG22203010 | BOSU | human | M | 45 | 0 | no | no | no | farmer | -0.394 | 1.047 | -0.105 | -0.112 | 4.5 | rural |
| I-0026-BOS | MDG22203010 | BOSU | human | F | 38 | 0 | no | no | no | other | -0.394 | 1.047 | -0.105 | -0.112 | 4.5 | rural |
| I-0027-BOS | MDG22203010 | BOSU | human | M | 34 | 0 | no | no | no | contact_env | -0.394 | 1.047 | -0.105 | -0.112 | 4.5 | rural |
| I-0029-BOS | MDG22203010 | BOSU | human | F | 18 | 0 | no | no | no | other | -0.394 | 1.047 | -0.105 | -0.112 | 4.5 | rural |
| I-0015-BOS | MDG22203010 | BOSU | human | F | 30 | 0 | no | no | yes | other | -0.394 | 1.047 | -0.105 | -0.112 | 4.5 | rural |
| I-0030-BOS | MDG22203010 | BOSU | human | M | 27 | 0 | yes | no | no | other | -0.394 | 1.047 | -0.105 | -0.112 | 4.5 | rural |
| I-0028-BOS | MDG22203010 | BOSU | human | M | 26 | 0 | no | no | no | other | -0.394 | 1.047 | -0.105 | -0.112 | 4.5 | rural |
| I-0004-BOS | MDG22203010 | BOSU | human | M | 58 | 0 | yes | no | no | farmer | -0.394 | 1.047 | -0.105 | -0.112 | 4.5 | rural |
| I-0017-BOS | MDG22203010 | BOSU | human | M | 56 | 0 | no | no | no | other | -0.394 | 1.047 | -0.105 | -0.112 | 4.5 | rural |
| I-0014-BOS | MDG22203010 | BOSU | human | M | 51 | 0 | no | no | no | farmer | -0.394 | 1.047 | -0.105 | -0.112 | 4.5 | rural |
| I-0001-BOS | MDG22203010 | BOSU | human | M | 32 | 0 | yes | no | no | farmer | -0.394 | 1.047 | -0.105 | -0.112 | 4.5 | rural |
| I-0003-BOS | MDG22203010 | BOSU | human | F | 22 | 0 | no | no | no | farmer | -0.394 | 1.047 | -0.105 | -0.112 | 4.5 | rural |
| I-0005-BOS | MDG22203010 | BOSU | human | M | 55 | 0 | yes | no | no | farmer | -0.394 | 1.047 | -0.105 | -0.112 | 4.5 | rural |
| I-0006-BOS | MDG22203010 | BOSU | human | F | 52 | 0 | yes | no | no | other | -0.394 | 1.047 | -0.105 | -0.112 | 4.5 | rural |
| I-0007-BOS | MDG22203010 | BOSU | human | M | 23 | 0 | yes | no | no | farmer | -0.394 | 1.047 | -0.105 | -0.112 | 4.5 | rural |
| I-0008-BOS | MDG22203010 | BOSU | human | F | 42 | 0 | no | no | no | other | -0.394 | 1.047 | -0.105 | -0.112 | 4.5 | rural |
| I-0009-BOS | MDG22203010 | BOSU | human | F | 25 | 0 | no | no | no | other | -0.394 | 1.047 | -0.105 | -0.112 | 4.5 | rural |
| I-0010-BOS | MDG22203010 | BOSU | human | F | 21 | 0 | no | no | no | other | -0.394 | 1.047 | -0.105 | -0.112 | 4.5 | rural |
| I-0011-BOS | MDG22203010 | BOSU | human | M | 26 | 0 | yes | no | no | other | -0.394 | 1.047 | -0.105 | -0.112 | 4.5 | rural |
| I-0012-BOS | MDG22203010 | BOSU | human | F | 23 | 0 | no | no | no | farmer | -0.394 | 1.047 | -0.105 | -0.112 | 4.5 | rural |
| I-0013-BOS | MDG22203010 | BOSU | human | F | 50 | 0 | no | no | no | other | -0.394 | 1.047 | -0.105 | -0.112 | 4.5 | rural |
| I-0002-BOS | MDG22203010 | BOSU | human | F | 70 | 0 | no | no | no | farmer | -0.394 | 1.047 | -0.105 | -0.112 | 4.5 | rural |
| I-0038-BOS | MDG22203190 | BOSR | human | F | 25 | 0 | yes | no | no | other | -0.449 | 1.077 | 0.007 | -0.141 | 5.66666666666666 | rural |
| I-0052-BOS | MDG22203190 | BOSR | human | F | 34 | 0 | no | no | no | farmer | -0.449 | 1.077 | 0.007 | -0.141 | 5.66666666666666 | rural |
| I-0048-BOS | MDG22203190 | BOSR | human | F | 54 | 0 | yes | no | no | farmer | -0.449 | 1.077 | 0.007 | -0.141 | 5.66666666666666 | rural |
| I-0049-BOS | MDG22203190 | BOSR | human | F | 26 | 0 | yes | no | no | farmer | -0.449 | 1.077 | 0.007 | -0.141 | 5.66666666666666 | rural |
| I-0050-BOS | MDG22203190 | BOSR | human | F | 24 | 0 | no | no | no | farmer | -0.449 | 1.077 | 0.007 | -0.141 | 5.66666666666666 | rural |
| I-0051-BOS | MDG22203190 | BOSR | human | M | 41 | 0 | no | no | no | farmer | -0.449 | 1.077 | 0.007 | -0.141 | 5.66666666666666 | rural |
| I-0060-BOS | MDG22203190 | BOSR | human | F | 84 | 0 | no | no | no | other | -0.449 | 1.077 | 0.007 | -0.141 | 5.66666666666666 | rural |
| I-0047-BOS | MDG22203190 | BOSR | human | F | 47 | 0 | yes | no | no | farmer | -0.449 | 1.077 | 0.007 | -0.141 | 5.66666666666666 | rural |
| I-0053-BOS | MDG22203190 | BOSR | human | M | 39 | 0 | yes | no | no | farmer | -0.449 | 1.077 | 0.007 | -0.141 | 5.66666666666666 | rural |
| I-0054-BOS | MDG22203190 | BOSR | human | F | 41 | 0 | yes | no | no | farmer | -0.449 | 1.077 | 0.007 | -0.141 | 5.66666666666666 | rural |
| I-0055-BOS | MDG22203190 | BOSR | human | F | 43 | 0 | no | no | no | farmer | -0.449 | 1.077 | 0.007 | -0.141 | 5.66666666666666 | rural |
| I-0056-BOS | MDG22203190 | BOSR | human | F | 55 | 0 | no | no | no | other | -0.449 | 1.077 | 0.007 | -0.141 | 5.66666666666666 | rural |
| I-0057-BOS | MDG22203190 | BOSR | human | F | 37 | 0 | yes | no | no | other | -0.449 | 1.077 | 0.007 | -0.141 | 5.66666666666666 | rural |
| I-0059-BOS | MDG22203190 | BOSR | human | M | 42 | 0 | no | no | no | farmer | -0.449 | 1.077 | 0.007 | -0.141 | 5.66666666666666 | rural |
| I-0058-BOS | MDG22203190 | BOSR | human | F | 43 | 0 | no | no | no | farmer | -0.449 | 1.077 | 0.007 | -0.141 | 5.66666666666666 | rural |

| ID | PCODE | site2 | sp | gender | age | IgG | contact_ruminant | contact_milk | contact_fresh_fluid | profession | fact1 | fact2 | fact3 | fact4 | cattle_density | habitat |
| --- | --- | --- | --- | --- | --- | --- | --- | --- | --- | --- | --- | --- | --- | --- | --- | --- |
| I-0035-BOS | MDG22203190 | BOSR | human | F | 19 | 0 | no | no | no | farmer | -0.449 | 1.077 | 0.007 | -0.141 | 5.66666666666666 | rural |
| I-0046-BOS | MDG22203190 | BOSR | human | M | 34 | 0 | yes | no | no | farmer | -0.449 | 1.077 | 0.007 | -0.141 | 5.66666666666666 | rural |
| I-0031-BOS | MDG22203190 | BOSR | human | F | 59 | 0 | no | no | no | farmer | -0.449 | 1.077 | 0.007 | -0.141 | 5.66666666666666 | rural |
| I-0032-BOS | MDG22203190 | BOSR | human | F | 23 | 0 | no | no | no | farmer | -0.449 | 1.077 | 0.007 | -0.141 | 5.66666666666666 | rural |
| I-0034-BOS | MDG22203190 | BOSR | human | F | 44 | 0 | no | no | no | farmer | -0.449 | 1.077 | 0.007 | -0.141 | 5.66666666666666 | rural |
| I-0040-BOS | MDG22203190 | BOSR | human | M | 28 | 0 | yes | no | no | farmer | -0.449 | 1.077 | 0.007 | -0.141 | 5.66666666666666 | rural |
| I-0036-BOS | MDG22203190 | BOSR | human | F | 46 | 0 | no | no | no | farmer | -0.449 | 1.077 | 0.007 | -0.141 | 5.66666666666666 | rural |
| I-0037-BOS | MDG22203190 | BOSR | human | M | 22 | 0 | yes | no | no | other | -0.449 | 1.077 | 0.007 | -0.141 | 5.66666666666666 | rural |
| I-0039-BOS | MDG22203190 | BOSR | human | F | 21 | 0 | yes | no | no | farmer | -0.449 | 1.077 | 0.007 | -0.141 | 5.66666666666666 | rural |
| I-0041-BOS | MDG22203190 | BOSR | human | M | 48 | 0 | no | no | no | farmer | -0.449 | 1.077 | 0.007 | -0.141 | 5.66666666666666 | rural |
| I-0042-BOS | MDG22203190 | BOSR | human | M | 40 | 0 | yes | no | no | farmer | -0.449 | 1.077 | 0.007 | -0.141 | 5.66666666666666 | rural |
| I-0043-BOS | MDG22203190 | BOSR | human | M | 41 | 0 | no | no | no | farmer | -0.449 | 1.077 | 0.007 | -0.141 | 5.66666666666666 | rural |
| I-0044-BOS | MDG22203190 | BOSR | human | F | 58 | 0 | no | no | no | farmer | -0.449 | 1.077 | 0.007 | -0.141 | 5.66666666666666 | rural |
| I-0045-BOS | MDG22203190 | BOSR | human | F | 41 | 0 | no | no | no | farmer | -0.449 | 1.077 | 0.007 | -0.141 | 5.66666666666666 | rural |
| I-0033-BOS | MDG22203190 | BOSR | human | F | 55 | 0 | yes | no | no | farmer | -0.449 | 1.077 | 0.007 | -0.141 | 5.66666666666666 | rural |
| I-0023-MNJ | MDG23209010 | MNJU | human | F | 42 | 0 | no | no | no | other | -1.008 | -0.681 | -0.666 | 0.407 | 2.33333333333333 | rural |
| I-0018-MNJ | MDG23209010 | MNJU | human | F | 30 | 0 | no | no | no | other | -1.008 | -0.681 | -0.666 | 0.407 | 2.33333333333333 | rural |
| I-0019-MNJ | MDG23209010 | MNJU | human | F | 24 | 0 | no | no | no | other | -1.008 | -0.681 | -0.666 | 0.407 | 2.33333333333333 | rural |
| I-0020-MNJ | MDG23209010 | MNJU | human | M | 23 | 0 | no | no | no | other | -1.008 | -0.681 | -0.666 | 0.407 | 2.33333333333333 | rural |
| I-0021-MNJ | MDG23209010 | MNJU | human | M | 53 | 0 | no | no | no | other | -1.008 | -0.681 | -0.666 | 0.407 | 2.33333333333333 | rural |
| I-0022-MNJ | MDG23209010 | MNJU | human | F | 22 | 0 | no | no | no | other | -1.008 | -0.681 | -0.666 | 0.407 | 2.33333333333333 | rural |
| I-0024-MNJ | MDG23209010 | MNJU | human | F | 40 | 0 | no | no | no | other | -1.008 | -0.681 | -0.666 | 0.407 | 2.33333333333333 | rural |
| I-0025-MNJ | MDG23209010 | MNJU | human | F | 19 | 0 | no | no | no | other | -1.008 | -0.681 | -0.666 | 0.407 | 2.33333333333333 | rural |
| I-0026-MNJ | MDG23209010 | MNJU | human | M | 75 | 0 | no | no | no | other | -1.008 | -0.681 | -0.666 | 0.407 | 2.33333333333333 | rural |
| I-0027-MNJ | MDG23209010 | MNJU | human | F | 53 | 0 | no | no | no | other | -1.008 | -0.681 | -0.666 | 0.407 | 2.33333333333333 | rural |
| I-0028-MNJ | MDG23209010 | MNJU | human | F | 36 | 0 | no | no | no | other | -1.008 | -0.681 | -0.666 | 0.407 | 2.33333333333333 | rural |
| I-0016-MNJ | MDG23209010 | MNJU | human | F | 20 | 0 | no | no | no | other | -1.008 | -0.681 | -0.666 | 0.407 | 2.33333333333333 | rural |
| I-0030-MNJ | MDG23209010 | MNJU | human | F | 36 | 0 | no | no | no | other | -1.008 | -0.681 | -0.666 | 0.407 | 2.33333333333333 | rural |
| I-0015-MNJ | MDG23209010 | MNJU | human | F | 48 | 0 | no | no | no | other | -1.008 | -0.681 | -0.666 | 0.407 | 2.33333333333333 | rural |
| I-0029-MNJ | MDG23209010 | MNJU | human | F | 44 | 0 | no | no | no | other | -1.008 | -0.681 | -0.666 | 0.407 | 2.33333333333333 | rural |
| I-0005-MNJ | MDG23209010 | MNJU | human | M | 43 | 0 | no | no | no | other | -1.008 | -0.681 | -0.666 | 0.407 | 2.33333333333333 | rural |
| I-0017-MNJ | MDG23209010 | MNJU | human | M | 27 | 0 | no | no | no | other | -1.008 | -0.681 | -0.666 | 0.407 | 2.33333333333333 | rural |
| I-0002-MNJ | MDG23209010 | MNJU | human | F | 73 | 0 | no | no | no | other | -1.008 | -0.681 | -0.666 | 0.407 | 2.33333333333333 | rural |
| I-0004-MNJ | MDG23209010 | MNJU | human | F | 45 | 0 | no | no | no | other | -1.008 | -0.681 | -0.666 | 0.407 | 2.33333333333333 | rural |
| I-0001-MNJ | MDG23209010 | MNJU | human | M | 54 | 0 | no | no | no | other | -1.008 | -0.681 | -0.666 | 0.407 | 2.33333333333333 | rural |
| I-0006-MNJ | MDG23209010 | MNJU | human | M | 18 | 0 | no | no | no | other | -1.008 | -0.681 | -0.666 | 0.407 | 2.33333333333333 | rural |
| I-0007-MNJ | MDG23209010 | MNJU | human | F | 40 | 0 | no | no | no | other | -1.008 | -0.681 | -0.666 | 0.407 | 2.33333333333333 | rural |
| I-0008-MNJ | MDG23209010 | MNJU | human | M | 38 | 0 | no | no | no | other | -1.008 | -0.681 | -0.666 | 0.407 | 2.33333333333333 | rural |
| I-0009-MNJ | MDG23209010 | MNJU | human | F | 26 | 0 | no | no | no | other | -1.008 | -0.681 | -0.666 | 0.407 | 2.33333333333333 | rural |
| I-0010-MNJ | MDG23209010 | MNJU | human | F | 49 | 0 | no | no | no | other | -1.008 | -0.681 | -0.666 | 0.407 | 2.33333333333333 | rural |
| I-0011-MNJ | MDG23209010 | MNJU | human | M | 45 | 1 | no | no | no | other | -1.008 | -0.681 | -0.666 | 0.407 | 2.33333333333333 | rural |
| I-0012-MNJ | MDG23209010 | MNJU | human | M | 46 | 0 | no | no | no | other | -1.008 | -0.681 | -0.666 | 0.407 | 2.33333333333333 | rural |
| I-0013-MNJ | MDG23209010 | MNJU | human | M | 20 | 0 | no | no | no | other | -1.008 | -0.681 | -0.666 | 0.407 | 2.33333333333333 | rural |
| I-0014-MNJ | MDG23209010 | MNJU | human | M | 48 | 0 | no | no | no | other | -1.008 | -0.681 | -0.666 | 0.407 | 2.33333333333333 | rural |
| I-0003-MNJ | MDG23209010 | MNJU | human | M | 47 | 0 | no | no | no | other | -1.008 | -0.681 | -0.666 | 0.407 | 2.33333333333333 | rural |
| I-0054-MNJ | MDG23209050 | MNJR | human | M | 23 | 0 | yes | no | no | farmer | -0.936 | -0.071 | -0.227 | 0.171 | 2.28571428571428 | rural |
| I-0048-MNJ | MDG23209050 | MNJR | human | M | 62 | 0 | no | no | no | farmer | -0.936 | -0.071 | -0.227 | 0.171 | 2.28571428571428 | rural |
| I-0050-MNJ | MDG23209050 | MNJR | human | M | 48 | 1 | no | no | no | contact_env | -0.936 | -0.071 | -0.227 | 0.171 | 2.28571428571428 | rural |
| I-0051-MNJ | MDG23209050 | MNJR | human | F | 28 | 0 | no | no | no | other | -0.936 | -0.071 | -0.227 | 0.171 | 2.28571428571428 | rural |
| I-0052-MNJ | MDG23209050 | MNJR | human | M | 28 | 0 | yes | no | no | farmer | -0.936 | -0.071 | -0.227 | 0.171 | 2.28571428571428 | rural |
| I-0053-MNJ | MDG23209050 | MNJR | human | F | 36 | 0 | no | no | no | farmer | -0.936 | -0.071 | -0.227 | 0.171 | 2.28571428571428 | rural |
| I-0055-MNJ | MDG23209050 | MNJR | human | M | 29 | 0 | yes | no | no | farmer | -0.936 | -0.071 | -0.227 | 0.171 | 2.28571428571428 | rural |
| I-0056-MNJ | MDG23209050 | MNJR | human | F | 21 | 0 | no | no | no | farmer | -0.936 | -0.071 | -0.227 | 0.171 | 2.28571428571428 | rural |
| I-0057-MNJ | MDG23209050 | MNJR | human | M | 55 | 1 | yes | no | no | farmer | -0.936 | -0.071 | -0.227 | 0.171 | 2.28571428571428 | rural |
| I-0058-MNJ | MDG23209050 | MNJR | human | F | 71 | 0 | no | no | no | farmer | -0.936 | -0.071 | -0.227 | 0.171 | 2.28571428571428 | rural |
| I-0059-MNJ | MDG23209050 | MNJR | human | F | 26 | 0 | no | no | no | other | -0.936 | -0.071 | -0.227 | 0.171 | 2.28571428571428 | rural |
| I-0060-MNJ | MDG23209050 | MNJR | human | M | 80 | 1 | no | no | no | farmer | -0.936 | -0.071 | -0.227 | 0.171 | 2.28571428571428 | rural |
| I-0047-MNJ | MDG23209050 | MNJR | human | F | 35 | 0 | no | no | no | farmer | -0.936 | -0.071 | -0.227 | 0.171 | 2.28571428571428 | rural |
| I-0034-MNJ | MDG23209050 | MNJR | human | F | 27 | 0 | no | no | no | other | -0.936 | -0.071 | -0.227 | 0.171 | 2.28571428571428 | rural |
| I-0046-MNJ | MDG23209050 | MNJR | human | M | 53 | 0 | no | no | no | farmer | -0.936 | -0.071 | -0.227 | 0.171 | 2.28571428571428 | rural |
| I-0049-MNJ | MDG23209050 | MNJR | human | M | 41 | 0 | no | no | no | farmer | -0.936 | -0.071 | -0.227 | 0.171 | 2.28571428571428 | rural |
| I-0031-MNJ | MDG23209050 | MNJR | human | F | 40 | 0 | no | no | no | farmer | -0.936 | -0.071 | -0.227 | 0.171 | 2.28571428571428 | rural |
| I-0033-MNJ | MDG23209050 | MNJR | human | M | 28 | 0 | no | no | no | contact_env | -0.936 | -0.071 | -0.227 | 0.171 | 2.28571428571428 | rural |

| ID | PCODE | site2 | sp | gender | age | IgG | contact_ruminant | contact_milk | contact_fresh_fluid | profession | fact1 | fact2 | fact3 | fact4 | cattle_density | habitat |
| --- | --- | --- | --- | --- | --- | --- | --- | --- | --- | --- | --- | --- | --- | --- | --- | --- |
| I-0035-MNJ | MDG23209050 | MNJR | human | F | 33 | 0 | no | no | no | other | -0.936 | -0.071 | -0.227 | 0.171 | 2.28571428571428 | rural |
| I-0036-MNJ | MDG23209050 | MNJR | human | M | 58 | 0 | yes | no | no | farmer | -0.936 | -0.071 | -0.227 | 0.171 | 2.28571428571428 | rural |
| I-0037-MNJ | MDG23209050 | MNJR | human | F | 57 | 0 | no | no | no | farmer | -0.936 | -0.071 | -0.227 | 0.171 | 2.28571428571428 | rural |
| I-0039-MNJ | MDG23209050 | MNJR | human | F | 18 | 0 | no | no | no | other | -0.936 | -0.071 | -0.227 | 0.171 | 2.28571428571428 | rural |
| I-0040-MNJ | MDG23209050 | MNJR | human | F | 51 | 0 | no | no | no | farmer | -0.936 | -0.071 | -0.227 | 0.171 | 2.28571428571428 | rural |
| I-0041-MNJ | MDG23209050 | MNJR | human | M | 82 | 0 | no | no | no | farmer | -0.936 | -0.071 | -0.227 | 0.171 | 2.28571428571428 | rural |
| I-0042-MNJ | MDG23209050 | MNJR | human | F | 53 | 0 | no | no | no | other | -0.936 | -0.071 | -0.227 | 0.171 | 2.28571428571428 | rural |
| I-0045-MNJ | MDG23209050 | MNJR | human | M | 57 | 0 | yes | no | no | farmer | -0.936 | -0.071 | -0.227 | 0.171 | 2.28571428571428 | rural |
| I-0043-MNJ | MDG23209050 | MNJR | human | M | 46 | 0 | yes | no | no | farmer | -0.936 | -0.071 | -0.227 | 0.171 | 2.28571428571428 | rural |
| I-0038-MNJ | MDG23209050 | MNJR | human | F | 43 | 0 | no | no | no | farmer | -0.936 | -0.071 | -0.227 | 0.171 | 2.28571428571428 | rural |
| I-0032-MNJ | MDG23209050 | MNJR | human | M | 19 | 0 | yes | no | no | contact_env | -0.936 | -0.071 | -0.227 | 0.171 | 2.28571428571428 | rural |
| I-0044-MNJ | MDG23209050 | MNJR | human | M | 21 | 1 | yes | no | no | farmer | -0.936 | -0.071 | -0.227 | 0.171 | 2.28571428571428 | rural |
| I-0021-FAR | MDG25213010 | FARU | human | F | 24 | 0 | no | no | no | other | -0.063 | 0.369 | 0.971 | 4.437 | 2.66666666666666 | rural |
| I-0026-FAR | MDG25213010 | FARU | human | F | 31 | 0 | no | no | no | other | -0.063 | 0.369 | 0.971 | 4.437 | 2.66666666666666 | rural |
| I-0020-FAR | MDG25213010 | FARU | human | M | 37 | 1 | no | no | no | other | -0.063 | 0.369 | 0.971 | 4.437 | 2.66666666666666 | rural |
| I-0022-FAR | MDG25213010 | FARU | human | F | 34 | 0 | no | no | no | other | -0.063 | 0.369 | 0.971 | 4.437 | 2.66666666666666 | rural |
| I-0023-FAR | MDG25213010 | FARU | human | F | 39 | 0 | no | no | no | other | -0.063 | 0.369 | 0.971 | 4.437 | 2.66666666666666 | rural |
| I-0024-FAR | MDG25213010 | FARU | human | F | 56 | 0 | no | no | no | other | -0.063 | 0.369 | 0.971 | 4.437 | 2.66666666666666 | rural |
| I-0025-FAR | MDG25213010 | FARU | human | M | 30 | 0 | no | no | no | other | -0.063 | 0.369 | 0.971 | 4.437 | 2.66666666666666 | rural |
| I-0027-FAR | MDG25213010 | FARU | human | M | 24 | 0 | no | no | no | other | -0.063 | 0.369 | 0.971 | 4.437 | 2.66666666666666 | rural |
| I-0028-FAR | MDG25213010 | FARU | human | M | 21 | 0 | no | no | no | other | -0.063 | 0.369 | 0.971 | 4.437 | 2.66666666666666 | rural |
| I-0029-FAR | MDG25213010 | FARU | human | F | 24 | 0 | no | no | no | other | -0.063 | 0.369 | 0.971 | 4.437 | 2.66666666666666 | rural |
| I-0030-FAR | MDG25213010 | FARU | human | M | 20 | 0 | no | no | no | other | -0.063 | 0.369 | 0.971 | 4.437 | 2.66666666666666 | rural |
| I-0016-FAR | MDG25213010 | FARU | human | M | 36 | 0 | no | no | no | other | -0.063 | 0.369 | 0.971 | 4.437 | 2.66666666666666 | rural |
| I-0019-FAR | MDG25213010 | FARU | human | F | 20 | 0 | no | no | no | other | -0.063 | 0.369 | 0.971 | 4.437 | 2.66666666666666 | rural |
| I-0004-FAR | MDG25213010 | FARU | human | F | 36 | 0 | no | no | no | other | -0.063 | 0.369 | 0.971 | 4.437 | 2.66666666666666 | rural |
| I-0018-FAR | MDG25213010 | FARU | human | F | 47 | 0 | no | no | no | other | -0.063 | 0.369 | 0.971 | 4.437 | 2.66666666666666 | rural |
| I-0017-FAR | MDG25213010 | FARU | human | F | 42 | 0 | no | no | no | other | -0.063 | 0.369 | 0.971 | 4.437 | 2.66666666666666 | rural |
| I-0003-FAR | MDG25213010 | FARU | human | F | 40 | 0 | no | no | no | other | -0.063 | 0.369 | 0.971 | 4.437 | 2.66666666666666 | rural |
| I-0005-FAR | MDG25213010 | FARU | human | F | 24 | 0 | no | no | no | other | -0.063 | 0.369 | 0.971 | 4.437 | 2.66666666666666 | rural |
| I-0006-FAR | MDG25213010 | FARU | human | M | 80 | 0 | no | no | no | health | -0.063 | 0.369 | 0.971 | 4.437 | 2.66666666666666 | rural |
| I-0007-FAR | MDG25213010 | FARU | human | F | 19 | 0 | no | no | no | other | -0.063 | 0.369 | 0.971 | 4.437 | 2.66666666666666 | rural |
| I-0008-FAR | MDG25213010 | FARU | human | F | 21 | 0 | yes | no | no | other | -0.063 | 0.369 | 0.971 | 4.437 | 2.66666666666666 | rural |
| I-0009-FAR | MDG25213010 | FARU | human | M | 61 | 1 | no | no | no | other | -0.063 | 0.369 | 0.971 | 4.437 | 2.66666666666666 | rural |
| I-0011-FAR | MDG25213010 | FARU | human | F | 46 | 0 | no | no | no | other | -0.063 | 0.369 | 0.971 | 4.437 | 2.66666666666666 | rural |
| I-0012-FAR | MDG25213010 | FARU | human | F | 27 | 0 | no | no | no | other | -0.063 | 0.369 | 0.971 | 4.437 | 2.66666666666666 | rural |
| I-0013-FAR | MDG25213010 | FARU | human | F | 38 | 1 | no | no | no | other | -0.063 | 0.369 | 0.971 | 4.437 | 2.66666666666666 | rural |
| I-0014-FAR | MDG25213010 | FARU | human | M | 22 | 0 | no | no | no | other | -0.063 | 0.369 | 0.971 | 4.437 | 2.66666666666666 | rural |
| I-0001-FAR | MDG25213010 | FARU | human | M | 23 | 0 | no | no | no | other | -0.063 | 0.369 | 0.971 | 4.437 | 2.66666666666666 | rural |
| I-0015-FAR | MDG25213010 | FARU | human | F | 21 | 0 | no | no | no | other | -0.063 | 0.369 | 0.971 | 4.437 | 2.66666666666666 | rural |
| I-0010-FAR | MDG25213010 | FARU | human | F | 36 | 0 | no | no | no | other | -0.063 | 0.369 | 0.971 | 4.437 | 2.66666666666666 | rural |
| I-0002-FAR | MDG25213010 | FARU | human | M | 19 | 0 | no | no | no | other | -0.063 | 0.369 | 0.971 | 4.437 | 2.66666666666666 | rural |
| I-0056-FAR | MDG25213130 | FARR | human | M | 22 | 0 | no | no | no | farmer | -0.568 | 0.384 | 0.469 | 0.193 | 2.75 | rural |
| I-0047-FAR | MDG25213130 | FARR | human | M | 28 | 0 | no | no | no | farmer | -0.568 | 0.384 | 0.469 | 0.193 | 2.75 | rural |
| I-0048-FAR | MDG25213130 | FARR | human | M | 47 | 0 | no | no | no | farmer | -0.568 | 0.384 | 0.469 | 0.193 | 2.75 | rural |
| I-0049-FAR | MDG25213130 | FARR | human | M | 52 | 0 | no | no | no | farmer | -0.568 | 0.384 | 0.469 | 0.193 | 2.75 | rural |
| I-0050-FAR | MDG25213130 | FARR | human | M | 55 | 1 | no | no | no | farmer | -0.568 | 0.384 | 0.469 | 0.193 | 2.75 | rural |
| I-0051-FAR | MDG25213130 | FARR | human | M | 42 | 0 | no | no | no | farmer | -0.568 | 0.384 | 0.469 | 0.193 | 2.75 | rural |
| I-0052-FAR | MDG25213130 | FARR | human | M | 38 | 0 | no | no | no | farmer | -0.568 | 0.384 | 0.469 | 0.193 | 2.75 | rural |
| I-0053-FAR | MDG25213130 | FARR | human | M | 19 | 0 | no | no | no | farmer | -0.568 | 0.384 | 0.469 | 0.193 | 2.75 | rural |
| I-0055-FAR | MDG25213130 | FARR | human | M | 37 | 0 | no | no | no | farmer | -0.568 | 0.384 | 0.469 | 0.193 | 2.75 | rural |
| I-0057-FAR | MDG25213130 | FARR | human | M | 33 | 0 | no | no | no | farmer | -0.568 | 0.384 | 0.469 | 0.193 | 2.75 | rural |
| I-0058-FAR | MDG25213130 | FARR | human | M | 59 | 0 | no | no | no | farmer | -0.568 | 0.384 | 0.469 | 0.193 | 2.75 | rural |
| I-0059-FAR | MDG25213130 | FARR | human | M | 41 | 0 | no | no | no | farmer | -0.568 | 0.384 | 0.469 | 0.193 | 2.75 | rural |
| I-0044-FAR | MDG25213130 | FARR | human | M | 62 | 1 | no | no | no | farmer | -0.568 | 0.384 | 0.469 | 0.193 | 2.75 | rural |
| I-0054-FAR | MDG25213130 | FARR | human | M | 21 | 0 | no | no | no | farmer | -0.568 | 0.384 | 0.469 | 0.193 | 2.75 | rural |
| I-0034-FAR | MDG25213130 | FARR | human | M | 65 | 1 | no | no | no | farmer | -0.568 | 0.384 | 0.469 | 0.193 | 2.75 | rural |
| I-0046-FAR | MDG25213130 | FARR | human | M | 36 | 0 | no | no | no | farmer | -0.568 | 0.384 | 0.469 | 0.193 | 2.75 | rural |
| I-0045-FAR | MDG25213130 | FARR | human | M | 50 | 1 | no | no | no | farmer | -0.568 | 0.384 | 0.469 | 0.193 | 2.75 | rural |
| I-0031-FAR | MDG25213130 | FARR | human | M | 25 | 0 | no | no | no | farmer | -0.568 | 0.384 | 0.469 | 0.193 | 2.75 | rural |
| I-0033-FAR | MDG25213130 | FARR | human | F | 56 | 0 | no | no | no | farmer | -0.568 | 0.384 | 0.469 | 0.193 | 2.75 | rural |
| I-0060-FAR | MDG25213130 | FARR | human | F | 42 | 1 | no | no | no | other | -0.568 | 0.384 | 0.469 | 0.193 | 2.75 | rural |
| I-0035-FAR | MDG25213130 | FARR | human | F | 25 | 0 | no | no | no | farmer | -0.568 | 0.384 | 0.469 | 0.193 | 2.75 | rural |

| ID | PCODE | site2 | sp | gender | age | IgG | contact_ruminant | contact_milk | contact_fresh_fluid | profession | fact1 | fact2 | fact3 | fact4 | cattle_density | habitat |
| --- | --- | --- | --- | --- | --- | --- | --- | --- | --- | --- | --- | --- | --- | --- | --- | --- |
| I-0036-FAR | MDG25213130 | FARR | human | M | 19 | 0 | no | no | no | farmer | -0.568 | 0.384 | 0.469 | 0.193 | 2.75 | rural |
| I-0037-FAR | MDG25213130 | FARR | human | M | 65 | 1 | no | no | no | farmer | -0.568 | 0.384 | 0.469 | 0.193 | 2.75 | rural |
| I-0038-FAR | MDG25213130 | FARR | human | F | 31 | 0 | no | no | no | other | -0.568 | 0.384 | 0.469 | 0.193 | 2.75 | rural |
| I-0039-FAR | MDG25213130 | FARR | human | F | 52 | 1 | no | no | no | other | -0.568 | 0.384 | 0.469 | 0.193 | 2.75 | rural |
| I-0040-FAR | MDG25213130 | FARR | human | F | 26 | 0 | no | no | no | farmer | -0.568 | 0.384 | 0.469 | 0.193 | 2.75 | rural |
| I-0041-FAR | MDG25213130 | FARR | human | M | 29 | 0 | no | no | no | farmer | -0.568 | 0.384 | 0.469 | 0.193 | 2.75 | rural |
| I-0042-FAR | MDG25213130 | FARR | human | M | 67 | 0 | no | no | no | farmer | -0.568 | 0.384 | 0.469 | 0.193 | 2.75 | rural |
| I-0043-FAR | MDG25213130 | FARR | human | M | 32 | 0 | no | no | no | farmer | -0.568 | 0.384 | 0.469 | 0.193 | 2.75 | rural |
| I-0032-FAR | MDG25213130 | FARR | human | F | 44 | 0 | no | no | no | farmer | -0.568 | 0.384 | 0.469 | 0.193 | 2.75 | rural |
| I-0060-IHO | MDG24216031 | IHOR | human | M | 29 | 0 | no | no | no | farmer | 1.431 | 0.094 | -0.913 | 0.045 | 11.1428571428571 | rural |
| I-0051-IHO | MDG24216031 | IHOR | human | M | 26 | 0 | yes | no | no | farmer | 1.431 | 0.094 | -0.913 | 0.045 | 11.1428571428571 | rural |
| I-0059-IHO | MDG24216031 | IHOR | human | M | 32 | 0 | yes | no | no | farmer | 1.431 | 0.094 | -0.913 | 0.045 | 11.1428571428571 | rural |
| I-0058-IHO | MDG24216031 | IHOR | human | F | 29 | 0 | no | no | no | other | 1.431 | 0.094 | -0.913 | 0.045 | 11.1428571428571 | rural |
| I-0057-IHO | MDG24216031 | IHOR | human | F | 42 | 0 | no | no | no | farmer | 1.431 | 0.094 | -0.913 | 0.045 | 11.1428571428571 | rural |
| I-0056-IHO | MDG24216031 | IHOR | human | F | 26 | 0 | no | no | no | other | 1.431 | 0.094 | -0.913 | 0.045 | 11.1428571428571 | rural |
| I-0055-IHO | MDG24216031 | IHOR | human | F | 44 | 0 | no | no | no | other | 1.431 | 0.094 | -0.913 | 0.045 | 11.1428571428571 | rural |
| I-0054-IHO | MDG24216031 | IHOR | human | F | 20 | 0 | yes | no | no | other | 1.431 | 0.094 | -0.913 | 0.045 | 11.1428571428571 | rural |
| I-0052-IHO | MDG24216031 | IHOR | human | F | 23 | 0 | no | no | no | other | 1.431 | 0.094 | -0.913 | 0.045 | 11.1428571428571 | rural |
| I-0012-IHO | MDG24216031 | IHOU | human | F | 62 | 0 | no | no | no | other | 1.431 | 0.094 | -0.913 | 0.045 | 11.1428571428571 | rural |
| I-0050-IHO | MDG24216031 | IHOR | human | F | 65 | 0 | yes | no | no | other | 1.431 | 0.094 | -0.913 | 0.045 | 11.1428571428571 | rural |
| I-0049-IHO | MDG24216031 | IHOR | human | F | 38 | 0 | no | no | no | farmer | 1.431 | 0.094 | -0.913 | 0.045 | 11.1428571428571 | rural |
| I-0048-IHO | MDG24216031 | IHOR | human | M | 40 | 0 | yes | no | no | farmer | 1.431 | 0.094 | -0.913 | 0.045 | 11.1428571428571 | rural |
| I-0053-IHO | MDG24216031 | IHOR | human | F | 46 | 0 | no | no | no | farmer | 1.431 | 0.094 | -0.913 | 0.045 | 11.1428571428571 | rural |
| I-0047-IHO | MDG24216031 | IHOR | human | F | 45 | 0 | no | no | no | other | 1.431 | 0.094 | -0.913 | 0.045 | 11.1428571428571 | rural |
| I-0009-IHO | MDG24216031 | IHOU | human | F | 19 | 0 | no | no | no | other | 1.431 | 0.094 | -0.913 | 0.045 | 11.1428571428571 | rural |
| I-0022-IHO | MDG24216031 | IHOU | human | M | 65 | 0 | no | no | no | farmer | 1.431 | 0.094 | -0.913 | 0.045 | 11.1428571428571 | rural |
| I-0021-IHO | MDG24216031 | IHOU | human | F | 21 | 0 | no | no | no | other | 1.431 | 0.094 | -0.913 | 0.045 | 11.1428571428571 | rural |
| I-0020-IHO | MDG24216031 | IHOU | human | M | 24 | 0 | no | no | no | farmer | 1.431 | 0.094 | -0.913 | 0.045 | 11.1428571428571 | rural |
| I-0019-IHO | MDG24216031 | IHOU | human | M | 24 | 0 | no | no | no | other | 1.431 | 0.094 | -0.913 | 0.045 | 11.1428571428571 | rural |
| I-0018-IHO | MDG24216031 | IHOU | human | F | 18 | 0 | no | no | no | farmer | 1.431 | 0.094 | -0.913 | 0.045 | 11.1428571428571 | rural |
| I-0016-IHO | MDG24216031 | IHOU | human | M | 36 | 0 | yes | no | no | farmer | 1.431 | 0.094 | -0.913 | 0.045 | 11.1428571428571 | rural |
| I-0013-IHO | MDG24216031 | IHOU | human | F | 26 | 0 | no | no | no | farmer | 1.431 | 0.094 | -0.913 | 0.045 | 11.1428571428571 | rural |
| I-0014-IHO | MDG24216031 | IHOU | human | M | 20 | 0 | yes | no | no | farmer | 1.431 | 0.094 | -0.913 | 0.045 | 11.1428571428571 | rural |
| I-0023-IHO | MDG24216031 | IHOU | human | M | 19 | 0 | yes | no | no | other | 1.431 | 0.094 | -0.913 | 0.045 | 11.1428571428571 | rural |
| I-0010-IHO | MDG24216031 | IHOU | human | F | 32 | 0 | no | no | no | other | 1.431 | 0.094 | -0.913 | 0.045 | 11.1428571428571 | rural |
| I-0017-IHO | MDG24216031 | IHOU | human | M | 50 | 0 | yes | no | no | other | 1.431 | 0.094 | -0.913 | 0.045 | 11.1428571428571 | rural |
| I-0008-IHO | MDG24216031 | IHOU | human | M | 50 | 0 | yes | no | no | other | 1.431 | 0.094 | -0.913 | 0.045 | 11.1428571428571 | rural |
| I-0007-IHO | MDG24216031 | IHOU | human | F | 46 | 0 | no | no | no | other | 1.431 | 0.094 | -0.913 | 0.045 | 11.1428571428571 | rural |
| I-0006-IHO | MDG24216031 | IHOU | human | F | 19 | 0 | no | no | no | contact_env | 1.431 | 0.094 | -0.913 | 0.045 | 11.1428571428571 | rural |
| I-0005-IHO | MDG24216031 | IHOU | human | F | 44 | 0 | no | no | no | farmer | 1.431 | 0.094 | -0.913 | 0.045 | 11.1428571428571 | rural |
| I-0004-IHO | MDG24216031 | IHOU | human | M | 67 | 0 | no | no | no | other | 1.431 | 0.094 | -0.913 | 0.045 | 11.1428571428571 | rural |
| I-0003-IHO | MDG24216031 | IHOU | human | M | 29 | 0 | yes | yes | no | farmer | 1.431 | 0.094 | -0.913 | 0.045 | 11.1428571428571 | rural |
| I-0002-IHO | MDG24216031 | IHOU | human | F | 28 | 0 | yes | no | no | farmer | 1.431 | 0.094 | -0.913 | 0.045 | 11.1428571428571 | rural |
| I-0001-IHO | MDG24216031 | IHOU | human | F | 56 | 0 | yes | no | no | farmer | 1.431 | 0.094 | -0.913 | 0.045 | 11.1428571428571 | rural |
| I-0046-IHO | MDG24216031 | IHOR | human | M | 57 | 1 | yes | no | no | farmer | 1.431 | 0.094 | -0.913 | 0.045 | 11.1428571428571 | rural |
| I-0011-IHO | MDG24216031 | IHOU | human | F | 20 | 0 | no | no | no | other | 1.431 | 0.094 | -0.913 | 0.045 | 11.1428571428571 | rural |
| I-0040-IHO | MDG24216031 | IHOR | human | M | 28 | 0 | yes | yes | no | farmer | 1.431 | 0.094 | -0.913 | 0.045 | 11.1428571428571 | rural |
| I-0045-IHO | MDG24216031 | IHOR | human | M | 48 | 0 | yes | no | no | farmer | 1.431 | 0.094 | -0.913 | 0.045 | 11.1428571428571 | rural |
| I-0044-IHO | MDG24216031 | IHOR | human | M | 35 | 0 | yes | yes | no | farmer | 1.431 | 0.094 | -0.913 | 0.045 | 11.1428571428571 | rural |
| I-0043-IHO | MDG24216031 | IHOR | human | M | 39 | 0 | yes | no | no | farmer | 1.431 | 0.094 | -0.913 | 0.045 | 11.1428571428571 | rural |
| I-0015-IHO | MDG24216031 | IHOU | human | M | 28 | 0 | yes | no | no | farmer | 1.431 | 0.094 | -0.913 | 0.045 | 11.1428571428571 | rural |
| I-0041-IHO | MDG24216031 | IHOR | human | F | 45 | 0 | no | no | no | other | 1.431 | 0.094 | -0.913 | 0.045 | 11.1428571428571 | rural |
| I-0024-IHO | MDG24216031 | IHOU | human | F | 57 | 0 | no | no | no | farmer | 1.431 | 0.094 | -0.913 | 0.045 | 11.1428571428571 | rural |
| I-0039-IHO | MDG24216031 | IHOR | human | M | 29 | 0 | no | no | no | farmer | 1.431 | 0.094 | -0.913 | 0.045 | 11.1428571428571 | rural |
| I-0038-IHO | MDG24216031 | IHOR | human | M | 23 | 0 | no | no | no | other | 1.431 | 0.094 | -0.913 | 0.045 | 11.1428571428571 | rural |
| I-0037-IHO | MDG24216031 | IHOR | human | M | 27 | 0 | yes | no | no | farmer | 1.431 | 0.094 | -0.913 | 0.045 | 11.1428571428571 | rural |
| I-0036-IHO | MDG24216031 | IHOR | human | M | 33 | 0 | yes | no | no | farmer | 1.431 | 0.094 | -0.913 | 0.045 | 11.1428571428571 | rural |
| I-0035-IHO | MDG24216031 | IHOR | human | M | 38 | 0 | yes | yes | no | farmer | 1.431 | 0.094 | -0.913 | 0.045 | 11.1428571428571 | rural |
| I-0028-IHO | MDG24216031 | IHOU | human | F | 44 | 0 | no | no | no | other | 1.431 | 0.094 | -0.913 | 0.045 | 11.1428571428571 | rural |
| I-0042-IHO | MDG24216031 | IHOR | human | M | 41 | 0 | no | no | no | other | 1.431 | 0.094 | -0.913 | 0.045 | 11.1428571428571 | rural |
| I-0034-IHO | MDG24216031 | IHOR | human | M | 35 | 0 | no | no | no | farmer | 1.431 | 0.094 | -0.913 | 0.045 | 11.1428571428571 | rural |
| I-0025-IHO | MDG24216031 | IHOU | human | M | 49 | 0 | yes | no | no | farmer | 1.431 | 0.094 | -0.913 | 0.045 | 11.1428571428571 | rural |
| I-0027-IHO | MDG24216031 | IHOU | human | M | 20 | 0 | yes | no | no | other | 1.431 | 0.094 | -0.913 | 0.045 | 11.1428571428571 | rural |

| ID | PCODE | site2 | sp | gender | age | IgG | contact_ruminant | contact_milk | contact_fresh_fluid | profession | fact1 | fact2 | fact3 | fact4 | cattle_density | habitat |
| --- | --- | --- | --- | --- | --- | --- | --- | --- | --- | --- | --- | --- | --- | --- | --- | --- |
| I-0029-IHO | MDG24216031 | IHOU | human | M | 33 | 0 | no | no | no | other | 1.431 | 0.094 | -0.913 | 0.045 | 11.1428571428571 | rural |
| I-0030-IHO | MDG24216031 | IHOU | human | M | 36 | 0 | no | no | no | farmer | 1.431 | 0.094 | -0.913 | 0.045 | 11.1428571428571 | rural |
| I-0031-IHO | MDG24216031 | IHOR | human | M | 56 | 0 | yes | yes | no | farmer | 1.431 | 0.094 | -0.913 | 0.045 | 11.1428571428571 | rural |
| I-0032-IHO | MDG24216031 | IHOR | human | F | 28 | 0 | no | no | no | other | 1.431 | 0.094 | -0.913 | 0.045 | 11.1428571428571 | rural |
| I-0033-IHO | MDG24216031 | IHOR | human | M | 46 | 0 | yes | no | no | farmer | 1.431 | 0.094 | -0.913 | 0.045 | 11.1428571428571 | rural |
| I-0026-IHO | MDG24216031 | IHOU | human | M | 23 | 0 | no | no | no | other | 1.431 | 0.094 | -0.913 | 0.045 | 11.1428571428571 | rural |
| I-0058-FIA | MDG21220430 | FIAR | human | M | 31 | 0 | yes | no | no | farmer | -0.604 | 0.716 | 0.369 | -0.378 | 16.3333333333333 | rural |
| I-0048-FIA | MDG21220430 | FIAR | human | M | 49 | 0 | yes | no | no | farmer | -0.604 | 0.716 | 0.369 | -0.378 | 16.3333333333333 | rural |
| I-0049-FIA | MDG21220430 | FIAR | human | F | 43 | 0 | no | no | no | farmer | -0.604 | 0.716 | 0.369 | -0.378 | 16.3333333333333 | rural |
| I-0050-FIA | MDG21220430 | FIAR | human | M | 24 | 1 | yes | no | no | farmer | -0.604 | 0.716 | 0.369 | -0.378 | 16.3333333333333 | rural |
| I-0051-FIA | MDG21220430 | FIAR | human | M | 49 | 0 | no | no | no | farmer | -0.604 | 0.716 | 0.369 | -0.378 | 16.3333333333333 | rural |
| I-0052-FIA | MDG21220430 | FIAR | human | M | 43 | 0 | yes | no | no | other | -0.604 | 0.716 | 0.369 | -0.378 | 16.3333333333333 | rural |
| I-0053-FIA | MDG21220430 | FIAR | human | F | 37 | 0 | yes | no | no | other | -0.604 | 0.716 | 0.369 | -0.378 | 16.3333333333333 | rural |
| I-0054-FIA | MDG21220430 | FIAR | human | M | 27 | 0 | yes | no | no | farmer | -0.604 | 0.716 | 0.369 | -0.378 | 16.3333333333333 | rural |
| I-0055-FIA | MDG21220430 | FIAR | human | M | 25 | 1 | yes | no | no | farmer | -0.604 | 0.716 | 0.369 | -0.378 | 16.3333333333333 | rural |
| I-0060-FIA | MDG21220430 | FIAR | human | F | 65 | 0 | yes | no | no | farmer | -0.604 | 0.716 | 0.369 | -0.378 | 16.3333333333333 | rural |
| I-0057-FIA | MDG21220430 | FIAR | human | F | 19 | 0 | no | no | no | farmer | -0.604 | 0.716 | 0.369 | -0.378 | 16.3333333333333 | rural |
| I-0047-FIA | MDG21220430 | FIAR | human | F | 30 | 0 | yes | no | no | farmer | -0.604 | 0.716 | 0.369 | -0.378 | 16.3333333333333 | rural |
| I-0059-FIA | MDG21220430 | FIAR | human | F | 26 | 0 | no | no | no | farmer | -0.604 | 0.716 | 0.369 | -0.378 | 16.3333333333333 | rural |
| I-0036-FIA | MDG21220430 | FIAR | human | F | 40 | 0 | no | no | no | farmer | -0.604 | 0.716 | 0.369 | -0.378 | 16.3333333333333 | rural |
| I-0056-FIA | MDG21220430 | FIAR | human | F | 21 | 0 | yes | no | no | farmer | -0.604 | 0.716 | 0.369 | -0.378 | 16.3333333333333 | rural |
| I-0037-FIA | MDG21220430 | FIAR | human | F | 21 | 0 | no | no | no | farmer | -0.604 | 0.716 | 0.369 | -0.378 | 16.3333333333333 | rural |
| I-0046-FIA | MDG21220430 | FIAR | human | M | 31 | 0 | no | no | no | contact_env | -0.604 | 0.716 | 0.369 | -0.378 | 16.3333333333333 | rural |
| I-0038-FIA | MDG21220430 | FIAR | human | F | 32 | 0 | no | no | no | other | -0.604 | 0.716 | 0.369 | -0.378 | 16.3333333333333 | rural |
| I-0033-FIA | MDG21220430 | FIAR | human | M | 56 | 0 | yes | no | no | farmer | -0.604 | 0.716 | 0.369 | -0.378 | 16.3333333333333 | rural |
| I-0034-FIA | MDG21220430 | FIAR | human | F | 19 | 0 | no | no | no | farmer | -0.604 | 0.716 | 0.369 | -0.378 | 16.3333333333333 | rural |
| I-0035-FIA | MDG21220430 | FIAR | human | M | 32 | 0 | yes | no | no | farmer | -0.604 | 0.716 | 0.369 | -0.378 | 16.3333333333333 | rural |
| I-0032-FIA | MDG21220430 | FIAR | human | F | 55 | 0 | no | no | no | other | -0.604 | 0.716 | 0.369 | -0.378 | 16.3333333333333 | rural |
| I-0031-FIA | MDG21220430 | FIAR | human | M | 22 | 0 | yes | no | no | other | -0.604 | 0.716 | 0.369 | -0.378 | 16.3333333333333 | rural |
| I-0039-FIA | MDG21220430 | FIAR | human | M | 54 | 0 | no | no | no | farmer | -0.604 | 0.716 | 0.369 | -0.378 | 16.3333333333333 | rural |
| I-0040-FIA | MDG21220430 | FIAR | human | M | 75 | 0 | yes | no | no | farmer | -0.604 | 0.716 | 0.369 | -0.378 | 16.3333333333333 | rural |
| I-0041-FIA | MDG21220430 | FIAR | human | F | 56 | 0 | yes | no | no | farmer | -0.604 | 0.716 | 0.369 | -0.378 | 16.3333333333333 | rural |
| I-0042-FIA | MDG21220430 | FIAR | human | F | 41 | 0 | yes | no | no | farmer | -0.604 | 0.716 | 0.369 | -0.378 | 16.3333333333333 | rural |
| I-0043-FIA | MDG21220430 | FIAR | human | M | 45 | 0 | yes | no | no | farmer | -0.604 | 0.716 | 0.369 | -0.378 | 16.3333333333333 | rural |
| I-0044-FIA | MDG21220430 | FIAR | human | F | 48 | 1 | no | no | no | other | -0.604 | 0.716 | 0.369 | -0.378 | 16.3333333333333 | rural |
| I-0045-FIA | MDG21220430 | FIAR | human | M | 55 | 0 | yes | no | no | farmer | -0.604 | 0.716 | 0.369 | -0.378 | 16.3333333333333 | rural |
| I-0023-TOA | MDG31301004 | TOAU | human | F | 32 | 0 | no | no | no | other | -0.125 | 0.635 | 1.750 | 0.335 | 12.5 | urban |
| I-0017-TOA | MDG31301004 | TOAU | human | F | 55 | 0 | no | no | no | other | -0.125 | 0.635 | 1.750 | 0.335 | 12.5 | urban |
| I-0018-TOA | MDG31301004 | TOAU | human | F | 53 | 0 | no | no | no | other | -0.125 | 0.635 | 1.750 | 0.335 | 12.5 | urban |
| I-0019-TOA | MDG31301004 | TOAU | human | F | 26 | 0 | no | no | no | other | -0.125 | 0.635 | 1.750 | 0.335 | 12.5 | urban |
| I-0020-TOA | MDG31301004 | TOAU | human | M | 46 | 0 | no | no | no | other | -0.125 | 0.635 | 1.750 | 0.335 | 12.5 | urban |
| I-0021-TOA | MDG31301004 | TOAU | human | F | 20 | 0 | no | no | no | other | -0.125 | 0.635 | 1.750 | 0.335 | 12.5 | urban |
| I-0022-TOA | MDG31301004 | TOAU | human | F | 24 | 0 | no | no | no | other | -0.125 | 0.635 | 1.750 | 0.335 | 12.5 | urban |
| I-0024-TOA | MDG31301004 | TOAU | human | M | 26 | 0 | no | no | no | other | -0.125 | 0.635 | 1.750 | 0.335 | 12.5 | urban |
| I-0025-TOA | MDG31301004 | TOAU | human | F | 53 | 0 | no | no | no | other | -0.125 | 0.635 | 1.750 | 0.335 | 12.5 | urban |
| I-0026-TOA | MDG31301004 | TOAU | human | F | 42 | 0 | no | no | no | other | -0.125 | 0.635 | 1.750 | 0.335 | 12.5 | urban |
| I-0027-TOA | MDG31301004 | TOAU | human | M | 19 | 0 | no | no | no | other | -0.125 | 0.635 | 1.750 | 0.335 | 12.5 | urban |
| I-0028-TOA | MDG31301004 | TOAU | human | M | 44 | 0 | no | no | no | other | -0.125 | 0.635 | 1.750 | 0.335 | 12.5 | urban |
| I-0029-TOA | MDG31301004 | TOAU | human | M | 20 | 0 | no | no | no | other | -0.125 | 0.635 | 1.750 | 0.335 | 12.5 | urban |
| I-0016-TOA | MDG31301004 | TOAU | human | M | 58 | 0 | no | no | no | other | -0.125 | 0.635 | 1.750 | 0.335 | 12.5 | urban |
| I-0030-TOA | MDG31301004 | TOAU | human | M | 28 | 0 | no | no | no | other | -0.125 | 0.635 | 1.750 | 0.335 | 12.5 | urban |
| I-0001-TOA | MDG31301004 | TOAU | human | F | 30 | 0 | no | no | no | other | -0.125 | 0.635 | 1.750 | 0.335 | 12.5 | urban |
| I-0015-TOA | MDG31301004 | TOAU | human | M | 29 | 0 | no | no | no | other | -0.125 | 0.635 | 1.750 | 0.335 | 12.5 | urban |
| I-0002-TOA | MDG31301004 | TOAU | human | M | 24 | 0 | no | no | no | other | -0.125 | 0.635 | 1.750 | 0.335 | 12.5 | urban |
| I-0003-TOA | MDG31301004 | TOAU | human | F | 19 | 0 | no | no | no | other | -0.125 | 0.635 | 1.750 | 0.335 | 12.5 | urban |
| I-0004-TOA | MDG31301004 | TOAU | human | F | 21 | 0 | no | no | no | other | -0.125 | 0.635 | 1.750 | 0.335 | 12.5 | urban |
| I-0005-TOA | MDG31301004 | TOAU | human | F | 19 | 0 | no | no | no | other | -0.125 | 0.635 | 1.750 | 0.335 | 12.5 | urban |
| I-0006-TOA | MDG31301004 | TOAU | human | F | 40 | 0 | no | no | no | other | -0.125 | 0.635 | 1.750 | 0.335 | 12.5 | urban |
| I-0007-TOA | MDG31301004 | TOAU | human | F | 43 | 0 | no | no | no | other | -0.125 | 0.635 | 1.750 | 0.335 | 12.5 | urban |
| I-0009-TOA | MDG31301004 | TOAU | human | M | 63 | 0 | no | no | no | other | -0.125 | 0.635 | 1.750 | 0.335 | 12.5 | urban |
| I-0010-TOA | MDG31301004 | TOAU | human | M | 27 | 0 | no | no | no | other | -0.125 | 0.635 | 1.750 | 0.335 | 12.5 | urban |
| I-0011-TOA | MDG31301004 | TOAU | human | F | 51 | 0 | no | no | no | other | -0.125 | 0.635 | 1.750 | 0.335 | 12.5 | urban |
| I-0012-TOA | MDG31301004 | TOAU | human | M | 25 | 0 | no | no | no | other | -0.125 | 0.635 | 1.750 | 0.335 | 12.5 | urban |

| ID | PCODE | site2 | sp | gender | age | IgG | contact_ruminant | contact_milk | contact_fresh_fluid | profession | fact1 | fact2 | fact3 | fact4 | cattle_density | habitat |
| --- | --- | --- | --- | --- | --- | --- | --- | --- | --- | --- | --- | --- | --- | --- | --- | --- |
| I-0013-TOA | MDG31301004 | TOAU | human | M | 20 | 0 | no | no | no | other | -0.125 | 0.635 | 1.750 | 0.335 | 12.5 | urban |
| I-0014-TOA | MDG31301004 | TOAU | human | M | 39 | 0 | no | no | no | other | -0.125 | 0.635 | 1.750 | 0.335 | 12.5 | urban |
| I-0008-TOA | MDG31301004 | TOAU | human | M | 41 | 0 | no | no | no | other | -0.125 | 0.635 | 1.750 | 0.335 | 12.5 | urban |
| I-0058-TOA | MDG31310030 | TOAR | human | F | 35 | 0 | no | no | no | farmer | -1.858 | -1.024 | 0.958 | 1.088 | 2.75 | rural |
| I-0048-TOA | MDG31310030 | TOAR | human | M | 45 | 1 | no | no | no | contact_env | -1.858 | -1.024 | 0.958 | 1.088 | 2.75 | rural |
| I-0049-TOA | MDG31310030 | TOAR | human | F | 31 | 0 | no | no | no | other | -1.858 | -1.024 | 0.958 | 1.088 | 2.75 | rural |
| I-0050-TOA | MDG31310030 | TOAR | human | M | 37 | 1 | yes | no | no | contact_env | -1.858 | -1.024 | 0.958 | 1.088 | 2.75 | rural |
| I-0051-TOA | MDG31310030 | TOAR | human | M | 41 | 0 | no | no | no | other | -1.858 | -1.024 | 0.958 | 1.088 | 2.75 | rural |
| I-0052-TOA | MDG31310030 | TOAR | human | F | 33 | 0 | no | no | no | farmer | -1.858 | -1.024 | 0.958 | 1.088 | 2.75 | rural |
| I-0053-TOA | MDG31310030 | TOAR | human | F | 22 | 0 | no | no | no | farmer | -1.858 | -1.024 | 0.958 | 1.088 | 2.75 | rural |
| I-0054-TOA | MDG31310030 | TOAR | human | F | 36 | 0 | yes | no | no | other | -1.858 | -1.024 | 0.958 | 1.088 | 2.75 | rural |
| I-0055-TOA | MDG31310030 | TOAR | human | M | 32 | 0 | yes | no | no | farmer | -1.858 | -1.024 | 0.958 | 1.088 | 2.75 | rural |
| I-0060-TOA | MDG31310030 | TOAR | human | M | 21 | 0 | no | no | no | contact_env | -1.858 | -1.024 | 0.958 | 1.088 | 2.75 | rural |
| I-0057-TOA | MDG31310030 | TOAR | human | F | 20 | 0 | yes | no | no | other | -1.858 | -1.024 | 0.958 | 1.088 | 2.75 | rural |
| I-0059-TOA | MDG31310030 | TOAR | human | F | 29 | 0 | no | no | no | other | -1.858 | -1.024 | 0.958 | 1.088 | 2.75 | rural |
| I-0046-TOA | MDG31310030 | TOAR | human | F | 32 | 0 | no | no | no | other | -1.858 | -1.024 | 0.958 | 1.088 | 2.75 | rural |
| I-0045-TOA | MDG31310030 | TOAR | human | M | 56 | 0 | no | no | no | farmer | -1.858 | -1.024 | 0.958 | 1.088 | 2.75 | rural |
| I-0056-TOA | MDG31310030 | TOAR | human | M | 21 | 1 | yes | no | no | contact_env | -1.858 | -1.024 | 0.958 | 1.088 | 2.75 | rural |
| I-0032-TOA | MDG31310030 | TOAR | human | M | 33 | 0 | no | no | no | farmer | -1.858 | -1.024 | 0.958 | 1.088 | 2.75 | rural |
| I-0047-TOA | MDG31310030 | TOAR | human | F | 52 | 0 | yes | no | no | other | -1.858 | -1.024 | 0.958 | 1.088 | 2.75 | rural |
| I-0031-TOA | MDG31310030 | TOAR | human | M | 39 | 0 | no | no | no | other | -1.858 | -1.024 | 0.958 | 1.088 | 2.75 | rural |
| I-0033-TOA | MDG31310030 | TOAR | human | M | 28 | 0 | yes | no | no | contact_env | -1.858 | -1.024 | 0.958 | 1.088 | 2.75 | rural |
| I-0034-TOA | MDG31310030 | TOAR | human | F | 38 | 0 | no | no | no | other | -1.858 | -1.024 | 0.958 | 1.088 | 2.75 | rural |
| I-0035-TOA | MDG31310030 | TOAR | human | F | 49 | 0 | no | no | no | other | -1.858 | -1.024 | 0.958 | 1.088 | 2.75 | rural |
| I-0036-TOA | MDG31310030 | TOAR | human | M | 22 | 0 | no | no | no | contact_env | -1.858 | -1.024 | 0.958 | 1.088 | 2.75 | rural |
| I-0037-TOA | MDG31310030 | TOAR | human | M | 32 | 0 | no | no | no | contact_env | -1.858 | -1.024 | 0.958 | 1.088 | 2.75 | rural |
| I-0039-TOA | MDG31310030 | TOAR | human | F | 25 | 0 | no | no | no | other | -1.858 | -1.024 | 0.958 | 1.088 | 2.75 | rural |
| I-0040-TOA | MDG31310030 | TOAR | human | F | 20 | 0 | no | no | no | other | -1.858 | -1.024 | 0.958 | 1.088 | 2.75 | rural |
| I-0041-TOA | MDG31310030 | TOAR | human | M | 49 | 0 | no | no | no | farmer | -1.858 | -1.024 | 0.958 | 1.088 | 2.75 | rural |
| I-0042-TOA | MDG31310030 | TOAR | human | F | 59 | 0 | no | no | no | other | -1.858 | -1.024 | 0.958 | 1.088 | 2.75 | rural |
| I-0043-TOA | MDG31310030 | TOAR | human | M | 44 | 0 | no | no | no | farmer | -1.858 | -1.024 | 0.958 | 1.088 | 2.75 | rural |
| I-0044-TOA | MDG31310030 | TOAR | human | F | 63 | 0 | no | no | no | other | -1.858 | -1.024 | 0.958 | 1.088 | 2.75 | rural |
| I-0038-TOA | MDG31310030 | TOAR | human | M | 27 | 1 | no | no | no | contact_env | -1.858 | -1.024 | 0.958 | 1.088 | 2.75 | rural |
| I-0028-ABZ | MDG33313010 | ABZU | human | F | 52 | 0 | no | no | no | other | 0.533 | 0.217 | -1.024 | 0.387 | 5 | rural |
| I-0018-ABZ | MDG33313010 | ABZU | human | M | 61 | 0 | no | no | no | other | 0.533 | 0.217 | -1.024 | 0.387 | 5 | rural |
| I-0019-ABZ | MDG33313010 | ABZU | human | M | 19 | 0 | no | no | no | other | 0.533 | 0.217 | -1.024 | 0.387 | 5 | rural |
| I-0020-ABZ | MDG33313010 | ABZU | human | M | 22 | 0 | no | no | no | other | 0.533 | 0.217 | -1.024 | 0.387 | 5 | rural |
| I-0021-ABZ | MDG33313010 | ABZU | human | F | 28 | 0 | no | no | no | other | 0.533 | 0.217 | -1.024 | 0.387 | 5 | rural |
| I-0022-ABZ | MDG33313010 | ABZU | human | F | 45 | 0 | no | no | no | farmer | 0.533 | 0.217 | -1.024 | 0.387 | 5 | rural |
| I-0023-ABZ | MDG33313010 | ABZU | human | F | 19 | 0 | no | no | no | other | 0.533 | 0.217 | -1.024 | 0.387 | 5 | rural |
| I-0024-ABZ | MDG33313010 | ABZU | human | M | 49 | 0 | no | no | no | farmer | 0.533 | 0.217 | -1.024 | 0.387 | 5 | rural |
| I-0025-ABZ | MDG33313010 | ABZU | human | M | 24 | 0 | no | no | no | other | 0.533 | 0.217 | -1.024 | 0.387 | 5 | rural |
| I-0029-ABZ | MDG33313010 | ABZU | human | F | 28 | 0 | no | no | no | other | 0.533 | 0.217 | -1.024 | 0.387 | 5 | rural |
| I-0027-ABZ | MDG33313010 | ABZU | human | F | 26 | 0 | no | no | no | other | 0.533 | 0.217 | -1.024 | 0.387 | 5 | rural |
| I-0030-ABZ | MDG33313010 | ABZU | human | M | 36 | 0 | no | no | no | farmer | 0.533 | 0.217 | -1.024 | 0.387 | 5 | rural |
| I-0017-ABZ | MDG33313010 | ABZU | human | F | 23 | 0 | no | no | no | other | 0.533 | 0.217 | -1.024 | 0.387 | 5 | rural |
| I-0001-ABZ | MDG33313010 | ABZU | human | M | 19 | 0 | no | no | no | other | 0.533 | 0.217 | -1.024 | 0.387 | 5 | rural |
| I-0026-ABZ | MDG33313010 | ABZU | human | F | 20 | 0 | no | no | no | other | 0.533 | 0.217 | -1.024 | 0.387 | 5 | rural |
| I-0005-ABZ | MDG33313010 | ABZU | human | M | 41 | 0 | no | no | no | other | 0.533 | 0.217 | -1.024 | 0.387 | 5 | rural |
| I-0003-ABZ | MDG33313010 | ABZU | human | F | 41 | 0 | no | no | no | other | 0.533 | 0.217 | -1.024 | 0.387 | 5 | rural |
| I-0016-ABZ | MDG33313010 | ABZU | human | M | 59 | 0 | no | no | no | other | 0.533 | 0.217 | -1.024 | 0.387 | 5 | rural |
| I-0004-ABZ | MDG33313010 | ABZU | human | F | 48 | 0 | no | no | no | other | 0.533 | 0.217 | -1.024 | 0.387 | 5 | rural |
| I-0006-ABZ | MDG33313010 | ABZU | human | M | 42 | 0 | no | no | no | other | 0.533 | 0.217 | -1.024 | 0.387 | 5 | rural |
| I-0007-ABZ | MDG33313010 | ABZU | human | F | 33 | 0 | no | no | no | farmer | 0.533 | 0.217 | -1.024 | 0.387 | 5 | rural |
| I-0008-ABZ | MDG33313010 | ABZU | human | F | 52 | 0 | no | no | no | other | 0.533 | 0.217 | -1.024 | 0.387 | 5 | rural |
| I-0014-ABZ | MDG33313010 | ABZU | human | F | 40 | 0 | no | no | no | other | 0.533 | 0.217 | -1.024 | 0.387 | 5 | rural |
| I-0010-ABZ | MDG33313010 | ABZU | human | M | 41 | 0 | no | no | no | other | 0.533 | 0.217 | -1.024 | 0.387 | 5 | rural |
| I-0011-ABZ | MDG33313010 | ABZU | human | M | 26 | 0 | no | no | no | farmer | 0.533 | 0.217 | -1.024 | 0.387 | 5 | rural |
| I-0012-ABZ | MDG33313010 | ABZU | human | F | 19 | 0 | no | no | no | other | 0.533 | 0.217 | -1.024 | 0.387 | 5 | rural |
| I-0013-ABZ | MDG33313010 | ABZU | human | F | 18 | 0 | no | no | no | other | 0.533 | 0.217 | -1.024 | 0.387 | 5 | rural |
| I-0009-ABZ | MDG33313010 | ABZU | human | M | 55 | 0 | no | no | no | farmer | 0.533 | 0.217 | -1.024 | 0.387 | 5 | rural |
| I-0002-ABZ | MDG33313010 | ABZU | human | F | 27 | 0 | no | no | no | farmer | 0.533 | 0.217 | -1.024 | 0.387 | 5 | rural |
| I-0015-ABZ | MDG33313010 | ABZU | human | F | 43 | 0 | no | no | no | other | 0.533 | 0.217 | -1.024 | 0.387 | 5 | rural |

| ID | PCODE | site2 | sp | gender | age | IgG | contact_ruminant | contact_milk | contact_fresh_fluid | profession | fact1 | fact2 | fact3 | fact4 | cattle_density | habitat |
| --- | --- | --- | --- | --- | --- | --- | --- | --- | --- | --- | --- | --- | --- | --- | --- | --- |
| I-0056-ABZ | MDG33313090 | ABZR | human | F | 80 | 0 | yes | no | no | other | -0.054 | 0.440 | 0.023 | 0.321 | 7.75 | rural |
| I-0055-ABZ | MDG33313090 | ABZR | human | F | 46 | 0 | no | no | no | farmer | -0.054 | 0.440 | 0.023 | 0.321 | 7.75 | rural |
| I-0054-ABZ | MDG33313090 | ABZR | human | M | 21 | 0 | yes | no | no | farmer | -0.054 | 0.440 | 0.023 | 0.321 | 7.75 | rural |
| I-0053-ABZ | MDG33313090 | ABZR | human | F | 35 | 0 | no | no | no | other | -0.054 | 0.440 | 0.023 | 0.321 | 7.75 | rural |
| I-0052-ABZ | MDG33313090 | ABZR | human | M | 23 | 0 | yes | no | no | farmer | -0.054 | 0.440 | 0.023 | 0.321 | 7.75 | rural |
| I-0050-ABZ | MDG33313090 | ABZR | human | F | 57 | 0 | no | no | no | other | -0.054 | 0.440 | 0.023 | 0.321 | 7.75 | rural |
| I-0057-ABZ | MDG33313090 | ABZR | human | F | 57 | 0 | yes | no | no | other | -0.054 | 0.440 | 0.023 | 0.321 | 7.75 | rural |
| I-0051-ABZ | MDG33313090 | ABZR | human | F | 38 | 0 | no | no | no | other | -0.054 | 0.440 | 0.023 | 0.321 | 7.75 | rural |
| I-0058-ABZ | MDG33313090 | ABZR | human | F | 57 | 0 | no | no | no | farmer | -0.054 | 0.440 | 0.023 | 0.321 | 7.75 | rural |
| I-0059-ABZ | MDG33313090 | ABZR | human | F | 59 | 0 | yes | no | no | farmer | -0.054 | 0.440 | 0.023 | 0.321 | 7.75 | rural |
| I-0060-ABZ | MDG33313090 | ABZR | human | F | 30 | 0 | yes | no | no | farmer | -0.054 | 0.440 | 0.023 | 0.321 | 7.75 | rural |
| I-0034-ABZ | MDG33313090 | ABZR | human | M | 29 | 0 | no | no | no | other | -0.054 | 0.440 | 0.023 | 0.321 | 7.75 | rural |
| I-0049-ABZ | MDG33313090 | ABZR | human | F | 71 | 0 | no | no | no | farmer | -0.054 | 0.440 | 0.023 | 0.321 | 7.75 | rural |
| I-0031-ABZ | MDG33313090 | ABZR | human | M | 64 | 0 | no | no | no | farmer | -0.054 | 0.440 | 0.023 | 0.321 | 7.75 | rural |
| I-0036-ABZ | MDG33313090 | ABZR | human | F | 22 | 0 | yes | no | no | farmer | -0.054 | 0.440 | 0.023 | 0.321 | 7.75 | rural |
| I-0032-ABZ | MDG33313090 | ABZR | human | F | 51 | 0 | no | no | no | health | -0.054 | 0.440 | 0.023 | 0.321 | 7.75 | rural |
| I-0033-ABZ | MDG33313090 | ABZR | human | F | 77 | 0 | no | no | no | other | -0.054 | 0.440 | 0.023 | 0.321 | 7.75 | rural |
| I-0035-ABZ | MDG33313090 | ABZR | human | F | 55 | 0 | no | no | no | farmer | -0.054 | 0.440 | 0.023 | 0.321 | 7.75 | rural |
| I-0037-ABZ | MDG33313090 | ABZR | human | F | 34 | 0 | no | no | no | farmer | -0.054 | 0.440 | 0.023 | 0.321 | 7.75 | rural |
| I-0038-ABZ | MDG33313090 | ABZR | human | F | 24 | 0 | no | no | no | other | -0.054 | 0.440 | 0.023 | 0.321 | 7.75 | rural |
| I-0039-ABZ | MDG33313090 | ABZR | human | M | 58 | 0 | no | no | no | other | -0.054 | 0.440 | 0.023 | 0.321 | 7.75 | rural |
| I-0047-ABZ | MDG33313090 | ABZR | human | F | 20 | 0 | no | no | no | other | -0.054 | 0.440 | 0.023 | 0.321 | 7.75 | rural |
| I-0041-ABZ | MDG33313090 | ABZR | human | M | 33 | 0 | no | no | no | farmer | -0.054 | 0.440 | 0.023 | 0.321 | 7.75 | rural |
| I-0042-ABZ | MDG33313090 | ABZR | human | F | 24 | 0 | no | no | no | farmer | -0.054 | 0.440 | 0.023 | 0.321 | 7.75 | rural |
| I-0043-ABZ | MDG33313090 | ABZR | human | F | 45 | 0 | no | no | no | farmer | -0.054 | 0.440 | 0.023 | 0.321 | 7.75 | rural |
| I-0044-ABZ | MDG33313090 | ABZR | human | M | 26 | 0 | no | no | no | farmer | -0.054 | 0.440 | 0.023 | 0.321 | 7.75 | rural |
| I-0045-ABZ | MDG33313090 | ABZR | human | M | 50 | 0 | no | no | no | farmer | -0.054 | 0.440 | 0.023 | 0.321 | 7.75 | rural |
| I-0046-ABZ | MDG33313090 | ABZR | human | F | 36 | 0 | no | no | no | farmer | -0.054 | 0.440 | 0.023 | 0.321 | 7.75 | rural |
| I-0048-ABZ | MDG33313090 | ABZR | human | M | 56 | 0 | no | no | no | farmer | -0.054 | 0.440 | 0.023 | 0.321 | 7.75 | rural |
| I-0040-ABZ | MDG33313090 | ABZR | human | F | 35 | 0 | no | no | no | other | -0.054 | 0.440 | 0.023 | 0.321 | 7.75 | rural |
| I-0021-MRG | MDG33314010 | MRGU | human | F | 66 | 0 | no | no | no | other | -1.184 | 1.058 | -0.102 | -0.220 | 11.6666666666666 | rural |
| I-0020-MRG | MDG33314010 | MRGU | human | M | 28 | 0 | no | no | no | other | -1.184 | 1.058 | -0.102 | -0.220 | 11.6666666666666 | rural |
| I-0019-MRG | MDG33314010 | MRGU | human | M | 56 | 0 | no | no | no | other | -1.184 | 1.058 | -0.102 | -0.220 | 11.6666666666666 | rural |
| I-0022-MRG | MDG33314010 | MRGU | human | M | 19 | 0 | no | no | no | other | -1.184 | 1.058 | -0.102 | -0.220 | 11.6666666666666 | rural |
| I-0017-MRG | MDG33314010 | MRGU | human | M | 47 | 0 | no | no | no | farmer | -1.184 | 1.058 | -0.102 | -0.220 | 11.6666666666666 | rural |
| I-0028-MRG | MDG33314010 | MRGU | human | M | 38 | 0 | no | no | no | other | -1.184 | 1.058 | -0.102 | -0.220 | 11.6666666666666 | rural |
| I-0018-MRG | MDG33314010 | MRGU | human | M | 41 | 0 | no | no | no | other | -1.184 | 1.058 | -0.102 | -0.220 | 11.6666666666666 | rural |
| I-0023-MRG | MDG33314010 | MRGU | human | M | 27 | 0 | no | no | no | other | -1.184 | 1.058 | -0.102 | -0.220 | 11.6666666666666 | rural |
| I-0024-MRG | MDG33314010 | MRGU | human | M | 27 | 0 | no | no | yes | butcher | -1.184 | 1.058 | -0.102 | -0.220 | 11.6666666666666 | rural |
| I-0025-MRG | MDG33314010 | MRGU | human | F | 56 | 0 | no | no | no | other | -1.184 | 1.058 | -0.102 | -0.220 | 11.6666666666666 | rural |
| I-0027-MRG | MDG33314010 | MRGU | human | F | 30 | 0 | no | no | no | other | -1.184 | 1.058 | -0.102 | -0.220 | 11.6666666666666 | rural |
| I-0030-MRG | MDG33314010 | MRGU | human | F | 19 | 0 | no | no | no | other | -1.184 | 1.058 | -0.102 | -0.220 | 11.6666666666666 | rural |
| I-0016-MRG | MDG33314010 | MRGU | human | M | 38 | 0 | no | no | no | other | -1.184 | 1.058 | -0.102 | -0.220 | 11.6666666666666 | rural |
| I-0029-MRG | MDG33314010 | MRGU | human | F | 37 | 0 | no | no | no | other | -1.184 | 1.058 | -0.102 | -0.220 | 11.6666666666666 | rural |
| I-0026-MRG | MDG33314010 | MRGU | human | M | 40 | 0 | no | no | no | other | -1.184 | 1.058 | -0.102 | -0.220 | 11.6666666666666 | rural |
| I-0002-MRG | MDG33314010 | MRGU | human | F | 38 | 0 | no | no | no | other | -1.184 | 1.058 | -0.102 | -0.220 | 11.6666666666666 | rural |
| I-0015-MRG | MDG33314010 | MRGU | human | F | 60 | 0 | no | no | no | other | -1.184 | 1.058 | -0.102 | -0.220 | 11.6666666666666 | rural |
| I-0001-MRG | MDG33314010 | MRGU | human | F | 38 | 0 | no | no | no | other | -1.184 | 1.058 | -0.102 | -0.220 | 11.6666666666666 | rural |
| I-0003-MRG | MDG33314010 | MRGU | human | F | 19 | 0 | no | no | no | other | -1.184 | 1.058 | -0.102 | -0.220 | 11.6666666666666 | rural |
| I-0004-MRG | MDG33314010 | MRGU | human | F | 19 | 0 | no | no | no | other | -1.184 | 1.058 | -0.102 | -0.220 | 11.6666666666666 | rural |
| I-0005-MRG | MDG33314010 | MRGU | human | F | 28 | 1 | no | no | no | other | -1.184 | 1.058 | -0.102 | -0.220 | 11.6666666666666 | rural |
| I-0006-MRG | MDG33314010 | MRGU | human | M | 35 | 0 | no | no | no | other | -1.184 | 1.058 | -0.102 | -0.220 | 11.6666666666666 | rural |
| I-0007-MRG | MDG33314010 | MRGU | human | F | 39 | 0 | no | no | no | other | -1.184 | 1.058 | -0.102 | -0.220 | 11.6666666666666 | rural |
| I-0009-MRG | MDG33314010 | MRGU | human | F | 37 | 0 | no | no | no | other | -1.184 | 1.058 | -0.102 | -0.220 | 11.6666666666666 | rural |
| I-0010-MRG | MDG33314010 | MRGU | human | F | 51 | 0 | no | no | no | other | -1.184 | 1.058 | -0.102 | -0.220 | 11.6666666666666 | rural |
| I-0011-MRG | MDG33314010 | MRGU | human | M | 49 | 0 | no | no | no | other | -1.184 | 1.058 | -0.102 | -0.220 | 11.6666666666666 | rural |
| I-0012-MRG | MDG33314010 | MRGU | human | M | 32 | 0 | no | no | no | other | -1.184 | 1.058 | -0.102 | -0.220 | 11.6666666666666 | rural |
| I-0014-MRG | MDG33314010 | MRGU | human | F | 34 | 0 | no | no | no | other | -1.184 | 1.058 | -0.102 | -0.220 | 11.6666666666666 | rural |
| I-0013-MRG | MDG33314010 | MRGU | human | M | 21 | 0 | no | no | no | other | -1.184 | 1.058 | -0.102 | -0.220 | 11.6666666666666 | rural |
| I-0008-MRG | MDG33314010 | MRGU | human | F | 23 | 0 | no | no | no | other | -1.184 | 1.058 | -0.102 | -0.220 | 11.6666666666666 | rural |
| I-0058-MRG | MDG33314030 | MRGR | human | M | 47 | 0 | no | no | no | other | -1.363 | 0.363 | -0.104 | -0.636 | 14.9090909090909 | rural |
| I-0048-MRG | MDG33314030 | MRGR | human | F | 41 | 0 | no | no | no | other | -1.363 | 0.363 | -0.104 | -0.636 | 14.9090909090909 | rural |
| I-0049-MRG | MDG33314030 | MRGR | human | M | 20 | 0 | no | no | no | other | -1.363 | 0.363 | -0.104 | -0.636 | 14.9090909090909 | rural |

| ID | PCODE | site2 | sp | gender | age | IgG | contact_ruminant | contact_milk | contact_fresh_fluid | profession | fact1 | fact2 | fact3 | fact4 | cattle_density | habitat |
| --- | --- | --- | --- | --- | --- | --- | --- | --- | --- | --- | --- | --- | --- | --- | --- | --- |
| I-0050-MRG | MDG33314030 | MRGR | human | F | 19 | 0 | no | no | no | other | -1.363 | 0.363 | -0.104 | -0.636 | 14.9090909090909 | rural |
| I-0051-MRG | MDG33314030 | MRGR | human | F | 48 | 0 | no | no | no | farmer | -1.363 | 0.363 | -0.104 | -0.636 | 14.9090909090909 | rural |
| I-0052-MRG | MDG33314030 | MRGR | human | M | 55 | 1 | yes | no | no | farmer | -1.363 | 0.363 | -0.104 | -0.636 | 14.9090909090909 | rural |
| I-0053-MRG | MDG33314030 | MRGR | human | F | 42 | 0 | no | no | no | farmer | -1.363 | 0.363 | -0.104 | -0.636 | 14.9090909090909 | rural |
| I-0054-MRG | MDG33314030 | MRGR | human | M | 49 | 0 | yes | no | no | farmer | -1.363 | 0.363 | -0.104 | -0.636 | 14.9090909090909 | rural |
| I-0055-MRG | MDG33314030 | MRGR | human | F | 25 | 0 | no | no | no | farmer | -1.363 | 0.363 | -0.104 | -0.636 | 14.9090909090909 | rural |
| I-0060-MRG | MDG33314030 | MRGR | human | F | 29 | 0 | no | no | no | farmer | -1.363 | 0.363 | -0.104 | -0.636 | 14.9090909090909 | rural |
| I-0057-MRG | MDG33314030 | MRGR | human | F | 43 | 0 | no | no | no | other | -1.363 | 0.363 | -0.104 | -0.636 | 14.9090909090909 | rural |
| I-0059-MRG | MDG33314030 | MRGR | human | F | 59 | 0 | no | no | no | farmer | -1.363 | 0.363 | -0.104 | -0.636 | 14.9090909090909 | rural |
| I-0031-MRG | MDG33314030 | MRGR | human | M | 19 | 0 | no | no | no | other | -1.363 | 0.363 | -0.104 | -0.636 | 14.9090909090909 | rural |
| I-0045-MRG | MDG33314030 | MRGR | human | F | 38 | 0 | no | no | no | farmer | -1.363 | 0.363 | -0.104 | -0.636 | 14.9090909090909 | rural |
| I-0056-MRG | MDG33314030 | MRGR | human | M | 67 | 0 | no | no | no | farmer | -1.363 | 0.363 | -0.104 | -0.636 | 14.9090909090909 | rural |
| I-0036-MRG | MDG33314030 | MRGR | human | M | 74 | 0 | no | no | no | other | -1.363 | 0.363 | -0.104 | -0.636 | 14.9090909090909 | rural |
| I-0047-MRG | MDG33314030 | MRGR | human | F | 30 | 0 | yes | no | no | other | -1.363 | 0.363 | -0.104 | -0.636 | 14.9090909090909 | rural |
| I-0046-MRG | MDG33314030 | MRGR | human | M | 41 | 0 | yes | no | no | farmer | -1.363 | 0.363 | -0.104 | -0.636 | 14.9090909090909 | rural |
| I-0033-MRG | MDG33314030 | MRGR | human | M | 51 | 0 | no | no | no | other | -1.363 | 0.363 | -0.104 | -0.636 | 14.9090909090909 | rural |
| I-0035-MRG | MDG33314030 | MRGR | human | F | 53 | 0 | no | no | no | other | -1.363 | 0.363 | -0.104 | -0.636 | 14.9090909090909 | rural |
| I-0032-MRG | MDG33314030 | MRGR | human | F | 45 | 0 | no | no | no | other | -1.363 | 0.363 | -0.104 | -0.636 | 14.9090909090909 | rural |
| I-0037-MRG | MDG33314030 | MRGR | human | F | 59 | 0 | no | no | no | farmer | -1.363 | 0.363 | -0.104 | -0.636 | 14.9090909090909 | rural |
| I-0038-MRG | MDG33314030 | MRGR | human | F | 35 | 0 | no | no | no | farmer | -1.363 | 0.363 | -0.104 | -0.636 | 14.9090909090909 | rural |
| I-0039-MRG | MDG33314030 | MRGR | human | M | 34 | 0 | no | no | no | farmer | -1.363 | 0.363 | -0.104 | -0.636 | 14.9090909090909 | rural |
| I-0040-MRG | MDG33314030 | MRGR | human | F | 25 | 0 | no | no | no | other | -1.363 | 0.363 | -0.104 | -0.636 | 14.9090909090909 | rural |
| I-0041-MRG | MDG33314030 | MRGR | human | F | 46 | 0 | no | no | no | farmer | -1.363 | 0.363 | -0.104 | -0.636 | 14.9090909090909 | rural |
| I-0042-MRG | MDG33314030 | MRGR | human | F | 50 | 0 | yes | no | no | farmer | -1.363 | 0.363 | -0.104 | -0.636 | 14.9090909090909 | rural |
| I-0043-MRG | MDG33314030 | MRGR | human | M | 49 | 0 | no | no | no | other | -1.363 | 0.363 | -0.104 | -0.636 | 14.9090909090909 | rural |
| I-0044-MRG | MDG33314030 | MRGR | human | F | 42 | 0 | no | no | no | other | -1.363 | 0.363 | -0.104 | -0.636 | 14.9090909090909 | rural |
| I-0034-MRG | MDG33314030 | MRGR | human | F | 36 | 0 | no | no | no | farmer | -1.363 | 0.363 | -0.104 | -0.636 | 14.9090909090909 | rural |
| I-0052-MJG | MDG41401001 | MJGU | human | F | 24 | 1 | no | no | no | other | 1.688 | -0.005 | -0.397 | 0.610 | 0 | urban |
| I-0047-MJG | MDG41401001 | MJGU | human | M | 19 | 0 | no | no | no | farmer | 1.688 | -0.005 | -0.397 | 0.610 | 0 | urban |
| I-0060-MJG | MDG41401001 | MJGU | human | F | 42 | 0 | no | no | no | other | 1.688 | -0.005 | -0.397 | 0.610 | 0 | urban |
| I-0049-MJG | MDG41401001 | MJGU | human | F | 33 | 0 | no | no | no | other | 1.688 | -0.005 | -0.397 | 0.610 | 0 | urban |
| I-0050-MJG | MDG41401001 | MJGU | human | F | 40 | 0 | no | no | no | other | 1.688 | -0.005 | -0.397 | 0.610 | 0 | urban |
| I-0051-MJG | MDG41401001 | MJGU | human | F | 26 | 0 | no | no | no | other | 1.688 | -0.005 | -0.397 | 0.610 | 0 | urban |
| I-0053-MJG | MDG41401001 | MJGU | human | M | 43 | 0 | yes | no | no | farmer | 1.688 | -0.005 | -0.397 | 0.610 | 0 | urban |
| I-0054-MJG | MDG41401001 | MJGU | human | M | 47 | 1 | no | no | no | other | 1.688 | -0.005 | -0.397 | 0.610 | 0 | urban |
| I-0055-MJG | MDG41401001 | MJGU | human | F | 39 | 0 | yes | no | no | farmer | 1.688 | -0.005 | -0.397 | 0.610 | 0 | urban |
| I-0056-MJG | MDG41401001 | MJGU | human | M | 32 | 0 | no | no | no | other | 1.688 | -0.005 | -0.397 | 0.610 | 0 | urban |
| I-0057-MJG | MDG41401001 | MJGU | human | F | 23 | 0 | no | no | no | other | 1.688 | -0.005 | -0.397 | 0.610 | 0 | urban |
| I-0058-MJG | MDG41401001 | MJGU | human | F | 46 | 0 | yes | no | no | other | 1.688 | -0.005 | -0.397 | 0.610 | 0 | urban |
| I-0046-MJG | MDG41401001 | MJGU | human | M | 49 | 0 | no | no | no | farmer | 1.688 | -0.005 | -0.397 | 0.610 | 0 | urban |
| I-0059-MJG | MDG41401001 | MJGU | human | M | 57 | 0 | yes | no | no | farmer | 1.688 | -0.005 | -0.397 | 0.610 | 0 | urban |
| I-0031-MJG | MDG41401001 | MJGU | human | F | 37 | 0 | no | no | no | other | 1.688 | -0.005 | -0.397 | 0.610 | 0 | urban |
| I-0048-MJG | MDG41401001 | MJGU | human | M | 53 | 0 | no | no | no | farmer | 1.688 | -0.005 | -0.397 | 0.610 | 0 | urban |
| I-0032-MJG | MDG41401001 | MJGU | human | F | 43 | 0 | no | no | no | other | 1.688 | -0.005 | -0.397 | 0.610 | 0 | urban |
| I-0033-MJG | MDG41401001 | MJGU | human | F | 43 | 0 | no | no | no | other | 1.688 | -0.005 | -0.397 | 0.610 | 0 | urban |
| I-0034-MJG | MDG41401001 | MJGU | human | M | 66 | 0 | no | no | no | other | 1.688 | -0.005 | -0.397 | 0.610 | 0 | urban |
| I-0035-MJG | MDG41401001 | MJGU | human | M | 46 | 0 | no | no | no | other | 1.688 | -0.005 | -0.397 | 0.610 | 0 | urban |
| I-0036-MJG | MDG41401001 | MJGU | human | F | 40 | 0 | no | no | no | other | 1.688 | -0.005 | -0.397 | 0.610 | 0 | urban |
| I-0037-MJG | MDG41401001 | MJGU | human | F | 35 | 0 | yes | no | no | other | 1.688 | -0.005 | -0.397 | 0.610 | 0 | urban |
| I-0039-MJG | MDG41401001 | MJGU | human | F | 50 | 0 | yes | no | no | farmer | 1.688 | -0.005 | -0.397 | 0.610 | 0 | urban |
| I-0040-MJG | MDG41401001 | MJGU | human | M | 21 | 0 | no | no | no | other | 1.688 | -0.005 | -0.397 | 0.610 | 0 | urban |
| I-0041-MJG | MDG41401001 | MJGU | human | F | 37 | 0 | no | no | no | other | 1.688 | -0.005 | -0.397 | 0.610 | 0 | urban |
| I-0042-MJG | MDG41401001 | MJGU | human | M | 24 | 0 | no | no | no | other | 1.688 | -0.005 | -0.397 | 0.610 | 0 | urban |
| I-0043-MJG | MDG41401001 | MJGU | human | F | 43 | 0 | no | no | no | other | 1.688 | -0.005 | -0.397 | 0.610 | 0 | urban |
| I-0044-MJG | MDG41401001 | MJGU | human | M | 39 | 0 | no | no | no | other | 1.688 | -0.005 | -0.397 | 0.610 | 0 | urban |
| I-0038-MJG | MDG41401001 | MJGU | human | M | 39 | 1 | no | no | no | contact_env | 1.688 | -0.005 | -0.397 | 0.610 | 0 | urban |
| I-0045-MJG | MDG41401001 | MJGU | human | M | 50 | 0 | yes | no | no | farmer | 1.688 | -0.005 | -0.397 | 0.610 | 0 | urban |
| I-0027-MAE | MDG43404010 | MAEU | human | F | 35 | 0 | no | no | no | other | 1.957 | -0.869 | 1.298 | 0.944 | 8.5 | rural |
| I-0017-MAE | MDG43404010 | MAEU | human | F | 53 | 0 | no | no | no | other | 1.957 | -0.869 | 1.298 | 0.944 | 8.5 | rural |
| I-0018-MAE | MDG43404010 | MAEU | human | M | 40 | 0 | no | no | no | contact_env | 1.957 | -0.869 | 1.298 | 0.944 | 8.5 | rural |
| I-0019-MAE | MDG43404010 | MAEU | human | F | 19 | 0 | no | no | no | farmer | 1.957 | -0.869 | 1.298 | 0.944 | 8.5 | rural |
| I-0020-MAE | MDG43404010 | MAEU | human | M | 46 | 0 | no | no | no | contact_env | 1.957 | -0.869 | 1.298 | 0.944 | 8.5 | rural |
| I-0021-MAE | MDG43404010 | MAEU | human | F | 31 | 0 | no | no | no | other | 1.957 | -0.869 | 1.298 | 0.944 | 8.5 | rural |

| ID | PCODE | site2 | sp | gender | age | IgG | contact_ruminant | contact_milk | contact_fresh_fluid | profession | fact1 | fact2 | fact3 | fact4 | cattle_density | habitat |
| --- | --- | --- | --- | --- | --- | --- | --- | --- | --- | --- | --- | --- | --- | --- | --- | --- |
| I-0022-MAE | MDG43404010 | MAEU | human | F | 50 | 0 | no | no | no | other | 1.957 | -0.869 | 1.298 | 0.944 | 8.5 | rural |
| I-0023-MAE | MDG43404010 | MAEU | human | M | 30 | 0 | no | no | no | contact_env | 1.957 | -0.869 | 1.298 | 0.944 | 8.5 | rural |
| I-0024-MAE | MDG43404010 | MAEU | human | M | 35 | 0 | no | no | no | other | 1.957 | -0.869 | 1.298 | 0.944 | 8.5 | rural |
| I-0030-MAE | MDG43404010 | MAEU | human | F | 23 | 0 | no | no | no | other | 1.957 | -0.869 | 1.298 | 0.944 | 8.5 | rural |
| I-0026-MAE | MDG43404010 | MAEU | human | F | 35 | 0 | no | no | no | other | 1.957 | -0.869 | 1.298 | 0.944 | 8.5 | rural |
| I-0028-MAE | MDG43404010 | MAEU | human | F | 29 | 0 | no | no | no | other | 1.957 | -0.869 | 1.298 | 0.944 | 8.5 | rural |
| I-0016-MAE | MDG43404010 | MAEU | human | M | 70 | 0 | no | no | no | farmer | 1.957 | -0.869 | 1.298 | 0.944 | 8.5 | rural |
| I-0029-MAE | MDG43404010 | MAEU | human | F | 48 | 0 | no | no | no | other | 1.957 | -0.869 | 1.298 | 0.944 | 8.5 | rural |
| I-0025-MAE | MDG43404010 | MAEU | human | F | 34 | 0 | no | no | no | other | 1.957 | -0.869 | 1.298 | 0.944 | 8.5 | rural |
| I-0006-MAE | MDG43404010 | MAEU | human | F | 33 | 0 | no | no | no | other | 1.957 | -0.869 | 1.298 | 0.944 | 8.5 | rural |
| I-0015-MAE | MDG43404010 | MAEU | human | M | 57 | 0 | no | no | no | other | 1.957 | -0.869 | 1.298 | 0.944 | 8.5 | rural |
| I-0002-MAE | MDG43404010 | MAEU | human | F | 22 | 1 | no | no | yes | other | 1.957 | -0.869 | 1.298 | 0.944 | 8.5 | rural |
| I-0003-MAE | MDG43404010 | MAEU | human | M | 31 | 0 | no | no | no | other | 1.957 | -0.869 | 1.298 | 0.944 | 8.5 | rural |
| I-0005-MAE | MDG43404010 | MAEU | human | F | 23 | 0 | no | no | no | other | 1.957 | -0.869 | 1.298 | 0.944 | 8.5 | rural |
| I-0001-MAE | MDG43404010 | MAEU | human | M | 21 | 0 | no | no | no | other | 1.957 | -0.869 | 1.298 | 0.944 | 8.5 | rural |
| I-0007-MAE | MDG43404010 | MAEU | human | M | 32 | 0 | no | no | no | other | 1.957 | -0.869 | 1.298 | 0.944 | 8.5 | rural |
| I-0008-MAE | MDG43404010 | MAEU | human | F | 47 | 0 | no | no | no | other | 1.957 | -0.869 | 1.298 | 0.944 | 8.5 | rural |
| I-0009-MAE | MDG43404010 | MAEU | human | F | 23 | 0 | no | no | no | other | 1.957 | -0.869 | 1.298 | 0.944 | 8.5 | rural |
| I-0010-MAE | MDG43404010 | MAEU | human | M | 32 | 0 | no | no | no | contact_env | 1.957 | -0.869 | 1.298 | 0.944 | 8.5 | rural |
| I-0011-MAE | MDG43404010 | MAEU | human | F | 73 | 0 | no | no | no | other | 1.957 | -0.869 | 1.298 | 0.944 | 8.5 | rural |
| I-0012-MAE | MDG43404010 | MAEU | human | M | 27 | 0 | no | no | no | other | 1.957 | -0.869 | 1.298 | 0.944 | 8.5 | rural |
| I-0013-MAE | MDG43404010 | MAEU | human | M | 28 | 0 | no | no | no | other | 1.957 | -0.869 | 1.298 | 0.944 | 8.5 | rural |
| I-0014-MAE | MDG43404010 | MAEU | human | M | 27 | 0 | no | no | no | other | 1.957 | -0.869 | 1.298 | 0.944 | 8.5 | rural |
| I-0004-MAE | MDG43404010 | MAEU | human | M | 37 | 0 | no | no | no | other | 1.957 | -0.869 | 1.298 | 0.944 | 8.5 | rural |
| I-0053-MAE | MDG43404031 | MAER | human | F | 20 | 0 | no | no | no | farmer | 1.678 | -1.006 | 0.926 | 0.527 | 8 | rural |
| I-0048-MAE | MDG43404031 | MAER | human | M | 24 | 1 | yes | no | no | farmer | 1.678 | -1.006 | 0.926 | 0.527 | 8 | rural |
| I-0049-MAE | MDG43404031 | MAER | human | F | 24 | 0 | no | no | no | farmer | 1.678 | -1.006 | 0.926 | 0.527 | 8 | rural |
| I-0050-MAE | MDG43404031 | MAER | human | F | 56 | 0 | yes | no | no | farmer | 1.678 | -1.006 | 0.926 | 0.527 | 8 | rural |
| I-0051-MAE | MDG43404031 | MAER | human | M | 63 | 1 | yes | no | no | farmer | 1.678 | -1.006 | 0.926 | 0.527 | 8 | rural |
| I-0052-MAE | MDG43404031 | MAER | human | M | 25 | 0 | yes | no | no | farmer | 1.678 | -1.006 | 0.926 | 0.527 | 8 | rural |
| I-0054-MAE | MDG43404031 | MAER | human | F | 33 | 0 | no | no | no | farmer | 1.678 | -1.006 | 0.926 | 0.527 | 8 | rural |
| I-0055-MAE | MDG43404031 | MAER | human | F | 34 | 0 | no | no | no | farmer | 1.678 | -1.006 | 0.926 | 0.527 | 8 | rural |
| I-0056-MAE | MDG43404031 | MAER | human | F | 21 | 0 | no | no | no | farmer | 1.678 | -1.006 | 0.926 | 0.527 | 8 | rural |
| I-0057-MAE | MDG43404031 | MAER | human | F | 25 | 0 | no | no | no | contact_env | 1.678 | -1.006 | 0.926 | 0.527 | 8 | rural |
| I-0058-MAE | MDG43404031 | MAER | human | F | 42 | 0 | yes | no | no | farmer | 1.678 | -1.006 | 0.926 | 0.527 | 8 | rural |
| I-0047-MAE | MDG43404031 | MAER | human | M | 28 | 0 | no | no | no | farmer | 1.678 | -1.006 | 0.926 | 0.527 | 8 | rural |
| I-0060-MAE | MDG43404031 | MAER | human | F | 50 | 0 | no | no | no | farmer | 1.678 | -1.006 | 0.926 | 0.527 | 8 | rural |
| I-0044-MAE | MDG43404031 | MAER | human | F | 34 | 0 | no | no | no | contact_env | 1.678 | -1.006 | 0.926 | 0.527 | 8 | rural |
| I-0059-MAE | MDG43404031 | MAER | human | M | 60 | 0 | yes | no | no | farmer | 1.678 | -1.006 | 0.926 | 0.527 | 8 | rural |
| I-0036-MAE | MDG43404031 | MAER | human | M | 38 | 0 | no | no | no | contact_env | 1.678 | -1.006 | 0.926 | 0.527 | 8 | rural |
| I-0046-MAE | MDG43404031 | MAER | human | M | 19 | 0 | yes | no | no | contact_env | 1.678 | -1.006 | 0.926 | 0.527 | 8 | rural |
| I-0031-MAE | MDG43404031 | MAER | human | M | 47 | 0 | yes | no | no | farmer | 1.678 | -1.006 | 0.926 | 0.527 | 8 | rural |
| I-0033-MAE | MDG43404031 | MAER | human | F | 22 | 0 | no | no | no | farmer | 1.678 | -1.006 | 0.926 | 0.527 | 8 | rural |
| I-0035-MAE | MDG43404031 | MAER | human | M | 47 | 0 | no | no | no | farmer | 1.678 | -1.006 | 0.926 | 0.527 | 8 | rural |
| I-0032-MAE | MDG43404031 | MAER | human | F | 80 | 0 | no | no | no | farmer | 1.678 | -1.006 | 0.926 | 0.527 | 8 | rural |
| I-0037-MAE | MDG43404031 | MAER | human | F | 27 | 0 | no | no | no | farmer | 1.678 | -1.006 | 0.926 | 0.527 | 8 | rural |
| I-0038-MAE | MDG43404031 | MAER | human | M | 66 | 1 | yes | no | no | farmer | 1.678 | -1.006 | 0.926 | 0.527 | 8 | rural |
| I-0039-MAE | MDG43404031 | MAER | human | M | 62 | 0 | yes | no | no | farmer | 1.678 | -1.006 | 0.926 | 0.527 | 8 | rural |
| I-0040-MAE | MDG43404031 | MAER | human | M | 38 | 0 | no | no | no | farmer | 1.678 | -1.006 | 0.926 | 0.527 | 8 | rural |
| I-0041-MAE | MDG43404031 | MAER | human | F | 39 | 0 | no | no | no | farmer | 1.678 | -1.006 | 0.926 | 0.527 | 8 | rural |
| I-0042-MAE | MDG43404031 | MAER | human | M | 30 | 0 | yes | no | no | contact_env | 1.678 | -1.006 | 0.926 | 0.527 | 8 | rural |
| I-0043-MAE | MDG43404031 | MAER | human | M | 19 | 0 | yes | no | no | contact_env | 1.678 | -1.006 | 0.926 | 0.527 | 8 | rural |
| I-0045-MAE | MDG43404031 | MAER | human | F | 42 | 0 | no | no | no | contact_env | 1.678 | -1.006 | 0.926 | 0.527 | 8 | rural |
| I-0034-MAE | MDG43404031 | MAER | human | M | 31 | 0 | no | no | no | contact_env | 1.678 | -1.006 | 0.926 | 0.527 | 8 | rural |
| I-0029-BOE | MDG41405019 | BOEU | human | M | 22 | 0 | no | no | no | other | 1.461 | -0.749 | 0.557 | 0.770 | 9.42857142857142 | rural |
| I-0018-BOE | MDG41405019 | BOEU | human | F | 27 | 0 | no | no | no | farmer | 1.461 | -0.749 | 0.557 | 0.770 | 9.42857142857142 | rural |
| I-0019-BOE | MDG41405019 | BOEU | human | M | 54 | 0 | no | no | no | other | 1.461 | -0.749 | 0.557 | 0.770 | 9.42857142857142 | rural |
| I-0020-BOE | MDG41405019 | BOEU | human | F | 35 | 0 | no | no | no | farmer | 1.461 | -0.749 | 0.557 | 0.770 | 9.42857142857142 | rural |
| I-0021-BOE | MDG41405019 | BOEU | human | F | 52 | 0 | no | no | no | other | 1.461 | -0.749 | 0.557 | 0.770 | 9.42857142857142 | rural |
| I-0022-BOE | MDG41405019 | BOEU | human | F | 20 | 0 | no | no | no | other | 1.461 | -0.749 | 0.557 | 0.770 | 9.42857142857142 | rural |
| I-0023-BOE | MDG41405019 | BOEU | human | M | 99 | 1 | no | no | no | other | 1.461 | -0.749 | 0.557 | 0.770 | 9.42857142857142 | rural |
| I-0024-BOE | MDG41405019 | BOEU | human | F | 18 | 0 | no | no | no | farmer | 1.461 | -0.749 | 0.557 | 0.770 | 9.42857142857142 | rural |
| I-0025-BOE | MDG41405019 | BOEU | human | M | 44 | 0 | no | no | no | other | 1.461 | -0.749 | 0.557 | 0.770 | 9.42857142857142 | rural |

| ID | PCODE | site2 | sp | gender | age | IgG | contact_ruminant | contact_milk | contact_fresh_fluid | profession | fact1 | fact2 | fact3 | fact4 | cattle_density | habitat |
| --- | --- | --- | --- | --- | --- | --- | --- | --- | --- | --- | --- | --- | --- | --- | --- | --- |
| I-0026-BOE | MDG41405019 | BOEU | human | F | 47 | 0 | no | no | no | farmer | 1.461 | -0.749 | 0.557 | 0.770 | 9.42857142857142 | rural |
| I-0028-BOE | MDG41405019 | BOEU | human | M | 44 | 1 | no | no | no | farmer | 1.461 | -0.749 | 0.557 | 0.770 | 9.42857142857142 | rural |
| I-0030-BOE | MDG41405019 | BOEU | human | M | 18 | 1 | yes | no | no | farmer | 1.461 | -0.749 | 0.557 | 0.770 | 9.42857142857142 | rural |
| I-0017-BOE | MDG41405019 | BOEU | human | M | 20 | 0 | no | no | no | other | 1.461 | -0.749 | 0.557 | 0.770 | 9.42857142857142 | rural |
| I-0014-BOE | MDG41405019 | BOEU | human | M | 39 | 0 | no | no | no | butcher | 1.461 | -0.749 | 0.557 | 0.770 | 9.42857142857142 | rural |
| I-0027-BOE | MDG41405019 | BOEU | human | M | 62 | 0 | no | no | no | farmer | 1.461 | -0.749 | 0.557 | 0.770 | 9.42857142857142 | rural |
| I-0002-BOE | MDG41405019 | BOEU | human | F | 37 | 0 | no | no | no | farmer | 1.461 | -0.749 | 0.557 | 0.770 | 9.42857142857142 | rural |
| I-0016-BOE | MDG41405019 | BOEU | human | M | 53 | 0 | no | no | no | other | 1.461 | -0.749 | 0.557 | 0.770 | 9.42857142857142 | rural |
| I-0001-BOE | MDG41405019 | BOEU | human | M | 45 | 0 | no | no | no | other | 1.461 | -0.749 | 0.557 | 0.770 | 9.42857142857142 | rural |
| I-0003-BOE | MDG41405019 | BOEU | human | M | 46 | 0 | yes | no | no | other | 1.461 | -0.749 | 0.557 | 0.770 | 9.42857142857142 | rural |
| I-0004-BOE | MDG41405019 | BOEU | human | M | 39 | 0 | yes | no | no | farmer | 1.461 | -0.749 | 0.557 | 0.770 | 9.42857142857142 | rural |
| I-0005-BOE | MDG41405019 | BOEU | human | F | 36 | 0 | yes | no | no | other | 1.461 | -0.749 | 0.557 | 0.770 | 9.42857142857142 | rural |
| I-0006-BOE | MDG41405019 | BOEU | human | M | 36 | 1 | no | no | no | farmer | 1.461 | -0.749 | 0.557 | 0.770 | 9.42857142857142 | rural |
| I-0007-BOE | MDG41405019 | BOEU | human | M | 51 | 0 | yes | no | no | farmer | 1.461 | -0.749 | 0.557 | 0.770 | 9.42857142857142 | rural |
| I-0009-BOE | MDG41405019 | BOEU | human | F | 25 | 0 | yes | yes | no | other | 1.461 | -0.749 | 0.557 | 0.770 | 9.42857142857142 | rural |
| I-0010-BOE | MDG41405019 | BOEU | human | F | 37 | 0 | no | no | no | other | 1.461 | -0.749 | 0.557 | 0.770 | 9.42857142857142 | rural |
| I-0011-BOE | MDG41405019 | BOEU | human | F | 30 | 0 | no | no | no | other | 1.461 | -0.749 | 0.557 | 0.770 | 9.42857142857142 | rural |
| I-0012-BOE | MDG41405019 | BOEU | human | M | 46 | 0 | yes | yes | no | farmer | 1.461 | -0.749 | 0.557 | 0.770 | 9.42857142857142 | rural |
| I-0013-BOE | MDG41405019 | BOEU | human | M | 23 | 0 | yes | no | no | other | 1.461 | -0.749 | 0.557 | 0.770 | 9.42857142857142 | rural |
| I-0015-BOE | MDG41405019 | BOEU | human | M | 69 | 0 | no | no | no | farmer | 1.461 | -0.749 | 0.557 | 0.770 | 9.42857142857142 | rural |
| I-0008-BOE | MDG41405019 | BOEU | human | F | 47 | 0 | yes | no | no | farmer | 1.461 | -0.749 | 0.557 | 0.770 | 9.42857142857142 | rural |
| I-0060-BOE | MDG41405051 | BOER | human | M | 26 | 0 | no | no | no | farmer | 1.130 | -0.490 | 0.585 | 0.643 | 9.27272727272727 | rural |
| I-0048-BOE | MDG41405051 | BOER | human | F | 39 | 1 | yes | yes | no | farmer | 1.130 | -0.490 | 0.585 | 0.643 | 9.27272727272727 | rural |
| I-0049-BOE | MDG41405051 | BOER | human | M | 26 | 1 | no | no | no | other | 1.130 | -0.490 | 0.585 | 0.643 | 9.27272727272727 | rural |
| I-0050-BOE | MDG41405051 | BOER | human | M | 21 | 0 | yes | no | no | farmer | 1.130 | -0.490 | 0.585 | 0.643 | 9.27272727272727 | rural |
| I-0051-BOE | MDG41405051 | BOER | human | F | 44 | 0 | yes | no | no | farmer | 1.130 | -0.490 | 0.585 | 0.643 | 9.27272727272727 | rural |
| I-0052-BOE | MDG41405051 | BOER | human | F | 36 | 0 | yes | yes | no | farmer | 1.130 | -0.490 | 0.585 | 0.643 | 9.27272727272727 | rural |
| I-0053-BOE | MDG41405051 | BOER | human | F | 60 | 0 | yes | no | no | farmer | 1.130 | -0.490 | 0.585 | 0.643 | 9.27272727272727 | rural |
| I-0054-BOE | MDG41405051 | BOER | human | F | 22 | 0 | no | no | no | farmer | 1.130 | -0.490 | 0.585 | 0.643 | 9.27272727272727 | rural |
| I-0055-BOE | MDG41405051 | BOER | human | M | 24 | 0 | no | no | no | farmer | 1.130 | -0.490 | 0.585 | 0.643 | 9.27272727272727 | rural |
| I-0059-BOE | MDG41405051 | BOER | human | M | 26 | 0 | yes | no | no | farmer | 1.130 | -0.490 | 0.585 | 0.643 | 9.27272727272727 | rural |
| I-0057-BOE | MDG41405051 | BOER | human | F | 45 | 0 | no | no | no | farmer | 1.130 | -0.490 | 0.585 | 0.643 | 9.27272727272727 | rural |
| I-0058-BOE | MDG41405051 | BOER | human | M | 30 | 0 | yes | no | no | farmer | 1.130 | -0.490 | 0.585 | 0.643 | 9.27272727272727 | rural |
| I-0032-BOE | MDG41405051 | BOER | human | F | 43 | 0 | yes | yes | no | farmer | 1.130 | -0.490 | 0.585 | 0.643 | 9.27272727272727 | rural |
| I-0047-BOE | MDG41405051 | BOER | human | M | 20 | 0 | yes | no | no | farmer | 1.130 | -0.490 | 0.585 | 0.643 | 9.27272727272727 | rural |
| I-0056-BOE | MDG41405051 | BOER | human | F | 22 | 0 | no | no | no | farmer | 1.130 | -0.490 | 0.585 | 0.643 | 9.27272727272727 | rural |
| I-0035-BOE | MDG41405051 | BOER | human | F | 27 | 1 | no | no | no | farmer | 1.130 | -0.490 | 0.585 | 0.643 | 9.27272727272727 | rural |
| I-0034-BOE | MDG41405051 | BOER | human | F | 44 | 1 | no | no | no | farmer | 1.130 | -0.490 | 0.585 | 0.643 | 9.27272727272727 | rural |
| I-0046-BOE | MDG41405051 | BOER | human | M | 30 | 0 | yes | no | no | farmer | 1.130 | -0.490 | 0.585 | 0.643 | 9.27272727272727 | rural |
| I-0033-BOE | MDG41405051 | BOER | human | F | 18 | 0 | no | no | no | farmer | 1.130 | -0.490 | 0.585 | 0.643 | 9.27272727272727 | rural |
| I-0036-BOE | MDG41405051 | BOER | human | F | 38 | 1 | yes | no | no | farmer | 1.130 | -0.490 | 0.585 | 0.643 | 9.27272727272727 | rural |
| I-0037-BOE | MDG41405051 | BOER | human | M | 64 | 1 | no | no | no | farmer | 1.130 | -0.490 | 0.585 | 0.643 | 9.27272727272727 | rural |
| I-0038-BOE | MDG41405051 | BOER | human | M | 44 | 0 | no | no | no | farmer | 1.130 | -0.490 | 0.585 | 0.643 | 9.27272727272727 | rural |
| I-0044-BOE | MDG41405051 | BOER | human | M | 28 | 0 | yes | no | no | farmer | 1.130 | -0.490 | 0.585 | 0.643 | 9.27272727272727 | rural |
| I-0040-BOE | MDG41405051 | BOER | human | M | 43 | 0 | yes | no | no | farmer | 1.130 | -0.490 | 0.585 | 0.643 | 9.27272727272727 | rural |
| I-0041-BOE | MDG41405051 | BOER | human | F | 19 | 0 | no | no | no | other | 1.130 | -0.490 | 0.585 | 0.643 | 9.27272727272727 | rural |
| I-0042-BOE | MDG41405051 | BOER | human | M | 48 | 0 | yes | no | no | farmer | 1.130 | -0.490 | 0.585 | 0.643 | 9.27272727272727 | rural |
| I-0043-BOE | MDG41405051 | BOER | human | F | 35 | 0 | yes | no | no | farmer | 1.130 | -0.490 | 0.585 | 0.643 | 9.27272727272727 | rural |
| I-0039-BOE | MDG41405051 | BOER | human | F | 45 | 0 | no | no | no | farmer | 1.130 | -0.490 | 0.585 | 0.643 | 9.27272727272727 | rural |
| I-0031-BOE | MDG41405051 | BOER | human | M | 42 | 0 | no | no | no | farmer | 1.130 | -0.490 | 0.585 | 0.643 | 9.27272727272727 | rural |
| I-0045-BOE | MDG41405051 | BOER | human | M | 35 | 0 | yes | no | no | farmer | 1.130 | -0.490 | 0.585 | 0.643 | 9.27272727272727 | rural |
| I-0030-MDT | MDG42410010 | MDTU | human | F | 55 | 0 | no | no | no | other | 1.086 | -0.076 | 0.297 | 0.127 | 38 | rural |
| I-0024-MDT | MDG42410010 | MDTU | human | F | 24 | 0 | no | no | no | other | 1.086 | -0.076 | 0.297 | 0.127 | 38 | rural |
| I-0017-MDT | MDG42410010 | MDTU | human | F | 31 | 0 | no | no | no | other | 1.086 | -0.076 | 0.297 | 0.127 | 38 | rural |
| I-0019-MDT | MDG42410010 | MDTU | human | M | 47 | 0 | yes | no | no | other | 1.086 | -0.076 | 0.297 | 0.127 | 38 | rural |
| I-0021-MDT | MDG42410010 | MDTU | human | F | 46 | 0 | no | no | no | other | 1.086 | -0.076 | 0.297 | 0.127 | 38 | rural |
| I-0022-MDT | MDG42410010 | MDTU | human | M | 43 | 0 | no | no | no | other | 1.086 | -0.076 | 0.297 | 0.127 | 38 | rural |
| I-0023-MDT | MDG42410010 | MDTU | human | F | 33 | 0 | no | no | no | other | 1.086 | -0.076 | 0.297 | 0.127 | 38 | rural |
| I-0025-MDT | MDG42410010 | MDTU | human | F | 83 | 0 | no | no | no | other | 1.086 | -0.076 | 0.297 | 0.127 | 38 | rural |
| I-0026-MDT | MDG42410010 | MDTU | human | M | 57 | 0 | no | no | no | other | 1.086 | -0.076 | 0.297 | 0.127 | 38 | rural |
| I-0027-MDT | MDG42410010 | MDTU | human | F | 52 | 0 | no | no | no | other | 1.086 | -0.076 | 0.297 | 0.127 | 38 | rural |
| I-0016-MDT | MDG42410010 | MDTU | human | M | 18 | 0 | no | no | no | other | 1.086 | -0.076 | 0.297 | 0.127 | 38 | rural |
| I-0029-MDT | MDG42410010 | MDTU | human | F | 21 | 0 | no | no | no | other | 1.086 | -0.076 | 0.297 | 0.127 | 38 | rural |

| ID | PCODE | site2 | sp | gender | age | IgG | contact_ruminant | contact_milk | contact_fresh_fluid | profession | fact1 | fact2 | fact3 | fact4 | cattle_density | habitat |
| --- | --- | --- | --- | --- | --- | --- | --- | --- | --- | --- | --- | --- | --- | --- | --- | --- |
| I-0018-MDT | MDG42410010 | MDTU | human | F | 32 | 0 | no | no | no | other | 1.086 | -0.076 | 0.297 | 0.127 | 38 | rural |
| I-0028-MDT | MDG42410010 | MDTU | human | F | 26 | 0 | no | no | no | other | 1.086 | -0.076 | 0.297 | 0.127 | 38 | rural |
| I-0002-MDT | MDG42410010 | MDTU | human | F | 18 | 0 | no | no | no | other | 1.086 | -0.076 | 0.297 | 0.127 | 38 | rural |
| I-0020-MDT | MDG42410010 | MDTU | human | M | 37 | 0 | no | no | no | other | 1.086 | -0.076 | 0.297 | 0.127 | 38 | rural |
| I-0001-MDT | MDG42410010 | MDTU | human | M | 22 | 0 | yes | no | no | other | 1.086 | -0.076 | 0.297 | 0.127 | 38 | rural |
| I-0015-MDT | MDG42410010 | MDTU | human | F | 23 | 0 | no | no | no | other | 1.086 | -0.076 | 0.297 | 0.127 | 38 | rural |
| I-0003-MDT | MDG42410010 | MDTU | human | F | 47 | 1 | no | no | no | farmer | 1.086 | -0.076 | 0.297 | 0.127 | 38 | rural |
| I-0004-MDT | MDG42410010 | MDTU | human | F | 19 | 1 | no | no | no | other | 1.086 | -0.076 | 0.297 | 0.127 | 38 | rural |
| I-0005-MDT | MDG42410010 | MDTU | human | F | 19 | 0 | no | no | no | other | 1.086 | -0.076 | 0.297 | 0.127 | 38 | rural |
| I-0006-MDT | MDG42410010 | MDTU | human | F | 23 | 0 | no | no | no | other | 1.086 | -0.076 | 0.297 | 0.127 | 38 | rural |
| I-0007-MDT | MDG42410010 | MDTU | human | M | 40 | 0 | no | no | no | other | 1.086 | -0.076 | 0.297 | 0.127 | 38 | rural |
| I-0009-MDT | MDG42410010 | MDTU | human | M | 19 | 0 | yes | no | no | other | 1.086 | -0.076 | 0.297 | 0.127 | 38 | rural |
| I-0010-MDT | MDG42410010 | MDTU | human | M | 57 | 0 | no | no | no | other | 1.086 | -0.076 | 0.297 | 0.127 | 38 | rural |
| I-0011-MDT | MDG42410010 | MDTU | human | F | 54 | 0 | no | no | no | other | 1.086 | -0.076 | 0.297 | 0.127 | 38 | rural |
| I-0012-MDT | MDG42410010 | MDTU | human | M | 33 | 0 | no | no | no | other | 1.086 | -0.076 | 0.297 | 0.127 | 38 | rural |
| I-0013-MDT | MDG42410010 | MDTU | human | F | 29 | 0 | no | no | no | other | 1.086 | -0.076 | 0.297 | 0.127 | 38 | rural |
| I-0014-MDT | MDG42410010 | MDTU | human | M | 45 | 0 | no | no | no | other | 1.086 | -0.076 | 0.297 | 0.127 | 38 | rural |
| I-0008-MDT | MDG42410010 | MDTU | human | M | 21 | 0 | yes | no | no | other | 1.086 | -0.076 | 0.297 | 0.127 | 38 | rural |
| I-0048-MDT | MDG42410050 | MDTR | human | M | 46 | 0 | yes | no | no | farmer | 0.120 | 0.423 | 0.228 | 0.014 | 32.875 | rural |
| I-0050-MDT | MDG42410050 | MDTR | human | F | 53 | 0 | no | no | no | farmer | 0.120 | 0.423 | 0.228 | 0.014 | 32.875 | rural |
| I-0052-MDT | MDG42410050 | MDTR | human | M | 56 | 1 | yes | no | no | farmer | 0.120 | 0.423 | 0.228 | 0.014 | 32.875 | rural |
| I-0053-MDT | MDG42410050 | MDTR | human | M | 18 | 0 | yes | no | no | farmer | 0.120 | 0.423 | 0.228 | 0.014 | 32.875 | rural |
| I-0054-MDT | MDG42410050 | MDTR | human | M | 46 | 0 | yes | no | no | farmer | 0.120 | 0.423 | 0.228 | 0.014 | 32.875 | rural |
| I-0055-MDT | MDG42410050 | MDTR | human | F | 46 | 0 | no | no | no | farmer | 0.120 | 0.423 | 0.228 | 0.014 | 32.875 | rural |
| I-0056-MDT | MDG42410050 | MDTR | human | F | 28 | 0 | no | no | no | farmer | 0.120 | 0.423 | 0.228 | 0.014 | 32.875 | rural |
| I-0057-MDT | MDG42410050 | MDTR | human | F | 31 | 0 | no | no | no | farmer | 0.120 | 0.423 | 0.228 | 0.014 | 32.875 | rural |
| I-0058-MDT | MDG42410050 | MDTR | human | F | 42 | 0 | no | no | no | farmer | 0.120 | 0.423 | 0.228 | 0.014 | 32.875 | rural |
| I-0060-MDT | MDG42410050 | MDTR | human | F | 20 | 0 | yes | no | no | farmer | 0.120 | 0.423 | 0.228 | 0.014 | 32.875 | rural |
| I-0049-MDT | MDG42410050 | MDTR | human | M | 63 | 0 | yes | no | no | other | 0.120 | 0.423 | 0.228 | 0.014 | 32.875 | rural |
| I-0047-MDT | MDG42410050 | MDTR | human | F | 53 | 0 | no | no | no | farmer | 0.120 | 0.423 | 0.228 | 0.014 | 32.875 | rural |
| I-0059-MDT | MDG42410050 | MDTR | human | F | 37 | 0 | no | no | no | farmer | 0.120 | 0.423 | 0.228 | 0.014 | 32.875 | rural |
| I-0034-MDT | MDG42410050 | MDTR | human | F | 51 | 1 | no | no | no | farmer | 0.120 | 0.423 | 0.228 | 0.014 | 32.875 | rural |
| I-0051-MDT | MDG42410050 | MDTR | human | M | 24 | 0 | yes | no | no | farmer | 0.120 | 0.423 | 0.228 | 0.014 | 32.875 | rural |
| I-0046-MDT | MDG42410050 | MDTR | human | M | 53 | 0 | yes | no | no | farmer | 0.120 | 0.423 | 0.228 | 0.014 | 32.875 | rural |
| I-0033-MDT | MDG42410050 | MDTR | human | M | 54 | 0 | yes | no | no | farmer | 0.120 | 0.423 | 0.228 | 0.014 | 32.875 | rural |
| I-0035-MDT | MDG42410050 | MDTR | human | F | 31 | 0 | no | no | no | farmer | 0.120 | 0.423 | 0.228 | 0.014 | 32.875 | rural |
| I-0036-MDT | MDG42410050 | MDTR | human | M | 24 | 0 | yes | no | no | farmer | 0.120 | 0.423 | 0.228 | 0.014 | 32.875 | rural |
| I-0037-MDT | MDG42410050 | MDTR | human | F | 19 | 0 | no | no | no | farmer | 0.120 | 0.423 | 0.228 | 0.014 | 32.875 | rural |
| I-0038-MDT | MDG42410050 | MDTR | human | F | 24 | 0 | no | no | no | farmer | 0.120 | 0.423 | 0.228 | 0.014 | 32.875 | rural |
| I-0039-MDT | MDG42410050 | MDTR | human | M | 42 | 0 | yes | no | no | other | 0.120 | 0.423 | 0.228 | 0.014 | 32.875 | rural |
| I-0041-MDT | MDG42410050 | MDTR | human | F | 33 | 1 | no | no | no | farmer | 0.120 | 0.423 | 0.228 | 0.014 | 32.875 | rural |
| I-0042-MDT | MDG42410050 | MDTR | human | M | 46 | 0 | yes | no | no | farmer | 0.120 | 0.423 | 0.228 | 0.014 | 32.875 | rural |
| I-0043-MDT | MDG42410050 | MDTR | human | M | 26 | 0 | yes | no | no | farmer | 0.120 | 0.423 | 0.228 | 0.014 | 32.875 | rural |
| I-0045-MDT | MDG42410050 | MDTR | human | F | 60 | 0 | no | no | no | farmer | 0.120 | 0.423 | 0.228 | 0.014 | 32.875 | rural |
| I-0044-MDT | MDG42410050 | MDTR | human | F | 44 | 0 | yes | no | no | farmer | 0.120 | 0.423 | 0.228 | 0.014 | 32.875 | rural |
| I-0031-MDT | MDG42410050 | MDTR | human | M | 36 | 0 | yes | no | no | other | 0.120 | 0.423 | 0.228 | 0.014 | 32.875 | rural |
| I-0040-MDT | MDG42410050 | MDTR | human | F | 38 | 0 | no | no | no | farmer | 0.120 | 0.423 | 0.228 | 0.014 | 32.875 | rural |
| I-0032-MDT | MDG42410050 | MDTR | human | F | 32 | 0 | no | no | no | farmer | 0.120 | 0.423 | 0.228 | 0.014 | 32.875 | rural |
| I-0026-AHH | MDG42413010 | AHHU | human | F | 41 | 0 | no | no | no | other | 1.575 | -0.683 | 0.752 | 0.818 | 26 | rural |
| I-0017-AHH | MDG42413010 | AHHU | human | M | 44 | 0 | no | no | no | butcher | 1.575 | -0.683 | 0.752 | 0.818 | 26 | rural |
| I-0018-AHH | MDG42413010 | AHHU | human | M | 40 | 1 | no | no | no | contact_env | 1.575 | -0.683 | 0.752 | 0.818 | 26 | rural |
| I-0019-AHH | MDG42413010 | AHHU | human | F | 30 | 0 | no | no | no | other | 1.575 | -0.683 | 0.752 | 0.818 | 26 | rural |
| I-0020-AHH | MDG42413010 | AHHU | human | F | 28 | 0 | no | no | no | other | 1.575 | -0.683 | 0.752 | 0.818 | 26 | rural |
| I-0021-AHH | MDG42413010 | AHHU | human | M | 43 | 1 | yes | no | no | other | 1.575 | -0.683 | 0.752 | 0.818 | 26 | rural |
| I-0022-AHH | MDG42413010 | AHHU | human | F | 30 | 0 | no | no | no | other | 1.575 | -0.683 | 0.752 | 0.818 | 26 | rural |
| I-0023-AHH | MDG42413010 | AHHU | human | F | 20 | 0 | no | no | no | other | 1.575 | -0.683 | 0.752 | 0.818 | 26 | rural |
| I-0025-AHH | MDG42413010 | AHHU | human | M | 40 | 1 | no | no | no | farmer | 1.575 | -0.683 | 0.752 | 0.818 | 26 | rural |
| I-0027-AHH | MDG42413010 | AHHU | human | F | 40 | 1 | no | no | no | other | 1.575 | -0.683 | 0.752 | 0.818 | 26 | rural |
| I-0028-AHH | MDG42413010 | AHHU | human | M | 42 | 0 | no | no | no | butcher | 1.575 | -0.683 | 0.752 | 0.818 | 26 | rural |
| I-0014-AHH | MDG42413010 | AHHU | human | F | 43 | 0 | no | no | no | farmer | 1.575 | -0.683 | 0.752 | 0.818 | 26 | rural |
| I-0029-AHH | MDG42413010 | AHHU | human | F | 39 | 0 | no | no | no | other | 1.575 | -0.683 | 0.752 | 0.818 | 26 | rural |
| I-0024-AHH | MDG42413010 | AHHU | human | M | 24 | 0 | no | no | no | other | 1.575 | -0.683 | 0.752 | 0.818 | 26 | rural |
| I-0004-AHH | MDG42413010 | AHHU | human | F | 60 | 0 | no | no | no | other | 1.575 | -0.683 | 0.752 | 0.818 | 26 | rural |

| ID | PCODE | site2 | sp | gender | age | IgG | contact_ruminant | contact_milk | contact_fresh_fluid | profession | fact1 | fact2 | fact3 | fact4 | cattle_density | habitat |
| --- | --- | --- | --- | --- | --- | --- | --- | --- | --- | --- | --- | --- | --- | --- | --- | --- |
| I-0016-AHH | MDG42413010 | AHHU | human | F | 33 | 0 | no | no | no | other | 1.575 | -0.683 | 0.752 | 0.818 | 26 | rural |
| I-0015-AHH | MDG42413010 | AHHU | human | F | 24 | 0 | no | yes | no | farmer | 1.575 | -0.683 | 0.752 | 0.818 | 26 | rural |
| I-0001-AHH | MDG42413010 | AHHU | human | M | 55 | 0 | no | no | no | other | 1.575 | -0.683 | 0.752 | 0.818 | 26 | rural |
| I-0003-AHH | MDG42413010 | AHHU | human | F | 25 | 1 | no | no | no | other | 1.575 | -0.683 | 0.752 | 0.818 | 26 | rural |
| I-0030-AHH | MDG42413010 | AHHU | human | F | 46 | 0 | no | no | no | other | 1.575 | -0.683 | 0.752 | 0.818 | 26 | rural |
| I-0005-AHH | MDG42413010 | AHHU | human | F | 68 | 0 | no | no | no | other | 1.575 | -0.683 | 0.752 | 0.818 | 26 | rural |
| I-0006-AHH | MDG42413010 | AHHU | human | F | 46 | 0 | no | no | no | other | 1.575 | -0.683 | 0.752 | 0.818 | 26 | rural |
| I-0007-AHH | MDG42413010 | AHHU | human | M | 37 | 0 | no | no | no | other | 1.575 | -0.683 | 0.752 | 0.818 | 26 | rural |
| I-0008-AHH | MDG42413010 | AHHU | human | M | 22 | 0 | no | no | no | other | 1.575 | -0.683 | 0.752 | 0.818 | 26 | rural |
| I-0009-AHH | MDG42413010 | AHHU | human | M | 20 | 0 | no | no | no | other | 1.575 | -0.683 | 0.752 | 0.818 | 26 | rural |
| I-0010-AHH | MDG42413010 | AHHU | human | F | 32 | 0 | no | no | no | other | 1.575 | -0.683 | 0.752 | 0.818 | 26 | rural |
| I-0011-AHH | MDG42413010 | AHHU | human | M | 34 | 0 | yes | no | no | health | 1.575 | -0.683 | 0.752 | 0.818 | 26 | rural |
| I-0012-AHH | MDG42413010 | AHHU | human | F | 29 | 0 | no | no | no | farmer | 1.575 | -0.683 | 0.752 | 0.818 | 26 | rural |
| I-0013-AHH | MDG42413010 | AHHU | human | M | 19 | 0 | no | no | no | other | 1.575 | -0.683 | 0.752 | 0.818 | 26 | rural |
| I-0002-AHH | MDG42413010 | AHHU | human | M | 52 | 0 | no | no | no | other | 1.575 | -0.683 | 0.752 | 0.818 | 26 | rural |
| I-0053-AHH | MDG42413090 | AHHR | human | M | 36 | 0 | no | no | no | farmer | 1.182 | -0.757 | 0.742 | 0.804 | 20.2777777777777 | rural |
| I-0047-AHH | MDG42413090 | AHHR | human | F | 48 | 0 | no | no | no | farmer | 1.182 | -0.757 | 0.742 | 0.804 | 20.2777777777777 | rural |
| I-0048-AHH | MDG42413090 | AHHR | human | F | 47 | 0 | no | no | no | farmer | 1.182 | -0.757 | 0.742 | 0.804 | 20.2777777777777 | rural |
| I-0049-AHH | MDG42413090 | AHHR | human | M | 47 | 0 | yes | no | no | farmer | 1.182 | -0.757 | 0.742 | 0.804 | 20.2777777777777 | rural |
| I-0050-AHH | MDG42413090 | AHHR | human | M | 48 | 0 | yes | no | no | farmer | 1.182 | -0.757 | 0.742 | 0.804 | 20.2777777777777 | rural |
| I-0051-AHH | MDG42413090 | AHHR | human | M | 19 | 0 | no | no | no | other | 1.182 | -0.757 | 0.742 | 0.804 | 20.2777777777777 | rural |
| I-0052-AHH | MDG42413090 | AHHR | human | F | 39 | 1 | no | no | no | farmer | 1.182 | -0.757 | 0.742 | 0.804 | 20.2777777777777 | rural |
| I-0054-AHH | MDG42413090 | AHHR | human | F | 34 | 0 | no | no | no | other | 1.182 | -0.757 | 0.742 | 0.804 | 20.2777777777777 | rural |
| I-0055-AHH | MDG42413090 | AHHR | human | F | 51 | 0 | no | no | no | farmer | 1.182 | -0.757 | 0.742 | 0.804 | 20.2777777777777 | rural |
| I-0056-AHH | MDG42413090 | AHHR | human | M | 55 | 0 | yes | no | no | farmer | 1.182 | -0.757 | 0.742 | 0.804 | 20.2777777777777 | rural |
| I-0057-AHH | MDG42413090 | AHHR | human | M | 29 | 0 | no | no | no | health | 1.182 | -0.757 | 0.742 | 0.804 | 20.2777777777777 | rural |
| I-0058-AHH | MDG42413090 | AHHR | human | M | 20 | 0 | no | no | no | farmer | 1.182 | -0.757 | 0.742 | 0.804 | 20.2777777777777 | rural |
| I-0059-AHH | MDG42413090 | AHHR | human | M | 57 | 0 | no | no | no | contact_env | 1.182 | -0.757 | 0.742 | 0.804 | 20.2777777777777 | rural |
| I-0046-AHH | MDG42413090 | AHHR | human | F | 32 | 0 | no | no | no | other | 1.182 | -0.757 | 0.742 | 0.804 | 20.2777777777777 | rural |
| I-0060-AHH | MDG42413090 | AHHR | human | F | 30 | 0 | no | no | no | farmer | 1.182 | -0.757 | 0.742 | 0.804 | 20.2777777777777 | rural |
| I-0031-AHH | MDG42413090 | AHHR | human | F | 27 | 0 | no | no | no | farmer | 1.182 | -0.757 | 0.742 | 0.804 | 20.2777777777777 | rural |
| I-0045-AHH | MDG42413090 | AHHR | human | F | 50 | 0 | no | no | no | farmer | 1.182 | -0.757 | 0.742 | 0.804 | 20.2777777777777 | rural |
| I-0032-AHH | MDG42413090 | AHHR | human | M | 20 | 0 | yes | no | no | farmer | 1.182 | -0.757 | 0.742 | 0.804 | 20.2777777777777 | rural |
| I-0033-AHH | MDG42413090 | AHHR | human | M | 36 | 0 | yes | no | no | farmer | 1.182 | -0.757 | 0.742 | 0.804 | 20.2777777777777 | rural |
| I-0034-AHH | MDG42413090 | AHHR | human | M | 47 | 0 | yes | no | no | farmer | 1.182 | -0.757 | 0.742 | 0.804 | 20.2777777777777 | rural |
| I-0035-AHH | MDG42413090 | AHHR | human | F | 45 | 0 | no | no | no | farmer | 1.182 | -0.757 | 0.742 | 0.804 | 20.2777777777777 | rural |
| I-0036-AHH | MDG42413090 | AHHR | human | F | 24 | 0 | no | no | no | farmer | 1.182 | -0.757 | 0.742 | 0.804 | 20.2777777777777 | rural |
| I-0037-AHH | MDG42413090 | AHHR | human | F | 26 | 0 | no | no | no | farmer | 1.182 | -0.757 | 0.742 | 0.804 | 20.2777777777777 | rural |
| I-0039-AHH | MDG42413090 | AHHR | human | F | 23 | 0 | no | no | no | farmer | 1.182 | -0.757 | 0.742 | 0.804 | 20.2777777777777 | rural |
| I-0040-AHH | MDG42413090 | AHHR | human | M | 65 | 1 | no | no | no | farmer | 1.182 | -0.757 | 0.742 | 0.804 | 20.2777777777777 | rural |
| I-0041-AHH | MDG42413090 | AHHR | human | M | 29 | 0 | no | no | no | farmer | 1.182 | -0.757 | 0.742 | 0.804 | 20.2777777777777 | rural |
| I-0042-AHH | MDG42413090 | AHHR | human | F | 26 | 1 | no | no | no | other | 1.182 | -0.757 | 0.742 | 0.804 | 20.2777777777777 | rural |
| I-0043-AHH | MDG42413090 | AHHR | human | M | 26 | 1 | yes | no | no | farmer | 1.182 | -0.757 | 0.742 | 0.804 | 20.2777777777777 | rural |
| I-0044-AHH | MDG42413090 | AHHR | human | F | 20 | 0 | no | no | no | farmer | 1.182 | -0.757 | 0.742 | 0.804 | 20.2777777777777 | rural |
| I-0038-AHH | MDG42413090 | AHHR | human | F | 47 | 0 | no | no | no | farmer | 1.182 | -0.757 | 0.742 | 0.804 | 20.2777777777777 | rural |
| I-0012-MJG | MDG41415011 | MJGR | human | M | 39 | 0 | yes | no | no | contact_env | 1.781 | -0.323 | 0.192 | 0.586 | 16.1 | rural |
| I-0013-MJG | MDG41415011 | MJGR | human | F | 34 | 0 | yes | no | no | farmer | 1.781 | -0.323 | 0.192 | 0.586 | 16.1 | rural |
| I-0014-MJG | MDG41415011 | MJGR | human | F | 25 | 0 | yes | no | no | farmer | 1.781 | -0.323 | 0.192 | 0.586 | 16.1 | rural |
| I-0015-MJG | MDG41415011 | MJGR | human | M | 19 | 0 | yes | no | no | contact_env | 1.781 | -0.323 | 0.192 | 0.586 | 16.1 | rural |
| I-0016-MJG | MDG41415011 | MJGR | human | F | 23 | 0 | yes | yes | no | farmer | 1.781 | -0.323 | 0.192 | 0.586 | 16.1 | rural |
| I-0018-MJG | MDG41415011 | MJGR | human | M | 18 | 0 | yes | no | no | farmer | 1.781 | -0.323 | 0.192 | 0.586 | 16.1 | rural |
| I-0019-MJG | MDG41415011 | MJGR | human | M | 19 | 0 | yes | no | no | farmer | 1.781 | -0.323 | 0.192 | 0.586 | 16.1 | rural |
| I-0011-MJG | MDG41415011 | MJGR | human | F | 66 | 0 | yes | no | no | farmer | 1.781 | -0.323 | 0.192 | 0.586 | 16.1 | rural |
| I-0002-MJG | MDG41415011 | MJGR | human | M | 69 | 0 | yes | no | no | farmer | 1.781 | -0.323 | 0.192 | 0.586 | 16.1 | rural |
| I-0017-MJG | MDG41415011 | MJGR | human | M | 28 | 1 | yes | yes | no | farmer | 1.781 | -0.323 | 0.192 | 0.586 | 16.1 | rural |
| I-0010-MJG | MDG41415011 | MJGR | human | F | 29 | 0 | yes | no | no | farmer | 1.781 | -0.323 | 0.192 | 0.586 | 16.1 | rural |
| I-0009-MJG | MDG41415011 | MJGR | human | M | 46 | 0 | yes | no | no | farmer | 1.781 | -0.323 | 0.192 | 0.586 | 16.1 | rural |
| I-0008-MJG | MDG41415011 | MJGR | human | F | 55 | 0 | no | yes | no | farmer | 1.781 | -0.323 | 0.192 | 0.586 | 16.1 | rural |
| I-0007-MJG | MDG41415011 | MJGR | human | M | 63 | 0 | yes | no | no | farmer | 1.781 | -0.323 | 0.192 | 0.586 | 16.1 | rural |
| I-0006-MJG | MDG41415011 | MJGR | human | M | 39 | 1 | yes | no | no | farmer | 1.781 | -0.323 | 0.192 | 0.586 | 16.1 | rural |
| I-0005-MJG | MDG41415011 | MJGR | human | M | 27 | 0 | yes | no | no | farmer | 1.781 | -0.323 | 0.192 | 0.586 | 16.1 | rural |
| I-0003-MJG | MDG41415011 | MJGR | human | F | 33 | 0 | yes | no | no | farmer | 1.781 | -0.323 | 0.192 | 0.586 | 16.1 | rural |
| I-0001-MJG | MDG41415011 | MJGR | human | F | 56 | 0 | yes | no | no | farmer | 1.781 | -0.323 | 0.192 | 0.586 | 16.1 | rural |

| ID | PCODE | site2 | sp | gender | age | IgG | contact_ruminant | contact_milk | contact_fresh_fluid | profession | fact1 | fact2 | fact3 | fact4 | cattle_density | habitat |
| --- | --- | --- | --- | --- | --- | --- | --- | --- | --- | --- | --- | --- | --- | --- | --- | --- |
| I-0022-MJG | MDG41415011 | MJGR | human | F | 30 | 0 | yes | no | no | farmer | 1.781 | -0.323 | 0.192 | 0.586 | 16.1 | rural |
| I-0004-MJG | MDG41415011 | MJGR | human | M | 30 | 1 | yes | no | no | farmer | 1.781 | -0.323 | 0.192 | 0.586 | 16.1 | rural |
| I-0028-MJG | MDG41415011 | MJGR | human | F | 36 | 0 | yes | no | no | farmer | 1.781 | -0.323 | 0.192 | 0.586 | 16.1 | rural |
| I-0020-MJG | MDG41415011 | MJGR | human | M | 48 | 0 | yes | no | no | farmer | 1.781 | -0.323 | 0.192 | 0.586 | 16.1 | rural |
| I-0029-MJG | MDG41415011 | MJGR | human | F | 49 | 0 | no | no | no | farmer | 1.781 | -0.323 | 0.192 | 0.586 | 16.1 | rural |
| I-0027-MJG | MDG41415011 | MJGR | human | M | 19 | 0 | yes | yes | no | farmer | 1.781 | -0.323 | 0.192 | 0.586 | 16.1 | rural |
| I-0026-MJG | MDG41415011 | MJGR | human | F | 20 | 0 | no | no | no | other | 1.781 | -0.323 | 0.192 | 0.586 | 16.1 | rural |
| I-0025-MJG | MDG41415011 | MJGR | human | M | 60 | 0 | no | no | no | farmer | 1.781 | -0.323 | 0.192 | 0.586 | 16.1 | rural |
| I-0024-MJG | MDG41415011 | MJGR | human | M | 34 | 0 | yes | no | no | farmer | 1.781 | -0.323 | 0.192 | 0.586 | 16.1 | rural |
| I-0023-MJG | MDG41415011 | MJGR | human | M | 33 | 0 | yes | no | no | contact_env | 1.781 | -0.323 | 0.192 | 0.586 | 16.1 | rural |
| I-0021-MJG | MDG41415011 | MJGR | human | M | 25 | 1 | yes | no | no | farmer | 1.781 | -0.323 | 0.192 | 0.586 | 16.1 | rural |
| I-0030-MJG | MDG41415011 | MJGR | human | F | 33 | 0 | no | no | no | farmer | 1.781 | -0.323 | 0.192 | 0.586 | 16.1 | rural |
| I-0024-TOL | MDG51501001 | TOLU | human | M | 39 | 0 | yes | no | no | other | 3.290 | -0.284 | -0.996 | 0.113 | 148 | urban |
| I-0019-TOL | MDG51501001 | TOLU | human | F | 33 | 0 | yes | no | no | other | 3.290 | -0.284 | -0.996 | 0.113 | 148 | urban |
| I-0020-TOL | MDG51501001 | TOLU | human | M | 20 | 0 | yes | no | no | other | 3.290 | -0.284 | -0.996 | 0.113 | 148 | urban |
| I-0021-TOL | MDG51501001 | TOLU | human | F | 35 | 0 | no | no | no | other | 3.290 | -0.284 | -0.996 | 0.113 | 148 | urban |
| I-0022-TOL | MDG51501001 | TOLU | human | F | 41 | 0 | no | no | no | other | 3.290 | -0.284 | -0.996 | 0.113 | 148 | urban |
| I-0023-TOL | MDG51501001 | TOLU | human | M | 25 | 0 | no | no | no | other | 3.290 | -0.284 | -0.996 | 0.113 | 148 | urban |
| I-0025-TOL | MDG51501001 | TOLU | human | M | 22 | 0 | yes | no | no | other | 3.290 | -0.284 | -0.996 | 0.113 | 148 | urban |
| I-0026-TOL | MDG51501001 | TOLU | human | F | 24 | 0 | no | no | no | other | 3.290 | -0.284 | -0.996 | 0.113 | 148 | urban |
| I-0027-TOL | MDG51501001 | TOLU | human | M | 48 | 0 | no | no | no | other | 3.290 | -0.284 | -0.996 | 0.113 | 148 | urban |
| I-0028-TOL | MDG51501001 | TOLU | human | M | 57 | 0 | no | no | no | other | 3.290 | -0.284 | -0.996 | 0.113 | 148 | urban |
| I-0029-TOL | MDG51501001 | TOLU | human | F | 30 | 0 | no | no | no | other | 3.290 | -0.284 | -0.996 | 0.113 | 148 | urban |
| I-0030-TOL | MDG51501001 | TOLU | human | F | 32 | 0 | no | no | no | other | 3.290 | -0.284 | -0.996 | 0.113 | 148 | urban |
| I-0017-TOL | MDG51501001 | TOLU | human | F | 24 | 0 | yes | no | no | other | 3.290 | -0.284 | -0.996 | 0.113 | 148 | urban |
| I-0016-TOL | MDG51501001 | TOLU | human | M | 67 | 0 | yes | yes | no | other | 3.290 | -0.284 | -0.996 | 0.113 | 148 | urban |
| I-0002-TOL | MDG51501001 | TOLU | human | F | 33 | 1 | no | no | no | farmer | 3.290 | -0.284 | -0.996 | 0.113 | 148 | urban |
| I-0018-TOL | MDG51501001 | TOLU | human | M | 32 | 0 | yes | no | no | other | 3.290 | -0.284 | -0.996 | 0.113 | 148 | urban |
| I-0003-TOL | MDG51501001 | TOLU | human | F | 41 | 0 | no | no | no | other | 3.290 | -0.284 | -0.996 | 0.113 | 148 | urban |
| I-0004-TOL | MDG51501001 | TOLU | human | F | 32 | 0 | no | no | no | other | 3.290 | -0.284 | -0.996 | 0.113 | 148 | urban |
| I-0005-TOL | MDG51501001 | TOLU | human | M | 50 | 0 | no | no | no | other | 3.290 | -0.284 | -0.996 | 0.113 | 148 | urban |
| I-0006-TOL | MDG51501001 | TOLU | human | F | 31 | 0 | no | no | no | other | 3.290 | -0.284 | -0.996 | 0.113 | 148 | urban |
| I-0007-TOL | MDG51501001 | TOLU | human | F | 40 | 0 | no | no | no | other | 3.290 | -0.284 | -0.996 | 0.113 | 148 | urban |
| I-0008-TOL | MDG51501001 | TOLU | human | M | 29 | 0 | no | no | no | other | 3.290 | -0.284 | -0.996 | 0.113 | 148 | urban |
| I-0001-TOL | MDG51501001 | TOLU | human | M | 32 | 0 | no | no | no | other | 3.290 | -0.284 | -0.996 | 0.113 | 148 | urban |
| I-0010-TOL | MDG51501001 | TOLU | human | F | 38 | 0 | no | no | no | other | 3.290 | -0.284 | -0.996 | 0.113 | 148 | urban |
| I-0011-TOL | MDG51501001 | TOLU | human | M | 24 | 0 | no | no | no | other | 3.290 | -0.284 | -0.996 | 0.113 | 148 | urban |
| I-0012-TOL | MDG51501001 | TOLU | human | M | 43 | 0 | no | no | no | other | 3.290 | -0.284 | -0.996 | 0.113 | 148 | urban |
| I-0013-TOL | MDG51501001 | TOLU | human | M | 62 | 0 | no | no | no | other | 3.290 | -0.284 | -0.996 | 0.113 | 148 | urban |
| I-0015-TOL | MDG51501001 | TOLU | human | M | 55 | 0 | no | no | no | other | 3.290 | -0.284 | -0.996 | 0.113 | 148 | urban |
| I-0014-TOL | MDG51501001 | TOLU | human | F | 32 | 1 | no | no | no | other | 3.290 | -0.284 | -0.996 | 0.113 | 148 | urban |
| I-0009-TOL | MDG51501001 | TOLU | human | M | 28 | 0 | no | no | no | other | 3.290 | -0.284 | -0.996 | 0.113 | 148 | urban |
| I-0045-MRB | MDG51504050 | MRBR | human | F | 36 | 0 | yes | yes | no | farmer | 2.064 | -0.315 | -0.082 | 0.092 | 10.2222222222222 | rural |
| I-0046-MRB | MDG51504050 | MRBR | human | M | 25 | 0 | yes | no | no | farmer | 2.064 | -0.315 | -0.082 | 0.092 | 10.2222222222222 | rural |
| I-0047-MRB | MDG51504050 | MRBR | human | M | 24 | 0 | yes | yes | no | farmer | 2.064 | -0.315 | -0.082 | 0.092 | 10.2222222222222 | rural |
| I-0048-MRB | MDG51504050 | MRBR | human | F | 19 | 1 | yes | yes | no | farmer | 2.064 | -0.315 | -0.082 | 0.092 | 10.2222222222222 | rural |
| I-0044-MRB | MDG51504050 | MRBR | human | F | 24 | 0 | yes | yes | no | farmer | 2.064 | -0.315 | -0.082 | 0.092 | 10.2222222222222 | rural |
| I-0050-MRB | MDG51504050 | MRBR | human | M | 18 | 0 | yes | no | no | farmer | 2.064 | -0.315 | -0.082 | 0.092 | 10.2222222222222 | rural |
| I-0038-MRB | MDG51504050 | MRBR | human | M | 18 | 1 | yes | yes | no | farmer | 2.064 | -0.315 | -0.082 | 0.092 | 10.2222222222222 | rural |
| I-0049-MRB | MDG51504050 | MRBR | human | M | 49 | 0 | yes | yes | no | farmer | 2.064 | -0.315 | -0.082 | 0.092 | 10.2222222222222 | rural |
| I-0043-MRB | MDG51504050 | MRBR | human | M | 42 | 1 | yes | yes | no | farmer | 2.064 | -0.315 | -0.082 | 0.092 | 10.2222222222222 | rural |
| I-0042-MRB | MDG51504050 | MRBR | human | M | 20 | 0 | yes | yes | no | farmer | 2.064 | -0.315 | -0.082 | 0.092 | 10.2222222222222 | rural |
| I-0041-MRB | MDG51504050 | MRBR | human | F | 20 | 0 | yes | no | no | farmer | 2.064 | -0.315 | -0.082 | 0.092 | 10.2222222222222 | rural |
| I-0036-MRB | MDG51504050 | MRBR | human | M | 20 | 0 | yes | yes | no | farmer | 2.064 | -0.315 | -0.082 | 0.092 | 10.2222222222222 | rural |
| I-0039-MRB | MDG51504050 | MRBR | human | M | 18 | 1 | yes | yes | no | farmer | 2.064 | -0.315 | -0.082 | 0.092 | 10.2222222222222 | rural |
| I-0037-MRB | MDG51504050 | MRBR | human | F | 24 | 1 | yes | yes | no | farmer | 2.064 | -0.315 | -0.082 | 0.092 | 10.2222222222222 | rural |
| I-0051-MRB | MDG51504050 | MRBR | human | M | 51 | 1 | no | no | no | farmer | 2.064 | -0.315 | -0.082 | 0.092 | 10.2222222222222 | rural |
| I-0060-MRB | MDG51504050 | MRBR | human | M | 27 | 0 | no | no | no | farmer | 2.064 | -0.315 | -0.082 | 0.092 | 10.2222222222222 | rural |
| I-0040-MRB | MDG51504050 | MRBR | human | M | 30 | 1 | yes | yes | no | farmer | 2.064 | -0.315 | -0.082 | 0.092 | 10.2222222222222 | rural |
| I-0059-MRB | MDG51504050 | MRBR | human | M | 50 | 0 | yes | yes | no | farmer | 2.064 | -0.315 | -0.082 | 0.092 | 10.2222222222222 | rural |
| I-0058-MRB | MDG51504050 | MRBR | human | M | 40 | 1 | yes | no | no | farmer | 2.064 | -0.315 | -0.082 | 0.092 | 10.2222222222222 | rural |
| I-0032-MRB | MDG51504050 | MRBR | human | F | 46 | 0 | no | no | no | other | 2.064 | -0.315 | -0.082 | 0.092 | 10.2222222222222 | rural |
| I-0052-MRB | MDG51504050 | MRBR | human | F | 40 | 0 | yes | no | no | farmer | 2.064 | -0.315 | -0.082 | 0.092 | 10.2222222222222 | rural |

| ID | PCODE | site2 | sp | gender | age | IgG | contact_ruminant | contact_milk | contact_fresh_fluid | profession | fact1 | fact2 | fact3 | fact4 | cattle_density | habitat |
| --- | --- | --- | --- | --- | --- | --- | --- | --- | --- | --- | --- | --- | --- | --- | --- | --- |
| I-0035-MRB | MDG51504050 | MRBR | human | M | 29 | 0 | yes | yes | no | farmer | 2.064 | -0.315 | -0.082 | 0.092 | 10.2222222222222 | rural |
| I-0057-MRB | MDG51504050 | MRBR | human | M | 18 | 0 | yes | no | no | farmer | 2.064 | -0.315 | -0.082 | 0.092 | 10.2222222222222 | rural |
| I-0056-MRB | MDG51504050 | MRBR | human | M | 25 | 1 | yes | yes | no | contact_env | 2.064 | -0.315 | -0.082 | 0.092 | 10.2222222222222 | rural |
| I-0055-MRB | MDG51504050 | MRBR | human | M | 45 | 0 | yes | no | no | farmer | 2.064 | -0.315 | -0.082 | 0.092 | 10.2222222222222 | rural |
| I-0054-MRB | MDG51504050 | MRBR | human | F | 55 | 1 | no | yes | no | farmer | 2.064 | -0.315 | -0.082 | 0.092 | 10.2222222222222 | rural |
| I-0053-MRB | MDG51504050 | MRBR | human | F | 41 | 1 | yes | yes | no | farmer | 2.064 | -0.315 | -0.082 | 0.092 | 10.2222222222222 | rural |
| I-0007-MRB | MDG51504050 | MRBU | human | F | 31 | 0 | yes | no | no | other | 2.064 | -0.315 | -0.082 | 0.092 | 10.2222222222222 | rural |
| I-0016-MRB | MDG51504050 | MRBU | human | F | 57 | 0 | yes | no | no | other | 2.064 | -0.315 | -0.082 | 0.092 | 10.2222222222222 | rural |
| I-0015-MRB | MDG51504050 | MRBU | human | M | 45 | 0 | no | no | no | other | 2.064 | -0.315 | -0.082 | 0.092 | 10.2222222222222 | rural |
| I-0014-MRB | MDG51504050 | MRBU | human | F | 40 | 0 | no | no | no | other | 2.064 | -0.315 | -0.082 | 0.092 | 10.2222222222222 | rural |
| I-0013-MRB | MDG51504050 | MRBU | human | F | 50 | 0 | no | no | no | other | 2.064 | -0.315 | -0.082 | 0.092 | 10.2222222222222 | rural |
| I-0011-MRB | MDG51504050 | MRBU | human | F | 37 | 0 | yes | no | no | farmer | 2.064 | -0.315 | -0.082 | 0.092 | 10.2222222222222 | rural |
| I-0034-MRB | MDG51504050 | MRBR | human | M | 28 | 0 | yes | yes | yes | farmer | 2.064 | -0.315 | -0.082 | 0.092 | 10.2222222222222 | rural |
| I-0017-MRB | MDG51504050 | MRBU | human | M | 31 | 0 | yes | no | no | other | 2.064 | -0.315 | -0.082 | 0.092 | 10.2222222222222 | rural |
| I-0008-MRB | MDG51504050 | MRBU | human | M | 44 | 0 | yes | no | no | other | 2.064 | -0.315 | -0.082 | 0.092 | 10.2222222222222 | rural |
| I-0012-MRB | MDG51504050 | MRBU | human | M | 59 | 0 | yes | no | no | farmer | 2.064 | -0.315 | -0.082 | 0.092 | 10.2222222222222 | rural |
| I-0006-MRB | MDG51504050 | MRBU | human | F | 19 | 0 | no | no | no | other | 2.064 | -0.315 | -0.082 | 0.092 | 10.2222222222222 | rural |
| I-0005-MRB | MDG51504050 | MRBU | human | M | 24 | 0 | yes | no | no | farmer | 2.064 | -0.315 | -0.082 | 0.092 | 10.2222222222222 | rural |
| I-0004-MRB | MDG51504050 | MRBU | human | F | 40 | 0 | no | no | no | farmer | 2.064 | -0.315 | -0.082 | 0.092 | 10.2222222222222 | rural |
| I-0003-MRB | MDG51504050 | MRBU | human | F | 24 | 0 | no | no | no | farmer | 2.064 | -0.315 | -0.082 | 0.092 | 10.2222222222222 | rural |
| I-0002-MRB | MDG51504050 | MRBU | human | M | 50 | 0 | yes | yes | no | farmer | 2.064 | -0.315 | -0.082 | 0.092 | 10.2222222222222 | rural |
| I-0001-MRB | MDG51504050 | MRBU | human | M | 39 | 0 | yes | no | no | farmer | 2.064 | -0.315 | -0.082 | 0.092 | 10.2222222222222 | rural |
| I-0009-MRB | MDG51504050 | MRBU | human | F | 40 | 0 | no | no | no | other | 2.064 | -0.315 | -0.082 | 0.092 | 10.2222222222222 | rural |
| I-0027-MRB | MDG51504050 | MRBU | human | F | 30 | 1 | no | no | no | other | 2.064 | -0.315 | -0.082 | 0.092 | 10.2222222222222 | rural |
| I-0033-MRB | MDG51504050 | MRBR | human | M | 33 | 0 | yes | no | no | farmer | 2.064 | -0.315 | -0.082 | 0.092 | 10.2222222222222 | rural |
| I-0010-MRB | MDG51504050 | MRBU | human | M | 41 | 1 | yes | no | no | other | 2.064 | -0.315 | -0.082 | 0.092 | 10.2222222222222 | rural |
| I-0018-MRB | MDG51504050 | MRBU | human | F | 18 | 0 | no | no | no | other | 2.064 | -0.315 | -0.082 | 0.092 | 10.2222222222222 | rural |
| I-0030-MRB | MDG51504050 | MRBU | human | M | 25 | 0 | no | no | no | other | 2.064 | -0.315 | -0.082 | 0.092 | 10.2222222222222 | rural |
| I-0028-MRB | MDG51504050 | MRBU | human | F | 24 | 0 | no | no | no | other | 2.064 | -0.315 | -0.082 | 0.092 | 10.2222222222222 | rural |
| I-0031-MRB | MDG51504050 | MRBR | human | M | 27 | 1 | no | no | no | farmer | 2.064 | -0.315 | -0.082 | 0.092 | 10.2222222222222 | rural |
| I-0026-MRB | MDG51504050 | MRBU | human | M | 41 | 0 | no | no | no | farmer | 2.064 | -0.315 | -0.082 | 0.092 | 10.2222222222222 | rural |
| I-0025-MRB | MDG51504050 | MRBU | human | F | 52 | 0 | yes | no | no | other | 2.064 | -0.315 | -0.082 | 0.092 | 10.2222222222222 | rural |
| I-0024-MRB | MDG51504050 | MRBU | human | M | 32 | 1 | yes | no | no | farmer | 2.064 | -0.315 | -0.082 | 0.092 | 10.2222222222222 | rural |
| I-0023-MRB | MDG51504050 | MRBU | human | F | 34 | 0 | no | no | no | other | 2.064 | -0.315 | -0.082 | 0.092 | 10.2222222222222 | rural |
| I-0022-MRB | MDG51504050 | MRBU | human | M | 39 | 0 | no | no | no | health | 2.064 | -0.315 | -0.082 | 0.092 | 10.2222222222222 | rural |
| I-0021-MRB | MDG51504050 | MRBU | human | M | 31 | 1 | no | no | no | farmer | 2.064 | -0.315 | -0.082 | 0.092 | 10.2222222222222 | rural |
| I-0020-MRB | MDG51504050 | MRBU | human | M | 33 | 0 | yes | no | no | farmer | 2.064 | -0.315 | -0.082 | 0.092 | 10.2222222222222 | rural |
| I-0019-MRB | MDG51504050 | MRBU | human | M | 52 | 0 | no | no | no | other | 2.064 | -0.315 | -0.082 | 0.092 | 10.2222222222222 | rural |
| I-0029-MRB | MDG51504050 | MRBU | human | F | 35 | 0 | no | no | no | other | 2.064 | -0.315 | -0.082 | 0.092 | 10.2222222222222 | rural |
| I-0017-EJD | MDG51507150 | EJDU | human | M | 62 | 0 | yes | no | no | other | 2.193 | -0.369 | -0.679 | -0.749 | 18.5945945945945 | rural |
| I-0022-EJD | MDG51507150 | EJDU | human | F | 27 | 0 | no | no | no | other | 2.193 | -0.369 | -0.679 | -0.749 | 18.5945945945945 | rural |
| I-0018-EJD | MDG51507150 | EJDU | human | F | 39 | 0 | yes | no | no | other | 2.193 | -0.369 | -0.679 | -0.749 | 18.5945945945945 | rural |
| I-0019-EJD | MDG51507150 | EJDU | human | M | 27 | 0 | yes | no | no | farmer | 2.193 | -0.369 | -0.679 | -0.749 | 18.5945945945945 | rural |
| I-0020-EJD | MDG51507150 | EJDU | human | F | 55 | 0 | no | no | no | other | 2.193 | -0.369 | -0.679 | -0.749 | 18.5945945945945 | rural |
| I-0021-EJD | MDG51507150 | EJDU | human | M | 21 | 0 | no | no | no | other | 2.193 | -0.369 | -0.679 | -0.749 | 18.5945945945945 | rural |
| I-0030-EJD | MDG51507150 | EJDU | human | M | 33 | 0 | yes | no | no | other | 2.193 | -0.369 | -0.679 | -0.749 | 18.5945945945945 | rural |
| I-0024-EJD | MDG51507150 | EJDU | human | M | 24 | 0 | no | no | no | other | 2.193 | -0.369 | -0.679 | -0.749 | 18.5945945945945 | rural |
| I-0026-EJD | MDG51507150 | EJDU | human | M | 54 | 0 | yes | no | no | farmer | 2.193 | -0.369 | -0.679 | -0.749 | 18.5945945945945 | rural |
| I-0027-EJD | MDG51507150 | EJDU | human | F | 49 | 0 | yes | no | no | other | 2.193 | -0.369 | -0.679 | -0.749 | 18.5945945945945 | rural |
| I-0028-EJD | MDG51507150 | EJDU | human | M | 20 | 1 | no | no | no | other | 2.193 | -0.369 | -0.679 | -0.749 | 18.5945945945945 | rural |
| I-0023-EJD | MDG51507150 | EJDU | human | M | 62 | 0 | no | no | no | health | 2.193 | -0.369 | -0.679 | -0.749 | 18.5945945945945 | rural |
| I-0016-EJD | MDG51507150 | EJDU | human | F | 37 | 0 | no | no | no | health | 2.193 | -0.369 | -0.679 | -0.749 | 18.5945945945945 | rural |
| I-0029-EJD | MDG51507150 | EJDU | human | M | 46 | 0 | yes | no | no | health | 2.193 | -0.369 | -0.679 | -0.749 | 18.5945945945945 | rural |
| I-0004-EJD | MDG51507150 | EJDU | human | F | 21 | 0 | no | no | no | other | 2.193 | -0.369 | -0.679 | -0.749 | 18.5945945945945 | rural |
| I-0025-EJD | MDG51507150 | EJDU | human | M | 19 | 0 | yes | no | no | other | 2.193 | -0.369 | -0.679 | -0.749 | 18.5945945945945 | rural |
| I-0003-EJD | MDG51507150 | EJDU | human | M | 38 | 0 | no | no | no | other | 2.193 | -0.369 | -0.679 | -0.749 | 18.5945945945945 | rural |
| I-0015-EJD | MDG51507150 | EJDU | human | M | 42 | 0 | yes | no | no | health | 2.193 | -0.369 | -0.679 | -0.749 | 18.5945945945945 | rural |
| I-0005-EJD | MDG51507150 | EJDU | human | M | 31 | 0 | no | no | no | other | 2.193 | -0.369 | -0.679 | -0.749 | 18.5945945945945 | rural |
| I-0006-EJD | MDG51507150 | EJDU | human | F | 20 | 0 | no | no | no | other | 2.193 | -0.369 | -0.679 | -0.749 | 18.5945945945945 | rural |
| I-0007-EJD | MDG51507150 | EJDU | human | M | 27 | 0 | no | no | no | other | 2.193 | -0.369 | -0.679 | -0.749 | 18.5945945945945 | rural |
| I-0014-EJD | MDG51507150 | EJDU | human | M | 53 | 0 | no | no | no | other | 2.193 | -0.369 | -0.679 | -0.749 | 18.5945945945945 | rural |
| I-0009-EJD | MDG51507150 | EJDU | human | F | 46 | 0 | no | no | no | other | 2.193 | -0.369 | -0.679 | -0.749 | 18.5945945945945 | rural |
| I-0010-EJD | MDG51507150 | EJDU | human | M | 24 | 0 | no | no | no | other | 2.193 | -0.369 | -0.679 | -0.749 | 18.5945945945945 | rural |

| ID | PCODE | site2 | sp | gender | age | IgG | contact_ruminant | contact_milk | contact_fresh_fluid | profession | fact1 | fact2 | fact3 | fact4 | cattle_density | habitat |
| --- | --- | --- | --- | --- | --- | --- | --- | --- | --- | --- | --- | --- | --- | --- | --- | --- |
| I-0011-EJD | MDG51507150 | EJDU | human | M | 26 | 0 | no | no | no | other | 2.193 | -0.369 | -0.679 | -0.749 | 18.5945945945945 | rural |
| I-0012-EJD | MDG51507150 | EJDU | human | F | 46 | 0 | no | no | no | farmer | 2.193 | -0.369 | -0.679 | -0.749 | 18.5945945945945 | rural |
| I-0013-EJD | MDG51507150 | EJDU | human | F | 49 | 0 | no | no | no | other | 2.193 | -0.369 | -0.679 | -0.749 | 18.5945945945945 | rural |
| I-0008-EJD | MDG51507150 | EJDU | human | M | 18 | 0 | no | no | no | other | 2.193 | -0.369 | -0.679 | -0.749 | 18.5945945945945 | rural |
| I-0002-EJD | MDG51507150 | EJDU | human | F | 28 | 0 | no | no | no | farmer | 2.193 | -0.369 | -0.679 | -0.749 | 18.5945945945945 | rural |
| I-0057-EJD | MDG51507170 | EJDR | human | M | 20 | 0 | yes | yes | no | farmer | 2.675 | -0.847 | -0.397 | -0.494 | 20.5555555555555 | rural |
| I-0056-EJD | MDG51507170 | EJDR | human | F | 49 | 0 | no | no | no | other | 2.675 | -0.847 | -0.397 | -0.494 | 20.5555555555555 | rural |
| I-0055-EJD | MDG51507170 | EJDR | human | M | 18 | 0 | yes | no | no | farmer | 2.675 | -0.847 | -0.397 | -0.494 | 20.5555555555555 | rural |
| I-0054-EJD | MDG51507170 | EJDR | human | F | 24 | 0 | no | no | no | other | 2.675 | -0.847 | -0.397 | -0.494 | 20.5555555555555 | rural |
| I-0053-EJD | MDG51507170 | EJDR | human | M | 29 | 0 | no | no | no | other | 2.675 | -0.847 | -0.397 | -0.494 | 20.5555555555555 | rural |
| I-0051-EJD | MDG51507170 | EJDR | human | M | 23 | 0 | yes | no | no | farmer | 2.675 | -0.847 | -0.397 | -0.494 | 20.5555555555555 | rural |
| I-0058-EJD | MDG51507170 | EJDR | human | F | 18 | 0 | yes | no | no | other | 2.675 | -0.847 | -0.397 | -0.494 | 20.5555555555555 | rural |
| I-0052-EJD | MDG51507170 | EJDR | human | F | 20 | 0 | yes | no | no | other | 2.675 | -0.847 | -0.397 | -0.494 | 20.5555555555555 | rural |
| I-0059-EJD | MDG51507170 | EJDR | human | F | 20 | 0 | no | no | no | other | 2.675 | -0.847 | -0.397 | -0.494 | 20.5555555555555 | rural |
| I-0060-EJD | MDG51507170 | EJDR | human | F | 50 | 0 | no | no | no | other | 2.675 | -0.847 | -0.397 | -0.494 | 20.5555555555555 | rural |
| I-0049-EJD | MDG51507170 | EJDR | human | F | 23 | 0 | yes | no | no | other | 2.675 | -0.847 | -0.397 | -0.494 | 20.5555555555555 | rural |
| I-0048-EJD | MDG51507170 | EJDR | human | F | 30 | 0 | yes | yes | no | other | 2.675 | -0.847 | -0.397 | -0.494 | 20.5555555555555 | rural |
| I-0033-EJD | MDG51507170 | EJDR | human | F | 47 | 0 | no | yes | no | other | 2.675 | -0.847 | -0.397 | -0.494 | 20.5555555555555 | rural |
| I-0031-EJD | MDG51507170 | EJDR | human | F | 42 | 0 | yes | no | no | other | 2.675 | -0.847 | -0.397 | -0.494 | 20.5555555555555 | rural |
| I-0050-EJD | MDG51507170 | EJDR | human | M | 21 | 0 | yes | no | no | other | 2.675 | -0.847 | -0.397 | -0.494 | 20.5555555555555 | rural |
| I-0032-EJD | MDG51507170 | EJDR | human | M | 47 | 0 | no | no | no | other | 2.675 | -0.847 | -0.397 | -0.494 | 20.5555555555555 | rural |
| I-0034-EJD | MDG51507170 | EJDR | human | F | 35 | 0 | no | no | no | farmer | 2.675 | -0.847 | -0.397 | -0.494 | 20.5555555555555 | rural |
| I-0035-EJD | MDG51507170 | EJDR | human | M | 43 | 0 | no | no | no | farmer | 2.675 | -0.847 | -0.397 | -0.494 | 20.5555555555555 | rural |
| I-0036-EJD | MDG51507170 | EJDR | human | F | 30 | 0 | no | no | no | other | 2.675 | -0.847 | -0.397 | -0.494 | 20.5555555555555 | rural |
| I-0037-EJD | MDG51507170 | EJDR | human | F | 57 | 0 | no | no | no | farmer | 2.675 | -0.847 | -0.397 | -0.494 | 20.5555555555555 | rural |
| I-0038-EJD | MDG51507170 | EJDR | human | F | 25 | 0 | no | no | no | other | 2.675 | -0.847 | -0.397 | -0.494 | 20.5555555555555 | rural |
| I-0039-EJD | MDG51507170 | EJDR | human | F | 22 | 0 | no | no | no | farmer | 2.675 | -0.847 | -0.397 | -0.494 | 20.5555555555555 | rural |
| I-0046-EJD | MDG51507170 | EJDR | human | F | 29 | 0 | yes | no | no | other | 2.675 | -0.847 | -0.397 | -0.494 | 20.5555555555555 | rural |
| I-0040-EJD | MDG51507170 | EJDR | human | F | 20 | 0 | no | no | no | other | 2.675 | -0.847 | -0.397 | -0.494 | 20.5555555555555 | rural |
| I-0047-EJD | MDG51507170 | EJDR | human | M | 52 | 0 | yes | no | no | other | 2.675 | -0.847 | -0.397 | -0.494 | 20.5555555555555 | rural |
| I-0045-EJD | MDG51507170 | EJDR | human | F | 44 | 0 | no | no | no | other | 2.675 | -0.847 | -0.397 | -0.494 | 20.5555555555555 | rural |
| I-0044-EJD | MDG51507170 | EJDR | human | M | 52 | 0 | yes | no | no | other | 2.675 | -0.847 | -0.397 | -0.494 | 20.5555555555555 | rural |
| I-0043-EJD | MDG51507170 | EJDR | human | M | 25 | 0 | yes | no | no | farmer | 2.675 | -0.847 | -0.397 | -0.494 | 20.5555555555555 | rural |
| I-0042-EJD | MDG51507170 | EJDR | human | M | 40 | 0 | yes | no | no | farmer | 2.675 | -0.847 | -0.397 | -0.494 | 20.5555555555555 | rural |
| I-0041-EJD | MDG51507170 | EJDR | human | F | 26 | 0 | yes | no | no | other | 2.675 | -0.847 | -0.397 | -0.494 | 20.5555555555555 | rural |
| I-0026-MDV | MDG54508010 | MDVU | human | F | 38 | 0 | no | no | no | other | 1.637 | 0.354 | -0.421 | 0.066 | 18.6666666666666 | rural |
| I-0016-MDV | MDG54508010 | MDVU | human | M | 46 | 0 | no | no | no | other | 1.637 | 0.354 | -0.421 | 0.066 | 18.6666666666666 | rural |
| I-0018-MDV | MDG54508010 | MDVU | human | M | 47 | 0 | no | no | no | butcher | 1.637 | 0.354 | -0.421 | 0.066 | 18.6666666666666 | rural |
| I-0019-MDV | MDG54508010 | MDVU | human | F | 38 | 0 | no | no | no | other | 1.637 | 0.354 | -0.421 | 0.066 | 18.6666666666666 | rural |
| I-0020-MDV | MDG54508010 | MDVU | human | F | 44 | 0 | no | no | no | other | 1.637 | 0.354 | -0.421 | 0.066 | 18.6666666666666 | rural |
| I-0021-MDV | MDG54508010 | MDVU | human | M | 24 | 0 | no | no | no | other | 1.637 | 0.354 | -0.421 | 0.066 | 18.6666666666666 | rural |
| I-0022-MDV | MDG54508010 | MDVU | human | F | 20 | 0 | no | no | no | other | 1.637 | 0.354 | -0.421 | 0.066 | 18.6666666666666 | rural |
| I-0023-MDV | MDG54508010 | MDVU | human | M | 31 | 0 | no | no | no | other | 1.637 | 0.354 | -0.421 | 0.066 | 18.6666666666666 | rural |
| I-0028-MDV | MDG54508010 | MDVU | human | F | 37 | 0 | no | no | no | other | 1.637 | 0.354 | -0.421 | 0.066 | 18.6666666666666 | rural |
| I-0025-MDV | MDG54508010 | MDVU | human | F | 30 | 0 | no | no | no | other | 1.637 | 0.354 | -0.421 | 0.066 | 18.6666666666666 | rural |
| I-0027-MDV | MDG54508010 | MDVU | human | F | 44 | 0 | no | no | no | other | 1.637 | 0.354 | -0.421 | 0.066 | 18.6666666666666 | rural |
| I-0015-MDV | MDG54508010 | MDVU | human | F | 22 | 0 | no | no | no | other | 1.637 | 0.354 | -0.421 | 0.066 | 18.6666666666666 | rural |
| I-0029-MDV | MDG54508010 | MDVU | human | M | 47 | 0 | no | no | no | health | 1.637 | 0.354 | -0.421 | 0.066 | 18.6666666666666 | rural |
| I-0024-MDV | MDG54508010 | MDVU | human | M | 54 | 0 | no | no | no | other | 1.637 | 0.354 | -0.421 | 0.066 | 18.6666666666666 | rural |
| I-0004-MDV | MDG54508010 | MDVU | human | M | 20 | 0 | no | no | no | other | 1.637 | 0.354 | -0.421 | 0.066 | 18.6666666666666 | rural |
| I-0017-MDV | MDG54508010 | MDVU | human | F | 47 | 0 | no | no | no | other | 1.637 | 0.354 | -0.421 | 0.066 | 18.6666666666666 | rural |
| I-0014-MDV | MDG54508010 | MDVU | human | M | 32 | 0 | no | no | no | other | 1.637 | 0.354 | -0.421 | 0.066 | 18.6666666666666 | rural |
| I-0001-MDV | MDG54508010 | MDVU | human | F | 34 | 0 | no | no | no | other | 1.637 | 0.354 | -0.421 | 0.066 | 18.6666666666666 | rural |
| I-0003-MDV | MDG54508010 | MDVU | human | F | 33 | 0 | no | no | no | other | 1.637 | 0.354 | -0.421 | 0.066 | 18.6666666666666 | rural |
| I-0030-MDV | MDG54508010 | MDVU | human | M | 27 | 0 | no | no | no | other | 1.637 | 0.354 | -0.421 | 0.066 | 18.6666666666666 | rural |
| I-0005-MDV | MDG54508010 | MDVU | human | F | 53 | 0 | no | no | no | other | 1.637 | 0.354 | -0.421 | 0.066 | 18.6666666666666 | rural |
| I-0006-MDV | MDG54508010 | MDVU | human | F | 18 | 0 | no | no | no | other | 1.637 | 0.354 | -0.421 | 0.066 | 18.6666666666666 | rural |
| I-0007-MDV | MDG54508010 | MDVU | human | M | 25 | 0 | no | no | no | other | 1.637 | 0.354 | -0.421 | 0.066 | 18.6666666666666 | rural |
| I-0008-MDV | MDG54508010 | MDVU | human | M | 33 | 0 | no | no | no | other | 1.637 | 0.354 | -0.421 | 0.066 | 18.6666666666666 | rural |
| I-0009-MDV | MDG54508010 | MDVU | human | M | 20 | 0 | no | no | no | other | 1.637 | 0.354 | -0.421 | 0.066 | 18.6666666666666 | rural |
| I-0010-MDV | MDG54508010 | MDVU | human | F | 26 | 0 | no | no | no | other | 1.637 | 0.354 | -0.421 | 0.066 | 18.6666666666666 | rural |
| I-0011-MDV | MDG54508010 | MDVU | human | F | 21 | 0 | no | no | no | other | 1.637 | 0.354 | -0.421 | 0.066 | 18.6666666666666 | rural |
| I-0012-MDV | MDG54508010 | MDVU | human | F | 47 | 0 | no | no | no | health | 1.637 | 0.354 | -0.421 | 0.066 | 18.6666666666666 | rural |

| ID | PCODE | site2 | sp | gender | age | IgG | contact_ruminant | contact_milk | contact_fresh_fluid | profession | fact1 | fact2 | fact3 | fact4 | cattle_density | habitat |
| --- | --- | --- | --- | --- | --- | --- | --- | --- | --- | --- | --- | --- | --- | --- | --- | --- |
| I-0013-MDV | MDG54508010 | MDVU | human | M | 22 | 0 | no | no | no | other | 1.637 | 0.354 | -0.421 | 0.066 | 18.6666666666666 | rural |
| I-0002-MDV | MDG54508010 | MDVU | human | M | 35 | 0 | no | no | no | contact_env | 1.637 | 0.354 | -0.421 | 0.066 | 18.6666666666666 | rural |
| I-0053-MDV | MDG54508059 | MDVR | human | F | 19 | 0 | yes | no | no | other | 1.114 | 0.239 | 0.484 | -0.100 | 9.06976744186046 | rural |
| I-0047-MDV | MDG54508059 | MDVR | human | M | 44 | 0 | no | no | no | farmer | 1.114 | 0.239 | 0.484 | -0.100 | 9.06976744186046 | rural |
| I-0048-MDV | MDG54508059 | MDVR | human | M | 25 | 0 | yes | yes | no | farmer | 1.114 | 0.239 | 0.484 | -0.100 | 9.06976744186046 | rural |
| I-0049-MDV | MDG54508059 | MDVR | human | F | 30 | 0 | no | no | no | farmer | 1.114 | 0.239 | 0.484 | -0.100 | 9.06976744186046 | rural |
| I-0050-MDV | MDG54508059 | MDVR | human | M | 18 | 0 | yes | no | no | farmer | 1.114 | 0.239 | 0.484 | -0.100 | 9.06976744186046 | rural |
| I-0051-MDV | MDG54508059 | MDVR | human | M | 20 | 0 | yes | no | no | farmer | 1.114 | 0.239 | 0.484 | -0.100 | 9.06976744186046 | rural |
| I-0052-MDV | MDG54508059 | MDVR | human | M | 20 | 1 | yes | no | no | farmer | 1.114 | 0.239 | 0.484 | -0.100 | 9.06976744186046 | rural |
| I-0054-MDV | MDG54508059 | MDVR | human | F | 51 | 0 | no | no | no | farmer | 1.114 | 0.239 | 0.484 | -0.100 | 9.06976744186046 | rural |
| I-0055-MDV | MDG54508059 | MDVR | human | M | 20 | 0 | yes | yes | no | farmer | 1.114 | 0.239 | 0.484 | -0.100 | 9.06976744186046 | rural |
| I-0056-MDV | MDG54508059 | MDVR | human | M | 24 | 0 | no | yes | no | farmer | 1.114 | 0.239 | 0.484 | -0.100 | 9.06976744186046 | rural |
| I-0057-MDV | MDG54508059 | MDVR | human | F | 43 | 1 | no | no | no | farmer | 1.114 | 0.239 | 0.484 | -0.100 | 9.06976744186046 | rural |
| I-0058-MDV | MDG54508059 | MDVR | human | M | 20 | 0 | yes | no | no | farmer | 1.114 | 0.239 | 0.484 | -0.100 | 9.06976744186046 | rural |
| I-0059-MDV | MDG54508059 | MDVR | human | F | 37 | 0 | yes | no | no | other | 1.114 | 0.239 | 0.484 | -0.100 | 9.06976744186046 | rural |
| I-0046-MDV | MDG54508059 | MDVR | human | M | 22 | 0 | yes | no | no | farmer | 1.114 | 0.239 | 0.484 | -0.100 | 9.06976744186046 | rural |
| I-0060-MDV | MDG54508059 | MDVR | human | F | 29 | 0 | yes | no | no | farmer | 1.114 | 0.239 | 0.484 | -0.100 | 9.06976744186046 | rural |
| I-0036-MDV | MDG54508059 | MDVR | human | F | 56 | 0 | no | yes | no | farmer | 1.114 | 0.239 | 0.484 | -0.100 | 9.06976744186046 | rural |
| I-0045-MDV | MDG54508059 | MDVR | human | M | 62 | 0 | yes | yes | no | farmer | 1.114 | 0.239 | 0.484 | -0.100 | 9.06976744186046 | rural |
| I-0032-MDV | MDG54508059 | MDVR | human | F | 40 | 0 | no | no | no | other | 1.114 | 0.239 | 0.484 | -0.100 | 9.06976744186046 | rural |
| I-0033-MDV | MDG54508059 | MDVR | human | M | 28 | 1 | yes | no | no | farmer | 1.114 | 0.239 | 0.484 | -0.100 | 9.06976744186046 | rural |
| I-0035-MDV | MDG54508059 | MDVR | human | M | 59 | 1 | yes | yes | no | farmer | 1.114 | 0.239 | 0.484 | -0.100 | 9.06976744186046 | rural |
| I-0031-MDV | MDG54508059 | MDVR | human | M | 50 | 0 | yes | no | no | farmer | 1.114 | 0.239 | 0.484 | -0.100 | 9.06976744186046 | rural |
| I-0037-MDV | MDG54508059 | MDVR | human | F | 81 | 1 | no | no | no | other | 1.114 | 0.239 | 0.484 | -0.100 | 9.06976744186046 | rural |
| I-0038-MDV | MDG54508059 | MDVR | human | M | 70 | 1 | no | no | no | farmer | 1.114 | 0.239 | 0.484 | -0.100 | 9.06976744186046 | rural |
| I-0039-MDV | MDG54508059 | MDVR | human | M | 36 | 1 | yes | no | no | farmer | 1.114 | 0.239 | 0.484 | -0.100 | 9.06976744186046 | rural |
| I-0040-MDV | MDG54508059 | MDVR | human | M | 27 | 1 | yes | no | no | farmer | 1.114 | 0.239 | 0.484 | -0.100 | 9.06976744186046 | rural |
| I-0041-MDV | MDG54508059 | MDVR | human | M | 18 | 0 | yes | no | no | farmer | 1.114 | 0.239 | 0.484 | -0.100 | 9.06976744186046 | rural |
| I-0042-MDV | MDG54508059 | MDVR | human | M | 30 | 1 | no | no | no | farmer | 1.114 | 0.239 | 0.484 | -0.100 | 9.06976744186046 | rural |
| I-0043-MDV | MDG54508059 | MDVR | human | M | 25 | 0 | no | no | no | farmer | 1.114 | 0.239 | 0.484 | -0.100 | 9.06976744186046 | rural |
| I-0044-MDV | MDG54508059 | MDVR | human | M | 59 | 1 | yes | no | no | farmer | 1.114 | 0.239 | 0.484 | -0.100 | 9.06976744186046 | rural |
| I-0034-MDV | MDG54508059 | MDVR | human | F | 30 | 0 | no | no | no | other | 1.114 | 0.239 | 0.484 | -0.100 | 9.06976744186046 | rural |
| I-0038-BEL | MDG54510011 | BELR | human | F | 21 | 0 | no | no | no | farmer | 1.242 | 0.206 | 0.749 | 1.415 | 6.28571428571428 | rural |
| I-0044-BEL | MDG54510011 | BELR | human | F | 28 | 0 | no | no | no | farmer | 1.242 | 0.206 | 0.749 | 1.415 | 6.28571428571428 | rural |
| I-0043-BEL | MDG54510011 | BELR | human | F | 30 | 0 | no | no | no | farmer | 1.242 | 0.206 | 0.749 | 1.415 | 6.28571428571428 | rural |
| I-0042-BEL | MDG54510011 | BELR | human | M | 57 | 0 | no | no | no | farmer | 1.242 | 0.206 | 0.749 | 1.415 | 6.28571428571428 | rural |
| I-0040-BEL | MDG54510011 | BELR | human | F | 32 | 0 | no | no | no | farmer | 1.242 | 0.206 | 0.749 | 1.415 | 6.28571428571428 | rural |
| I-0037-BEL | MDG54510011 | BELR | human | M | 32 | 1 | no | no | no | other | 1.242 | 0.206 | 0.749 | 1.415 | 6.28571428571428 | rural |
| I-0036-BEL | MDG54510011 | BELR | human | M | 26 | 0 | yes | no | no | farmer | 1.242 | 0.206 | 0.749 | 1.415 | 6.28571428571428 | rural |
| I-0035-BEL | MDG54510011 | BELR | human | F | 27 | 0 | no | no | no | farmer | 1.242 | 0.206 | 0.749 | 1.415 | 6.28571428571428 | rural |
| I-0034-BEL | MDG54510011 | BELR | human | M | 40 | 1 | no | no | no | farmer | 1.242 | 0.206 | 0.749 | 1.415 | 6.28571428571428 | rural |
| I-0045-BEL | MDG54510011 | BELR | human | F | 59 | 0 | no | no | no | farmer | 1.242 | 0.206 | 0.749 | 1.415 | 6.28571428571428 | rural |
| I-0032-BEL | MDG54510011 | BELR | human | M | 48 | 0 | yes | no | no | farmer | 1.242 | 0.206 | 0.749 | 1.415 | 6.28571428571428 | rural |
| I-0053-BEL | MDG54510011 | BELR | human | F | 24 | 1 | no | no | no | farmer | 1.242 | 0.206 | 0.749 | 1.415 | 6.28571428571428 | rural |
| I-0033-BEL | MDG54510011 | BELR | human | M | 18 | 0 | yes | no | no | farmer | 1.242 | 0.206 | 0.749 | 1.415 | 6.28571428571428 | rural |
| I-0039-BEL | MDG54510011 | BELR | human | F | 20 | 0 | no | no | no | farmer | 1.242 | 0.206 | 0.749 | 1.415 | 6.28571428571428 | rural |
| I-0060-BEL | MDG54510011 | BELR | human | M | 19 | 0 | yes | no | no | other | 1.242 | 0.206 | 0.749 | 1.415 | 6.28571428571428 | rural |
| I-0059-BEL | MDG54510011 | BELR | human | M | 52 | 0 | no | no | no | farmer | 1.242 | 0.206 | 0.749 | 1.415 | 6.28571428571428 | rural |
| I-0058-BEL | MDG54510011 | BELR | human | F | 26 | 1 | no | no | no | farmer | 1.242 | 0.206 | 0.749 | 1.415 | 6.28571428571428 | rural |
| I-0057-BEL | MDG54510011 | BELR | human | F | 22 | 0 | no | no | no | farmer | 1.242 | 0.206 | 0.749 | 1.415 | 6.28571428571428 | rural |
| I-0056-BEL | MDG54510011 | BELR | human | M | 30 | 0 | no | no | no | farmer | 1.242 | 0.206 | 0.749 | 1.415 | 6.28571428571428 | rural |
| I-0051-BEL | MDG54510011 | BELR | human | M | 32 | 0 | no | no | no | farmer | 1.242 | 0.206 | 0.749 | 1.415 | 6.28571428571428 | rural |
| I-0054-BEL | MDG54510011 | BELR | human | F | 34 | 0 | no | no | no | farmer | 1.242 | 0.206 | 0.749 | 1.415 | 6.28571428571428 | rural |
| I-0046-BEL | MDG54510011 | BELR | human | M | 61 | 1 | yes | yes | no | farmer | 1.242 | 0.206 | 0.749 | 1.415 | 6.28571428571428 | rural |
| I-0052-BEL | MDG54510011 | BELR | human | F | 38 | 0 | yes | no | no | farmer | 1.242 | 0.206 | 0.749 | 1.415 | 6.28571428571428 | rural |
| I-0031-BEL | MDG54510011 | BELR | human | F | 41 | 0 | yes | no | no | farmer | 1.242 | 0.206 | 0.749 | 1.415 | 6.28571428571428 | rural |
| I-0050-BEL | MDG54510011 | BELR | human | M | 22 | 0 | yes | no | no | farmer | 1.242 | 0.206 | 0.749 | 1.415 | 6.28571428571428 | rural |
| I-0049-BEL | MDG54510011 | BELR | human | M | 47 | 1 | yes | no | no | farmer | 1.242 | 0.206 | 0.749 | 1.415 | 6.28571428571428 | rural |
| I-0048-BEL | MDG54510011 | BELR | human | F | 20 | 0 | yes | no | no | farmer | 1.242 | 0.206 | 0.749 | 1.415 | 6.28571428571428 | rural |
| I-0047-BEL | MDG54510011 | BELR | human | F | 27 | 0 | no | no | no | farmer | 1.242 | 0.206 | 0.749 | 1.415 | 6.28571428571428 | rural |
| I-0055-BEL | MDG54510011 | BELR | human | M | 38 | 0 | no | no | no | farmer | 1.242 | 0.206 | 0.749 | 1.415 | 6.28571428571428 | rural |
| I-0008-BEL | MDG54510011 | BELU | human | M | 42 | 0 | no | no | no | other | 1.242 | 0.206 | 0.749 | 1.415 | 6.28571428571428 | rural |
| I-0041-BEL | MDG54510011 | BELR | human | F | 43 | 1 | yes | no | no | farmer | 1.242 | 0.206 | 0.749 | 1.415 | 6.28571428571428 | rural |

| ID | PCODE | site2 | sp | gender | age | IgG | contact_ruminant | contact_milk | contact_fresh_fluid | profession | fact1 | fact2 | fact3 | fact4 | cattle_density | habitat |
| --- | --- | --- | --- | --- | --- | --- | --- | --- | --- | --- | --- | --- | --- | --- | --- | --- |
| I-0030-BEL | MDG54510011 | BELU | human | M | 40 | 1 | no | no | no | farmer | 1.242 | 0.206 | 0.749 | 1.415 | 6.28571428571428 | rural |
| I-0001-BEL | MDG54510011 | BELU | human | M | 18 | 0 | no | no | no | other | 1.242 | 0.206 | 0.749 | 1.415 | 6.28571428571428 | rural |
| I-0002-BEL | MDG54510011 | BELU | human | F | 35 | 0 | no | no | no | other | 1.242 | 0.206 | 0.749 | 1.415 | 6.28571428571428 | rural |
| I-0003-BEL | MDG54510011 | BELU | human | M | 31 | 0 | no | no | no | other | 1.242 | 0.206 | 0.749 | 1.415 | 6.28571428571428 | rural |
| I-0004-BEL | MDG54510011 | BELU | human | M | 23 | 0 | no | no | no | other | 1.242 | 0.206 | 0.749 | 1.415 | 6.28571428571428 | rural |
| I-0005-BEL | MDG54510011 | BELU | human | F | 54 | 0 | no | no | no | other | 1.242 | 0.206 | 0.749 | 1.415 | 6.28571428571428 | rural |
| I-0007-BEL | MDG54510011 | BELU | human | F | 34 | 1 | no | no | no | other | 1.242 | 0.206 | 0.749 | 1.415 | 6.28571428571428 | rural |
| I-0009-BEL | MDG54510011 | BELU | human | M | 63 | 0 | no | no | no | other | 1.242 | 0.206 | 0.749 | 1.415 | 6.28571428571428 | rural |
| I-0010-BEL | MDG54510011 | BELU | human | F | 24 | 0 | yes | no | no | other | 1.242 | 0.206 | 0.749 | 1.415 | 6.28571428571428 | rural |
| I-0011-BEL | MDG54510011 | BELU | human | M | 37 | 0 | yes | no | no | farmer | 1.242 | 0.206 | 0.749 | 1.415 | 6.28571428571428 | rural |
| I-0012-BEL | MDG54510011 | BELU | human | F | 37 | 0 | yes | no | no | other | 1.242 | 0.206 | 0.749 | 1.415 | 6.28571428571428 | rural |
| I-0013-BEL | MDG54510011 | BELU | human | M | 29 | 0 | yes | no | no | farmer | 1.242 | 0.206 | 0.749 | 1.415 | 6.28571428571428 | rural |
| I-0014-BEL | MDG54510011 | BELU | human | M | 47 | 0 | yes | no | no | farmer | 1.242 | 0.206 | 0.749 | 1.415 | 6.28571428571428 | rural |
| I-0023-BEL | MDG54510011 | BELU | human | M | 55 | 0 | no | no | no | other | 1.242 | 0.206 | 0.749 | 1.415 | 6.28571428571428 | rural |
| I-0029-BEL | MDG54510011 | BELU | human | F | 62 | 1 | no | no | no | other | 1.242 | 0.206 | 0.749 | 1.415 | 6.28571428571428 | rural |
| I-0006-BEL | MDG54510011 | BELU | human | F | 18 | 0 | no | no | no | other | 1.242 | 0.206 | 0.749 | 1.415 | 6.28571428571428 | rural |
| I-0015-BEL | MDG54510011 | BELU | human | F | 27 | 0 | no | no | no | other | 1.242 | 0.206 | 0.749 | 1.415 | 6.28571428571428 | rural |
| I-0027-BEL | MDG54510011 | BELU | human | M | 22 | 0 | yes | no | no | other | 1.242 | 0.206 | 0.749 | 1.415 | 6.28571428571428 | rural |
| I-0026-BEL | MDG54510011 | BELU | human | M | 57 | 1 | yes | no | no | farmer | 1.242 | 0.206 | 0.749 | 1.415 | 6.28571428571428 | rural |
| I-0024-BEL | MDG54510011 | BELU | human | F | 49 | 0 | no | no | no | other | 1.242 | 0.206 | 0.749 | 1.415 | 6.28571428571428 | rural |
| I-0028-BEL | MDG54510011 | BELU | human | M | 57 | 0 | no | no | no | farmer | 1.242 | 0.206 | 0.749 | 1.415 | 6.28571428571428 | rural |
| I-0022-BEL | MDG54510011 | BELU | human | F | 38 | 0 | no | no | no | other | 1.242 | 0.206 | 0.749 | 1.415 | 6.28571428571428 | rural |
| I-0021-BEL | MDG54510011 | BELU | human | M | 31 | 0 | no | no | no | contact_env | 1.242 | 0.206 | 0.749 | 1.415 | 6.28571428571428 | rural |
| I-0020-BEL | MDG54510011 | BELU | human | F | 51 | 0 | no | no | no | farmer | 1.242 | 0.206 | 0.749 | 1.415 | 6.28571428571428 | rural |
| I-0019-BEL | MDG54510011 | BELU | human | M | 30 | 0 | yes | no | no | farmer | 1.242 | 0.206 | 0.749 | 1.415 | 6.28571428571428 | rural |
| I-0018-BEL | MDG54510011 | BELU | human | F | 48 | 0 | no | no | no | farmer | 1.242 | 0.206 | 0.749 | 1.415 | 6.28571428571428 | rural |
| I-0017-BEL | MDG54510011 | BELU | human | M | 43 | 0 | yes | no | no | farmer | 1.242 | 0.206 | 0.749 | 1.415 | 6.28571428571428 | rural |
| I-0016-BEL | MDG54510011 | BELU | human | M | 29 | 0 | no | yes | no | other | 1.242 | 0.206 | 0.749 | 1.415 | 6.28571428571428 | rural |
| I-0025-BEL | MDG54510011 | BELU | human | M | 20 | 0 | yes | no | no | other | 1.242 | 0.206 | 0.749 | 1.415 | 6.28571428571428 | rural |
| I-0047-MIA | MDG54511011 | MIAR | human | M | 21 | 0 | yes | no | no | farmer | 1.592 | -0.797 | 0.579 | 0.449 | 7.6875 | rural |
| I-0048-MIA | MDG54511011 | MIAR | human | M | 39 | 0 | no | no | no | farmer | 1.592 | -0.797 | 0.579 | 0.449 | 7.6875 | rural |
| I-0042-MIA | MDG54511011 | MIAR | human | M | 29 | 0 | no | no | no | farmer | 1.592 | -0.797 | 0.579 | 0.449 | 7.6875 | rural |
| I-0046-MIA | MDG54511011 | MIAR | human | F | 56 | 0 | yes | yes | no | farmer | 1.592 | -0.797 | 0.579 | 0.449 | 7.6875 | rural |
| I-0045-MIA | MDG54511011 | MIAR | human | F | 67 | 1 | no | no | no | farmer | 1.592 | -0.797 | 0.579 | 0.449 | 7.6875 | rural |
| I-0044-MIA | MDG54511011 | MIAR | human | M | 71 | 0 | no | no | no | farmer | 1.592 | -0.797 | 0.579 | 0.449 | 7.6875 | rural |
| I-0043-MIA | MDG54511011 | MIAR | human | M | 27 | 1 | no | no | no | farmer | 1.592 | -0.797 | 0.579 | 0.449 | 7.6875 | rural |
| I-0041-MIA | MDG54511011 | MIAR | human | F | 30 | 0 | no | no | no | farmer | 1.592 | -0.797 | 0.579 | 0.449 | 7.6875 | rural |
| I-0040-MIA | MDG54511011 | MIAR | human | F | 54 | 0 | no | no | no | other | 1.592 | -0.797 | 0.579 | 0.449 | 7.6875 | rural |
| I-0039-MIA | MDG54511011 | MIAR | human | M | 69 | 0 | no | no | no | farmer | 1.592 | -0.797 | 0.579 | 0.449 | 7.6875 | rural |
| I-0038-MIA | MDG54511011 | MIAR | human | F | 23 | 0 | no | yes | no | farmer | 1.592 | -0.797 | 0.579 | 0.449 | 7.6875 | rural |
| I-0037-MIA | MDG54511011 | MIAR | human | F | 29 | 0 | no | yes | no | farmer | 1.592 | -0.797 | 0.579 | 0.449 | 7.6875 | rural |
| I-0035-MIA | MDG54511011 | MIAR | human | M | 34 | 1 | no | yes | no | farmer | 1.592 | -0.797 | 0.579 | 0.449 | 7.6875 | rural |
| I-0036-MIA | MDG54511011 | MIAR | human | M | 53 | 0 | yes | no | no | farmer | 1.592 | -0.797 | 0.579 | 0.449 | 7.6875 | rural |
| I-0055-MIA | MDG54511011 | MIAR | human | F | 40 | 1 | no | no | no | farmer | 1.592 | -0.797 | 0.579 | 0.449 | 7.6875 | rural |
| I-0034-MIA | MDG54511011 | MIAR | human | M | 31 | 0 | yes | no | no | farmer | 1.592 | -0.797 | 0.579 | 0.449 | 7.6875 | rural |
| I-0060-MIA | MDG54511011 | MIAR | human | F | 28 | 0 | no | no | no | farmer | 1.592 | -0.797 | 0.579 | 0.449 | 7.6875 | rural |
| I-0059-MIA | MDG54511011 | MIAR | human | F | 59 | 0 | no | no | no | farmer | 1.592 | -0.797 | 0.579 | 0.449 | 7.6875 | rural |
| I-0058-MIA | MDG54511011 | MIAR | human | F | 24 | 0 | no | no | no | farmer | 1.592 | -0.797 | 0.579 | 0.449 | 7.6875 | rural |
| I-0056-MIA | MDG54511011 | MIAR | human | F | 47 | 0 | no | no | no | farmer | 1.592 | -0.797 | 0.579 | 0.449 | 7.6875 | rural |
| I-0049-MIA | MDG54511011 | MIAR | human | F | 25 | 0 | no | no | no | farmer | 1.592 | -0.797 | 0.579 | 0.449 | 7.6875 | rural |
| I-0054-MIA | MDG54511011 | MIAR | human | M | 44 | 0 | no | no | no | farmer | 1.592 | -0.797 | 0.579 | 0.449 | 7.6875 | rural |
| I-0053-MIA | MDG54511011 | MIAR | human | M | 30 | 0 | no | no | no | farmer | 1.592 | -0.797 | 0.579 | 0.449 | 7.6875 | rural |
| I-0052-MIA | MDG54511011 | MIAR | human | M | 57 | 0 | no | no | no | farmer | 1.592 | -0.797 | 0.579 | 0.449 | 7.6875 | rural |
| I-0051-MIA | MDG54511011 | MIAR | human | F | 23 | 0 | no | no | no | farmer | 1.592 | -0.797 | 0.579 | 0.449 | 7.6875 | rural |
| I-0050-MIA | MDG54511011 | MIAR | human | F | 43 | 0 | no | no | no | farmer | 1.592 | -0.797 | 0.579 | 0.449 | 7.6875 | rural |
| I-0016-MIA | MDG54511011 | MIAU | human | F | 30 | 0 | no | no | no | farmer | 1.592 | -0.797 | 0.579 | 0.449 | 7.6875 | rural |
| I-0057-MIA | MDG54511011 | MIAR | human | M | 53 | 0 | no | no | no | farmer | 1.592 | -0.797 | 0.579 | 0.449 | 7.6875 | rural |
| I-0006-MIA | MDG54511011 | MIAU | human | M | 44 | 0 | no | no | no | other | 1.592 | -0.797 | 0.579 | 0.449 | 7.6875 | rural |
| I-0013-MIA | MDG54511011 | MIAU | human | F | 29 | 0 | no | no | no | other | 1.592 | -0.797 | 0.579 | 0.449 | 7.6875 | rural |
| I-0012-MIA | MDG54511011 | MIAU | human | F | 25 | 0 | no | no | no | farmer | 1.592 | -0.797 | 0.579 | 0.449 | 7.6875 | rural |
| I-0011-MIA | MDG54511011 | MIAU | human | M | 35 | 0 | no | no | no | farmer | 1.592 | -0.797 | 0.579 | 0.449 | 7.6875 | rural |
| I-0010-MIA | MDG54511011 | MIAU | human | M | 38 | 0 | no | no | no | farmer | 1.592 | -0.797 | 0.579 | 0.449 | 7.6875 | rural |
| I-0009-MIA | MDG54511011 | MIAU | human | F | 39 | 0 | no | no | no | other | 1.592 | -0.797 | 0.579 | 0.449 | 7.6875 | rural |

| ID | PCODE | site2 | sp | gender | age | IgG | contact_ruminant | contact_milk | contact_fresh_fluid | profession | fact1 | fact2 | fact3 | fact4 | cattle_density | habitat |
| --- | --- | --- | --- | --- | --- | --- | --- | --- | --- | --- | --- | --- | --- | --- | --- | --- |
| I-0014-MIA | MDG54511011 | MIAU | human | F | 27 | 0 | no | no | no | farmer | 1.592 | -0.797 | 0.579 | 0.449 | 7.6875 | rural |
| I-0007-MIA | MDG54511011 | MIAU | human | M | 24 | 0 | yes | yes | no | farmer | 1.592 | -0.797 | 0.579 | 0.449 | 7.6875 | rural |
| I-0001-MIA | MDG54511011 | MIAU | human | M | 37 | 0 | no | no | no | other | 1.592 | -0.797 | 0.579 | 0.449 | 7.6875 | rural |
| I-0005-MIA | MDG54511011 | MIAU | human | F | 42 | 0 | no | no | no | other | 1.592 | -0.797 | 0.579 | 0.449 | 7.6875 | rural |
| I-0004-MIA | MDG54511011 | MIAU | human | F | 36 | 0 | yes | no | no | other | 1.592 | -0.797 | 0.579 | 0.449 | 7.6875 | rural |
| I-0003-MIA | MDG54511011 | MIAU | human | M | 36 | 0 | no | no | no | other | 1.592 | -0.797 | 0.579 | 0.449 | 7.6875 | rural |
| I-0002-MIA | MDG54511011 | MIAU | human | F | 29 | 0 | no | no | no | other | 1.592 | -0.797 | 0.579 | 0.449 | 7.6875 | rural |
| I-0033-MIA | MDG54511011 | MIAR | human | F | 28 | 0 | no | no | no | farmer | 1.592 | -0.797 | 0.579 | 0.449 | 7.6875 | rural |
| I-0018-MIA | MDG54511011 | MIAU | human | F | 52 | 0 | no | no | no | farmer | 1.592 | -0.797 | 0.579 | 0.449 | 7.6875 | rural |
| I-0008-MIA | MDG54511011 | MIAU | human | M | 34 | 0 | yes | no | no | farmer | 1.592 | -0.797 | 0.579 | 0.449 | 7.6875 | rural |
| I-0027-MIA | MDG54511011 | MIAU | human | F | 28 | 0 | no | no | no | other | 1.592 | -0.797 | 0.579 | 0.449 | 7.6875 | rural |
| I-0015-MIA | MDG54511011 | MIAU | human | F | 27 | 0 | no | no | no | farmer | 1.592 | -0.797 | 0.579 | 0.449 | 7.6875 | rural |
| I-0030-MIA | MDG54511011 | MIAU | human | F | 80 | 0 | yes | no | no | farmer | 1.592 | -0.797 | 0.579 | 0.449 | 7.6875 | rural |
| I-0032-MIA | MDG54511011 | MIAR | human | F | 20 | 0 | no | no | no | farmer | 1.592 | -0.797 | 0.579 | 0.449 | 7.6875 | rural |
| I-0028-MIA | MDG54511011 | MIAU | human | M | 28 | 0 | no | no | no | other | 1.592 | -0.797 | 0.579 | 0.449 | 7.6875 | rural |
| I-0031-MIA | MDG54511011 | MIAR | human | M | 28 | 1 | no | yes | no | farmer | 1.592 | -0.797 | 0.579 | 0.449 | 7.6875 | rural |
| I-0026-MIA | MDG54511011 | MIAU | human | F | 22 | 0 | no | no | no | other | 1.592 | -0.797 | 0.579 | 0.449 | 7.6875 | rural |
| I-0025-MIA | MDG54511011 | MIAU | human | F | 45 | 0 | no | no | no | other | 1.592 | -0.797 | 0.579 | 0.449 | 7.6875 | rural |
| I-0024-MIA | MDG54511011 | MIAU | human | F | 37 | 0 | no | no | no | other | 1.592 | -0.797 | 0.579 | 0.449 | 7.6875 | rural |
| I-0023-MIA | MDG54511011 | MIAU | human | F | 51 | 0 | no | no | no | other | 1.592 | -0.797 | 0.579 | 0.449 | 7.6875 | rural |
| I-0022-MIA | MDG54511011 | MIAU | human | F | 28 | 0 | no | no | no | farmer | 1.592 | -0.797 | 0.579 | 0.449 | 7.6875 | rural |
| I-0021-MIA | MDG54511011 | MIAU | human | M | 38 | 0 | no | no | no | farmer | 1.592 | -0.797 | 0.579 | 0.449 | 7.6875 | rural |
| I-0020-MIA | MDG54511011 | MIAU | human | M | 33 | 0 | no | no | no | farmer | 1.592 | -0.797 | 0.579 | 0.449 | 7.6875 | rural |
| I-0019-MIA | MDG54511011 | MIAU | human | M | 31 | 0 | no | no | no | other | 1.592 | -0.797 | 0.579 | 0.449 | 7.6875 | rural |
| I-0017-MIA | MDG54511011 | MIAU | human | M | 41 | 0 | no | no | no | farmer | 1.592 | -0.797 | 0.579 | 0.449 | 7.6875 | rural |
| I-0029-MIA | MDG54511011 | MIAU | human | M | 57 | 1 | no | no | no | contact_env | 1.592 | -0.797 | 0.579 | 0.449 | 7.6875 | rural |
| I-0029-TGR | MDG53515010 | TGRU | human | M | 28 | 0 | no | no | no | contact_env | 0.379 | -0.941 | -0.901 | -0.505 | 0.666666666666666 | rural |
| I-0018-TGR | MDG53515010 | TGRU | human | M | 42 | 0 | no | no | no | contact_env | 0.379 | -0.941 | -0.901 | -0.505 | 0.666666666666666 | rural |
| I-0019-TGR | MDG53515010 | TGRU | human | F | 30 | 0 | no | no | no | other | 0.379 | -0.941 | -0.901 | -0.505 | 0.666666666666666 | rural |
| I-0020-TGR | MDG53515010 | TGRU | human | F | 35 | 0 | no | no | no | other | 0.379 | -0.941 | -0.901 | -0.505 | 0.666666666666666 | rural |
| I-0021-TGR | MDG53515010 | TGRU | human | F | 27 | 0 | no | no | no | other | 0.379 | -0.941 | -0.901 | -0.505 | 0.666666666666666 | rural |
| I-0022-TGR | MDG53515010 | TGRU | human | F | 23 | 0 | no | no | no | other | 0.379 | -0.941 | -0.901 | -0.505 | 0.666666666666666 | rural |
| I-0023-TGR | MDG53515010 | TGRU | human | F | 30 | 0 | no | no | no | other | 0.379 | -0.941 | -0.901 | -0.505 | 0.666666666666666 | rural |
| I-0024-TGR | MDG53515010 | TGRU | human | F | 46 | 0 | no | no | no | other | 0.379 | -0.941 | -0.901 | -0.505 | 0.666666666666666 | rural |
| I-0025-TGR | MDG53515010 | TGRU | human | F | 37 | 0 | no | no | no | other | 0.379 | -0.941 | -0.901 | -0.505 | 0.666666666666666 | rural |
| I-0026-TGR | MDG53515010 | TGRU | human | M | 41 | 1 | no | no | no | other | 0.379 | -0.941 | -0.901 | -0.505 | 0.666666666666666 | rural |
| I-0062-TGR | MDG53515010 | TGRU | human | F | 19 | 0 | no | no | no | other | 0.379 | -0.941 | -0.901 | -0.505 | 0.666666666666666 | rural |
| I-0028-TGR | MDG53515010 | TGRU | human | M | 28 | 0 | no | no | no | other | 0.379 | -0.941 | -0.901 | -0.505 | 0.666666666666666 | rural |
| I-0017-TGR | MDG53515010 | TGRU | human | F | 46 | 0 | no | no | no | other | 0.379 | -0.941 | -0.901 | -0.505 | 0.666666666666666 | rural |
| I-0030-TGR | MDG53515010 | TGRU | human | M | 60 | 0 | no | no | no | other | 0.379 | -0.941 | -0.901 | -0.505 | 0.666666666666666 | rural |
| I-0008-TGR | MDG53515010 | TGRU | human | F | 56 | 0 | no | no | no | other | 0.379 | -0.941 | -0.901 | -0.505 | 0.666666666666666 | rural |
| I-0027-TGR | MDG53515010 | TGRU | human | M | 49 | 0 | no | no | no | other | 0.379 | -0.941 | -0.901 | -0.505 | 0.666666666666666 | rural |
| I-0002-TGR | MDG53515010 | TGRU | human | M | 26 | 0 | no | yes | no | other | 0.379 | -0.941 | -0.901 | -0.505 | 0.666666666666666 | rural |
| I-0010-TGR | MDG53515010 | TGRU | human | M | 20 | 0 | no | no | no | other | 0.379 | -0.941 | -0.901 | -0.505 | 0.666666666666666 | rural |
| I-0001-TGR | MDG53515010 | TGRU | human | F | 38 | 0 | no | no | no | other | 0.379 | -0.941 | -0.901 | -0.505 | 0.666666666666666 | rural |
| I-0016-TGR | MDG53515010 | TGRU | human | F | 29 | 0 | no | no | no | other | 0.379 | -0.941 | -0.901 | -0.505 | 0.666666666666666 | rural |
| I-0003-TGR | MDG53515010 | TGRU | human | M | 51 | 0 | no | no | no | other | 0.379 | -0.941 | -0.901 | -0.505 | 0.666666666666666 | rural |
| I-0005-TGR | MDG53515010 | TGRU | human | F | 28 | 0 | no | no | no | other | 0.379 | -0.941 | -0.901 | -0.505 | 0.666666666666666 | rural |
| I-0006-TGR | MDG53515010 | TGRU | human | F | 48 | 0 | no | no | no | other | 0.379 | -0.941 | -0.901 | -0.505 | 0.666666666666666 | rural |
| I-0007-TGR | MDG53515010 | TGRU | human | M | 24 | 0 | no | no | no | other | 0.379 | -0.941 | -0.901 | -0.505 | 0.666666666666666 | rural |
| I-0009-TGR | MDG53515010 | TGRU | human | F | 24 | 0 | no | no | no | other | 0.379 | -0.941 | -0.901 | -0.505 | 0.666666666666666 | rural |
| I-0011-TGR | MDG53515010 | TGRU | human | F | 40 | 0 | no | no | no | other | 0.379 | -0.941 | -0.901 | -0.505 | 0.666666666666666 | rural |
| I-0012-TGR | MDG53515010 | TGRU | human | F | 22 | 0 | no | no | no | other | 0.379 | -0.941 | -0.901 | -0.505 | 0.666666666666666 | rural |
| I-0013-TGR | MDG53515010 | TGRU | human | F | 50 | 0 | no | no | no | other | 0.379 | -0.941 | -0.901 | -0.505 | 0.666666666666666 | rural |
| I-0014-TGR | MDG53515010 | TGRU | human | M | 58 | 0 | no | no | no | other | 0.379 | -0.941 | -0.901 | -0.505 | 0.666666666666666 | rural |
| I-0015-TGR | MDG53515010 | TGRU | human | M | 29 | 0 | no | no | no | other | 0.379 | -0.941 | -0.901 | -0.505 | 0.666666666666666 | rural |
| I-0058-TGR | MDG53515031 | TGRR | human | M | 22 | 1 | no | no | no | farmer | -0.556 | -0.387 | -1.287 | -0.053 | 3.66666666666666 | rural |
| I-0048-TGR | MDG53515031 | TGRR | human | M | 69 | 0 | no | no | no | other | -0.556 | -0.387 | -1.287 | -0.053 | 3.66666666666666 | rural |
| I-0049-TGR | MDG53515031 | TGRR | human | F | 19 | 0 | yes | no | no | other | -0.556 | -0.387 | -1.287 | -0.053 | 3.66666666666666 | rural |
| I-0050-TGR | MDG53515031 | TGRR | human | M | 50 | 1 | no | no | no | butcher | -0.556 | -0.387 | -1.287 | -0.053 | 3.66666666666666 | rural |
| I-0051-TGR | MDG53515031 | TGRR | human | M | 30 | 0 | no | no | no | other | -0.556 | -0.387 | -1.287 | -0.053 | 3.66666666666666 | rural |
| I-0052-TGR | MDG53515031 | TGRR | human | F | 46 | 1 | no | no | no | other | -0.556 | -0.387 | -1.287 | -0.053 | 3.66666666666666 | rural |
| I-0053-TGR | MDG53515031 | TGRR | human | F | 49 | 0 | no | no | no | other | -0.556 | -0.387 | -1.287 | -0.053 | 3.66666666666666 | rural |

| ID | PCODE | site2 | sp | gender | age | IgG | contact_ruminant | contact_milk | contact_fresh_fluid | profession | fact1 | fact2 | fact3 | fact4 | cattle_density | habitat |
| --- | --- | --- | --- | --- | --- | --- | --- | --- | --- | --- | --- | --- | --- | --- | --- | --- |
| I-0054-TGR | MDG53515031 | TGRR | human | M | 42 | 0 | no | no | no | farmer | -0.556 | -0.387 | -1.287 | -0.053 | 3.66666666666666 | rural |
| I-0055-TGR | MDG53515031 | TGRR | human | M | 21 | 0 | no | no | no | farmer | -0.556 | -0.387 | -1.287 | -0.053 | 3.66666666666666 | rural |
| I-0057-TGR | MDG53515031 | TGRR | human | F | 21 | 0 | no | no | no | other | -0.556 | -0.387 | -1.287 | -0.053 | 3.66666666666666 | rural |
| I-0037-TGR | MDG53515031 | TGRR | human | M | 26 | 0 | no | no | no | farmer | -0.556 | -0.387 | -1.287 | -0.053 | 3.66666666666666 | rural |
| I-0059-TGR | MDG53515031 | TGRR | human | F | 43 | 0 | no | no | no | other | -0.556 | -0.387 | -1.287 | -0.053 | 3.66666666666666 | rural |
| I-0060-TGR | MDG53515031 | TGRR | human | F | 43 | 1 | no | no | no | other | -0.556 | -0.387 | -1.287 | -0.053 | 3.66666666666666 | rural |
| I-0047-TGR | MDG53515031 | TGRR | human | F | 60 | 0 | no | no | no | other | -0.556 | -0.387 | -1.287 | -0.053 | 3.66666666666666 | rural |
| I-0056-TGR | MDG53515031 | TGRR | human | F | 39 | 0 | no | no | no | farmer | -0.556 | -0.387 | -1.287 | -0.053 | 3.66666666666666 | rural |
| I-0033-TGR | MDG53515031 | TGRR | human | M | 30 | 0 | no | no | no | other | -0.556 | -0.387 | -1.287 | -0.053 | 3.66666666666666 | rural |
| I-0039-TGR | MDG53515031 | TGRR | human | F | 33 | 0 | no | no | no | other | -0.556 | -0.387 | -1.287 | -0.053 | 3.66666666666666 | rural |
| I-0046-TGR | MDG53515031 | TGRR | human | F | 30 | 0 | no | no | no | other | -0.556 | -0.387 | -1.287 | -0.053 | 3.66666666666666 | rural |
| I-0032-TGR | MDG53515031 | TGRR | human | M | 41 | 0 | no | no | no | contact_env | -0.556 | -0.387 | -1.287 | -0.053 | 3.66666666666666 | rural |
| I-0034-TGR | MDG53515031 | TGRR | human | F | 35 | 0 | no | no | no | other | -0.556 | -0.387 | -1.287 | -0.053 | 3.66666666666666 | rural |
| I-0035-TGR | MDG53515031 | TGRR | human | F | 47 | 0 | yes | no | no | other | -0.556 | -0.387 | -1.287 | -0.053 | 3.66666666666666 | rural |
| I-0036-TGR | MDG53515031 | TGRR | human | F | 22 | 0 | yes | no | no | other | -0.556 | -0.387 | -1.287 | -0.053 | 3.66666666666666 | rural |
| I-0038-TGR | MDG53515031 | TGRR | human | F | 20 | 0 | no | no | no | farmer | -0.556 | -0.387 | -1.287 | -0.053 | 3.66666666666666 | rural |
| I-0040-TGR | MDG53515031 | TGRR | human | M | 62 | 0 | no | no | no | other | -0.556 | -0.387 | -1.287 | -0.053 | 3.66666666666666 | rural |
| I-0041-TGR | MDG53515031 | TGRR | human | F | 33 | 0 | no | no | no | other | -0.556 | -0.387 | -1.287 | -0.053 | 3.66666666666666 | rural |
| I-0042-TGR | MDG53515031 | TGRR | human | M | 43 | 1 | no | no | no | other | -0.556 | -0.387 | -1.287 | -0.053 | 3.66666666666666 | rural |
| I-0045-TGR | MDG53515031 | TGRR | human | F | 20 | 0 | no | no | no | other | -0.556 | -0.387 | -1.287 | -0.053 | 3.66666666666666 | rural |
| I-0043-TGR | MDG53515031 | TGRR | human | F | 40 | 0 | yes | no | no | other | -0.556 | -0.387 | -1.287 | -0.053 | 3.66666666666666 | rural |
| I-0044-TGR | MDG53515031 | TGRR | human | F | 33 | 0 | no | no | no | other | -0.556 | -0.387 | -1.287 | -0.053 | 3.66666666666666 | rural |
| I-0031-TGR | MDG53515031 | TGRR | human | F | 40 | 0 | no | no | no | other | -0.556 | -0.387 | -1.287 | -0.053 | 3.66666666666666 | rural |
| I-0044-ABV | MDG52516151 | ABVR | human | M | 19 | 0 | yes | no | no | farmer | 2.439 | -0.727 | -0.774 | -0.516 | 27.52 | rural |
| I-0033-ABV | MDG52516151 | ABVR | human | M | 63 | 0 | yes | yes | no | farmer | 2.439 | -0.727 | -0.774 | -0.516 | 27.52 | rural |
| I-0034-ABV | MDG52516151 | ABVR | human | M | 23 | 0 | no | yes | no | other | 2.439 | -0.727 | -0.774 | -0.516 | 27.52 | rural |
| I-0035-ABV | MDG52516151 | ABVR | human | M | 33 | 0 | no | no | no | farmer | 2.439 | -0.727 | -0.774 | -0.516 | 27.52 | rural |
| I-0036-ABV | MDG52516151 | ABVR | human | F | 33 | 0 | no | no | no | farmer | 2.439 | -0.727 | -0.774 | -0.516 | 27.52 | rural |
| I-0037-ABV | MDG52516151 | ABVR | human | M | 48 | 0 | yes | no | no | farmer | 2.439 | -0.727 | -0.774 | -0.516 | 27.52 | rural |
| I-0055-ABV | MDG52516151 | ABVR | human | M | 32 | 0 | yes | no | no | farmer | 2.439 | -0.727 | -0.774 | -0.516 | 27.52 | rural |
| I-0038-ABV | MDG52516151 | ABVR | human | F | 18 | 0 | yes | no | no | farmer | 2.439 | -0.727 | -0.774 | -0.516 | 27.52 | rural |
| I-0039-ABV | MDG52516151 | ABVR | human | F | 38 | 0 | no | no | no | farmer | 2.439 | -0.727 | -0.774 | -0.516 | 27.52 | rural |
| I-0040-ABV | MDG52516151 | ABVR | human | F | 33 | 0 | yes | yes | no | farmer | 2.439 | -0.727 | -0.774 | -0.516 | 27.52 | rural |
| I-0041-ABV | MDG52516151 | ABVR | human | M | 45 | 0 | yes | no | no | farmer | 2.439 | -0.727 | -0.774 | -0.516 | 27.52 | rural |
| I-0042-ABV | MDG52516151 | ABVR | human | M | 44 | 0 | yes | no | no | farmer | 2.439 | -0.727 | -0.774 | -0.516 | 27.52 | rural |
| I-0043-ABV | MDG52516151 | ABVR | human | M | 39 | 0 | no | no | no | farmer | 2.439 | -0.727 | -0.774 | -0.516 | 27.52 | rural |
| I-0032-ABV | MDG52516151 | ABVR | human | F | 28 | 0 | no | yes | no | other | 2.439 | -0.727 | -0.774 | -0.516 | 27.52 | rural |
| I-0045-ABV | MDG52516151 | ABVR | human | M | 28 | 0 | yes | no | no | farmer | 2.439 | -0.727 | -0.774 | -0.516 | 27.52 | rural |
| I-0046-ABV | MDG52516151 | ABVR | human | M | 27 | 0 | yes | no | no | farmer | 2.439 | -0.727 | -0.774 | -0.516 | 27.52 | rural |
| I-0047-ABV | MDG52516151 | ABVR | human | F | 19 | 0 | no | yes | no | other | 2.439 | -0.727 | -0.774 | -0.516 | 27.52 | rural |
| I-0060-ABV | MDG52516151 | ABVR | human | F | 53 | 0 | no | no | no | farmer | 2.439 | -0.727 | -0.774 | -0.516 | 27.52 | rural |
| I-0049-ABV | MDG52516151 | ABVR | human | M | 66 | 0 | no | yes | no | farmer | 2.439 | -0.727 | -0.774 | -0.516 | 27.52 | rural |
| I-0048-ABV | MDG52516151 | ABVR | human | M | 19 | 0 | no | yes | no | farmer | 2.439 | -0.727 | -0.774 | -0.516 | 27.52 | rural |
| I-0051-ABV | MDG52516151 | ABVR | human | F | 36 | 0 | yes | yes | no | farmer | 2.439 | -0.727 | -0.774 | -0.516 | 27.52 | rural |
| I-0052-ABV | MDG52516151 | ABVR | human | M | 37 | 0 | no | yes | no | farmer | 2.439 | -0.727 | -0.774 | -0.516 | 27.52 | rural |
| I-0054-ABV | MDG52516151 | ABVR | human | F | 22 | 0 | yes | no | no | farmer | 2.439 | -0.727 | -0.774 | -0.516 | 27.52 | rural |
| I-0056-ABV | MDG52516151 | ABVR | human | F | 23 | 0 | yes | no | no | farmer | 2.439 | -0.727 | -0.774 | -0.516 | 27.52 | rural |
| I-0057-ABV | MDG52516151 | ABVR | human | F | 20 | 0 | yes | no | no | farmer | 2.439 | -0.727 | -0.774 | -0.516 | 27.52 | rural |
| I-0058-ABV | MDG52516151 | ABVR | human | M | 21 | 0 | yes | no | no | farmer | 2.439 | -0.727 | -0.774 | -0.516 | 27.52 | rural |
| I-0059-ABV | MDG52516151 | ABVR | human | M | 23 | 0 | yes | yes | no | farmer | 2.439 | -0.727 | -0.774 | -0.516 | 27.52 | rural |
| I-0031-ABV | MDG52516151 | ABVR | human | M | 51 | 0 | yes | no | no | other | 2.439 | -0.727 | -0.774 | -0.516 | 27.52 | rural |
| I-0053-ABV | MDG52516151 | ABVR | human | M | 37 | 1 | yes | no | no | farmer | 2.439 | -0.727 | -0.774 | -0.516 | 27.52 | rural |
| I-0008-ABV | MDG52516151 | ABVU | human | F | 22 | 0 | yes | yes | no | other | 2.439 | -0.727 | -0.774 | -0.516 | 27.52 | rural |
| I-0030-ABV | MDG52516151 | ABVU | human | F | 37 | 0 | no | yes | no | other | 2.439 | -0.727 | -0.774 | -0.516 | 27.52 | rural |
| I-0050-ABV | MDG52516151 | ABVR | human | M | 70 | 0 | no | yes | no | farmer | 2.439 | -0.727 | -0.774 | -0.516 | 27.52 | rural |
| I-0001-ABV | MDG52516151 | ABVU | human | F | 41 | 0 | no | no | no | other | 2.439 | -0.727 | -0.774 | -0.516 | 27.52 | rural |
| I-0002-ABV | MDG52516151 | ABVU | human | M | 33 | 0 | no | no | no | other | 2.439 | -0.727 | -0.774 | -0.516 | 27.52 | rural |
| I-0003-ABV | MDG52516151 | ABVU | human | M | 35 | 0 | no | yes | no | farmer | 2.439 | -0.727 | -0.774 | -0.516 | 27.52 | rural |
| I-0004-ABV | MDG52516151 | ABVU | human | F | 33 | 0 | no | yes | no | farmer | 2.439 | -0.727 | -0.774 | -0.516 | 27.52 | rural |
| I-0005-ABV | MDG52516151 | ABVU | human | M | 48 | 0 | no | yes | no | other | 2.439 | -0.727 | -0.774 | -0.516 | 27.52 | rural |
| I-0007-ABV | MDG52516151 | ABVU | human | M | 20 | 0 | yes | yes | no | farmer | 2.439 | -0.727 | -0.774 | -0.516 | 27.52 | rural |
| I-0009-ABV | MDG52516151 | ABVU | human | F | 25 | 0 | no | yes | no | other | 2.439 | -0.727 | -0.774 | -0.516 | 27.52 | rural |
| I-0010-ABV | MDG52516151 | ABVU | human | M | 76 | 0 | yes | yes | no | farmer | 2.439 | -0.727 | -0.774 | -0.516 | 27.52 | rural |

| ID | PCODE | site2 | sp | gender | age | IgG | contact_ruminant | contact_milk | contact_fresh_fluid | profession | fact1 | fact2 | fact3 | fact4 | cattle_density | habitat |
| --- | --- | --- | --- | --- | --- | --- | --- | --- | --- | --- | --- | --- | --- | --- | --- | --- |
| I-0011-ABV | MDG52516151 | ABVU | human | M | 19 | 0 | yes | yes | no | other | 2.439 | -0.727 | -0.774 | -0.516 | 27.52 | rural |
| I-0012-ABV | MDG52516151 | ABVU | human | F | 43 | 0 | yes | no | no | other | 2.439 | -0.727 | -0.774 | -0.516 | 27.52 | rural |
| I-0013-ABV | MDG52516151 | ABVU | human | F | 45 | 0 | no | yes | no | other | 2.439 | -0.727 | -0.774 | -0.516 | 27.52 | rural |
| I-0014-ABV | MDG52516151 | ABVU | human | M | 28 | 0 | no | no | no | other | 2.439 | -0.727 | -0.774 | -0.516 | 27.52 | rural |
| I-0028-ABV | MDG52516151 | ABVU | human | F | 50 | 0 | yes | yes | no | farmer | 2.439 | -0.727 | -0.774 | -0.516 | 27.52 | rural |
| I-0006-ABV | MDG52516151 | ABVU | human | M | 62 | 0 | no | no | no | other | 2.439 | -0.727 | -0.774 | -0.516 | 27.52 | rural |
| I-0029-ABV | MDG52516151 | ABVU | human | M | 49 | 0 | no | yes | no | health | 2.439 | -0.727 | -0.774 | -0.516 | 27.52 | rural |
| I-0015-ABV | MDG52516151 | ABVU | human | M | 19 | 0 | yes | yes | no | farmer | 2.439 | -0.727 | -0.774 | -0.516 | 27.52 | rural |
| I-0027-ABV | MDG52516151 | ABVU | human | F | 31 | 0 | yes | no | no | farmer | 2.439 | -0.727 | -0.774 | -0.516 | 27.52 | rural |
| I-0026-ABV | MDG52516151 | ABVU | human | M | 38 | 0 | yes | yes | no | other | 2.439 | -0.727 | -0.774 | -0.516 | 27.52 | rural |
| I-0025-ABV | MDG52516151 | ABVU | human | M | 58 | 0 | yes | yes | no | other | 2.439 | -0.727 | -0.774 | -0.516 | 27.52 | rural |
| I-0024-ABV | MDG52516151 | ABVU | human | F | 23 | 0 | yes | yes | no | farmer | 2.439 | -0.727 | -0.774 | -0.516 | 27.52 | rural |
| I-0023-ABV | MDG52516151 | ABVU | human | F | 28 | 0 | no | yes | no | farmer | 2.439 | -0.727 | -0.774 | -0.516 | 27.52 | rural |
| I-0022-ABV | MDG52516151 | ABVU | human | F | 20 | 0 | no | yes | no | farmer | 2.439 | -0.727 | -0.774 | -0.516 | 27.52 | rural |
| I-0021-ABV | MDG52516151 | ABVU | human | F | 30 | 0 | no | yes | no | farmer | 2.439 | -0.727 | -0.774 | -0.516 | 27.52 | rural |
| I-0020-ABV | MDG52516151 | ABVU | human | F | 46 | 0 | no | yes | no | farmer | 2.439 | -0.727 | -0.774 | -0.516 | 27.52 | rural |
| I-0019-ABV | MDG52516151 | ABVU | human | F | 36 | 0 | no | no | no | farmer | 2.439 | -0.727 | -0.774 | -0.516 | 27.52 | rural |
| I-0018-ABV | MDG52516151 | ABVU | human | M | 42 | 0 | no | no | no | farmer | 2.439 | -0.727 | -0.774 | -0.516 | 27.52 | rural |
| I-0017-ABV | MDG52516151 | ABVU | human | F | 43 | 0 | no | yes | no | farmer | 2.439 | -0.727 | -0.774 | -0.516 | 27.52 | rural |
| I-0016-ABV | MDG52516151 | ABVU | human | M | 50 | 0 | yes | yes | no | farmer | 2.439 | -0.727 | -0.774 | -0.516 | 27.52 | rural |
| I-0049-TOL | MDG51520071 | TOLR | human | F | 47 | 0 | yes | no | no | farmer | 2.159 | -0.479 | -0.827 | 0.114 | 64.4285714285714 | rural |
| I-0058-TOL | MDG51520071 | TOLR | human | M | 39 | 0 | yes | no | no | other | 2.159 | -0.479 | -0.827 | 0.114 | 64.4285714285714 | rural |
| I-0055-TOL | MDG51520071 | TOLR | human | F | 29 | 0 | yes | no | no | contact_env | 2.159 | -0.479 | -0.827 | 0.114 | 64.4285714285714 | rural |
| I-0051-TOL | MDG51520071 | TOLR | human | F | 37 | 0 | no | no | no | farmer | 2.159 | -0.479 | -0.827 | 0.114 | 64.4285714285714 | rural |
| I-0052-TOL | MDG51520071 | TOLR | human | M | 26 | 0 | no | no | no | farmer | 2.159 | -0.479 | -0.827 | 0.114 | 64.4285714285714 | rural |
| I-0053-TOL | MDG51520071 | TOLR | human | M | 25 | 0 | yes | no | no | other | 2.159 | -0.479 | -0.827 | 0.114 | 64.4285714285714 | rural |
| I-0054-TOL | MDG51520071 | TOLR | human | M | 90 | 0 | no | no | no | farmer | 2.159 | -0.479 | -0.827 | 0.114 | 64.4285714285714 | rural |
| I-0050-TOL | MDG51520071 | TOLR | human | M | 20 | 0 | no | no | no | farmer | 2.159 | -0.479 | -0.827 | 0.114 | 64.4285714285714 | rural |
| I-0056-TOL | MDG51520071 | TOLR | human | F | 26 | 0 | yes | no | no | contact_env | 2.159 | -0.479 | -0.827 | 0.114 | 64.4285714285714 | rural |
| I-0048-TOL | MDG51520071 | TOLR | human | M | 18 | 0 | yes | no | no | other | 2.159 | -0.479 | -0.827 | 0.114 | 64.4285714285714 | rural |
| I-0059-TOL | MDG51520071 | TOLR | human | F | 56 | 0 | no | no | no | other | 2.159 | -0.479 | -0.827 | 0.114 | 64.4285714285714 | rural |
| I-0060-TOL | MDG51520071 | TOLR | human | M | 23 | 0 | yes | no | no | contact_env | 2.159 | -0.479 | -0.827 | 0.114 | 64.4285714285714 | rural |
| I-0035-TOL | MDG51520071 | TOLR | human | M | 40 | 0 | no | no | no | farmer | 2.159 | -0.479 | -0.827 | 0.114 | 64.4285714285714 | rural |
| I-0057-TOL | MDG51520071 | TOLR | human | M | 60 | 0 | no | no | no | contact_env | 2.159 | -0.479 | -0.827 | 0.114 | 64.4285714285714 | rural |
| I-0040-TOL | MDG51520071 | TOLR | human | M | 54 | 1 | yes | no | no | farmer | 2.159 | -0.479 | -0.827 | 0.114 | 64.4285714285714 | rural |
| I-0037-TOL | MDG51520071 | TOLR | human | M | 23 | 0 | yes | no | no | farmer | 2.159 | -0.479 | -0.827 | 0.114 | 64.4285714285714 | rural |
| I-0031-TOL | MDG51520071 | TOLR | human | M | 56 | 0 | yes | no | no | farmer | 2.159 | -0.479 | -0.827 | 0.114 | 64.4285714285714 | rural |
| I-0032-TOL | MDG51520071 | TOLR | human | M | 29 | 0 | yes | no | no | farmer | 2.159 | -0.479 | -0.827 | 0.114 | 64.4285714285714 | rural |
| I-0033-TOL | MDG51520071 | TOLR | human | F | 18 | 0 | no | no | no | other | 2.159 | -0.479 | -0.827 | 0.114 | 64.4285714285714 | rural |
| I-0034-TOL | MDG51520071 | TOLR | human | M | 45 | 0 | yes | no | no | farmer | 2.159 | -0.479 | -0.827 | 0.114 | 64.4285714285714 | rural |
| I-0036-TOL | MDG51520071 | TOLR | human | F | 37 | 0 | no | no | no | other | 2.159 | -0.479 | -0.827 | 0.114 | 64.4285714285714 | rural |
| I-0047-TOL | MDG51520071 | TOLR | human | M | 33 | 0 | no | no | no | farmer | 2.159 | -0.479 | -0.827 | 0.114 | 64.4285714285714 | rural |
| I-0039-TOL | MDG51520071 | TOLR | human | F | 42 | 0 | yes | no | no | other | 2.159 | -0.479 | -0.827 | 0.114 | 64.4285714285714 | rural |
| I-0041-TOL | MDG51520071 | TOLR | human | M | 36 | 0 | yes | no | no | farmer | 2.159 | -0.479 | -0.827 | 0.114 | 64.4285714285714 | rural |
| I-0042-TOL | MDG51520071 | TOLR | human | F | 71 | 0 | no | no | no | other | 2.159 | -0.479 | -0.827 | 0.114 | 64.4285714285714 | rural |
| I-0043-TOL | MDG51520071 | TOLR | human | M | 40 | 0 | yes | no | no | farmer | 2.159 | -0.479 | -0.827 | 0.114 | 64.4285714285714 | rural |
| I-0044-TOL | MDG51520071 | TOLR | human | F | 44 | 0 | no | no | no | other | 2.159 | -0.479 | -0.827 | 0.114 | 64.4285714285714 | rural |
| I-0045-TOL | MDG51520071 | TOLR | human | M | 23 | 0 | yes | yes | no | farmer | 2.159 | -0.479 | -0.827 | 0.114 | 64.4285714285714 | rural |
| I-0046-TOL | MDG51520071 | TOLR | human | M | 30 | 0 | yes | no | no | farmer | 2.159 | -0.479 | -0.827 | 0.114 | 64.4285714285714 | rural |
| I-0038-TOL | MDG51520071 | TOLR | human | F | 27 | 0 | yes | no | no | farmer | 2.159 | -0.479 | -0.827 | 0.114 | 64.4285714285714 | rural |
| I-0019-SBV | MDG72711010 | SBVU | human | F | 39 | 0 | no | no | no | other | -1.341 | -0.612 | -1.416 | 0.406 | 1.8 | rural |
| I-0020-SBV | MDG72711010 | SBVU | human | F | 34 | 0 | no | no | no | other | -1.341 | -0.612 | -1.416 | 0.406 | 1.8 | rural |
| I-0018-SBV | MDG72711010 | SBVU | human | F | 25 | 0 | no | no | no | other | -1.341 | -0.612 | -1.416 | 0.406 | 1.8 | rural |
| I-0021-SBV | MDG72711010 | SBVU | human | F | 57 | 0 | no | no | no | other | -1.341 | -0.612 | -1.416 | 0.406 | 1.8 | rural |
| I-0022-SBV | MDG72711010 | SBVU | human | M | 65 | 0 | no | no | no | other | -1.341 | -0.612 | -1.416 | 0.406 | 1.8 | rural |
| I-0061-SBV | MDG72711010 | SBVU | human | F | 61 | 0 | no | no | no | other | -1.341 | -0.612 | -1.416 | 0.406 | 1.8 | rural |
| I-0023-SBV | MDG72711010 | SBVU | human | F | 35 | 0 | no | no | no | other | -1.341 | -0.612 | -1.416 | 0.406 | 1.8 | rural |
| I-0024-SBV | MDG72711010 | SBVU | human | F | 23 | 0 | no | no | no | other | -1.341 | -0.612 | -1.416 | 0.406 | 1.8 | rural |
| I-0025-SBV | MDG72711010 | SBVU | human | M | 52 | 0 | no | no | no | farmer | -1.341 | -0.612 | -1.416 | 0.406 | 1.8 | rural |
| I-0026-SBV | MDG72711010 | SBVU | human | F | 35 | 0 | yes | no | no | other | -1.341 | -0.612 | -1.416 | 0.406 | 1.8 | rural |
| I-0027-SBV | MDG72711010 | SBVU | human | M | 58 | 0 | no | no | no | other | -1.341 | -0.612 | -1.416 | 0.406 | 1.8 | rural |
| I-0030-SBV | MDG72711010 | SBVU | human | F | 34 | 0 | no | no | no | other | -1.341 | -0.612 | -1.416 | 0.406 | 1.8 | rural |
| I-0007-SBV | MDG72711010 | SBVU | human | M | 24 | 0 | no | no | no | other | -1.341 | -0.612 | -1.416 | 0.406 | 1.8 | rural |

| ID | PCODE | site2 | sp | gender | age | IgG | contact_ruminant | contact_milk | contact_fresh_fluid | profession | fact1 | fact2 | fact3 | fact4 | cattle_density | habitat |
| --- | --- | --- | --- | --- | --- | --- | --- | --- | --- | --- | --- | --- | --- | --- | --- | --- |
| I-0017-SBV | MDG72711010 | SBVU | human | M | 45 | 0 | no | no | no | other | -1.341 | -0.612 | -1.416 | 0.406 | 1.8 | rural |
| I-0029-SBV | MDG72711010 | SBVU | human | F | 42 | 0 | no | no | no | other | -1.341 | -0.612 | -1.416 | 0.406 | 1.8 | rural |
| I-0004-SBV | MDG72711010 | SBVU | human | F | 20 | 0 | yes | yes | no | other | -1.341 | -0.612 | -1.416 | 0.406 | 1.8 | rural |
| I-0009-SBV | MDG72711010 | SBVU | human | F | 21 | 0 | no | no | no | other | -1.341 | -0.612 | -1.416 | 0.406 | 1.8 | rural |
| I-0001-SBV | MDG72711010 | SBVU | human | F | 30 | 0 | no | no | no | other | -1.341 | -0.612 | -1.416 | 0.406 | 1.8 | rural |
| I-0016-SBV | MDG72711010 | SBVU | human | F | 62 | 0 | no | no | no | other | -1.341 | -0.612 | -1.416 | 0.406 | 1.8 | rural |
| I-0003-SBV | MDG72711010 | SBVU | human | F | 35 | 0 | no | no | no | other | -1.341 | -0.612 | -1.416 | 0.406 | 1.8 | rural |
| I-0005-SBV | MDG72711010 | SBVU | human | F | 59 | 0 | no | no | no | other | -1.341 | -0.612 | -1.416 | 0.406 | 1.8 | rural |
| I-0006-SBV | MDG72711010 | SBVU | human | F | 41 | 0 | no | no | no | other | -1.341 | -0.612 | -1.416 | 0.406 | 1.8 | rural |
| I-0008-SBV | MDG72711010 | SBVU | human | M | 46 | 0 | no | no | no | other | -1.341 | -0.612 | -1.416 | 0.406 | 1.8 | rural |
| I-0010-SBV | MDG72711010 | SBVU | human | M | 35 | 0 | no | no | no | other | -1.341 | -0.612 | -1.416 | 0.406 | 1.8 | rural |
| I-0011-SBV | MDG72711010 | SBVU | human | M | 25 | 0 | no | no | no | other | -1.341 | -0.612 | -1.416 | 0.406 | 1.8 | rural |
| I-0012-SBV | MDG72711010 | SBVU | human | F | 34 | 0 | no | no | no | other | -1.341 | -0.612 | -1.416 | 0.406 | 1.8 | rural |
| I-0013-SBV | MDG72711010 | SBVU | human | M | 31 | 0 | no | no | no | other | -1.341 | -0.612 | -1.416 | 0.406 | 1.8 | rural |
| I-0014-SBV | MDG72711010 | SBVU | human | M | 31 | 0 | no | no | no | other | -1.341 | -0.612 | -1.416 | 0.406 | 1.8 | rural |
| I-0015-SBV | MDG72711010 | SBVU | human | F | 38 | 1 | no | no | no | other | -1.341 | -0.612 | -1.416 | 0.406 | 1.8 | rural |
| I-0002-SBV | MDG72711010 | SBVU | human | M | 34 | 0 | no | no | no | other | -1.341 | -0.612 | -1.416 | 0.406 | 1.8 | rural |
| I-0059-SBV | MDG72711079 | SBVR | human | F | 36 | 0 | yes | no | no | farmer | -1.278 | -0.698 | -1.442 | 0.438 | 1.8 | rural |
| I-0047-SBV | MDG72711079 | SBVR | human | M | 19 | 0 | no | no | no | farmer | -1.278 | -0.698 | -1.442 | 0.438 | 1.8 | rural |
| I-0048-SBV | MDG72711079 | SBVR | human | M | 28 | 0 | yes | no | no | other | -1.278 | -0.698 | -1.442 | 0.438 | 1.8 | rural |
| I-0049-SBV | MDG72711079 | SBVR | human | F | 24 | 0 | yes | no | no | farmer | -1.278 | -0.698 | -1.442 | 0.438 | 1.8 | rural |
| I-0050-SBV | MDG72711079 | SBVR | human | M | 27 | 1 | no | no | no | farmer | -1.278 | -0.698 | -1.442 | 0.438 | 1.8 | rural |
| I-0051-SBV | MDG72711079 | SBVR | human | M | 22 | 0 | yes | no | no | farmer | -1.278 | -0.698 | -1.442 | 0.438 | 1.8 | rural |
| I-0052-SBV | MDG72711079 | SBVR | human | M | 29 | 1 | no | no | no | other | -1.278 | -0.698 | -1.442 | 0.438 | 1.8 | rural |
| I-0053-SBV | MDG72711079 | SBVR | human | M | 54 | 0 | no | no | no | contact_env | -1.278 | -0.698 | -1.442 | 0.438 | 1.8 | rural |
| I-0054-SBV | MDG72711079 | SBVR | human | M | 20 | 1 | no | no | no | other | -1.278 | -0.698 | -1.442 | 0.438 | 1.8 | rural |
| I-0055-SBV | MDG72711079 | SBVR | human | F | 20 | 0 | no | no | no | farmer | -1.278 | -0.698 | -1.442 | 0.438 | 1.8 | rural |
| I-0056-SBV | MDG72711079 | SBVR | human | M | 28 | 0 | no | no | no | contact_env | -1.278 | -0.698 | -1.442 | 0.438 | 1.8 | rural |
| I-0046-SBV | MDG72711079 | SBVR | human | F | 72 | 0 | no | no | no | farmer | -1.278 | -0.698 | -1.442 | 0.438 | 1.8 | rural |
| I-0058-SBV | MDG72711079 | SBVR | human | F | 50 | 0 | no | no | no | contact_env | -1.278 | -0.698 | -1.442 | 0.438 | 1.8 | rural |
| I-0037-SBV | MDG72711079 | SBVR | human | M | 36 | 1 | no | no | no | farmer | -1.278 | -0.698 | -1.442 | 0.438 | 1.8 | rural |
| I-0057-SBV | MDG72711079 | SBVR | human | F | 24 | 0 | no | no | no | other | -1.278 | -0.698 | -1.442 | 0.438 | 1.8 | rural |
| I-0045-SBV | MDG72711079 | SBVR | human | F | 31 | 0 | no | no | no | other | -1.278 | -0.698 | -1.442 | 0.438 | 1.8 | rural |
| I-0044-SBV | MDG72711079 | SBVR | human | F | 37 | 0 | no | no | no | farmer | -1.278 | -0.698 | -1.442 | 0.438 | 1.8 | rural |
| I-0043-SBV | MDG72711079 | SBVR | human | F | 20 | 0 | no | no | no | other | -1.278 | -0.698 | -1.442 | 0.438 | 1.8 | rural |
| I-0042-SBV | MDG72711079 | SBVR | human | M | 46 | 1 | no | yes | no | farmer | -1.278 | -0.698 | -1.442 | 0.438 | 1.8 | rural |
| I-0041-SBV | MDG72711079 | SBVR | human | F | 61 | 0 | no | no | no | health | -1.278 | -0.698 | -1.442 | 0.438 | 1.8 | rural |
| I-0040-SBV | MDG72711079 | SBVR | human | M | 28 | 1 | yes | no | no | other | -1.278 | -0.698 | -1.442 | 0.438 | 1.8 | rural |
| I-0038-SBV | MDG72711079 | SBVR | human | F | 49 | 0 | no | no | no | farmer | -1.278 | -0.698 | -1.442 | 0.438 | 1.8 | rural |
| I-0036-SBV | MDG72711079 | SBVR | human | F | 45 | 1 | no | no | no | other | -1.278 | -0.698 | -1.442 | 0.438 | 1.8 | rural |
| I-0035-SBV | MDG72711079 | SBVR | human | M | 48 | 1 | no | no | no | farmer | -1.278 | -0.698 | -1.442 | 0.438 | 1.8 | rural |
| I-0034-SBV | MDG72711079 | SBVR | human | F | 33 | 0 | yes | no | no | farmer | -1.278 | -0.698 | -1.442 | 0.438 | 1.8 | rural |
| I-0033-SBV | MDG72711079 | SBVR | human | M | 31 | 1 | yes | no | no | farmer | -1.278 | -0.698 | -1.442 | 0.438 | 1.8 | rural |
| I-0032-SBV | MDG72711079 | SBVR | human | F | 35 | 1 | no | no | no | farmer | -1.278 | -0.698 | -1.442 | 0.438 | 1.8 | rural |
| I-0031-SBV | MDG72711079 | SBVR | human | M | 48 | 0 | yes | no | no | other | -1.278 | -0.698 | -1.442 | 0.438 | 1.8 | rural |
| I-0060-SBV | MDG72711079 | SBVR | human | M | 26 | 0 | yes | no | no | farmer | -1.278 | -0.698 | -1.442 | 0.438 | 1.8 | rural |
| I-0039-SBV | MDG72711079 | SBVR | human | F | 30 | 0 | yes | no | no | farmer | -1.278 | -0.698 | -1.442 | 0.438 | 1.8 | rural |
| I-0044-DIE | MDG71715009 | DIER | human | F | 39 | 0 | yes | no | no | farmer | 1.473 | 0.046 | -1.476 | 0.089 | 108.5 | rural |
| I-0043-DIE | MDG71715009 | DIER | human | M | 63 | 0 | yes | no | no | other | 1.473 | 0.046 | -1.476 | 0.089 | 108.5 | rural |
| I-0042-DIE | MDG71715009 | DIER | human | M | 71 | 0 | yes | yes | no | farmer | 1.473 | 0.046 | -1.476 | 0.089 | 108.5 | rural |
| I-0041-DIE | MDG71715009 | DIER | human | M | 46 | 0 | yes | no | no | farmer | 1.473 | 0.046 | -1.476 | 0.089 | 108.5 | rural |
| I-0040-DIE | MDG71715009 | DIER | human | F | 58 | 0 | yes | no | no | other | 1.473 | 0.046 | -1.476 | 0.089 | 108.5 | rural |
| I-0039-DIE | MDG71715009 | DIER | human | M | 59 | 1 | no | no | no | other | 1.473 | 0.046 | -1.476 | 0.089 | 108.5 | rural |
| I-0038-DIE | MDG71715009 | DIER | human | F | 30 | 0 | yes | no | no | farmer | 1.473 | 0.046 | -1.476 | 0.089 | 108.5 | rural |
| I-0037-DIE | MDG71715009 | DIER | human | M | 37 | 0 | yes | no | no | farmer | 1.473 | 0.046 | -1.476 | 0.089 | 108.5 | rural |
| I-0036-DIE | MDG71715009 | DIER | human | M | 39 | 0 | yes | no | no | farmer | 1.473 | 0.046 | -1.476 | 0.089 | 108.5 | rural |
| I-0035-DIE | MDG71715009 | DIER | human | F | 25 | 0 | no | no | no | farmer | 1.473 | 0.046 | -1.476 | 0.089 | 108.5 | rural |
| I-0045-DIE | MDG71715009 | DIER | human | F | 28 | 0 | yes | no | no | farmer | 1.473 | 0.046 | -1.476 | 0.089 | 108.5 | rural |
| I-0033-DIE | MDG71715009 | DIER | human | M | 54 | 0 | no | no | no | farmer | 1.473 | 0.046 | -1.476 | 0.089 | 108.5 | rural |
| I-0054-DIE | MDG71715009 | DIER | human | F | 33 | 0 | no | no | no | farmer | 1.473 | 0.046 | -1.476 | 0.089 | 108.5 | rural |
| I-0034-DIE | MDG71715009 | DIER | human | M | 21 | 0 | yes | no | no | farmer | 1.473 | 0.046 | -1.476 | 0.089 | 108.5 | rural |
| I-0053-DIE | MDG71715009 | DIER | human | M | 64 | 0 | yes | no | no | farmer | 1.473 | 0.046 | -1.476 | 0.089 | 108.5 | rural |
| I-0021-DIE | MDG71715009 | DIEU | human | F | 29 | 0 | no | no | no | other | 1.473 | 0.046 | -1.476 | 0.089 | 108.5 | rural |

| ID | PCODE | site2 | sp | gender | age | IgG | contact_ruminant | contact_milk | contact_fresh_fluid | profession | fact1 | fact2 | fact3 | fact4 | cattle_density | habitat |
| --- | --- | --- | --- | --- | --- | --- | --- | --- | --- | --- | --- | --- | --- | --- | --- | --- |
| I-0032-DIE | MDG71715009 | DIER | human | F | 27 | 0 | no | no | no | farmer | 1.473 | 0.046 | -1.476 | 0.089 | 108.5 | rural |
| I-0059-DIE | MDG71715009 | DIER | human | F | 25 | 0 | no | no | no | farmer | 1.473 | 0.046 | -1.476 | 0.089 | 108.5 | rural |
| I-0058-DIE | MDG71715009 | DIER | human | F | 25 | 0 | no | no | no | farmer | 1.473 | 0.046 | -1.476 | 0.089 | 108.5 | rural |
| I-0057-DIE | MDG71715009 | DIER | human | F | 26 | 0 | no | no | no | other | 1.473 | 0.046 | -1.476 | 0.089 | 108.5 | rural |
| I-0052-DIE | MDG71715009 | DIER | human | M | 26 | 0 | yes | no | no | farmer | 1.473 | 0.046 | -1.476 | 0.089 | 108.5 | rural |
| I-0055-DIE | MDG71715009 | DIER | human | F | 22 | 0 | no | no | no | other | 1.473 | 0.046 | -1.476 | 0.089 | 108.5 | rural |
| I-0046-DIE | MDG71715009 | DIER | human | M | 33 | 0 | yes | no | no | farmer | 1.473 | 0.046 | -1.476 | 0.089 | 108.5 | rural |
| I-0060-DIE | MDG71715009 | DIER | human | F | 30 | 0 | no | no | no | health | 1.473 | 0.046 | -1.476 | 0.089 | 108.5 | rural |
| I-0051-DIE | MDG71715009 | DIER | human | F | 29 | 0 | no | no | no | farmer | 1.473 | 0.046 | -1.476 | 0.089 | 108.5 | rural |
| I-0050-DIE | MDG71715009 | DIER | human | M | 20 | 0 | no | no | no | other | 1.473 | 0.046 | -1.476 | 0.089 | 108.5 | rural |
| I-0049-DIE | MDG71715009 | DIER | human | M | 53 | 0 | yes | no | no | other | 1.473 | 0.046 | -1.476 | 0.089 | 108.5 | rural |
| I-0048-DIE | MDG71715009 | DIER | human | F | 30 | 0 | yes | no | no | farmer | 1.473 | 0.046 | -1.476 | 0.089 | 108.5 | rural |
| I-0047-DIE | MDG71715009 | DIER | human | F | 60 | 0 | no | no | no | farmer | 1.473 | 0.046 | -1.476 | 0.089 | 108.5 | rural |
| I-0056-DIE | MDG71715009 | DIER | human | F | 38 | 0 | no | no | no | other | 1.473 | 0.046 | -1.476 | 0.089 | 108.5 | rural |
| I-0009-DIE | MDG71715009 | DIEU | human | M | 26 | 0 | no | no | no | other | 1.473 | 0.046 | -1.476 | 0.089 | 108.5 | rural |
| I-0001-DIE | MDG71715009 | DIEU | human | F | 27 | 1 | no | no | no | other | 1.473 | 0.046 | -1.476 | 0.089 | 108.5 | rural |
| I-0002-DIE | MDG71715009 | DIEU | human | M | 55 | 0 | no | no | no | other | 1.473 | 0.046 | -1.476 | 0.089 | 108.5 | rural |
| I-0003-DIE | MDG71715009 | DIEU | human | M | 21 | 0 | no | no | no | other | 1.473 | 0.046 | -1.476 | 0.089 | 108.5 | rural |
| I-0004-DIE | MDG71715009 | DIEU | human | M | 22 | 0 | no | no | no | other | 1.473 | 0.046 | -1.476 | 0.089 | 108.5 | rural |
| I-0005-DIE | MDG71715009 | DIEU | human | M | 47 | 0 | no | no | no | farmer | 1.473 | 0.046 | -1.476 | 0.089 | 108.5 | rural |
| I-0006-DIE | MDG71715009 | DIEU | human | M | 23 | 0 | no | no | no | other | 1.473 | 0.046 | -1.476 | 0.089 | 108.5 | rural |
| I-0023-DIE | MDG71715009 | DIEU | human | F | 35 | 0 | no | no | no | other | 1.473 | 0.046 | -1.476 | 0.089 | 108.5 | rural |
| I-0008-DIE | MDG71715009 | DIEU | human | M | 49 | 1 | no | no | no | other | 1.473 | 0.046 | -1.476 | 0.089 | 108.5 | rural |
| I-0031-DIE | MDG71715009 | DIER | human | M | 27 | 0 | yes | no | no | farmer | 1.473 | 0.046 | -1.476 | 0.089 | 108.5 | rural |
| I-0010-DIE | MDG71715009 | DIEU | human | M | 47 | 0 | no | no | no | other | 1.473 | 0.046 | -1.476 | 0.089 | 108.5 | rural |
| I-0011-DIE | MDG71715009 | DIEU | human | M | 22 | 1 | no | no | no | other | 1.473 | 0.046 | -1.476 | 0.089 | 108.5 | rural |
| I-0012-DIE | MDG71715009 | DIEU | human | M | 74 | 0 | no | no | no | other | 1.473 | 0.046 | -1.476 | 0.089 | 108.5 | rural |
| I-0013-DIE | MDG71715009 | DIEU | human | F | 52 | 0 | no | no | no | other | 1.473 | 0.046 | -1.476 | 0.089 | 108.5 | rural |
| I-0014-DIE | MDG71715009 | DIEU | human | F | 35 | 0 | no | no | no | other | 1.473 | 0.046 | -1.476 | 0.089 | 108.5 | rural |
| I-0026-DIE | MDG71715009 | DIEU | human | F | 62 | 0 | no | no | no | other | 1.473 | 0.046 | -1.476 | 0.089 | 108.5 | rural |
| I-0007-DIE | MDG71715009 | DIEU | human | M | 36 | 0 | no | no | no | other | 1.473 | 0.046 | -1.476 | 0.089 | 108.5 | rural |
| I-0015-DIE | MDG71715009 | DIEU | human | F | 41 | 0 | no | no | no | other | 1.473 | 0.046 | -1.476 | 0.089 | 108.5 | rural |
| I-0029-DIE | MDG71715009 | DIEU | human | M | 28 | 0 | no | no | no | farmer | 1.473 | 0.046 | -1.476 | 0.089 | 108.5 | rural |
| I-0027-DIE | MDG71715009 | DIEU | human | M | 37 | 0 | no | no | no | other | 1.473 | 0.046 | -1.476 | 0.089 | 108.5 | rural |
| I-0030-DIE | MDG71715009 | DIEU | human | F | 31 | 0 | no | no | no | other | 1.473 | 0.046 | -1.476 | 0.089 | 108.5 | rural |
| I-0025-DIE | MDG71715009 | DIEU | human | F | 33 | 0 | no | no | no | other | 1.473 | 0.046 | -1.476 | 0.089 | 108.5 | rural |
| I-0024-DIE | MDG71715009 | DIEU | human | M | 32 | 0 | no | no | no | other | 1.473 | 0.046 | -1.476 | 0.089 | 108.5 | rural |
| I-0022-DIE | MDG71715009 | DIEU | human | F | 24 | 0 | no | no | no | other | 1.473 | 0.046 | -1.476 | 0.089 | 108.5 | rural |
| I-0020-DIE | MDG71715009 | DIEU | human | F | 34 | 0 | no | no | no | other | 1.473 | 0.046 | -1.476 | 0.089 | 108.5 | rural |
| I-0019-DIE | MDG71715009 | DIEU | human | F | 53 | 0 | no | no | no | other | 1.473 | 0.046 | -1.476 | 0.089 | 108.5 | rural |
| I-0018-DIE | MDG71715009 | DIEU | human | F | 70 | 0 | no | no | no | other | 1.473 | 0.046 | -1.476 | 0.089 | 108.5 | rural |
| I-0017-DIE | MDG71715009 | DIEU | human | F | 29 | 0 | no | no | no | other | 1.473 | 0.046 | -1.476 | 0.089 | 108.5 | rural |
| I-0016-DIE | MDG71715009 | DIEU | human | F | 32 | 0 | no | no | no | other | 1.473 | 0.046 | -1.476 | 0.089 | 108.5 | rural |
| I-0028-DIE | MDG71715009 | DIEU | human | M | 37 | 0 | no | no | no | other | 1.473 | 0.046 | -1.476 | 0.089 | 108.5 | rural |
| I-0030-NSB | MDG71718001 | NSBU | human | M | 58 | 1 | no | no | no | other | -0.146 | -1.161 | 0.339 | 0.156 | 18.3333333333333 | rural |
| I-0022-NSB | MDG71718001 | NSBU | human | F | 43 | 0 | no | no | no | other | -0.146 | -1.161 | 0.339 | 0.156 | 18.3333333333333 | rural |
| I-0017-NSB | MDG71718001 | NSBU | human | M | 34 | 0 | no | no | no | other | -0.146 | -1.161 | 0.339 | 0.156 | 18.3333333333333 | rural |
| I-0018-NSB | MDG71718001 | NSBU | human | F | 60 | 1 | no | no | no | health | -0.146 | -1.161 | 0.339 | 0.156 | 18.3333333333333 | rural |
| I-0019-NSB | MDG71718001 | NSBU | human | F | 52 | 0 | no | no | no | other | -0.146 | -1.161 | 0.339 | 0.156 | 18.3333333333333 | rural |
| I-0020-NSB | MDG71718001 | NSBU | human | M | 39 | 0 | no | no | no | other | -0.146 | -1.161 | 0.339 | 0.156 | 18.3333333333333 | rural |
| I-0021-NSB | MDG71718001 | NSBU | human | F | 22 | 0 | no | no | no | other | -0.146 | -1.161 | 0.339 | 0.156 | 18.3333333333333 | rural |
| I-0023-NSB | MDG71718001 | NSBU | human | F | 38 | 0 | no | no | no | other | -0.146 | -1.161 | 0.339 | 0.156 | 18.3333333333333 | rural |
| I-0024-NSB | MDG71718001 | NSBU | human | F | 54 | 1 | no | yes | no | other | -0.146 | -1.161 | 0.339 | 0.156 | 18.3333333333333 | rural |
| I-0025-NSB | MDG71718001 | NSBU | human | M | 60 | 0 | no | no | no | other | -0.146 | -1.161 | 0.339 | 0.156 | 18.3333333333333 | rural |
| I-0026-NSB | MDG71718001 | NSBU | human | F | 48 | 0 | no | no | no | other | -0.146 | -1.161 | 0.339 | 0.156 | 18.3333333333333 | rural |
| I-0027-NSB | MDG71718001 | NSBU | human | F | 28 | 0 | no | no | no | other | -0.146 | -1.161 | 0.339 | 0.156 | 18.3333333333333 | rural |
| I-0029-NSB | MDG71718001 | NSBU | human | F | 21 | 0 | no | no | no | other | -0.146 | -1.161 | 0.339 | 0.156 | 18.3333333333333 | rural |
| I-0014-NSB | MDG71718001 | NSBU | human | F | 27 | 0 | no | no | no | other | -0.146 | -1.161 | 0.339 | 0.156 | 18.3333333333333 | rural |
| I-0028-NSB | MDG71718001 | NSBU | human | F | 27 | 0 | no | no | no | other | -0.146 | -1.161 | 0.339 | 0.156 | 18.3333333333333 | rural |
| I-0016-NSB | MDG71718001 | NSBU | human | M | 34 | 0 | no | no | no | other | -0.146 | -1.161 | 0.339 | 0.156 | 18.3333333333333 | rural |
| I-0015-NSB | MDG71718001 | NSBU | human | M | 22 | 0 | no | yes | no | other | -0.146 | -1.161 | 0.339 | 0.156 | 18.3333333333333 | rural |
| I-0001-NSB | MDG71718001 | NSBU | human | F | 19 | 1 | no | no | no | other | -0.146 | -1.161 | 0.339 | 0.156 | 18.3333333333333 | rural |
| I-0002-NSB | MDG71718001 | NSBU | human | F | 44 | 0 | no | yes | no | other | -0.146 | -1.161 | 0.339 | 0.156 | 18.3333333333333 | rural |

| ID | PCODE | site2 | sp | gender | age | IgG | contact_ruminant | contact_milk | contact_fresh_fluid | profession | fact1 | fact2 | fact3 | fact4 | cattle_density | habitat |
| --- | --- | --- | --- | --- | --- | --- | --- | --- | --- | --- | --- | --- | --- | --- | --- | --- |
| I-0003-NSB | MDG71718001 | NSBU | human | M | 20 | 1 | no | yes | no | other | -0.146 | -1.161 | 0.339 | 0.156 | 18.3333333333333 | rural |
| I-0004-NSB | MDG71718001 | NSBU | human | M | 78 | 1 | no | no | no | other | -0.146 | -1.161 | 0.339 | 0.156 | 18.3333333333333 | rural |
| I-0005-NSB | MDG71718001 | NSBU | human | F | 26 | 0 | no | no | no | other | -0.146 | -1.161 | 0.339 | 0.156 | 18.3333333333333 | rural |
| I-0006-NSB | MDG71718001 | NSBU | human | F | 53 | 0 | no | no | no | other | -0.146 | -1.161 | 0.339 | 0.156 | 18.3333333333333 | rural |
| I-0008-NSB | MDG71718001 | NSBU | human | M | 54 | 0 | no | no | no | health | -0.146 | -1.161 | 0.339 | 0.156 | 18.3333333333333 | rural |
| I-0009-NSB | MDG71718001 | NSBU | human | M | 33 | 0 | no | no | no | other | -0.146 | -1.161 | 0.339 | 0.156 | 18.3333333333333 | rural |
| I-0010-NSB | MDG71718001 | NSBU | human | F | 32 | 0 | no | no | no | other | -0.146 | -1.161 | 0.339 | 0.156 | 18.3333333333333 | rural |
| I-0011-NSB | MDG71718001 | NSBU | human | M | 21 | 0 | no | no | no | other | -0.146 | -1.161 | 0.339 | 0.156 | 18.3333333333333 | rural |
| I-0012-NSB | MDG71718001 | NSBU | human | F | 43 | 0 | no | no | no | other | -0.146 | -1.161 | 0.339 | 0.156 | 18.3333333333333 | rural |
| I-0013-NSB | MDG71718001 | NSBU | human | F | 36 | 0 | no | no | no | other | -0.146 | -1.161 | 0.339 | 0.156 | 18.3333333333333 | rural |
| I-0007-NSB | MDG71718001 | NSBU | human | M | 53 | 0 | no | no | no | health | -0.146 | -1.161 | 0.339 | 0.156 | 18.3333333333333 | rural |
| I-0058-NSB | MDG71718003 | NSBR | human | F | 40 | 0 | yes | no | no | farmer | -1.067 | -1.867 | -0.025 | 0.248 | 19.1999999999999 | rural |
| I-0048-NSB | MDG71718003 | NSBR | human | F | 38 | 0 | no | no | no | farmer | -1.067 | -1.867 | -0.025 | 0.248 | 19.1999999999999 | rural |
| I-0049-NSB | MDG71718003 | NSBR | human | F | 28 | 0 | no | no | no | health | -1.067 | -1.867 | -0.025 | 0.248 | 19.1999999999999 | rural |
| I-0050-NSB | MDG71718003 | NSBR | human | M | 28 | 1 | no | no | no | other | -1.067 | -1.867 | -0.025 | 0.248 | 19.1999999999999 | rural |
| I-0051-NSB | MDG71718003 | NSBR | human | F | 28 | 0 | no | no | no | farmer | -1.067 | -1.867 | -0.025 | 0.248 | 19.1999999999999 | rural |
| I-0052-NSB | MDG71718003 | NSBR | human | M | 61 | 1 | yes | no | no | farmer | -1.067 | -1.867 | -0.025 | 0.248 | 19.1999999999999 | rural |
| I-0053-NSB | MDG71718003 | NSBR | human | F | 37 | 0 | no | no | no | farmer | -1.067 | -1.867 | -0.025 | 0.248 | 19.1999999999999 | rural |
| I-0054-NSB | MDG71718003 | NSBR | human | M | 22 | 0 | yes | no | no | farmer | -1.067 | -1.867 | -0.025 | 0.248 | 19.1999999999999 | rural |
| I-0055-NSB | MDG71718003 | NSBR | human | F | 58 | 0 | no | no | no | farmer | -1.067 | -1.867 | -0.025 | 0.248 | 19.1999999999999 | rural |
| I-0060-NSB | MDG71718003 | NSBR | human | F | 37 | 0 | no | no | no | farmer | -1.067 | -1.867 | -0.025 | 0.248 | 19.1999999999999 | rural |
| I-0057-NSB | MDG71718003 | NSBR | human | F | 44 | 0 | no | no | no | other | -1.067 | -1.867 | -0.025 | 0.248 | 19.1999999999999 | rural |
| I-0059-NSB | MDG71718003 | NSBR | human | M | 49 | 1 | yes | no | no | farmer | -1.067 | -1.867 | -0.025 | 0.248 | 19.1999999999999 | rural |
| I-0046-NSB | MDG71718003 | NSBR | human | M | 24 | 0 | no | no | no | farmer | -1.067 | -1.867 | -0.025 | 0.248 | 19.1999999999999 | rural |
| I-0045-NSB | MDG71718003 | NSBR | human | F | 37 | 0 | no | no | no | farmer | -1.067 | -1.867 | -0.025 | 0.248 | 19.1999999999999 | rural |
| I-0056-NSB | MDG71718003 | NSBR | human | M | 20 | 0 | no | no | no | farmer | -1.067 | -1.867 | -0.025 | 0.248 | 19.1999999999999 | rural |
| I-0036-NSB | MDG71718003 | NSBR | human | F | 59 | 0 | no | no | no | farmer | -1.067 | -1.867 | -0.025 | 0.248 | 19.1999999999999 | rural |
| I-0047-NSB | MDG71718003 | NSBR | human | M | 37 | 0 | no | no | no | farmer | -1.067 | -1.867 | -0.025 | 0.248 | 19.1999999999999 | rural |
| I-0033-NSB | MDG71718003 | NSBR | human | M | 61 | 0 | yes | yes | no | other | -1.067 | -1.867 | -0.025 | 0.248 | 19.1999999999999 | rural |
| I-0031-NSB | MDG71718003 | NSBR | human | M | 48 | 0 | yes | no | no | other | -1.067 | -1.867 | -0.025 | 0.248 | 19.1999999999999 | rural |
| I-0035-NSB | MDG71718003 | NSBR | human | M | 75 | 0 | yes | no | no | farmer | -1.067 | -1.867 | -0.025 | 0.248 | 19.1999999999999 | rural |
| I-0032-NSB | MDG71718003 | NSBR | human | M | 24 | 0 | yes | yes | no | farmer | -1.067 | -1.867 | -0.025 | 0.248 | 19.1999999999999 | rural |
| I-0037-NSB | MDG71718003 | NSBR | human | F | 20 | 0 | yes | no | no | other | -1.067 | -1.867 | -0.025 | 0.248 | 19.1999999999999 | rural |
| I-0038-NSB | MDG71718003 | NSBR | human | F | 54 | 0 | no | no | no | farmer | -1.067 | -1.867 | -0.025 | 0.248 | 19.1999999999999 | rural |
| I-0039-NSB | MDG71718003 | NSBR | human | M | 32 | 0 | no | no | no | farmer | -1.067 | -1.867 | -0.025 | 0.248 | 19.1999999999999 | rural |
| I-0040-NSB | MDG71718003 | NSBR | human | F | 70 | 0 | no | no | no | farmer | -1.067 | -1.867 | -0.025 | 0.248 | 19.1999999999999 | rural |
| I-0041-NSB | MDG71718003 | NSBR | human | M | 56 | 1 | no | no | no | farmer | -1.067 | -1.867 | -0.025 | 0.248 | 19.1999999999999 | rural |
| I-0042-NSB | MDG71718003 | NSBR | human | M | 83 | 0 | no | no | no | farmer | -1.067 | -1.867 | -0.025 | 0.248 | 19.1999999999999 | rural |
| I-0043-NSB | MDG71718003 | NSBR | human | F | 40 | 0 | no | yes | no | other | -1.067 | -1.867 | -0.025 | 0.248 | 19.1999999999999 | rural |
| I-0044-NSB | MDG71718003 | NSBR | human | F | 23 | 0 | no | no | no | other | -1.067 | -1.867 | -0.025 | 0.248 | 19.1999999999999 | rural |
| I-0034-NSB | MDG71718003 | NSBR | human | F | 51 | 0 | no | no | no | farmer | -1.067 | -1.867 | -0.025 | 0.248 | 19.1999999999999 | rural |
| AMB_067 | MDG11103171 | NA | bovine | M | 8 | 0 | NA | NA | NA | NA | 0.050 | 1.206 | -0.427 | -0.229 | 14.25 | NA |
| AMB_018 | MDG11103171 | NA | bovine | M | 8 | 1 | NA | NA | NA | NA | 0.050 | 1.206 | -0.427 | -0.229 | 14.25 | NA |
| AMB_025 | MDG11103171 | NA | bovine | M | 8 | 1 | NA | NA | NA | NA | 0.050 | 1.206 | -0.427 | -0.229 | 14.25 | NA |
| AMB_033 | MDG11103171 | NA | bovine | M | 8 | 1 | NA | NA | NA | NA | 0.050 | 1.206 | -0.427 | -0.229 | 14.25 | NA |
| AMB_035 | MDG11103171 | NA | bovine | M | 2 | 1 | NA | NA | NA | NA | 0.050 | 1.206 | -0.427 | -0.229 | 14.25 | NA |
| AMB_053 | MDG11103171 | NA | bovine | M | 9 | 0 | NA | NA | NA | NA | 0.050 | 1.206 | -0.427 | -0.229 | 14.25 | NA |
| AMB_054 | MDG11103171 | NA | bovine | M | 9 | 0 | NA | NA | NA | NA | 0.050 | 1.206 | -0.427 | -0.229 | 14.25 | NA |
| AMB_055 | MDG11103171 | NA | bovine | F | 8 | 1 | NA | NA | NA | NA | 0.050 | 1.206 | -0.427 | -0.229 | 14.25 | NA |
| AMB_056 | MDG11103171 | NA | bovine | M | 8 | 1 | NA | NA | NA | NA | 0.050 | 1.206 | -0.427 | -0.229 | 14.25 | NA |
| AMB_062 | MDG11103171 | NA | bovine | M | 12 | 1 | NA | NA | NA | NA | 0.050 | 1.206 | -0.427 | -0.229 | 14.25 | NA |
| AMB_063 | MDG11103171 | NA | bovine | M | 8 | 0 | NA | NA | NA | NA | 0.050 | 1.206 | -0.427 | -0.229 | 14.25 | NA |
| AMB_064 | MDG11103171 | NA | bovine | M | 5 | 0 | NA | NA | NA | NA | 0.050 | 1.206 | -0.427 | -0.229 | 14.25 | NA |
| AMB_017 | MDG11103171 | NA | bovine | M | 12 | 1 | NA | NA | NA | NA | 0.050 | 1.206 | -0.427 | -0.229 | 14.25 | NA |
| AMB_066 | MDG11103171 | NA | bovine | M | 6 | 0 | NA | NA | NA | NA | 0.050 | 1.206 | -0.427 | -0.229 | 14.25 | NA |
| AMB_034 | MDG11103171 | NA | bovine | M | 9 | 1 | NA | NA | NA | NA | 0.050 | 1.206 | -0.427 | -0.229 | 14.25 | NA |
| AMB_068 | MDG11103171 | NA | bovine | M | 8 | 0 | NA | NA | NA | NA | 0.050 | 1.206 | -0.427 | -0.229 | 14.25 | NA |
| AMB_069 | MDG11103171 | NA | bovine | M | 7 | 0 | NA | NA | NA | NA | 0.050 | 1.206 | -0.427 | -0.229 | 14.25 | NA |
| AMB_076 | MDG11103171 | NA | bovine | M | 4 | 1 | NA | NA | NA | NA | 0.050 | 1.206 | -0.427 | -0.229 | 14.25 | NA |
| AMB_077 | MDG11103171 | NA | bovine | M | 8 | 0 | NA | NA | NA | NA | 0.050 | 1.206 | -0.427 | -0.229 | 14.25 | NA |
| AMB_078 | MDG11103171 | NA | bovine | M | 6 | 0 | NA | NA | NA | NA | 0.050 | 1.206 | -0.427 | -0.229 | 14.25 | NA |
| AMB_079 | MDG11103171 | NA | bovine | M | 5 | 0 | NA | NA | NA | NA | 0.050 | 1.206 | -0.427 | -0.229 | 14.25 | NA |
| AMB_080 | MDG11103171 | NA | bovine | M | 6 | 0 | NA | NA | NA | NA | 0.050 | 1.206 | -0.427 | -0.229 | 14.25 | NA |

| ID | PCODE | site2 | sp | gender | age | IgG | contact_ruminant | contact_milk | contact_fresh_fluid | profession | fact1 | fact2 | fact3 | fact4 | cattle_density | habitat |
| --- | --- | --- | --- | --- | --- | --- | --- | --- | --- | --- | --- | --- | --- | --- | --- | --- |
| AMB_081 | MDG11103171 | NA | bovine | M | 3 | 1 | NA | NA | NA | NA | 0.050 | 1.206 | -0.427 | -0.229 | 14.25 | NA |
| AMB_082 | MDG11103171 | NA | bovine | M | 8 | 0 | NA | NA | NA | NA | 0.050 | 1.206 | -0.427 | -0.229 | 14.25 | NA |
| AMB_083 | MDG11103171 | NA | bovine | M | 2 | 0 | NA | NA | NA | NA | 0.050 | 1.206 | -0.427 | -0.229 | 14.25 | NA |
| AMB_092 | MDG11103171 | NA | bovine | M | 2 | 0 | NA | NA | NA | NA | 0.050 | 1.206 | -0.427 | -0.229 | 14.25 | NA |
| AMB_097 | MDG11103171 | NA | bovine | M | 1 | 0 | NA | NA | NA | NA | 0.050 | 1.206 | -0.427 | -0.229 | 14.25 | NA |
| AMB_065 | MDG11103171 | NA | bovine | M | 5 | 1 | NA | NA | NA | NA | 0.050 | 1.206 | -0.427 | -0.229 | 14.25 | NA |
| AMB_036 | MDG11103171 | NA | bovine | F | 2 | 1 | NA | NA | NA | NA | 0.050 | 1.206 | -0.427 | -0.229 | 14.25 | NA |
| MANJ 023 | MDG11106030 | NA | bovine | F | 8 | 0 | NA | NA | NA | NA | -1.243 | 0.925 | -0.538 | -0.273 | 22 | NA |
| MANJ 017 | MDG11106030 | NA | bovine | F | 9 | 0 | NA | NA | NA | NA | -1.243 | 0.925 | -0.538 | -0.273 | 22 | NA |
| MANJ 022 | MDG11106050 | NA | bovine | F | 8 | 0 | NA | NA | NA | NA | -1.634 | 1.077 | -0.277 | -0.224 | 10 | NA |
| MANJ 016 | MDG11106050 | NA | bovine | F | 7 | 0 | NA | NA | NA | NA | -1.634 | 1.077 | -0.277 | -0.224 | 10 | NA |
| MANJ 033 | MDG11106050 | NA | bovine | F | 9 | 0 | NA | NA | NA | NA | -1.634 | 1.077 | -0.277 | -0.224 | 10 | NA |
| MANJ 061 | MDG11106130 | NA | bovine | M | 5 | 0 | NA | NA | NA | NA | -1.677 | 1.071 | -0.233 | -0.223 | 20.75 | NA |
| MANJ 060 | MDG11106130 | NA | bovine | M | 5 | 0 | NA | NA | NA | NA | -1.677 | 1.071 | -0.233 | -0.223 | 20.75 | NA |
| MANJ 059 | MDG11106130 | NA | bovine | M | 3 | 1 | NA | NA | NA | NA | -1.677 | 1.071 | -0.233 | -0.223 | 20.75 | NA |
| MANJ 057 | MDG11106130 | NA | bovine | M | 5 | 1 | NA | NA | NA | NA | -1.677 | 1.071 | -0.233 | -0.223 | 20.75 | NA |
| MANJ 012 | MDG11106170 | NA | bovine | F | 6 | 0 | NA | NA | NA | NA | -1.044 | 1.242 | -0.246 | -0.368 | 28.6666666666666 | NA |
| MANJ 015 | MDG11106170 | NA | bovine | F | 5 | 0 | NA | NA | NA | NA | -1.044 | 1.242 | -0.246 | -0.368 | 28.6666666666666 | NA |
| MANJ 013 | MDG11106170 | NA | bovine | F | 6 | 0 | NA | NA | NA | NA | -1.044 | 1.242 | -0.246 | -0.368 | 28.6666666666666 | NA |
| MANJ 011 | MDG11106170 | NA | bovine | F | 2 | 0 | NA | NA | NA | NA | -1.044 | 1.242 | -0.246 | -0.368 | 28.6666666666666 | NA |
| MANJ 010 | MDG11106170 | NA | bovine | F | 5 | 0 | NA | NA | NA | NA | -1.044 | 1.242 | -0.246 | -0.368 | 28.6666666666666 | NA |
| MANJ 008 | MDG11106170 | NA | bovine | F | 5 | 0 | NA | NA | NA | NA | -1.044 | 1.242 | -0.246 | -0.368 | 28.6666666666666 | NA |
| MANJ 007 | MDG11106170 | NA | bovine | M | 1 | 0 | NA | NA | NA | NA | -1.044 | 1.242 | -0.246 | -0.368 | 28.6666666666666 | NA |
| MANJ 006 | MDG11106170 | NA | bovine | F | 7 | 0 | NA | NA | NA | NA | -1.044 | 1.242 | -0.246 | -0.368 | 28.6666666666666 | NA |
| MANJ 005 | MDG11106170 | NA | bovine | M | 5 | 0 | NA | NA | NA | NA | -1.044 | 1.242 | -0.246 | -0.368 | 28.6666666666666 | NA |
| MANJ 004 | MDG11106170 | NA | bovine | F | 1 | 1 | NA | NA | NA | NA | -1.044 | 1.242 | -0.246 | -0.368 | 28.6666666666666 | NA |
| MANJ 003 | MDG11106170 | NA | bovine | F | 6 | 1 | NA | NA | NA | NA | -1.044 | 1.242 | -0.246 | -0.368 | 28.6666666666666 | NA |
| MANJ 002 | MDG11106170 | NA | bovine | F | 6 | 1 | NA | NA | NA | NA | -1.044 | 1.242 | -0.246 | -0.368 | 28.6666666666666 | NA |
| MANJ 001 | MDG11106170 | NA | bovine | F | 1 | 1 | NA | NA | NA | NA | -1.044 | 1.242 | -0.246 | -0.368 | 28.6666666666666 | NA |
| MANJ 014 | MDG11106170 | NA | bovine | F | 1 | 0 | NA | NA | NA | NA | -1.044 | 1.242 | -0.246 | -0.368 | 28.6666666666666 | NA |
| MANJ 009 | MDG11106170 | NA | bovine | F | 2 | 0 | NA | NA | NA | NA | -1.044 | 1.242 | -0.246 | -0.368 | 28.6666666666666 | NA |
| MANJ 058 | MDG11106251 | NA | bovine | M | 4 | 0 | NA | NA | NA | NA | -1.037 | 1.282 | -0.149 | -0.380 | 19.8 | NA |
| MANJ 064 | MDG11106251 | NA | bovine | M | 6 | 1 | NA | NA | NA | NA | -1.037 | 1.282 | -0.149 | -0.380 | 19.8 | NA |
| MANJ 049 | MDG11106271 | NA | bovine | M | 1 | 0 | NA | NA | NA | NA | -1.187 | 1.290 | -0.181 | -0.390 | 21.6 | NA |
| MANJ 062 | MDG11106271 | NA | bovine | M | 6 | 0 | NA | NA | NA | NA | -1.187 | 1.290 | -0.181 | -0.390 | 21.6 | NA |
| MANJ 055 | MDG11106271 | NA | bovine | M | 8 | 0 | NA | NA | NA | NA | -1.187 | 1.290 | -0.181 | -0.390 | 21.6 | NA |
| MANJ 054 | MDG11106271 | NA | bovine | M | 8 | 0 | NA | NA | NA | NA | -1.187 | 1.290 | -0.181 | -0.390 | 21.6 | NA |
| MANJ 043 | MDG11106271 | NA | bovine | M | 6 | 0 | NA | NA | NA | NA | -1.187 | 1.290 | -0.181 | -0.390 | 21.6 | NA |
| MANJ 053 | MDG11106271 | NA | bovine | M | 6 | 1 | NA | NA | NA | NA | -1.187 | 1.290 | -0.181 | -0.390 | 21.6 | NA |
| MANJ 052 | MDG11106271 | NA | bovine | M | 7 | 0 | NA | NA | NA | NA | -1.187 | 1.290 | -0.181 | -0.390 | 21.6 | NA |
| MANJ 065 | MDG11106271 | NA | bovine | M | 2 | 0 | NA | NA | NA | NA | -1.187 | 1.290 | -0.181 | -0.390 | 21.6 | NA |
| MANJ 050 | MDG11106271 | NA | bovine | F | 6 | 1 | NA | NA | NA | NA | -1.187 | 1.290 | -0.181 | -0.390 | 21.6 | NA |
| MANJ 048 | MDG11106271 | NA | bovine | F | 4 | 0 | NA | NA | NA | NA | -1.187 | 1.290 | -0.181 | -0.390 | 21.6 | NA |
| MANJ 047 | MDG11106271 | NA | bovine | M | 5 | 0 | NA | NA | NA | NA | -1.187 | 1.290 | -0.181 | -0.390 | 21.6 | NA |
| MANJ 046 | MDG11106271 | NA | bovine | M | 4 | 0 | NA | NA | NA | NA | -1.187 | 1.290 | -0.181 | -0.390 | 21.6 | NA |
| MANJ 045 | MDG11106271 | NA | bovine | M | 6 | 0 | NA | NA | NA | NA | -1.187 | 1.290 | -0.181 | -0.390 | 21.6 | NA |
| MANJ 044 | MDG11106271 | NA | bovine | M | 6 | 0 | NA | NA | NA | NA | -1.187 | 1.290 | -0.181 | -0.390 | 21.6 | NA |
| MANJ 067 | MDG11106271 | NA | bovine | M | 7 | 1 | NA | NA | NA | NA | -1.187 | 1.290 | -0.181 | -0.390 | 21.6 | NA |
| MANJ 051 | MDG11106271 | NA | bovine | F | 6 | 1 | NA | NA | NA | NA | -1.187 | 1.290 | -0.181 | -0.390 | 21.6 | NA |
| MANJ 041 | MDG11106271 | NA | bovine | F | 8 | 0 | NA | NA | NA | NA | -1.187 | 1.290 | -0.181 | -0.390 | 21.6 | NA |
| MANJ 068 | MDG11106271 | NA | bovine | M | 8 | 0 | NA | NA | NA | NA | -1.187 | 1.290 | -0.181 | -0.390 | 21.6 | NA |
| MANJ 069 | MDG11106271 | NA | bovine | M | 3 | 0 | NA | NA | NA | NA | -1.187 | 1.290 | -0.181 | -0.390 | 21.6 | NA |
| MANJ 070 | MDG11106271 | NA | bovine | M | 5 | 0 | NA | NA | NA | NA | -1.187 | 1.290 | -0.181 | -0.390 | 21.6 | NA |
| MANJ 072 | MDG11106271 | NA | bovine | F | 2 | 0 | NA | NA | NA | NA | -1.187 | 1.290 | -0.181 | -0.390 | 21.6 | NA |
| MANJ 073 | MDG11106271 | NA | bovine | M | 7 | 1 | NA | NA | NA | NA | -1.187 | 1.290 | -0.181 | -0.390 | 21.6 | NA |
| MANJ 074 | MDG11106271 | NA | bovine | F | 8 | 0 | NA | NA | NA | NA | -1.187 | 1.290 | -0.181 | -0.390 | 21.6 | NA |
| MANJ 042 | MDG11106271 | NA | bovine | M | 4 | 1 | NA | NA | NA | NA | -1.187 | 1.290 | -0.181 | -0.390 | 21.6 | NA |
| MANJ 066 | MDG11106271 | NA | bovine | M | 6 | 0 | NA | NA | NA | NA | -1.187 | 1.290 | -0.181 | -0.390 | 21.6 | NA |
| MANJ 040 | MDG11106271 | NA | bovine | M | 5 | 0 | NA | NA | NA | NA | -1.187 | 1.290 | -0.181 | -0.390 | 21.6 | NA |
| MANJ 039 | MDG11106271 | NA | bovine | M | 6 | 0 | NA | NA | NA | NA | -1.187 | 1.290 | -0.181 | -0.390 | 21.6 | NA |
| MANJ 038 | MDG11106271 | NA | bovine | M | 5 | 0 | NA | NA | NA | NA | -1.187 | 1.290 | -0.181 | -0.390 | 21.6 | NA |
| MANJ 037 | MDG11106271 | NA | bovine | M | 5 | 0 | NA | NA | NA | NA | -1.187 | 1.290 | -0.181 | -0.390 | 21.6 | NA |
| MANJ 036 | MDG11106271 | NA | bovine | M | 6 | 0 | NA | NA | NA | NA | -1.187 | 1.290 | -0.181 | -0.390 | 21.6 | NA |

| ID | PCODE | site2 | sp | gender | age | IgG | contact_ruminant | contact_milk | contact_fresh_fluid | profession | fact1 | fact2 | fact3 | fact4 | cattle_density | habitat |
| --- | --- | --- | --- | --- | --- | --- | --- | --- | --- | --- | --- | --- | --- | --- | --- | --- |
| MANJ 075 | MDG11106271 | NA | bovine | M | 5 | 0 | NA | NA | NA | NA | -1.187 | 1.290 | -0.181 | -0.390 | 21.6 | NA |
| MANJ 063 | MDG11106272 | NA | bovine | M | 4 | 1 | NA | NA | NA | NA | -1.578 | 1.112 | -0.181 | -0.227 | 27.8 | NA |
| MANJ 071 | MDG11106272 | NA | bovine | F | 4 | 0 | NA | NA | NA | NA | -1.578 | 1.112 | -0.181 | -0.227 | 27.8 | NA |
| MANJ 056 | MDG11106371 | NA | bovine | M | 4 | 1 | NA | NA | NA | NA | -0.800 | 1.312 | -0.010 | -0.245 | 23 | NA |
| MIA 017 | MDG13112010 | NA | bovine | M | 6 | 0 | NA | NA | NA | NA | -0.154 | 1.362 | 0.210 | -0.297 | 28 | NA |
| MIA 015 | MDG13112010 | NA | bovine | F | 6 | 0 | NA | NA | NA | NA | -0.154 | 1.362 | 0.210 | -0.297 | 28 | NA |
| MIA 082 | MDG13112010 | NA | bovine | M | 5 | 0 | NA | NA | NA | NA | -0.154 | 1.362 | 0.210 | -0.297 | 28 | NA |
| MIA 014 | MDG13112010 | NA | bovine | F | 2 | 0 | NA | NA | NA | NA | -0.154 | 1.362 | 0.210 | -0.297 | 28 | NA |
| MIA 013 | MDG13112010 | NA | bovine | F | 6 | 1 | NA | NA | NA | NA | -0.154 | 1.362 | 0.210 | -0.297 | 28 | NA |
| MIA 080 | MDG13112010 | NA | bovine | M | 6 | 1 | NA | NA | NA | NA | -0.154 | 1.362 | 0.210 | -0.297 | 28 | NA |
| MIA 001 | MDG13112010 | NA | bovine | M | 6 | 0 | NA | NA | NA | NA | -0.154 | 1.362 | 0.210 | -0.297 | 28 | NA |
| MIA 079 | MDG13112010 | NA | bovine | F | 5 | 0 | NA | NA | NA | NA | -0.154 | 1.362 | 0.210 | -0.297 | 28 | NA |
| MIA 081 | MDG13112010 | NA | bovine | M | 6 | 0 | NA | NA | NA | NA | -0.154 | 1.362 | 0.210 | -0.297 | 28 | NA |
| MIA 023 | MDG13112030 | NA | bovine | M | 3 | 1 | NA | NA | NA | NA | 0.022 | 1.297 | 0.104 | -0.261 | 26.3333333333333 | NA |
| MIA 003 | MDG13112030 | NA | bovine | M | 8 | 0 | NA | NA | NA | NA | 0.022 | 1.297 | 0.104 | -0.261 | 26.3333333333333 | NA |
| MIA 031 | MDG13112030 | NA | bovine | M | 2 | 1 | NA | NA | NA | NA | 0.022 | 1.297 | 0.104 | -0.261 | 26.3333333333333 | NA |
| MIA 025 | MDG13112030 | NA | bovine | M | 9 | 0 | NA | NA | NA | NA | 0.022 | 1.297 | 0.104 | -0.261 | 26.3333333333333 | NA |
| MIA 022 | MDG13112030 | NA | bovine | F | 4 | 0 | NA | NA | NA | NA | 0.022 | 1.297 | 0.104 | -0.261 | 26.3333333333333 | NA |
| MIA 021 | MDG13112030 | NA | bovine | F | 3 | 0 | NA | NA | NA | NA | 0.022 | 1.297 | 0.104 | -0.261 | 26.3333333333333 | NA |
| MIA 005 | MDG13112030 | NA | bovine | M | 8 | 1 | NA | NA | NA | NA | 0.022 | 1.297 | 0.104 | -0.261 | 26.3333333333333 | NA |
| MIA 011 | MDG13112030 | NA | bovine | M | 7 | 0 | NA | NA | NA | NA | 0.022 | 1.297 | 0.104 | -0.261 | 26.3333333333333 | NA |
| MIA 010 | MDG13112030 | NA | bovine | F | 6 | 0 | NA | NA | NA | NA | 0.022 | 1.297 | 0.104 | -0.261 | 26.3333333333333 | NA |
| MIA 006 | MDG13112030 | NA | bovine | M | 6 | 0 | NA | NA | NA | NA | 0.022 | 1.297 | 0.104 | -0.261 | 26.3333333333333 | NA |
| MIA 004 | MDG13112030 | NA | bovine | M | 8 | 0 | NA | NA | NA | NA | 0.022 | 1.297 | 0.104 | -0.261 | 26.3333333333333 | NA |
| MIA 028 | MDG13112030 | NA | bovine | F | 10 | 1 | NA | NA | NA | NA | 0.022 | 1.297 | 0.104 | -0.261 | 26.3333333333333 | NA |
| MIA 049 | MDG13112090 | NA | bovine | M | 9 | 0 | NA | NA | NA | NA | -0.210 | 1.384 | -0.476 | -0.342 | 30.2222222222222 | NA |
| MIA 050 | MDG13112090 | NA | bovine | F | 5 | 0 | NA | NA | NA | NA | -0.210 | 1.384 | -0.476 | -0.342 | 30.2222222222222 | NA |
| MIA 051 | MDG13112090 | NA | bovine | M | 11 | 0 | NA | NA | NA | NA | -0.210 | 1.384 | -0.476 | -0.342 | 30.2222222222222 | NA |
| MIA 052 | MDG13112090 | NA | bovine | M | 11 | 1 | NA | NA | NA | NA | -0.210 | 1.384 | -0.476 | -0.342 | 30.2222222222222 | NA |
| MIA 053 | MDG13112090 | NA | bovine | M | 7 | 1 | NA | NA | NA | NA | -0.210 | 1.384 | -0.476 | -0.342 | 30.2222222222222 | NA |
| MIA 055 | MDG13112090 | NA | bovine | M | 2 | 0 | NA | NA | NA | NA | -0.210 | 1.384 | -0.476 | -0.342 | 30.2222222222222 | NA |
| MIA 056 | MDG13112090 | NA | bovine | F | 5 | 0 | NA | NA | NA | NA | -0.210 | 1.384 | -0.476 | -0.342 | 30.2222222222222 | NA |
| MIA 048 | MDG13112090 | NA | bovine | F | 12 | 0 | NA | NA | NA | NA | -0.210 | 1.384 | -0.476 | -0.342 | 30.2222222222222 | NA |
| MIA 036 | MDG13112090 | NA | bovine | M | 7 | 0 | NA | NA | NA | NA | -0.210 | 1.384 | -0.476 | -0.342 | 30.2222222222222 | NA |
| MIA 057 | MDG13112090 | NA | bovine | M | 3 | 0 | NA | NA | NA | NA | -0.210 | 1.384 | -0.476 | -0.342 | 30.2222222222222 | NA |
| MIA 054 | MDG13112090 | NA | bovine | M | 8 | 0 | NA | NA | NA | NA | -0.210 | 1.384 | -0.476 | -0.342 | 30.2222222222222 | NA |
| MIA 047 | MDG13112090 | NA | bovine | M | 9 | 1 | NA | NA | NA | NA | -0.210 | 1.384 | -0.476 | -0.342 | 30.2222222222222 | NA |
| MIA 046 | MDG13112090 | NA | bovine | M | 5 | 0 | NA | NA | NA | NA | -0.210 | 1.384 | -0.476 | -0.342 | 30.2222222222222 | NA |
| MIA 045 | MDG13112090 | NA | bovine | M | 7 | 1 | NA | NA | NA | NA | -0.210 | 1.384 | -0.476 | -0.342 | 30.2222222222222 | NA |
| MIA 044 | MDG13112090 | NA | bovine | M | 7 | 0 | NA | NA | NA | NA | -0.210 | 1.384 | -0.476 | -0.342 | 30.2222222222222 | NA |
| MIA 043 | MDG13112090 | NA | bovine | M | 8 | 0 | NA | NA | NA | NA | -0.210 | 1.384 | -0.476 | -0.342 | 30.2222222222222 | NA |
| MIA 042 | MDG13112090 | NA | bovine | M | 9 | 0 | NA | NA | NA | NA | -0.210 | 1.384 | -0.476 | -0.342 | 30.2222222222222 | NA |
| MIA 037 | MDG13112090 | NA | bovine | M | 7 | 1 | NA | NA | NA | NA | -0.210 | 1.384 | -0.476 | -0.342 | 30.2222222222222 | NA |
| MIA 035 | MDG13112090 | NA | bovine | M | 8 | 1 | NA | NA | NA | NA | -0.210 | 1.384 | -0.476 | -0.342 | 30.2222222222222 | NA |
| MIA 034 | MDG13112090 | NA | bovine | M | 4 | 0 | NA | NA | NA | NA | -0.210 | 1.384 | -0.476 | -0.342 | 30.2222222222222 | NA |
| MIA 072 | MDG13112090 | NA | bovine | M | 3 | 0 | NA | NA | NA | NA | -0.210 | 1.384 | -0.476 | -0.342 | 30.2222222222222 | NA |
| MIA 058 | MDG13112090 | NA | bovine | M | 6 | 0 | NA | NA | NA | NA | -0.210 | 1.384 | -0.476 | -0.342 | 30.2222222222222 | NA |
| MIA 041 | MDG13112090 | NA | bovine | M | 5 | 0 | NA | NA | NA | NA | -0.210 | 1.384 | -0.476 | -0.342 | 30.2222222222222 | NA |
| MIA 069 | MDG13112090 | NA | bovine | F | 7 | 0 | NA | NA | NA | NA | -0.210 | 1.384 | -0.476 | -0.342 | 30.2222222222222 | NA |
| MIA 060 | MDG13112090 | NA | bovine | M | 9 | 0 | NA | NA | NA | NA | -0.210 | 1.384 | -0.476 | -0.342 | 30.2222222222222 | NA |
| MIA 061 | MDG13112090 | NA | bovine | M | 7 | 0 | NA | NA | NA | NA | -0.210 | 1.384 | -0.476 | -0.342 | 30.2222222222222 | NA |
| MIA 062 | MDG13112090 | NA | bovine | M | 4 | 0 | NA | NA | NA | NA | -0.210 | 1.384 | -0.476 | -0.342 | 30.2222222222222 | NA |
| MIA 063 | MDG13112090 | NA | bovine | M | 5 | 0 | NA | NA | NA | NA | -0.210 | 1.384 | -0.476 | -0.342 | 30.2222222222222 | NA |
| MIA 065 | MDG13112090 | NA | bovine | M | 5 | 0 | NA | NA | NA | NA | -0.210 | 1.384 | -0.476 | -0.342 | 30.2222222222222 | NA |
| MIA 070 | MDG13112090 | NA | bovine | F | 3 | 0 | NA | NA | NA | NA | -0.210 | 1.384 | -0.476 | -0.342 | 30.2222222222222 | NA |
| MIA 059 | MDG13112090 | NA | bovine | M | 5 | 0 | NA | NA | NA | NA | -0.210 | 1.384 | -0.476 | -0.342 | 30.2222222222222 | NA |
| MIA 068 | MDG13112090 | NA | bovine | F | 9 | 0 | NA | NA | NA | NA | -0.210 | 1.384 | -0.476 | -0.342 | 30.2222222222222 | NA |
| MIA 064 | MDG13112090 | NA | bovine | M | 8 | 0 | NA | NA | NA | NA | -0.210 | 1.384 | -0.476 | -0.342 | 30.2222222222222 | NA |
| MIA 071 | MDG13112090 | NA | bovine | F | 8 | 1 | NA | NA | NA | NA | -0.210 | 1.384 | -0.476 | -0.342 | 30.2222222222222 | NA |
| MIA 073 | MDG13112090 | NA | bovine | M | 7 | 0 | NA | NA | NA | NA | -0.210 | 1.384 | -0.476 | -0.342 | 30.2222222222222 | NA |
| MIA 074 | MDG13112090 | NA | bovine | M | 7 | 0 | NA | NA | NA | NA | -0.210 | 1.384 | -0.476 | -0.342 | 30.2222222222222 | NA |
| MIA 075 | MDG13112090 | NA | bovine | M | 6 | 1 | NA | NA | NA | NA | -0.210 | 1.384 | -0.476 | -0.342 | 30.2222222222222 | NA |
| MIA 076 | MDG13112090 | NA | bovine | F | 8 | 1 | NA | NA | NA | NA | -0.210 | 1.384 | -0.476 | -0.342 | 30.2222222222222 | NA |

| ID | PCODE | site2 | sp | gender | age | IgG | contact_ruminant | contact_milk | contact_fresh_fluid | profession | fact1 | fact2 | fact3 | fact4 | cattle_density | habitat |
| --- | --- | --- | --- | --- | --- | --- | --- | --- | --- | --- | --- | --- | --- | --- | --- | --- |
| MIA 077 | MDG13112090 | NA | bovine | F | 7 | 0 | NA | NA | NA | NA | -0.210 | 1.384 | -0.476 | -0.342 | 30.2222222222222 | NA |
| MIA 078 | MDG13112090 | NA | bovine | F | 5 | 0 | NA | NA | NA | NA | -0.210 | 1.384 | -0.476 | -0.342 | 30.2222222222222 | NA |
| MIA 067 | MDG13112090 | NA | bovine | M | 9 | 0 | NA | NA | NA | NA | -0.210 | 1.384 | -0.476 | -0.342 | 30.2222222222222 | NA |
| MIA 066 | MDG13112090 | NA | bovine | F | 8 | 0 | NA | NA | NA | NA | -0.210 | 1.384 | -0.476 | -0.342 | 30.2222222222222 | NA |
| MIA 020 | MDG13112111 | NA | bovine | M | 8 | 0 | NA | NA | NA | NA | 0.535 | 1.036 | 0.239 | -0.049 | 19.1999999999999 | NA |
| MIA 040 | MDG13112111 | NA | bovine | M | 7 | 1 | NA | NA | NA | NA | 0.535 | 1.036 | 0.239 | -0.049 | 19.1999999999999 | NA |
| MIA 038 | MDG13112111 | NA | bovine | M | 8 | 1 | NA | NA | NA | NA | 0.535 | 1.036 | 0.239 | -0.049 | 19.1999999999999 | NA |
| MIA 032 | MDG13112111 | NA | bovine | M | 7 | 1 | NA | NA | NA | NA | 0.535 | 1.036 | 0.239 | -0.049 | 19.1999999999999 | NA |
| MIA 033 | MDG13112111 | NA | bovine | M | 7 | 0 | NA | NA | NA | NA | 0.535 | 1.036 | 0.239 | -0.049 | 19.1999999999999 | NA |
| MIA 008 | MDG13112112 | NA | bovine | M | 7 | 0 | NA | NA | NA | NA | 0.433 | 0.783 | 0.525 | 0.342 | 14.625 | NA |
| FEN_038 | MDG14119010 | NA | bovine | M | 4 | 0 | NA | NA | NA | NA | 0.513 | 0.756 | 1.261 | -0.208 | 9.6590909090909 | NA |
| FEN_041 | MDG14119010 | NA | bovine | M | 7 | 0 | NA | NA | NA | NA | 0.513 | 0.756 | 1.261 | -0.208 | 9.6590909090909 | NA |
| FEN_042 | MDG14119010 | NA | bovine | F | 4 | 1 | NA | NA | NA | NA | 0.513 | 0.756 | 1.261 | -0.208 | 9.6590909090909 | NA |
| FEN_039 | MDG14119010 | NA | bovine | M | 3 | 0 | NA | NA | NA | NA | 0.513 | 0.756 | 1.261 | -0.208 | 9.6590909090909 | NA |
| FEN_046 | MDG14119010 | NA | bovine | F | 2 | 0 | NA | NA | NA | NA | 0.513 | 0.756 | 1.261 | -0.208 | 9.6590909090909 | NA |
| FEN_003 | MDG14119010 | NA | bovine | F | 6 | 0 | NA | NA | NA | NA | 0.513 | 0.756 | 1.261 | -0.208 | 9.6590909090909 | NA |
| FEN_002 | MDG14119010 | NA | bovine | M | 9 | 0 | NA | NA | NA | NA | 0.513 | 0.756 | 1.261 | -0.208 | 9.6590909090909 | NA |
| FEN_001 | MDG14119010 | NA | bovine | M | 6 | 1 | NA | NA | NA | NA | 0.513 | 0.756 | 1.261 | -0.208 | 9.6590909090909 | NA |
| FEN_040 | MDG14119010 | NA | bovine | M | 7 | 1 | NA | NA | NA | NA | 0.513 | 0.756 | 1.261 | -0.208 | 9.6590909090909 | NA |
| FEN_043 | MDG14119010 | NA | bovine | M | 6 | 1 | NA | NA | NA | NA | 0.513 | 0.756 | 1.261 | -0.208 | 9.6590909090909 | NA |
| FEN_005 | MDG14119010 | NA | bovine | M | 11 | 0 | NA | NA | NA | NA | 0.513 | 0.756 | 1.261 | -0.208 | 9.6590909090909 | NA |
| FEN_045 | MDG14119010 | NA | bovine | M | 3 | 0 | NA | NA | NA | NA | 0.513 | 0.756 | 1.261 | -0.208 | 9.6590909090909 | NA |
| FEN_047 | MDG14119010 | NA | bovine | M | 5 | 0 | NA | NA | NA | NA | 0.513 | 0.756 | 1.261 | -0.208 | 9.6590909090909 | NA |
| FEN_048 | MDG14119010 | NA | bovine | M | 5 | 0 | NA | NA | NA | NA | 0.513 | 0.756 | 1.261 | -0.208 | 9.6590909090909 | NA |
| FEN_049 | MDG14119010 | NA | bovine | M | 8 | 0 | NA | NA | NA | NA | 0.513 | 0.756 | 1.261 | -0.208 | 9.6590909090909 | NA |
| FEN_050 | MDG14119010 | NA | bovine | M | 8 | 0 | NA | NA | NA | NA | 0.513 | 0.756 | 1.261 | -0.208 | 9.6590909090909 | NA |
| FEN_051 | MDG14119010 | NA | bovine | F | 5 | 1 | NA | NA | NA | NA | 0.513 | 0.756 | 1.261 | -0.208 | 9.6590909090909 | NA |
| FEN_052 | MDG14119010 | NA | bovine | M | 4 | 1 | NA | NA | NA | NA | 0.513 | 0.756 | 1.261 | -0.208 | 9.6590909090909 | NA |
| FEN_053 | MDG14119010 | NA | bovine | F | 5 | 0 | NA | NA | NA | NA | 0.513 | 0.756 | 1.261 | -0.208 | 9.6590909090909 | NA |
| FEN_054 | MDG14119010 | NA | bovine | M | 9 | 1 | NA | NA | NA | NA | 0.513 | 0.756 | 1.261 | -0.208 | 9.6590909090909 | NA |
| FEN_044 | MDG14119010 | NA | bovine | M | 5 | 0 | NA | NA | NA | NA | 0.513 | 0.756 | 1.261 | -0.208 | 9.6590909090909 | NA |
| FEN_004 | MDG14119010 | NA | bovine | M | 12 | 0 | NA | NA | NA | NA | 0.513 | 0.756 | 1.261 | -0.208 | 9.6590909090909 | NA |
| FEN_033 | MDG14119010 | NA | bovine | M | 5 | 0 | NA | NA | NA | NA | 0.513 | 0.756 | 1.261 | -0.208 | 9.6590909090909 | NA |
| FEN_034 | MDG14119010 | NA | bovine | M | 5 | 1 | NA | NA | NA | NA | 0.513 | 0.756 | 1.261 | -0.208 | 9.6590909090909 | NA |
| FEN_035 | MDG14119010 | NA | bovine | M | 8 | 1 | NA | NA | NA | NA | 0.513 | 0.756 | 1.261 | -0.208 | 9.6590909090909 | NA |
| FEN_036 | MDG14119010 | NA | bovine | F | 6 | 1 | NA | NA | NA | NA | 0.513 | 0.756 | 1.261 | -0.208 | 9.6590909090909 | NA |
| FEN_037 | MDG14119010 | NA | bovine | F | 1 | 1 | NA | NA | NA | NA | 0.513 | 0.756 | 1.261 | -0.208 | 9.6590909090909 | NA |
| FEN_031 | MDG14119010 | NA | bovine | F | 2 | 0 | NA | NA | NA | NA | 0.513 | 0.756 | 1.261 | -0.208 | 9.6590909090909 | NA |
| FEN_030 | MDG14119010 | NA | bovine | M | 5 | 0 | NA | NA | NA | NA | 0.513 | 0.756 | 1.261 | -0.208 | 9.6590909090909 | NA |
| FEN_029 | MDG14119010 | NA | bovine | M | 3 | 0 | NA | NA | NA | NA | 0.513 | 0.756 | 1.261 | -0.208 | 9.6590909090909 | NA |
| FEN_007 | MDG14119010 | NA | bovine | M | 5 | 0 | NA | NA | NA | NA | 0.513 | 0.756 | 1.261 | -0.208 | 9.6590909090909 | NA |
| FEN_026 | MDG14119010 | NA | bovine | M | 5 | 0 | NA | NA | NA | NA | 0.513 | 0.756 | 1.261 | -0.208 | 9.6590909090909 | NA |
| FEN_006 | MDG14119010 | NA | bovine | M | 9 | 0 | NA | NA | NA | NA | 0.513 | 0.756 | 1.261 | -0.208 | 9.6590909090909 | NA |
| FEN_025 | MDG14119010 | NA | bovine | M | 5 | 0 | NA | NA | NA | NA | 0.513 | 0.756 | 1.261 | -0.208 | 9.6590909090909 | NA |
| FEN_012 | MDG14119010 | NA | bovine | M | 7 | 0 | NA | NA | NA | NA | 0.513 | 0.756 | 1.261 | -0.208 | 9.6590909090909 | NA |
| FEN_011 | MDG14119010 | NA | bovine | M | 5 | 0 | NA | NA | NA | NA | 0.513 | 0.756 | 1.261 | -0.208 | 9.6590909090909 | NA |
| FEN_010 | MDG14119010 | NA | bovine | F | 5 | 0 | NA | NA | NA | NA | 0.513 | 0.756 | 1.261 | -0.208 | 9.6590909090909 | NA |
| FEN_009 | MDG14119010 | NA | bovine | F | 4 | 0 | NA | NA | NA | NA | 0.513 | 0.756 | 1.261 | -0.208 | 9.6590909090909 | NA |
| FEN_056 | MDG14119010 | NA | bovine | F | 2 | 0 | NA | NA | NA | NA | 0.513 | 0.756 | 1.261 | -0.208 | 9.6590909090909 | NA |
| FEN_008 | MDG14119010 | NA | bovine | F | 3 | 0 | NA | NA | NA | NA | 0.513 | 0.756 | 1.261 | -0.208 | 9.6590909090909 | NA |
| FEN_032 | MDG14119010 | NA | bovine | F | 4 | 0 | NA | NA | NA | NA | 0.513 | 0.756 | 1.261 | -0.208 | 9.6590909090909 | NA |
| FEN_089 | MDG14119010 | NA | bovine | M | 6 | 0 | NA | NA | NA | NA | 0.513 | 0.756 | 1.261 | -0.208 | 9.6590909090909 | NA |
| FEN_027 | MDG14119010 | NA | bovine | M | 8 | 0 | NA | NA | NA | NA | 0.513 | 0.756 | 1.261 | -0.208 | 9.6590909090909 | NA |
| FEN_100 | MDG14119010 | NA | bovine | M | 7 | 0 | NA | NA | NA | NA | 0.513 | 0.756 | 1.261 | -0.208 | 9.6590909090909 | NA |
| FEN_082 | MDG14119010 | NA | bovine | F | 5 | 0 | NA | NA | NA | NA | 0.513 | 0.756 | 1.261 | -0.208 | 9.6590909090909 | NA |
| FEN_084 | MDG14119010 | NA | bovine | M | 8 | 0 | NA | NA | NA | NA | 0.513 | 0.756 | 1.261 | -0.208 | 9.6590909090909 | NA |
| FEN_090 | MDG14119010 | NA | bovine | M | 6 | 0 | NA | NA | NA | NA | 0.513 | 0.756 | 1.261 | -0.208 | 9.6590909090909 | NA |
| FEN_091 | MDG14119010 | NA | bovine | M | 5 | 0 | NA | NA | NA | NA | 0.513 | 0.756 | 1.261 | -0.208 | 9.6590909090909 | NA |
| FEN_092 | MDG14119010 | NA | bovine | M | 6 | 0 | NA | NA | NA | NA | 0.513 | 0.756 | 1.261 | -0.208 | 9.6590909090909 | NA |
| FEN_093 | MDG14119010 | NA | bovine | M | 7 | 0 | NA | NA | NA | NA | 0.513 | 0.756 | 1.261 | -0.208 | 9.6590909090909 | NA |
| FEN_094 | MDG14119010 | NA | bovine | F | 6 | 0 | NA | NA | NA | NA | 0.513 | 0.756 | 1.261 | -0.208 | 9.6590909090909 | NA |
| FEN_095 | MDG14119010 | NA | bovine | F | 5 | 0 | NA | NA | NA | NA | 0.513 | 0.756 | 1.261 | -0.208 | 9.6590909090909 | NA |
| FEN_096 | MDG14119010 | NA | bovine | M | 9 | 0 | NA | NA | NA | NA | 0.513 | 0.756 | 1.261 | -0.208 | 9.6590909090909 | NA |

| ID | PCODE | site2 | sp | gender | age | IgG | contact_ruminant | contact_milk | contact_fresh_fluid | profession | fact1 | fact2 | fact3 | fact4 | cattle_density | habitat |
| --- | --- | --- | --- | --- | --- | --- | --- | --- | --- | --- | --- | --- | --- | --- | --- | --- |
| FEN_080 | MDG14119010 | NA | bovine | M | 7 | 0 | NA | NA | NA | NA | 0.513 | 0.756 | 1.261 | -0.208 | 9.6590909090909 | NA |
| FEN_098 | MDG14119010 | NA | bovine | F | 4 | 0 | NA | NA | NA | NA | 0.513 | 0.756 | 1.261 | -0.208 | 9.6590909090909 | NA |
| FEN_099 | MDG14119010 | NA | bovine | M | 7 | 0 | NA | NA | NA | NA | 0.513 | 0.756 | 1.261 | -0.208 | 9.6590909090909 | NA |
| FEN_083 | MDG14119010 | NA | bovine | M | 4 | 0 | NA | NA | NA | NA | 0.513 | 0.756 | 1.261 | -0.208 | 9.6590909090909 | NA |
| FEN_102 | MDG14119010 | NA | bovine | M | 8 | 0 | NA | NA | NA | NA | 0.513 | 0.756 | 1.261 | -0.208 | 9.6590909090909 | NA |
| FEN_103 | MDG14119010 | NA | bovine | F | 6 | 0 | NA | NA | NA | NA | 0.513 | 0.756 | 1.261 | -0.208 | 9.6590909090909 | NA |
| FEN_104 | MDG14119010 | NA | bovine | F | 7 | 0 | NA | NA | NA | NA | 0.513 | 0.756 | 1.261 | -0.208 | 9.6590909090909 | NA |
| FEN_106 | MDG14119010 | NA | bovine | M | 8 | 0 | NA | NA | NA | NA | 0.513 | 0.756 | 1.261 | -0.208 | 9.6590909090909 | NA |
| FEN_107 | MDG14119010 | NA | bovine | F | 4 | 0 | NA | NA | NA | NA | 0.513 | 0.756 | 1.261 | -0.208 | 9.6590909090909 | NA |
| FEN_108 | MDG14119010 | NA | bovine | F | 3 | 0 | NA | NA | NA | NA | 0.513 | 0.756 | 1.261 | -0.208 | 9.6590909090909 | NA |
| FEN_109 | MDG14119010 | NA | bovine | F | 5 | 0 | NA | NA | NA | NA | 0.513 | 0.756 | 1.261 | -0.208 | 9.6590909090909 | NA |
| FEN_110 | MDG14119010 | NA | bovine | M | 7 | 0 | NA | NA | NA | NA | 0.513 | 0.756 | 1.261 | -0.208 | 9.6590909090909 | NA |
| FEN_097 | MDG14119010 | NA | bovine | F | 5 | 0 | NA | NA | NA | NA | 0.513 | 0.756 | 1.261 | -0.208 | 9.6590909090909 | NA |
| FEN_069 | MDG14119010 | NA | bovine | F | 8 | 1 | NA | NA | NA | NA | 0.513 | 0.756 | 1.261 | -0.208 | 9.6590909090909 | NA |
| FEN_061 | MDG14119010 | NA | bovine | F | 4 | 1 | NA | NA | NA | NA | 0.513 | 0.756 | 1.261 | -0.208 | 9.6590909090909 | NA |
| FEN_062 | MDG14119010 | NA | bovine | F | 6 | 0 | NA | NA | NA | NA | 0.513 | 0.756 | 1.261 | -0.208 | 9.6590909090909 | NA |
| FEN_064 | MDG14119010 | NA | bovine | M | 5 | 0 | NA | NA | NA | NA | 0.513 | 0.756 | 1.261 | -0.208 | 9.6590909090909 | NA |
| FEN_079 | MDG14119010 | NA | bovine | M | 7 | 0 | NA | NA | NA | NA | 0.513 | 0.756 | 1.261 | -0.208 | 9.6590909090909 | NA |
| FEN_063 | MDG14119010 | NA | bovine | M | 5 | 1 | NA | NA | NA | NA | 0.513 | 0.756 | 1.261 | -0.208 | 9.6590909090909 | NA |
| FEN_078 | MDG14119010 | NA | bovine | M | 10 | 0 | NA | NA | NA | NA | 0.513 | 0.756 | 1.261 | -0.208 | 9.6590909090909 | NA |
| FEN_076 | MDG14119010 | NA | bovine | M | 6 | 0 | NA | NA | NA | NA | 0.513 | 0.756 | 1.261 | -0.208 | 9.6590909090909 | NA |
| FEN_075 | MDG14119010 | NA | bovine | M | 6 | 1 | NA | NA | NA | NA | 0.513 | 0.756 | 1.261 | -0.208 | 9.6590909090909 | NA |
| FEN_074 | MDG14119010 | NA | bovine | M | 3 | 0 | NA | NA | NA | NA | 0.513 | 0.756 | 1.261 | -0.208 | 9.6590909090909 | NA |
| FEN_073 | MDG14119010 | NA | bovine | F | 2 | 0 | NA | NA | NA | NA | 0.513 | 0.756 | 1.261 | -0.208 | 9.6590909090909 | NA |
| FEN_072 | MDG14119010 | NA | bovine | F | 4 | 0 | NA | NA | NA | NA | 0.513 | 0.756 | 1.261 | -0.208 | 9.6590909090909 | NA |
| FEN_101 | MDG14119010 | NA | bovine | M | 6 | 0 | NA | NA | NA | NA | 0.513 | 0.756 | 1.261 | -0.208 | 9.6590909090909 | NA |
| FEN_070 | MDG14119010 | NA | bovine | F | 7 | 1 | NA | NA | NA | NA | 0.513 | 0.756 | 1.261 | -0.208 | 9.6590909090909 | NA |
| FEN_068 | MDG14119010 | NA | bovine | F | 5 | 0 | NA | NA | NA | NA | 0.513 | 0.756 | 1.261 | -0.208 | 9.6590909090909 | NA |
| FEN_066 | MDG14119010 | NA | bovine | M | 10 | 1 | NA | NA | NA | NA | 0.513 | 0.756 | 1.261 | -0.208 | 9.6590909090909 | NA |
| FEN_067 | MDG14119010 | NA | bovine | M | 3 | 0 | NA | NA | NA | NA | 0.513 | 0.756 | 1.261 | -0.208 | 9.6590909090909 | NA |
| FEN_071 | MDG14119010 | NA | bovine | M | 5 | 1 | NA | NA | NA | NA | 0.513 | 0.756 | 1.261 | -0.208 | 9.6590909090909 | NA |
| FEN_059 | MDG14119031 | NA | bovine | M | 5 | 0 | NA | NA | NA | NA | 0.239 | 1.355 | 0.305 | -0.302 | 11.9565217391304 | NA |
| FEN_060 | MDG14119031 | NA | bovine | M | 6 | 1 | NA | NA | NA | NA | 0.239 | 1.355 | 0.305 | -0.302 | 11.9565217391304 | NA |
| FEN_055 | MDG14119031 | NA | bovine | M | 7 | 0 | NA | NA | NA | NA | 0.239 | 1.355 | 0.305 | -0.302 | 11.9565217391304 | NA |
| FEN_057 | MDG14119031 | NA | bovine | F | 3 | 0 | NA | NA | NA | NA | 0.239 | 1.355 | 0.305 | -0.302 | 11.9565217391304 | NA |
| MB 058 | MDG22203010 | NA | bovine | F | 1 | 0 | NA | NA | NA | NA | -0.394 | 1.047 | -0.105 | -0.112 | 4.5 | NA |
| MB 064 | MDG22203030 | NA | bovine | F | 6 | 0 | NA | NA | NA | NA | -0.780 | 1.146 | 0.488 | -0.204 | 4.4 | NA |
| MB 065 | MDG22203030 | NA | bovine | M | 3 | 0 | NA | NA | NA | NA | -0.780 | 1.146 | 0.488 | -0.204 | 4.4 | NA |
| MB 063 | MDG22203030 | NA | bovine | M | 2 | 0 | NA | NA | NA | NA | -0.780 | 1.146 | 0.488 | -0.204 | 4.4 | NA |
| MB 067 | MDG22203030 | NA | bovine | F | 6 | 0 | NA | NA | NA | NA | -0.780 | 1.146 | 0.488 | -0.204 | 4.4 | NA |
| MB 061 | MDG22203030 | NA | bovine | F | 5 | 0 | NA | NA | NA | NA | -0.780 | 1.146 | 0.488 | -0.204 | 4.4 | NA |
| MB 068 | MDG22203030 | NA | bovine | F | 1 | 0 | NA | NA | NA | NA | -0.780 | 1.146 | 0.488 | -0.204 | 4.4 | NA |
| MB 069 | MDG22203030 | NA | bovine | F | 6 | 0 | NA | NA | NA | NA | -0.780 | 1.146 | 0.488 | -0.204 | 4.4 | NA |
| MB 070 | MDG22203030 | NA | bovine | M | 3 | 1 | NA | NA | NA | NA | -0.780 | 1.146 | 0.488 | -0.204 | 4.4 | NA |
| MB 066 | MDG22203030 | NA | bovine | M | 1 | 0 | NA | NA | NA | NA | -0.780 | 1.146 | 0.488 | -0.204 | 4.4 | NA |
| MB 072 | MDG22203030 | NA | bovine | M | 2 | 0 | NA | NA | NA | NA | -0.780 | 1.146 | 0.488 | -0.204 | 4.4 | NA |
| MB 074 | MDG22203030 | NA | bovine | F | 2 | 0 | NA | NA | NA | NA | -0.780 | 1.146 | 0.488 | -0.204 | 4.4 | NA |
| MB 081 | MDG22203050 | NA | bovine | M | 3 | 0 | NA | NA | NA | NA | -1.002 | 1.376 | 0.203 | -0.331 | 4 | NA |
| MB 077 | MDG22203050 | NA | bovine | F | 3 | 0 | NA | NA | NA | NA | -1.002 | 1.376 | 0.203 | -0.331 | 4 | NA |
| MB 079 | MDG22203050 | NA | bovine | F | 2 | 0 | NA | NA | NA | NA | -1.002 | 1.376 | 0.203 | -0.331 | 4 | NA |
| MB 080 | MDG22203050 | NA | bovine | F | 6 | 0 | NA | NA | NA | NA | -1.002 | 1.376 | 0.203 | -0.331 | 4 | NA |
| MB 082 | MDG22203050 | NA | bovine | F | 8 | 0 | NA | NA | NA | NA | -1.002 | 1.376 | 0.203 | -0.331 | 4 | NA |
| MB 033 | MDG22203070 | NA | bovine | F | 7 | 0 | NA | NA | NA | NA | -0.679 | 1.387 | 0.523 | -0.310 | 2.5 | NA |
| MB 038 | MDG22203070 | NA | bovine | F | 6 | 0 | NA | NA | NA | NA | -0.679 | 1.387 | 0.523 | -0.310 | 2.5 | NA |
| MB 026 | MDG22203070 | NA | bovine | F | 5 | 0 | NA | NA | NA | NA | -0.679 | 1.387 | 0.523 | -0.310 | 2.5 | NA |
| MB 029 | MDG22203070 | NA | bovine | F | 6 | 0 | NA | NA | NA | NA | -0.679 | 1.387 | 0.523 | -0.310 | 2.5 | NA |
| MB 030 | MDG22203070 | NA | bovine | M | 3 | 0 | NA | NA | NA | NA | -0.679 | 1.387 | 0.523 | -0.310 | 2.5 | NA |
| MB 031 | MDG22203070 | NA | bovine | M | 5 | 0 | NA | NA | NA | NA | -0.679 | 1.387 | 0.523 | -0.310 | 2.5 | NA |
| MB 025 | MDG22203070 | NA | bovine | F | 6 | 0 | NA | NA | NA | NA | -0.679 | 1.387 | 0.523 | -0.310 | 2.5 | NA |
| MB 032 | MDG22203070 | NA | bovine | F | 7 | 0 | NA | NA | NA | NA | -0.679 | 1.387 | 0.523 | -0.310 | 2.5 | NA |
| MB 028 | MDG22203070 | NA | bovine | M | 3 | 0 | NA | NA | NA | NA | -0.679 | 1.387 | 0.523 | -0.310 | 2.5 | NA |
| MB 027 | MDG22203070 | NA | bovine | M | 2 | 0 | NA | NA | NA | NA | -0.679 | 1.387 | 0.523 | -0.310 | 2.5 | NA |
| MB 045 | MDG22203070 | NA | bovine | F | 3 | 0 | NA | NA | NA | NA | -0.679 | 1.387 | 0.523 | -0.310 | 2.5 | NA |

| ID | PCODE | site2 | sp | gender | age | IgG | contact_ruminant | contact_milk | contact_fresh_fluid | profession | fact1 | fact2 | fact3 | fact4 | cattle_density | habitat |
| --- | --- | --- | --- | --- | --- | --- | --- | --- | --- | --- | --- | --- | --- | --- | --- | --- |
| MB 046 | MDG22203070 | NA | bovine | F | 3 | 0 | NA | NA | NA | NA | -0.679 | 1.387 | 0.523 | -0.310 | 2.5 | NA |
| MB 053 | MDG22203070 | NA | bovine | M | 5 | 0 | NA | NA | NA | NA | -0.679 | 1.387 | 0.523 | -0.310 | 2.5 | NA |
| MB 052 | MDG22203070 | NA | bovine | F | 4 | 0 | NA | NA | NA | NA | -0.679 | 1.387 | 0.523 | -0.310 | 2.5 | NA |
| MB 051 | MDG22203070 | NA | bovine | F | 3 | 0 | NA | NA | NA | NA | -0.679 | 1.387 | 0.523 | -0.310 | 2.5 | NA |
| MB 050 | MDG22203070 | NA | bovine | F | 6 | 0 | NA | NA | NA | NA | -0.679 | 1.387 | 0.523 | -0.310 | 2.5 | NA |
| MB 049 | MDG22203070 | NA | bovine | M | 6 | 0 | NA | NA | NA | NA | -0.679 | 1.387 | 0.523 | -0.310 | 2.5 | NA |
| MB 036 | MDG22203070 | NA | bovine | M | 6 | 0 | NA | NA | NA | NA | -0.679 | 1.387 | 0.523 | -0.310 | 2.5 | NA |
| MB 047 | MDG22203070 | NA | bovine | M | 3 | 0 | NA | NA | NA | NA | -0.679 | 1.387 | 0.523 | -0.310 | 2.5 | NA |
| MB 034 | MDG22203070 | NA | bovine | M | 5 | 0 | NA | NA | NA | NA | -0.679 | 1.387 | 0.523 | -0.310 | 2.5 | NA |
| MB 043 | MDG22203070 | NA | bovine | F | 5 | 0 | NA | NA | NA | NA | -0.679 | 1.387 | 0.523 | -0.310 | 2.5 | NA |
| MB 042 | MDG22203070 | NA | bovine | F | 5 | 0 | NA | NA | NA | NA | -0.679 | 1.387 | 0.523 | -0.310 | 2.5 | NA |
| MB 041 | MDG22203070 | NA | bovine | F | 2 | 0 | NA | NA | NA | NA | -0.679 | 1.387 | 0.523 | -0.310 | 2.5 | NA |
| MB 040 | MDG22203070 | NA | bovine | M | 5 | 0 | NA | NA | NA | NA | -0.679 | 1.387 | 0.523 | -0.310 | 2.5 | NA |
| MB 039 | MDG22203070 | NA | bovine | M | 4 | 0 | NA | NA | NA | NA | -0.679 | 1.387 | 0.523 | -0.310 | 2.5 | NA |
| MB 037 | MDG22203070 | NA | bovine | M | 7 | 0 | NA | NA | NA | NA | -0.679 | 1.387 | 0.523 | -0.310 | 2.5 | NA |
| MB 035 | MDG22203070 | NA | bovine | F | 4 | 0 | NA | NA | NA | NA | -0.679 | 1.387 | 0.523 | -0.310 | 2.5 | NA |
| MB 048 | MDG22203070 | NA | bovine | F | 6 | 0 | NA | NA | NA | NA | -0.679 | 1.387 | 0.523 | -0.310 | 2.5 | NA |
| MB 044 | MDG22203070 | NA | bovine | M | 6 | 0 | NA | NA | NA | NA | -0.679 | 1.387 | 0.523 | -0.310 | 2.5 | NA |
| MB 015 | MDG22203090 | NA | bovine | M | 6 | 0 | NA | NA | NA | NA | -0.307 | 1.345 | 0.548 | -0.282 | 5.33333333333333 | NA |
| MB 022 | MDG22203090 | NA | bovine | F | 4 | 0 | NA | NA | NA | NA | -0.307 | 1.345 | 0.548 | -0.282 | 5.33333333333333 | NA |
| MB 021 | MDG22203090 | NA | bovine | F | 4 | 0 | NA | NA | NA | NA | -0.307 | 1.345 | 0.548 | -0.282 | 5.33333333333333 | NA |
| MB 020 | MDG22203090 | NA | bovine | F | 6 | 1 | NA | NA | NA | NA | -0.307 | 1.345 | 0.548 | -0.282 | 5.33333333333333 | NA |
| MB 019 | MDG22203090 | NA | bovine | M | 6 | 0 | NA | NA | NA | NA | -0.307 | 1.345 | 0.548 | -0.282 | 5.33333333333333 | NA |
| MB 018 | MDG22203090 | NA | bovine | F | 6 | 0 | NA | NA | NA | NA | -0.307 | 1.345 | 0.548 | -0.282 | 5.33333333333333 | NA |
| MB 016 | MDG22203090 | NA | bovine | F | 6 | 0 | NA | NA | NA | NA | -0.307 | 1.345 | 0.548 | -0.282 | 5.33333333333333 | NA |
| MB 014 | MDG22203090 | NA | bovine | M | 6 | 0 | NA | NA | NA | NA | -0.307 | 1.345 | 0.548 | -0.282 | 5.33333333333333 | NA |
| MB 013 | MDG22203090 | NA | bovine | F | 9 | 0 | NA | NA | NA | NA | -0.307 | 1.345 | 0.548 | -0.282 | 5.33333333333333 | NA |
| MB 012 | MDG22203090 | NA | bovine | F | 3 | 0 | NA | NA | NA | NA | -0.307 | 1.345 | 0.548 | -0.282 | 5.33333333333333 | NA |
| MB 003 | MDG22203090 | NA | bovine | F | 3 | 0 | NA | NA | NA | NA | -0.307 | 1.345 | 0.548 | -0.282 | 5.33333333333333 | NA |
| MB 017 | MDG22203090 | NA | bovine | M | 3 | 0 | NA | NA | NA | NA | -0.307 | 1.345 | 0.548 | -0.282 | 5.33333333333333 | NA |
| MB 002 | MDG22203090 | NA | bovine | F | 5 | 0 | NA | NA | NA | NA | -0.307 | 1.345 | 0.548 | -0.282 | 5.33333333333333 | NA |
| MB 010 | MDG22203090 | NA | bovine | M | 2 | 0 | NA | NA | NA | NA | -0.307 | 1.345 | 0.548 | -0.282 | 5.33333333333333 | NA |
| MB 004 | MDG22203090 | NA | bovine | F | 2 | 0 | NA | NA | NA | NA | -0.307 | 1.345 | 0.548 | -0.282 | 5.33333333333333 | NA |
| MB 005 | MDG22203090 | NA | bovine | M | 4 | 0 | NA | NA | NA | NA | -0.307 | 1.345 | 0.548 | -0.282 | 5.33333333333333 | NA |
| MB 006 | MDG22203090 | NA | bovine | F | 3 | 0 | NA | NA | NA | NA | -0.307 | 1.345 | 0.548 | -0.282 | 5.33333333333333 | NA |
| MB 007 | MDG22203090 | NA | bovine | M | 5 | 0 | NA | NA | NA | NA | -0.307 | 1.345 | 0.548 | -0.282 | 5.33333333333333 | NA |
| MB 008 | MDG22203090 | NA | bovine | M | 5 | 0 | NA | NA | NA | NA | -0.307 | 1.345 | 0.548 | -0.282 | 5.33333333333333 | NA |
| MB 009 | MDG22203090 | NA | bovine | M | 3 | 0 | NA | NA | NA | NA | -0.307 | 1.345 | 0.548 | -0.282 | 5.33333333333333 | NA |
| MB 001 | MDG22203090 | NA | bovine | M | 4 | 0 | NA | NA | NA | NA | -0.307 | 1.345 | 0.548 | -0.282 | 5.33333333333333 | NA |
| MB 097 | MDG22203130 | NA | bovine | M | 3 | 0 | NA | NA | NA | NA | -0.717 | 1.490 | 0.435 | -0.349 | 2.79999999999999 | NA |
| MB 102 | MDG22203130 | NA | bovine | F | 6 | 0 | NA | NA | NA | NA | -0.717 | 1.490 | 0.435 | -0.349 | 2.79999999999999 | NA |
| MB 106 | MDG22203130 | NA | bovine | M | 4 | 0 | NA | NA | NA | NA | -0.717 | 1.490 | 0.435 | -0.349 | 2.79999999999999 | NA |
| MB 105 | MDG22203130 | NA | bovine | F | 3 | 0 | NA | NA | NA | NA | -0.717 | 1.490 | 0.435 | -0.349 | 2.79999999999999 | NA |
| MB 103 | MDG22203130 | NA | bovine | F | 6 | 0 | NA | NA | NA | NA | -0.717 | 1.490 | 0.435 | -0.349 | 2.79999999999999 | NA |
| MB 101 | MDG22203130 | NA | bovine | F | 5 | 1 | NA | NA | NA | NA | -0.717 | 1.490 | 0.435 | -0.349 | 2.79999999999999 | NA |
| MB 100 | MDG22203130 | NA | bovine | M | 3 | 0 | NA | NA | NA | NA | -0.717 | 1.490 | 0.435 | -0.349 | 2.79999999999999 | NA |
| MB 099 | MDG22203130 | NA | bovine | F | 5 | 0 | NA | NA | NA | NA | -0.717 | 1.490 | 0.435 | -0.349 | 2.79999999999999 | NA |
| MB 098 | MDG22203130 | NA | bovine | F | 2 | 1 | NA | NA | NA | NA | -0.717 | 1.490 | 0.435 | -0.349 | 2.79999999999999 | NA |
| MB 090 | MDG22203130 | NA | bovine | F | 7 | 1 | NA | NA | NA | NA | -0.717 | 1.490 | 0.435 | -0.349 | 2.79999999999999 | NA |
| MB 095 | MDG22203130 | NA | bovine | F | 1 | 0 | NA | NA | NA | NA | -0.717 | 1.490 | 0.435 | -0.349 | 2.79999999999999 | NA |
| MB 094 | MDG22203130 | NA | bovine | F | 3 | 0 | NA | NA | NA | NA | -0.717 | 1.490 | 0.435 | -0.349 | 2.79999999999999 | NA |
| MB 093 | MDG22203130 | NA | bovine | F | 2 | 1 | NA | NA | NA | NA | -0.717 | 1.490 | 0.435 | -0.349 | 2.79999999999999 | NA |
| MB 092 | MDG22203130 | NA | bovine | M | 4 | 0 | NA | NA | NA | NA | -0.717 | 1.490 | 0.435 | -0.349 | 2.79999999999999 | NA |
| MB 091 | MDG22203130 | NA | bovine | F | 4 | 0 | NA | NA | NA | NA | -0.717 | 1.490 | 0.435 | -0.349 | 2.79999999999999 | NA |
| MB 096 | MDG22203130 | NA | bovine | M | 3 | 0 | NA | NA | NA | NA | -0.717 | 1.490 | 0.435 | -0.349 | 2.79999999999999 | NA |
| MB 104 | MDG22203130 | NA | bovine | F | 3 | 0 | NA | NA | NA | NA | -0.717 | 1.490 | 0.435 | -0.349 | 2.79999999999999 | NA |
| MB 056 | MDG22203150 | NA | bovine | F | 6 | 0 | NA | NA | NA | NA | -0.369 | 1.309 | 0.358 | -0.268 | 13.6 | NA |
| MB 057 | MDG22203150 | NA | bovine | M | 4 | 0 | NA | NA | NA | NA | -0.369 | 1.309 | 0.358 | -0.268 | 13.6 | NA |
| LAVA_041 | MDG21205010 | NA | bovine | M | 3 | 0 | NA | NA | NA | NA | 0.805 | 0.373 | -0.037 | -0.073 | 14.4444444444444 | NA |
| LAVA_002 | MDG21205010 | NA | bovine | M | 7 | 0 | NA | NA | NA | NA | 0.805 | 0.373 | -0.037 | -0.073 | 14.4444444444444 | NA |
| LAVA_012 | MDG21205010 | NA | bovine | M | 4 | 0 | NA | NA | NA | NA | 0.805 | 0.373 | -0.037 | -0.073 | 14.4444444444444 | NA |
| LAVA_013 | MDG21205010 | NA | bovine | M | 3 | 0 | NA | NA | NA | NA | 0.805 | 0.373 | -0.037 | -0.073 | 14.4444444444444 | NA |
| LAVA_014 | MDG21205010 | NA | bovine | F | 2 | 0 | NA | NA | NA | NA | 0.805 | 0.373 | -0.037 | -0.073 | 14.4444444444444 | NA |

| ID | PCODE | site2 | sp | gender | age | IgG | contact_ruminant | contact_milk | contact_fresh_fluid | profession | fact1 | fact2 | fact3 | fact4 | cattle_density | habitat |
| --- | --- | --- | --- | --- | --- | --- | --- | --- | --- | --- | --- | --- | --- | --- | --- | --- |
| LAVA_015 | MDG21205010 | NA | bovine | M | 6 | 0 | NA | NA | NA | NA | 0.805 | 0.373 | -0.037 | -0.073 | 14.4444444444444 | NA |
| LAVA_017 | MDG21205010 | NA | bovine | F | 3 | 0 | NA | NA | NA | NA | 0.805 | 0.373 | -0.037 | -0.073 | 14.4444444444444 | NA |
| LAVA_040 | MDG21205010 | NA | bovine | F | 2 | 0 | NA | NA | NA | NA | 0.805 | 0.373 | -0.037 | -0.073 | 14.4444444444444 | NA |
| LAVA_039 | MDG21205010 | NA | bovine | M | 4 | 0 | NA | NA | NA | NA | 0.805 | 0.373 | -0.037 | -0.073 | 14.4444444444444 | NA |
| LAVA_018 | MDG21205010 | NA | bovine | M | 9 | 0 | NA | NA | NA | NA | 0.805 | 0.373 | -0.037 | -0.073 | 14.4444444444444 | NA |
| LAVA_019 | MDG21205010 | NA | bovine | M | 3 | 1 | NA | NA | NA | NA | 0.805 | 0.373 | -0.037 | -0.073 | 14.4444444444444 | NA |
| LAVA_020 | MDG21205010 | NA | bovine | F | 1 | 0 | NA | NA | NA | NA | 0.805 | 0.373 | -0.037 | -0.073 | 14.4444444444444 | NA |
| LAVA_021 | MDG21205010 | NA | bovine | M | 2 | 0 | NA | NA | NA | NA | 0.805 | 0.373 | -0.037 | -0.073 | 14.4444444444444 | NA |
| LAVA_016 | MDG21205010 | NA | bovine | M | 3 | 0 | NA | NA | NA | NA | 0.805 | 0.373 | -0.037 | -0.073 | 14.4444444444444 | NA |
| LAVA_045 | MDG21205030 | NA | bovine | M | 5 | 0 | NA | NA | NA | NA | 0.463 | 0.857 | 0.114 | -0.084 | 12.5 | NA |
| LAVA_043 | MDG21205030 | NA | bovine | F | 6 | 0 | NA | NA | NA | NA | 0.463 | 0.857 | 0.114 | -0.084 | 12.5 | NA |
| LAVA_042 | MDG21205030 | NA | bovine | F | 2 | 0 | NA | NA | NA | NA | 0.463 | 0.857 | 0.114 | -0.084 | 12.5 | NA |
| LAVA_044 | MDG21205030 | NA | bovine | M | 3 | 0 | NA | NA | NA | NA | 0.463 | 0.857 | 0.114 | -0.084 | 12.5 | NA |
| LAVA_046 | MDG21205030 | NA | bovine | M | 3 | 0 | NA | NA | NA | NA | 0.463 | 0.857 | 0.114 | -0.084 | 12.5 | NA |
| LAVA_037 | MDG21205050 | NA | bovine | M | 2 | 0 | NA | NA | NA | NA | 0.897 | 0.517 | -0.305 | -0.008 | 20.375 | NA |
| LAVA_029 | MDG21205050 | NA | bovine | F | 5 | 0 | NA | NA | NA | NA | 0.897 | 0.517 | -0.305 | -0.008 | 20.375 | NA |
| LAVA_030 | MDG21205050 | NA | bovine | M | 6 | 0 | NA | NA | NA | NA | 0.897 | 0.517 | -0.305 | -0.008 | 20.375 | NA |
| LAVA_031 | MDG21205050 | NA | bovine | F | 6 | 0 | NA | NA | NA | NA | 0.897 | 0.517 | -0.305 | -0.008 | 20.375 | NA |
| LAVA_032 | MDG21205050 | NA | bovine | F | 10 | 0 | NA | NA | NA | NA | 0.897 | 0.517 | -0.305 | -0.008 | 20.375 | NA |
| LAVA_033 | MDG21205050 | NA | bovine | M | 5 | 0 | NA | NA | NA | NA | 0.897 | 0.517 | -0.305 | -0.008 | 20.375 | NA |
| LAVA_034 | MDG21205050 | NA | bovine | M | 3 | 0 | NA | NA | NA | NA | 0.897 | 0.517 | -0.305 | -0.008 | 20.375 | NA |
| LAVA_035 | MDG21205050 | NA | bovine | M | 3 | 0 | NA | NA | NA | NA | 0.897 | 0.517 | -0.305 | -0.008 | 20.375 | NA |
| LAVA_038 | MDG21205050 | NA | bovine | M | 4 | 0 | NA | NA | NA | NA | 0.897 | 0.517 | -0.305 | -0.008 | 20.375 | NA |
| LAVA_036 | MDG21205050 | NA | bovine | M | 5 | 0 | NA | NA | NA | NA | 0.897 | 0.517 | -0.305 | -0.008 | 20.375 | NA |
| LAVA_069 | MDG21205071 | NA | bovine | M | 2 | 0 | NA | NA | NA | NA | 0.742 | 0.066 | 0.058 | -0.418 | 13.125 | NA |
| LAVA_073 | MDG21205071 | NA | bovine | F | 8 | 0 | NA | NA | NA | NA | 0.742 | 0.066 | 0.058 | -0.418 | 13.125 | NA |
| LAVA_071 | MDG21205071 | NA | bovine | F | 6 | 0 | NA | NA | NA | NA | 0.742 | 0.066 | 0.058 | -0.418 | 13.125 | NA |
| LAVA_075 | MDG21205071 | NA | bovine | M | 3 | 0 | NA | NA | NA | NA | 0.742 | 0.066 | 0.058 | -0.418 | 13.125 | NA |
| LAVA_074 | MDG21205071 | NA | bovine | M | 3 | 0 | NA | NA | NA | NA | 0.742 | 0.066 | 0.058 | -0.418 | 13.125 | NA |
| LAVA_070 | MDG21205071 | NA | bovine | M | 4 | 0 | NA | NA | NA | NA | 0.742 | 0.066 | 0.058 | -0.418 | 13.125 | NA |
| LAVA_067 | MDG21205071 | NA | bovine | F | 5 | 0 | NA | NA | NA | NA | 0.742 | 0.066 | 0.058 | -0.418 | 13.125 | NA |
| LAVA_068 | MDG21205071 | NA | bovine | M | 3 | 0 | NA | NA | NA | NA | 0.742 | 0.066 | 0.058 | -0.418 | 13.125 | NA |
| LAVA_066 | MDG21205071 | NA | bovine | M | 4 | 0 | NA | NA | NA | NA | 0.742 | 0.066 | 0.058 | -0.418 | 13.125 | NA |
| LAVA_072 | MDG21205071 | NA | bovine | M | 3 | 0 | NA | NA | NA | NA | 0.742 | 0.066 | 0.058 | -0.418 | 13.125 | NA |
| LAVA_047 | MDG21205090 | NA | bovine | F | 7 | 0 | NA | NA | NA | NA | 0.273 | 0.091 | 0.319 | -0.662 | 17.25 | NA |
| LAVA_048 | MDG21205090 | NA | bovine | M | 6 | 0 | NA | NA | NA | NA | 0.273 | 0.091 | 0.319 | -0.662 | 17.25 | NA |
| LAVA_049 | MDG21205090 | NA | bovine | M | 4 | 0 | NA | NA | NA | NA | 0.273 | 0.091 | 0.319 | -0.662 | 17.25 | NA |
| LAVA_009 | MDG21205150 | NA | bovine | M | 3 | 0 | NA | NA | NA | NA | 1.202 | 0.321 | -0.472 | 0.008 | 15.7142857142857 | NA |
| LAVA_010 | MDG21205150 | NA | bovine | F | 2 | 0 | NA | NA | NA | NA | 1.202 | 0.321 | -0.472 | 0.008 | 15.7142857142857 | NA |
| LAVA_011 | MDG21205150 | NA | bovine | M | 5 | 0 | NA | NA | NA | NA | 1.202 | 0.321 | -0.472 | 0.008 | 15.7142857142857 | NA |
| LAVA_008 | MDG21205170 | NA | bovine | M | 5 | 0 | NA | NA | NA | NA | -0.307 | 0.274 | 0.644 | -0.650 | 25.4444444444444 | NA |
| LAVA_057 | MDG21205211 | NA | bovine | F | 6 | 0 | NA | NA | NA | NA | 1.315 | 0.136 | -0.540 | 0.174 | 26.4666666666666 | NA |
| LAVA_058 | MDG21205211 | NA | bovine | F | 5 | 0 | NA | NA | NA | NA | 1.315 | 0.136 | -0.540 | 0.174 | 26.4666666666666 | NA |
| LAVA_056 | MDG21205211 | NA | bovine | F | 4 | 0 | NA | NA | NA | NA | 1.315 | 0.136 | -0.540 | 0.174 | 26.4666666666666 | NA |
| LAVA_055 | MDG21205211 | NA | bovine | M | 4 | 0 | NA | NA | NA | NA | 1.315 | 0.136 | -0.540 | 0.174 | 26.4666666666666 | NA |
| LAVA_054 | MDG21205211 | NA | bovine | M | 3 | 0 | NA | NA | NA | NA | 1.315 | 0.136 | -0.540 | 0.174 | 26.4666666666666 | NA |
| LAVA_001 | MDG21205211 | NA | bovine | F | 2 | 0 | NA | NA | NA | NA | 1.315 | 0.136 | -0.540 | 0.174 | 26.4666666666666 | NA |
| LAVA_053 | MDG21205211 | NA | bovine | F | 5 | 0 | NA | NA | NA | NA | 1.315 | 0.136 | -0.540 | 0.174 | 26.4666666666666 | NA |
| LAVA_052 | MDG21205211 | NA | bovine | F | 3 | 0 | NA | NA | NA | NA | 1.315 | 0.136 | -0.540 | 0.174 | 26.4666666666666 | NA |
| LAVA_051 | MDG21205211 | NA | bovine | M | 2 | 0 | NA | NA | NA | NA | 1.315 | 0.136 | -0.540 | 0.174 | 26.4666666666666 | NA |
| LAVA_050 | MDG21205211 | NA | bovine | M | 2 | 0 | NA | NA | NA | NA | 1.315 | 0.136 | -0.540 | 0.174 | 26.4666666666666 | NA |
| LAVA_004 | MDG21205250 | NA | bovine | M | 2 | 0 | NA | NA | NA | NA | -1.011 | 0.384 | 0.605 | -0.756 | 18 | NA |
| LAVA_007 | MDG21205250 | NA | bovine | F | 2 | 1 | NA | NA | NA | NA | -1.011 | 0.384 | 0.605 | -0.756 | 18 | NA |
| LAVA_006 | MDG21205250 | NA | bovine | M | 6 | 0 | NA | NA | NA | NA | -1.011 | 0.384 | 0.605 | -0.756 | 18 | NA |
| LAVA_005 | MDG21205250 | NA | bovine | M | 3 | 0 | NA | NA | NA | NA | -1.011 | 0.384 | 0.605 | -0.756 | 18 | NA |
| LAVA_003 | MDG21205250 | NA | bovine | M | 3 | 0 | NA | NA | NA | NA | -1.011 | 0.384 | 0.605 | -0.756 | 18 | NA |
| LAVA_064 | MDG21205270 | NA | bovine | M | 2 | 0 | NA | NA | NA | NA | 0.776 | 0.216 | 0.290 | -0.558 | 16.75 | NA |
| LAVA_059 | MDG21205270 | NA | bovine | M | 2 | 0 | NA | NA | NA | NA | 0.776 | 0.216 | 0.290 | -0.558 | 16.75 | NA |
| LAVA_060 | MDG21205270 | NA | bovine | M | 6 | 0 | NA | NA | NA | NA | 0.776 | 0.216 | 0.290 | -0.558 | 16.75 | NA |
| LAVA_061 | MDG21205270 | NA | bovine | M | 2 | 0 | NA | NA | NA | NA | 0.776 | 0.216 | 0.290 | -0.558 | 16.75 | NA |
| LAVA_063 | MDG21205270 | NA | bovine | M | 5 | 0 | NA | NA | NA | NA | 0.776 | 0.216 | 0.290 | -0.558 | 16.75 | NA |
| LAVA_065 | MDG21205270 | NA | bovine | F | 2 | 0 | NA | NA | NA | NA | 0.776 | 0.216 | 0.290 | -0.558 | 16.75 | NA |
| LAVA_062 | MDG21205270 | NA | bovine | F | 2 | 0 | NA | NA | NA | NA | 0.776 | 0.216 | 0.290 | -0.558 | 16.75 | NA |

| ID | PCODE | site2 | sp | gender | age | IgG | contact_ruminant | contact_milk | contact_fresh_fluid | profession | fact1 | fact2 | fact3 | fact4 | cattle_density | habitat |
| --- | --- | --- | --- | --- | --- | --- | --- | --- | --- | --- | --- | --- | --- | --- | --- | --- |
| MANA 044 | MDG23210051 | NA | bovine | F | 6 | 0 | NA | NA | NA | NA | -0.909 | 0.121 | 0.393 | 0.553 | 0 | NA |
| MANA 038 | MDG23210051 | NA | bovine | F | 5 | 0 | NA | NA | NA | NA | -0.909 | 0.121 | 0.393 | 0.553 | 0 | NA |
| MANA 047 | MDG23210051 | NA | bovine | F | 7 | 1 | NA | NA | NA | NA | -0.909 | 0.121 | 0.393 | 0.553 | 0 | NA |
| MANA 049 | MDG23210051 | NA | bovine | M | 9 | 1 | NA | NA | NA | NA | -0.909 | 0.121 | 0.393 | 0.553 | 0 | NA |
| MANA 046 | MDG23210051 | NA | bovine | M | 3 | 1 | NA | NA | NA | NA | -0.909 | 0.121 | 0.393 | 0.553 | 0 | NA |
| MANA 045 | MDG23210051 | NA | bovine | M | 6 | 0 | NA | NA | NA | NA | -0.909 | 0.121 | 0.393 | 0.553 | 0 | NA |
| MANA 048 | MDG23210051 | NA | bovine | F | 8 | 0 | NA | NA | NA | NA | -0.909 | 0.121 | 0.393 | 0.553 | 0 | NA |
| MANA 043 | MDG23210051 | NA | bovine | F | 5 | 1 | NA | NA | NA | NA | -0.909 | 0.121 | 0.393 | 0.553 | 0 | NA |
| MANA 042 | MDG23210051 | NA | bovine | M | 8 | 1 | NA | NA | NA | NA | -0.909 | 0.121 | 0.393 | 0.553 | 0 | NA |
| MANA 041 | MDG23210051 | NA | bovine | F | 6 | 0 | NA | NA | NA | NA | -0.909 | 0.121 | 0.393 | 0.553 | 0 | NA |
| MANA 050 | MDG23210051 | NA | bovine | M | 9 | 1 | NA | NA | NA | NA | -0.909 | 0.121 | 0.393 | 0.553 | 0 | NA |
| MANA 039 | MDG23210051 | NA | bovine | M | 3 | 0 | NA | NA | NA | NA | -0.909 | 0.121 | 0.393 | 0.553 | 0 | NA |
| MANA 063 | MDG23210051 | NA | bovine | M | 3 | 1 | NA | NA | NA | NA | -0.909 | 0.121 | 0.393 | 0.553 | 0 | NA |
| MANA 037 | MDG23210051 | NA | bovine | F | 6 | 0 | NA | NA | NA | NA | -0.909 | 0.121 | 0.393 | 0.553 | 0 | NA |
| MANA 036 | MDG23210051 | NA | bovine | F | 3 | 0 | NA | NA | NA | NA | -0.909 | 0.121 | 0.393 | 0.553 | 0 | NA |
| MANA 035 | MDG23210051 | NA | bovine | F | 4 | 0 | NA | NA | NA | NA | -0.909 | 0.121 | 0.393 | 0.553 | 0 | NA |
| MANA 040 | MDG23210051 | NA | bovine | F | 7 | 0 | NA | NA | NA | NA | -0.909 | 0.121 | 0.393 | 0.553 | 0 | NA |
| MANA 067 | MDG23210051 | NA | bovine | M | 4 | 0 | NA | NA | NA | NA | -0.909 | 0.121 | 0.393 | 0.553 | 0 | NA |
| MANA 058 | MDG23210051 | NA | bovine | F | 4 | 0 | NA | NA | NA | NA | -0.909 | 0.121 | 0.393 | 0.553 | 0 | NA |
| MANA 034 | MDG23210051 | NA | bovine | M | 2 | 0 | NA | NA | NA | NA | -0.909 | 0.121 | 0.393 | 0.553 | 0 | NA |
| MANA 076 | MDG23210051 | NA | bovine | M | 6 | 0 | NA | NA | NA | NA | -0.909 | 0.121 | 0.393 | 0.553 | 0 | NA |
| MANA 075 | MDG23210051 | NA | bovine | M | 8 | 1 | NA | NA | NA | NA | -0.909 | 0.121 | 0.393 | 0.553 | 0 | NA |
| MANA 074 | MDG23210051 | NA | bovine | F | 8 | 0 | NA | NA | NA | NA | -0.909 | 0.121 | 0.393 | 0.553 | 0 | NA |
| MANA 073 | MDG23210051 | NA | bovine | F | 8 | 1 | NA | NA | NA | NA | -0.909 | 0.121 | 0.393 | 0.553 | 0 | NA |
| MANA 061 | MDG23210051 | NA | bovine | M | 2 | 1 | NA | NA | NA | NA | -0.909 | 0.121 | 0.393 | 0.553 | 0 | NA |
| MANA 068 | MDG23210051 | NA | bovine | M | 3 | 1 | NA | NA | NA | NA | -0.909 | 0.121 | 0.393 | 0.553 | 0 | NA |
| MANA 051 | MDG23210051 | NA | bovine | M | 10 | 0 | NA | NA | NA | NA | -0.909 | 0.121 | 0.393 | 0.553 | 0 | NA |
| MANA 066 | MDG23210051 | NA | bovine | M | 4 | 1 | NA | NA | NA | NA | -0.909 | 0.121 | 0.393 | 0.553 | 0 | NA |
| MANA 065 | MDG23210051 | NA | bovine | M | 5 | 0 | NA | NA | NA | NA | -0.909 | 0.121 | 0.393 | 0.553 | 0 | NA |
| MANA 064 | MDG23210051 | NA | bovine | M | 7 | 0 | NA | NA | NA | NA | -0.909 | 0.121 | 0.393 | 0.553 | 0 | NA |
| MANA 062 | MDG23210051 | NA | bovine | M | 4 | 0 | NA | NA | NA | NA | -0.909 | 0.121 | 0.393 | 0.553 | 0 | NA |
| MANA 059 | MDG23210051 | NA | bovine | F | 5 | 1 | NA | NA | NA | NA | -0.909 | 0.121 | 0.393 | 0.553 | 0 | NA |
| MANA 053 | MDG23210051 | NA | bovine | M | 8 | 0 | NA | NA | NA | NA | -0.909 | 0.121 | 0.393 | 0.553 | 0 | NA |
| MANA 052 | MDG23210051 | NA | bovine | F | 9 | 1 | NA | NA | NA | NA | -0.909 | 0.121 | 0.393 | 0.553 | 0 | NA |
| MANA 069 | MDG23210051 | NA | bovine | F | 5 | 1 | NA | NA | NA | NA | -0.909 | 0.121 | 0.393 | 0.553 | 0 | NA |
| MANA 060 | MDG23210051 | NA | bovine | M | 4 | 1 | NA | NA | NA | NA | -0.909 | 0.121 | 0.393 | 0.553 | 0 | NA |
| IHO 048 | MDG24216010 | NA | bovine | M | 2 | 0 | NA | NA | NA | NA | 1.469 | 0.202 | -0.965 | 0.011 | 8 | NA |
| IHO 065 | MDG24216010 | NA | bovine | F | 5 | 0 | NA | NA | NA | NA | 1.469 | 0.202 | -0.965 | 0.011 | 8 | NA |
| IHO 060 | MDG24216010 | NA | bovine | M | 1 | 1 | NA | NA | NA | NA | 1.469 | 0.202 | -0.965 | 0.011 | 8 | NA |
| IHO 064 | MDG24216010 | NA | bovine | M | 1 | 0 | NA | NA | NA | NA | 1.469 | 0.202 | -0.965 | 0.011 | 8 | NA |
| IHO 063 | MDG24216010 | NA | bovine | F | 1 | 0 | NA | NA | NA | NA | 1.469 | 0.202 | -0.965 | 0.011 | 8 | NA |
| IHO 047 | MDG24216010 | NA | bovine | M | 2 | 0 | NA | NA | NA | NA | 1.469 | 0.202 | -0.965 | 0.011 | 8 | NA |
| IHO 049 | MDG24216010 | NA | bovine | F | 3 | 0 | NA | NA | NA | NA | 1.469 | 0.202 | -0.965 | 0.011 | 8 | NA |
| IHO 050 | MDG24216010 | NA | bovine | M | 1 | 0 | NA | NA | NA | NA | 1.469 | 0.202 | -0.965 | 0.011 | 8 | NA |
| IHO 051 | MDG24216010 | NA | bovine | F | 1 | 0 | NA | NA | NA | NA | 1.469 | 0.202 | -0.965 | 0.011 | 8 | NA |
| IHO 052 | MDG24216010 | NA | bovine | F | 1 | 0 | NA | NA | NA | NA | 1.469 | 0.202 | -0.965 | 0.011 | 8 | NA |
| IHO 053 | MDG24216010 | NA | bovine | F | 2 | 0 | NA | NA | NA | NA | 1.469 | 0.202 | -0.965 | 0.011 | 8 | NA |
| IHO 054 | MDG24216010 | NA | bovine | M | 1 | 0 | NA | NA | NA | NA | 1.469 | 0.202 | -0.965 | 0.011 | 8 | NA |
| IHO 062 | MDG24216010 | NA | bovine | F | 1 | 0 | NA | NA | NA | NA | 1.469 | 0.202 | -0.965 | 0.011 | 8 | NA |
| IHO 061 | MDG24216010 | NA | bovine | F | 2 | 0 | NA | NA | NA | NA | 1.469 | 0.202 | -0.965 | 0.011 | 8 | NA |
| IHO 034 | MDG24216031 | NA | bovine | F | 2 | 0 | NA | NA | NA | NA | 1.431 | 0.094 | -0.913 | 0.045 | 11.1428571428571 | NA |
| IHO 025 | MDG24216031 | NA | bovine | M | 2 | 0 | NA | NA | NA | NA | 1.431 | 0.094 | -0.913 | 0.045 | 11.1428571428571 | NA |
| IHO 026 | MDG24216031 | NA | bovine | F | 7 | 0 | NA | NA | NA | NA | 1.431 | 0.094 | -0.913 | 0.045 | 11.1428571428571 | NA |
| IHO 027 | MDG24216031 | NA | bovine | F | 2 | 0 | NA | NA | NA | NA | 1.431 | 0.094 | -0.913 | 0.045 | 11.1428571428571 | NA |
| IHO 028 | MDG24216031 | NA | bovine | F | 2 | 0 | NA | NA | NA | NA | 1.431 | 0.094 | -0.913 | 0.045 | 11.1428571428571 | NA |
| IHO 029 | MDG24216031 | NA | bovine | M | 3 | 0 | NA | NA | NA | NA | 1.431 | 0.094 | -0.913 | 0.045 | 11.1428571428571 | NA |
| IHO 030 | MDG24216031 | NA | bovine | M | 2 | 0 | NA | NA | NA | NA | 1.431 | 0.094 | -0.913 | 0.045 | 11.1428571428571 | NA |
| IHO 031 | MDG24216031 | NA | bovine | F | 6 | 0 | NA | NA | NA | NA | 1.431 | 0.094 | -0.913 | 0.045 | 11.1428571428571 | NA |
| IHO 033 | MDG24216031 | NA | bovine | M | 4 | 0 | NA | NA | NA | NA | 1.431 | 0.094 | -0.913 | 0.045 | 11.1428571428571 | NA |
| IHO 032 | MDG24216031 | NA | bovine | F | 2 | 0 | NA | NA | NA | NA | 1.431 | 0.094 | -0.913 | 0.045 | 11.1428571428571 | NA |
| IHO 006 | MDG24216032 | NA | bovine | F | 2 | 0 | NA | NA | NA | NA | 1.551 | 0.033 | -0.905 | 0.058 | 8.8 | NA |
| IHO 004 | MDG24216032 | NA | bovine | M | 3 | 0 | NA | NA | NA | NA | 1.551 | 0.033 | -0.905 | 0.058 | 8.8 | NA |
| IHO 005 | MDG24216032 | NA | bovine | M | 1 | 0 | NA | NA | NA | NA | 1.551 | 0.033 | -0.905 | 0.058 | 8.8 | NA |

| ID | PCODE | site2 | sp | gender | age | IgG | contact_ruminant | contact_milk | contact_fresh_fluid | profession | fact1 | fact2 | fact3 | fact4 | cattle_density | habitat |
| --- | --- | --- | --- | --- | --- | --- | --- | --- | --- | --- | --- | --- | --- | --- | --- | --- |
| IHO 003 | MDG24216032 | NA | bovine | F | 3 | 0 | NA | NA | NA | NA | 1.551 | 0.033 | -0.905 | 0.058 | 8.8 | NA |
| IHO 001 | MDG24216032 | NA | bovine | M | 3 | 0 | NA | NA | NA | NA | 1.551 | 0.033 | -0.905 | 0.058 | 8.8 | NA |
| IHO 010 | MDG24216032 | NA | bovine | M | 2 | 0 | NA | NA | NA | NA | 1.551 | 0.033 | -0.905 | 0.058 | 8.8 | NA |
| IHO 007 | MDG24216032 | NA | bovine | M | 2 | 0 | NA | NA | NA | NA | 1.551 | 0.033 | -0.905 | 0.058 | 8.8 | NA |
| IHO 008 | MDG24216032 | NA | bovine | F | 3 | 1 | NA | NA | NA | NA | 1.551 | 0.033 | -0.905 | 0.058 | 8.8 | NA |
| IHO 009 | MDG24216032 | NA | bovine | F | 3 | 0 | NA | NA | NA | NA | 1.551 | 0.033 | -0.905 | 0.058 | 8.8 | NA |
| IHO 011 | MDG24216032 | NA | bovine | F | 2 | 0 | NA | NA | NA | NA | 1.551 | 0.033 | -0.905 | 0.058 | 8.8 | NA |
| IHO 002 | MDG24216032 | NA | bovine | F | 3 | 1 | NA | NA | NA | NA | 1.551 | 0.033 | -0.905 | 0.058 | 8.8 | NA |
| IHO 019 | MDG24216033 | NA | bovine | F | 3 | 1 | NA | NA | NA | NA | 1.171 | 0.372 | -0.778 | -0.104 | 9.66666666666666 | NA |
| IHO 017 | MDG24216033 | NA | bovine | F | 1 | 0 | NA | NA | NA | NA | 1.171 | 0.372 | -0.778 | -0.104 | 9.66666666666666 | NA |
| IHO 020 | MDG24216033 | NA | bovine | F | 2 | 0 | NA | NA | NA | NA | 1.171 | 0.372 | -0.778 | -0.104 | 9.66666666666666 | NA |
| IHO 024 | MDG24216033 | NA | bovine | M | 1 | 0 | NA | NA | NA | NA | 1.171 | 0.372 | -0.778 | -0.104 | 9.66666666666666 | NA |
| IHO 023 | MDG24216033 | NA | bovine | F | 2 | 0 | NA | NA | NA | NA | 1.171 | 0.372 | -0.778 | -0.104 | 9.66666666666666 | NA |
| IHO 022 | MDG24216033 | NA | bovine | F | 2 | 0 | NA | NA | NA | NA | 1.171 | 0.372 | -0.778 | -0.104 | 9.66666666666666 | NA |
| IHO 016 | MDG24216033 | NA | bovine | F | 2 | 0 | NA | NA | NA | NA | 1.171 | 0.372 | -0.778 | -0.104 | 9.66666666666666 | NA |
| IHO 012 | MDG24216033 | NA | bovine | M | 3 | 0 | NA | NA | NA | NA | 1.171 | 0.372 | -0.778 | -0.104 | 9.66666666666666 | NA |
| IHO 013 | MDG24216033 | NA | bovine | M | 3 | 0 | NA | NA | NA | NA | 1.171 | 0.372 | -0.778 | -0.104 | 9.66666666666666 | NA |
| IHO 021 | MDG24216033 | NA | bovine | F | 2 | 0 | NA | NA | NA | NA | 1.171 | 0.372 | -0.778 | -0.104 | 9.66666666666666 | NA |
| IHO 014 | MDG24216033 | NA | bovine | F | 8 | 0 | NA | NA | NA | NA | 1.171 | 0.372 | -0.778 | -0.104 | 9.66666666666666 | NA |
| IHO 018 | MDG24216033 | NA | bovine | M | 2 | 0 | NA | NA | NA | NA | 1.171 | 0.372 | -0.778 | -0.104 | 9.66666666666666 | NA |
| IHO 036 | MDG24216070 | NA | bovine | F | 6 | 0 | NA | NA | NA | NA | 1.101 | 0.307 | -0.624 | -0.088 | 7.66666666666666 | NA |
| IHO 045 | MDG24216070 | NA | bovine | M | 1 | 0 | NA | NA | NA | NA | 1.101 | 0.307 | -0.624 | -0.088 | 7.66666666666666 | NA |
| IHO 041 | MDG24216070 | NA | bovine | F | 1 | 0 | NA | NA | NA | NA | 1.101 | 0.307 | -0.624 | -0.088 | 7.66666666666666 | NA |
| IHO 043 | MDG24216070 | NA | bovine | F | 2 | 0 | NA | NA | NA | NA | 1.101 | 0.307 | -0.624 | -0.088 | 7.66666666666666 | NA |
| IHO 038 | MDG24216070 | NA | bovine | F | 3 | 0 | NA | NA | NA | NA | 1.101 | 0.307 | -0.624 | -0.088 | 7.66666666666666 | NA |
| IHO 042 | MDG24216070 | NA | bovine | F | 1 | 0 | NA | NA | NA | NA | 1.101 | 0.307 | -0.624 | -0.088 | 7.66666666666666 | NA |
| IHO 046 | MDG24216070 | NA | bovine | M | 2 | 0 | NA | NA | NA | NA | 1.101 | 0.307 | -0.624 | -0.088 | 7.66666666666666 | NA |
| IHO 040 | MDG24216070 | NA | bovine | M | 1 | 0 | NA | NA | NA | NA | 1.101 | 0.307 | -0.624 | -0.088 | 7.66666666666666 | NA |
| IHO 039 | MDG24216070 | NA | bovine | M | 3 | 1 | NA | NA | NA | NA | 1.101 | 0.307 | -0.624 | -0.088 | 7.66666666666666 | NA |
| IHO 037 | MDG24216070 | NA | bovine | F | 8 | 0 | NA | NA | NA | NA | 1.101 | 0.307 | -0.624 | -0.088 | 7.66666666666666 | NA |
| IHO 044 | MDG24216070 | NA | bovine | F | 3 | 0 | NA | NA | NA | NA | 1.101 | 0.307 | -0.624 | -0.088 | 7.66666666666666 | NA |
| IHO 084 | MDG24216131 | NA | bovine | M | 2 | 0 | NA | NA | NA | NA | 1.630 | -0.122 | -0.914 | -0.442 | 9.175 | NA |
| IHO 096 | MDG24216131 | NA | bovine | F | 7 | 0 | NA | NA | NA | NA | 1.630 | -0.122 | -0.914 | -0.442 | 9.175 | NA |
| IHO 095 | MDG24216131 | NA | bovine | M | 4 | 0 | NA | NA | NA | NA | 1.630 | -0.122 | -0.914 | -0.442 | 9.175 | NA |
| IHO 094 | MDG24216131 | NA | bovine | F | 6 | 0 | NA | NA | NA | NA | 1.630 | -0.122 | -0.914 | -0.442 | 9.175 | NA |
| IHO 093 | MDG24216131 | NA | bovine | M | 3 | 0 | NA | NA | NA | NA | 1.630 | -0.122 | -0.914 | -0.442 | 9.175 | NA |
| IHO 091 | MDG24216131 | NA | bovine | F | 8 | 0 | NA | NA | NA | NA | 1.630 | -0.122 | -0.914 | -0.442 | 9.175 | NA |
| IHO 090 | MDG24216131 | NA | bovine | F | 7 | 0 | NA | NA | NA | NA | 1.630 | -0.122 | -0.914 | -0.442 | 9.175 | NA |
| IHO 089 | MDG24216131 | NA | bovine | F | 8 | 1 | NA | NA | NA | NA | 1.630 | -0.122 | -0.914 | -0.442 | 9.175 | NA |
| IHO 088 | MDG24216131 | NA | bovine | F | 6 | 0 | NA | NA | NA | NA | 1.630 | -0.122 | -0.914 | -0.442 | 9.175 | NA |
| IHO 087 | MDG24216131 | NA | bovine | F | 7 | 0 | NA | NA | NA | NA | 1.630 | -0.122 | -0.914 | -0.442 | 9.175 | NA |
| IHO 072 | MDG24216131 | NA | bovine | F | 7 | 0 | NA | NA | NA | NA | 1.630 | -0.122 | -0.914 | -0.442 | 9.175 | NA |
| IHO 085 | MDG24216131 | NA | bovine | F | 8 | 0 | NA | NA | NA | NA | 1.630 | -0.122 | -0.914 | -0.442 | 9.175 | NA |
| IHO 070 | MDG24216131 | NA | bovine | F | 7 | 0 | NA | NA | NA | NA | 1.630 | -0.122 | -0.914 | -0.442 | 9.175 | NA |
| IHO 083 | MDG24216131 | NA | bovine | F | 7 | 0 | NA | NA | NA | NA | 1.630 | -0.122 | -0.914 | -0.442 | 9.175 | NA |
| IHO 082 | MDG24216131 | NA | bovine | F | 8 | 0 | NA | NA | NA | NA | 1.630 | -0.122 | -0.914 | -0.442 | 9.175 | NA |
| IHO 081 | MDG24216131 | NA | bovine | F | 7 | 0 | NA | NA | NA | NA | 1.630 | -0.122 | -0.914 | -0.442 | 9.175 | NA |
| IHO 080 | MDG24216131 | NA | bovine | F | 8 | 0 | NA | NA | NA | NA | 1.630 | -0.122 | -0.914 | -0.442 | 9.175 | NA |
| IHO 079 | MDG24216131 | NA | bovine | M | 4 | 0 | NA | NA | NA | NA | 1.630 | -0.122 | -0.914 | -0.442 | 9.175 | NA |
| IHO 078 | MDG24216131 | NA | bovine | F | 8 | 0 | NA | NA | NA | NA | 1.630 | -0.122 | -0.914 | -0.442 | 9.175 | NA |
| IHO 075 | MDG24216131 | NA | bovine | F | 3 | 1 | NA | NA | NA | NA | 1.630 | -0.122 | -0.914 | -0.442 | 9.175 | NA |
| IHO 074 | MDG24216131 | NA | bovine | F | 6 | 1 | NA | NA | NA | NA | 1.630 | -0.122 | -0.914 | -0.442 | 9.175 | NA |
| IHO 073 | MDG24216131 | NA | bovine | F | 8 | 1 | NA | NA | NA | NA | 1.630 | -0.122 | -0.914 | -0.442 | 9.175 | NA |
| IHO 086 | MDG24216131 | NA | bovine | F | 3 | 0 | NA | NA | NA | NA | 1.630 | -0.122 | -0.914 | -0.442 | 9.175 | NA |
| IHO 076 | MDG24216131 | NA | bovine | F | 8 | 0 | NA | NA | NA | NA | 1.630 | -0.122 | -0.914 | -0.442 | 9.175 | NA |
| IHO 055 | MDG24216151 | NA | bovine | F | 2 | 1 | NA | NA | NA | NA | 1.438 | 0.137 | -0.446 | 0.047 | 15.1515151515151 | NA |
| IHO 056 | MDG24216151 | NA | bovine | F | 2 | 0 | NA | NA | NA | NA | 1.438 | 0.137 | -0.446 | 0.047 | 15.1515151515151 | NA |
| IHO 057 | MDG24216151 | NA | bovine | M | 3 | 0 | NA | NA | NA | NA | 1.438 | 0.137 | -0.446 | 0.047 | 15.1515151515151 | NA |
| IHO 058 | MDG24216151 | NA | bovine | M | 2 | 0 | NA | NA | NA | NA | 1.438 | 0.137 | -0.446 | 0.047 | 15.1515151515151 | NA |
| IHO 059 | MDG24216151 | NA | bovine | F | 5 | 0 | NA | NA | NA | NA | 1.438 | 0.137 | -0.446 | 0.047 | 15.1515151515151 | NA |
| IHO 035 | MDG24216151 | NA | bovine | M | 4 | 0 | NA | NA | NA | NA | 1.438 | 0.137 | -0.446 | 0.047 | 15.1515151515151 | NA |
| IHO 112 | MDG24216191 | NA | bovine | M | 3 | 0 | NA | NA | NA | NA | 1.634 | -0.712 | -0.509 | -0.449 | 9.80645161290322 | NA |
| IHO 111 | MDG24216191 | NA | bovine | M | 4 | 0 | NA | NA | NA | NA | 1.634 | -0.712 | -0.509 | -0.449 | 9.80645161290322 | NA |

| ID | PCODE | site2 | sp | gender | age | IgG | contact_ruminant | contact_milk | contact_fresh_fluid | profession | fact1 | fact2 | fact3 | fact4 | cattle_density | habitat |
| --- | --- | --- | --- | --- | --- | --- | --- | --- | --- | --- | --- | --- | --- | --- | --- | --- |
| IHO 110 | MDG24216191 | NA | bovine | M | 4 | 1 | NA | NA | NA | NA | 1.634 | -0.712 | -0.509 | -0.449 | 9.80645161290322 | NA |
| IHO 109 | MDG24216191 | NA | bovine | M | 4 | 1 | NA | NA | NA | NA | 1.634 | -0.712 | -0.509 | -0.449 | 9.80645161290322 | NA |
| IHO 108 | MDG24216191 | NA | bovine | M | 4 | 0 | NA | NA | NA | NA | 1.634 | -0.712 | -0.509 | -0.449 | 9.80645161290322 | NA |
| IHO 107 | MDG24216191 | NA | bovine | M | 3 | 0 | NA | NA | NA | NA | 1.634 | -0.712 | -0.509 | -0.449 | 9.80645161290322 | NA |
| IHO 104 | MDG24216191 | NA | bovine | M | 3 | 0 | NA | NA | NA | NA | 1.634 | -0.712 | -0.509 | -0.449 | 9.80645161290322 | NA |
| IHO 113 | MDG24216191 | NA | bovine | M | 4 | 0 | NA | NA | NA | NA | 1.634 | -0.712 | -0.509 | -0.449 | 9.80645161290322 | NA |
| IHO 102 | MDG24216191 | NA | bovine | M | 4 | 0 | NA | NA | NA | NA | 1.634 | -0.712 | -0.509 | -0.449 | 9.80645161290322 | NA |
| IHO 127 | MDG24216191 | NA | bovine | M | 4 | 0 | NA | NA | NA | NA | 1.634 | -0.712 | -0.509 | -0.449 | 9.80645161290322 | NA |
| IHO 101 | MDG24216191 | NA | bovine | M | 3 | 0 | NA | NA | NA | NA | 1.634 | -0.712 | -0.509 | -0.449 | 9.80645161290322 | NA |
| IHO 099 | MDG24216191 | NA | bovine | M | 3 | 0 | NA | NA | NA | NA | 1.634 | -0.712 | -0.509 | -0.449 | 9.80645161290322 | NA |
| IHO 098 | MDG24216191 | NA | bovine | M | 2 | 0 | NA | NA | NA | NA | 1.634 | -0.712 | -0.509 | -0.449 | 9.80645161290322 | NA |
| IHO 097 | MDG24216191 | NA | bovine | M | 4 | 0 | NA | NA | NA | NA | 1.634 | -0.712 | -0.509 | -0.449 | 9.80645161290322 | NA |
| IHO 106 | MDG24216191 | NA | bovine | M | 2 | 0 | NA | NA | NA | NA | 1.634 | -0.712 | -0.509 | -0.449 | 9.80645161290322 | NA |
| IHO 123 | MDG24216191 | NA | bovine | M | 4 | 0 | NA | NA | NA | NA | 1.634 | -0.712 | -0.509 | -0.449 | 9.80645161290322 | NA |
| IHO 103 | MDG24216191 | NA | bovine | M | 4 | 1 | NA | NA | NA | NA | 1.634 | -0.712 | -0.509 | -0.449 | 9.80645161290322 | NA |
| IHO 115 | MDG24216191 | NA | bovine | M | 4 | 0 | NA | NA | NA | NA | 1.634 | -0.712 | -0.509 | -0.449 | 9.80645161290322 | NA |
| IHO 121 | MDG24216191 | NA | bovine | M | 4 | 0 | NA | NA | NA | NA | 1.634 | -0.712 | -0.509 | -0.449 | 9.80645161290322 | NA |
| IHO 120 | MDG24216191 | NA | bovine | M | 4 | 0 | NA | NA | NA | NA | 1.634 | -0.712 | -0.509 | -0.449 | 9.80645161290322 | NA |
| IHO 119 | MDG24216191 | NA | bovine | M | 4 | 0 | NA | NA | NA | NA | 1.634 | -0.712 | -0.509 | -0.449 | 9.80645161290322 | NA |
| IHO 125 | MDG24216191 | NA | bovine | M | 4 | 0 | NA | NA | NA | NA | 1.634 | -0.712 | -0.509 | -0.449 | 9.80645161290322 | NA |
| IHO 117 | MDG24216191 | NA | bovine | M | 4 | 0 | NA | NA | NA | NA | 1.634 | -0.712 | -0.509 | -0.449 | 9.80645161290322 | NA |
| IHO 114 | MDG24216191 | NA | bovine | M | 4 | 0 | NA | NA | NA | NA | 1.634 | -0.712 | -0.509 | -0.449 | 9.80645161290322 | NA |
| IHO 130 | MDG24216191 | NA | bovine | M | 4 | 0 | NA | NA | NA | NA | 1.634 | -0.712 | -0.509 | -0.449 | 9.80645161290322 | NA |
| IHO 129 | MDG24216191 | NA | bovine | M | 4 | 0 | NA | NA | NA | NA | 1.634 | -0.712 | -0.509 | -0.449 | 9.80645161290322 | NA |
| IHO 128 | MDG24216191 | NA | bovine | M | 4 | 0 | NA | NA | NA | NA | 1.634 | -0.712 | -0.509 | -0.449 | 9.80645161290322 | NA |
| IHO 105 | MDG24216191 | NA | bovine | M | 2 | 0 | NA | NA | NA | NA | 1.634 | -0.712 | -0.509 | -0.449 | 9.80645161290322 | NA |
| IHO 126 | MDG24216191 | NA | bovine | M | 4 | 0 | NA | NA | NA | NA | 1.634 | -0.712 | -0.509 | -0.449 | 9.80645161290322 | NA |
| IHO 122 | MDG24216191 | NA | bovine | M | 4 | 0 | NA | NA | NA | NA | 1.634 | -0.712 | -0.509 | -0.449 | 9.80645161290322 | NA |
| IHO 124 | MDG24216191 | NA | bovine | M | 4 | 0 | NA | NA | NA | NA | 1.634 | -0.712 | -0.509 | -0.449 | 9.80645161290322 | NA |
| IHO 118 | MDG24216191 | NA | bovine | M | 4 | 0 | NA | NA | NA | NA | 1.634 | -0.712 | -0.509 | -0.449 | 9.80645161290322 | NA |
| BOE 181 | MDG41405112 | NA | bovine | M | 2 | 0 | NA | NA | NA | NA | 1.536 | -0.365 | 0.691 | 0.473 | 13.1818181818181 | NA |
| BOE 182 | MDG41405112 | NA | bovine | M | 1 | 0 | NA | NA | NA | NA | 1.536 | -0.365 | 0.691 | 0.473 | 13.1818181818181 | NA |
| BOE 183 | MDG41405112 | NA | bovine | F | 1 | 0 | NA | NA | NA | NA | 1.536 | -0.365 | 0.691 | 0.473 | 13.1818181818181 | NA |
| BOE 184 | MDG41405112 | NA | bovine | M | 2 | 0 | NA | NA | NA | NA | 1.536 | -0.365 | 0.691 | 0.473 | 13.1818181818181 | NA |
| BOE 076 | MDG41405112 | NA | bovine | M | 1 | 0 | NA | NA | NA | NA | 1.536 | -0.365 | 0.691 | 0.473 | 13.1818181818181 | NA |
| BOE 021 | MDG41405112 | NA | bovine | M | 2 | 1 | NA | NA | NA | NA | 1.536 | -0.365 | 0.691 | 0.473 | 13.1818181818181 | NA |
| BOE 022 | MDG41405112 | NA | bovine | M | 2 | 0 | NA | NA | NA | NA | 1.536 | -0.365 | 0.691 | 0.473 | 13.1818181818181 | NA |
| BOE 023 | MDG41405112 | NA | bovine | F | 2 | 1 | NA | NA | NA | NA | 1.536 | -0.365 | 0.691 | 0.473 | 13.1818181818181 | NA |
| BOE 024 | MDG41405112 | NA | bovine | M | 2 | 0 | NA | NA | NA | NA | 1.536 | -0.365 | 0.691 | 0.473 | 13.1818181818181 | NA |
| BOE 071 | MDG41405112 | NA | bovine | F | 6 | 1 | NA | NA | NA | NA | 1.536 | -0.365 | 0.691 | 0.473 | 13.1818181818181 | NA |
| BOE 072 | MDG41405112 | NA | bovine | F | 2 | 0 | NA | NA | NA | NA | 1.536 | -0.365 | 0.691 | 0.473 | 13.1818181818181 | NA |
| BOE 073 | MDG41405112 | NA | bovine | F | 2 | 0 | NA | NA | NA | NA | 1.536 | -0.365 | 0.691 | 0.473 | 13.1818181818181 | NA |
| BOE 180 | MDG41405112 | NA | bovine | M | 2 | 0 | NA | NA | NA | NA | 1.536 | -0.365 | 0.691 | 0.473 | 13.1818181818181 | NA |
| BOE 075 | MDG41405112 | NA | bovine | M | 2 | 0 | NA | NA | NA | NA | 1.536 | -0.365 | 0.691 | 0.473 | 13.1818181818181 | NA |
| BOE 179 | MDG41405112 | NA | bovine | F | 3 | 0 | NA | NA | NA | NA | 1.536 | -0.365 | 0.691 | 0.473 | 13.1818181818181 | NA |
| BOE 077 | MDG41405112 | NA | bovine | M | 1 | 0 | NA | NA | NA | NA | 1.536 | -0.365 | 0.691 | 0.473 | 13.1818181818181 | NA |
| BOE 078 | MDG41405112 | NA | bovine | F | 4 | 0 | NA | NA | NA | NA | 1.536 | -0.365 | 0.691 | 0.473 | 13.1818181818181 | NA |
| BOE 079 | MDG41405112 | NA | bovine | F | 2 | 1 | NA | NA | NA | NA | 1.536 | -0.365 | 0.691 | 0.473 | 13.1818181818181 | NA |
| BOE 080 | MDG41405112 | NA | bovine | F | 2 | 0 | NA | NA | NA | NA | 1.536 | -0.365 | 0.691 | 0.473 | 13.1818181818181 | NA |
| BOE 096 | MDG41405112 | NA | bovine | M | 5 | 0 | NA | NA | NA | NA | 1.536 | -0.365 | 0.691 | 0.473 | 13.1818181818181 | NA |
| BOE 178 | MDG41405112 | NA | bovine | M | 2 | 1 | NA | NA | NA | NA | 1.536 | -0.365 | 0.691 | 0.473 | 13.1818181818181 | NA |
| BOE 187 | MDG41405112 | NA | bovine | M | 1 | 1 | NA | NA | NA | NA | 1.536 | -0.365 | 0.691 | 0.473 | 13.1818181818181 | NA |
| BOE 074 | MDG41405112 | NA | bovine | M | 1 | 0 | NA | NA | NA | NA | 1.536 | -0.365 | 0.691 | 0.473 | 13.1818181818181 | NA |
| BOE 229 | MDG41405112 | NA | bovine | M | 5 | 1 | NA | NA | NA | NA | 1.536 | -0.365 | 0.691 | 0.473 | 13.1818181818181 | NA |
| BOE 185 | MDG41405112 | NA | bovine | M | 2 | 0 | NA | NA | NA | NA | 1.536 | -0.365 | 0.691 | 0.473 | 13.1818181818181 | NA |
| BOE 186 | MDG41405112 | NA | bovine | M | 2 | 0 | NA | NA | NA | NA | 1.536 | -0.365 | 0.691 | 0.473 | 13.1818181818181 | NA |
| BOE 227 | MDG41405150 | NA | bovine | M | 2 | 0 | NA | NA | NA | NA | 1.722 | -0.894 | 0.461 | 0.559 | 12.4444444444444 | NA |
| BOE 226 | MDG41405150 | NA | bovine | F | 2 | 0 | NA | NA | NA | NA | 1.722 | -0.894 | 0.461 | 0.559 | 12.4444444444444 | NA |
| BOE 225 | MDG41405150 | NA | bovine | M | 2 | 0 | NA | NA | NA | NA | 1.722 | -0.894 | 0.461 | 0.559 | 12.4444444444444 | NA |
| BOE 224 | MDG41405150 | NA | bovine | M | 2 | 1 | NA | NA | NA | NA | 1.722 | -0.894 | 0.461 | 0.559 | 12.4444444444444 | NA |
| BOE 223 | MDG41405150 | NA | bovine | F | 3 | 0 | NA | NA | NA | NA | 1.722 | -0.894 | 0.461 | 0.559 | 12.4444444444444 | NA |
| BOE 214 | MDG41405150 | NA | bovine | F | 2 | 0 | NA | NA | NA | NA | 1.722 | -0.894 | 0.461 | 0.559 | 12.4444444444444 | NA |
| BOE 213 | MDG41405150 | NA | bovine | F | 2 | 1 | NA | NA | NA | NA | 1.722 | -0.894 | 0.461 | 0.559 | 12.4444444444444 | NA |

| ID | PCODE | site2 | sp | gender | age | IgG | contact_ruminant | contact_milk | contact_fresh_fluid | profession | fact1 | fact2 | fact3 | fact4 | cattle_density | habitat |
| --- | --- | --- | --- | --- | --- | --- | --- | --- | --- | --- | --- | --- | --- | --- | --- | --- |
| BOE 212 | MDG41405150 | NA | bovine | M | 2 | 0 | NA | NA | NA | NA | 1.722 | -0.894 | 0.461 | 0.559 | 12.4444444444444 | NA |
| BOE 211 | MDG41405150 | NA | bovine | F | 4 | 0 | NA | NA | NA | NA | 1.722 | -0.894 | 0.461 | 0.559 | 12.4444444444444 | NA |
| BOE 210 | MDG41405150 | NA | bovine | F | 1 | 0 | NA | NA | NA | NA | 1.722 | -0.894 | 0.461 | 0.559 | 12.4444444444444 | NA |
| BOE 209 | MDG41405150 | NA | bovine | F | 3 | 0 | NA | NA | NA | NA | 1.722 | -0.894 | 0.461 | 0.559 | 12.4444444444444 | NA |
| BOE 228 | MDG41405150 | NA | bovine | F | 3 | 1 | NA | NA | NA | NA | 1.722 | -0.894 | 0.461 | 0.559 | 12.4444444444444 | NA |
| BOE 235 | MDG41405170 | NA | bovine | M | 8 | 0 | NA | NA | NA | NA | 1.019 | -0.697 | 0.729 | 0.296 | 13.7692307692307 | NA |
| BOE 281 | MDG41405170 | NA | bovine | F | 3 | 0 | NA | NA | NA | NA | 1.019 | -0.697 | 0.729 | 0.296 | 13.7692307692307 | NA |
| BOE 230 | MDG41405170 | NA | bovine | F | 2 | 0 | NA | NA | NA | NA | 1.019 | -0.697 | 0.729 | 0.296 | 13.7692307692307 | NA |
| BOE 280 | MDG41405170 | NA | bovine | F | 2 | 1 | NA | NA | NA | NA | 1.019 | -0.697 | 0.729 | 0.296 | 13.7692307692307 | NA |
| BOE 232 | MDG41405170 | NA | bovine | M | 10 | 1 | NA | NA | NA | NA | 1.019 | -0.697 | 0.729 | 0.296 | 13.7692307692307 | NA |
| BOE 233 | MDG41405170 | NA | bovine | M | 7 | 0 | NA | NA | NA | NA | 1.019 | -0.697 | 0.729 | 0.296 | 13.7692307692307 | NA |
| BOE 282 | MDG41405170 | NA | bovine | M | 4 | 1 | NA | NA | NA | NA | 1.019 | -0.697 | 0.729 | 0.296 | 13.7692307692307 | NA |
| BOE 234 | MDG41405170 | NA | bovine | M | 7 | 0 | NA | NA | NA | NA | 1.019 | -0.697 | 0.729 | 0.296 | 13.7692307692307 | NA |
| BOE 207 | MDG41405170 | NA | bovine | M | 3 | 0 | NA | NA | NA | NA | 1.019 | -0.697 | 0.729 | 0.296 | 13.7692307692307 | NA |
| BOE 236 | MDG41405170 | NA | bovine | M | 7 | 1 | NA | NA | NA | NA | 1.019 | -0.697 | 0.729 | 0.296 | 13.7692307692307 | NA |
| BOE 286 | MDG41405170 | NA | bovine | F | 6 | 0 | NA | NA | NA | NA | 1.019 | -0.697 | 0.729 | 0.296 | 13.7692307692307 | NA |
| BOE 284 | MDG41405170 | NA | bovine | F | 7 | 0 | NA | NA | NA | NA | 1.019 | -0.697 | 0.729 | 0.296 | 13.7692307692307 | NA |
| BOE 283 | MDG41405170 | NA | bovine | F | 6 | 0 | NA | NA | NA | NA | 1.019 | -0.697 | 0.729 | 0.296 | 13.7692307692307 | NA |
| BOE 237 | MDG41405170 | NA | bovine | M | 2 | 0 | NA | NA | NA | NA | 1.019 | -0.697 | 0.729 | 0.296 | 13.7692307692307 | NA |
| BOE 238 | MDG41405170 | NA | bovine | M | 5 | 0 | NA | NA | NA | NA | 1.019 | -0.697 | 0.729 | 0.296 | 13.7692307692307 | NA |
| BOE 239 | MDG41405170 | NA | bovine | M | 3 | 0 | NA | NA | NA | NA | 1.019 | -0.697 | 0.729 | 0.296 | 13.7692307692307 | NA |
| BOE 278 | MDG41405170 | NA | bovine | F | 2 | 0 | NA | NA | NA | NA | 1.019 | -0.697 | 0.729 | 0.296 | 13.7692307692307 | NA |
| BOE 285 | MDG41405170 | NA | bovine | F | 4 | 0 | NA | NA | NA | NA | 1.019 | -0.697 | 0.729 | 0.296 | 13.7692307692307 | NA |
| BOE 208 | MDG41405170 | NA | bovine | M | 3 | 0 | NA | NA | NA | NA | 1.019 | -0.697 | 0.729 | 0.296 | 13.7692307692307 | NA |
| BOE 289 | MDG41405170 | NA | bovine | F | 4 | 0 | NA | NA | NA | NA | 1.019 | -0.697 | 0.729 | 0.296 | 13.7692307692307 | NA |
| BOE 287 | MDG41405170 | NA | bovine | F | 4 | 1 | NA | NA | NA | NA | 1.019 | -0.697 | 0.729 | 0.296 | 13.7692307692307 | NA |
| BOE 288 | MDG41405170 | NA | bovine | F | 5 | 0 | NA | NA | NA | NA | 1.019 | -0.697 | 0.729 | 0.296 | 13.7692307692307 | NA |
| BOE 231 | MDG41405170 | NA | bovine | F | 7 | 1 | NA | NA | NA | NA | 1.019 | -0.697 | 0.729 | 0.296 | 13.7692307692307 | NA |
| MAND 020 | MDG42410010 | NA | bovine | M | 4 | 0 | NA | NA | NA | NA | 1.086 | -0.076 | 0.297 | 0.127 | 38 | NA |
| MAND 038 | MDG42410010 | NA | bovine | F | 5 | 0 | NA | NA | NA | NA | 1.086 | -0.076 | 0.297 | 0.127 | 38 | NA |
| MAND 039 | MDG42410010 | NA | bovine | F | 1 | 0 | NA | NA | NA | NA | 1.086 | -0.076 | 0.297 | 0.127 | 38 | NA |
| MAND 063 | MDG42410010 | NA | bovine | M | 2 | 0 | NA | NA | NA | NA | 1.086 | -0.076 | 0.297 | 0.127 | 38 | NA |
| MAND 025 | MDG42410010 | NA | bovine | M | 4 | 0 | NA | NA | NA | NA | 1.086 | -0.076 | 0.297 | 0.127 | 38 | NA |
| MAND 024 | MDG42410010 | NA | bovine | M | 2 | 0 | NA | NA | NA | NA | 1.086 | -0.076 | 0.297 | 0.127 | 38 | NA |
| MAND 094 | MDG42410010 | NA | bovine | F | 8 | 1 | NA | NA | NA | NA | 1.086 | -0.076 | 0.297 | 0.127 | 38 | NA |
| MAND 023 | MDG42410010 | NA | bovine | M | 7 | 1 | NA | NA | NA | NA | 1.086 | -0.076 | 0.297 | 0.127 | 38 | NA |
| MAND 067 | MDG42410010 | NA | bovine | M | 7 | 1 | NA | NA | NA | NA | 1.086 | -0.076 | 0.297 | 0.127 | 38 | NA |
| MAND 021 | MDG42410010 | NA | bovine | M | 9 | 0 | NA | NA | NA | NA | 1.086 | -0.076 | 0.297 | 0.127 | 38 | NA |
| MAND 041 | MDG42410010 | NA | bovine | M | 4 | 1 | NA | NA | NA | NA | 1.086 | -0.076 | 0.297 | 0.127 | 38 | NA |
| MAND 005 | MDG42410010 | NA | bovine | M | 4 | 0 | NA | NA | NA | NA | 1.086 | -0.076 | 0.297 | 0.127 | 38 | NA |
| MAND 093 | MDG42410010 | NA | bovine | M | 8 | 0 | NA | NA | NA | NA | 1.086 | -0.076 | 0.297 | 0.127 | 38 | NA |
| MAND 022 | MDG42410010 | NA | bovine | M | 5 | 0 | NA | NA | NA | NA | 1.086 | -0.076 | 0.297 | 0.127 | 38 | NA |
| MAND 099 | MDG42410030 | NA | bovine | M | 1 | 1 | NA | NA | NA | NA | 0.832 | -0.095 | -0.036 | -0.222 | 35.9090909090909 | NA |
| MAND 078 | MDG42410030 | NA | bovine | M | 9 | 0 | NA | NA | NA | NA | 0.832 | -0.095 | -0.036 | -0.222 | 35.9090909090909 | NA |
| MAND 070 | MDG42410030 | NA | bovine | F | 1 | 0 | NA | NA | NA | NA | 0.832 | -0.095 | -0.036 | -0.222 | 35.9090909090909 | NA |
| MAND 068 | MDG42410030 | NA | bovine | M | 8 | 0 | NA | NA | NA | NA | 0.832 | -0.095 | -0.036 | -0.222 | 35.9090909090909 | NA |
| MAND 047 | MDG42410030 | NA | bovine | M | 5 | 0 | NA | NA | NA | NA | 0.832 | -0.095 | -0.036 | -0.222 | 35.9090909090909 | NA |
| MAND 056 | MDG42410030 | NA | bovine | F | 2 | 0 | NA | NA | NA | NA | 0.832 | -0.095 | -0.036 | -0.222 | 35.9090909090909 | NA |
| MAND 008 | MDG42410030 | NA | bovine | M | 1 | 1 | NA | NA | NA | NA | 0.832 | -0.095 | -0.036 | -0.222 | 35.9090909090909 | NA |
| MAND 015 | MDG42410030 | NA | bovine | M | 10 | 0 | NA | NA | NA | NA | 0.832 | -0.095 | -0.036 | -0.222 | 35.9090909090909 | NA |
| MAND 061 | MDG42410030 | NA | bovine | M | 2 | 0 | NA | NA | NA | NA | 0.832 | -0.095 | -0.036 | -0.222 | 35.9090909090909 | NA |
| MAND 072 | MDG42410030 | NA | bovine | M | 5 | 0 | NA | NA | NA | NA | 0.832 | -0.095 | -0.036 | -0.222 | 35.9090909090909 | NA |
| MAND 095 | MDG42410050 | NA | bovine | M | 6 | 0 | NA | NA | NA | NA | 0.120 | 0.423 | 0.228 | 0.014 | 32.875 | NA |
| MAND 051 | MDG42410050 | NA | bovine | M | 2 | 1 | NA | NA | NA | NA | 0.120 | 0.423 | 0.228 | 0.014 | 32.875 | NA |
| MAND 010 | MDG42410050 | NA | bovine | M | 8 | 0 | NA | NA | NA | NA | 0.120 | 0.423 | 0.228 | 0.014 | 32.875 | NA |
| MAND 027 | MDG42410050 | NA | bovine | M | 5 | 1 | NA | NA | NA | NA | 0.120 | 0.423 | 0.228 | 0.014 | 32.875 | NA |
| MAND 034 | MDG42410050 | NA | bovine | F | 6 | 0 | NA | NA | NA | NA | 0.120 | 0.423 | 0.228 | 0.014 | 32.875 | NA |
| MAND 096 | MDG42410050 | NA | bovine | F | 4 | 1 | NA | NA | NA | NA | 0.120 | 0.423 | 0.228 | 0.014 | 32.875 | NA |
| MAND 040 | MDG42410050 | NA | bovine | M | 7 | 1 | NA | NA | NA | NA | 0.120 | 0.423 | 0.228 | 0.014 | 32.875 | NA |
| MAND 045 | MDG42410050 | NA | bovine | F | 5 | 0 | NA | NA | NA | NA | 0.120 | 0.423 | 0.228 | 0.014 | 32.875 | NA |
| MAND 048 | MDG42410050 | NA | bovine | F | 3 | 0 | NA | NA | NA | NA | 0.120 | 0.423 | 0.228 | 0.014 | 32.875 | NA |
| MAND 007 | MDG42410050 | NA | bovine | M | 6 | 1 | NA | NA | NA | NA | 0.120 | 0.423 | 0.228 | 0.014 | 32.875 | NA |
| MAND 050 | MDG42410050 | NA | bovine | M | 1 | 0 | NA | NA | NA | NA | 0.120 | 0.423 | 0.228 | 0.014 | 32.875 | NA |

| ID | PCODE | site2 | sp | gender | age | IgG | contact_ruminant | contact_milk | contact_fresh_fluid | profession | fact1 | fact2 | fact3 | fact4 | cattle_density | habitat |
| --- | --- | --- | --- | --- | --- | --- | --- | --- | --- | --- | --- | --- | --- | --- | --- | --- |
| MAND 028 | MDG42410050 | NA | bovine | M | 9 | 1 | NA | NA | NA | NA | 0.120 | 0.423 | 0.228 | 0.014 | 32.875 | NA |
| MAND 052 | MDG42410050 | NA | bovine | M | 1 | 0 | NA | NA | NA | NA | 0.120 | 0.423 | 0.228 | 0.014 | 32.875 | NA |
| MAND 053 | MDG42410050 | NA | bovine | M | 3 | 0 | NA | NA | NA | NA | 0.120 | 0.423 | 0.228 | 0.014 | 32.875 | NA |
| MAND 060 | MDG42410050 | NA | bovine | M | 3 | 0 | NA | NA | NA | NA | 0.120 | 0.423 | 0.228 | 0.014 | 32.875 | NA |
| MAND 062 | MDG42410050 | NA | bovine | F | 4 | 0 | NA | NA | NA | NA | 0.120 | 0.423 | 0.228 | 0.014 | 32.875 | NA |
| MAND 064 | MDG42410050 | NA | bovine | M | 3 | 0 | NA | NA | NA | NA | 0.120 | 0.423 | 0.228 | 0.014 | 32.875 | NA |
| MAND 071 | MDG42410050 | NA | bovine | M | 4 | 0 | NA | NA | NA | NA | 0.120 | 0.423 | 0.228 | 0.014 | 32.875 | NA |
| MAND 075 | MDG42410050 | NA | bovine | M | 8 | 0 | NA | NA | NA | NA | 0.120 | 0.423 | 0.228 | 0.014 | 32.875 | NA |
| MAND 080 | MDG42410050 | NA | bovine | M | 2 | 0 | NA | NA | NA | NA | 0.120 | 0.423 | 0.228 | 0.014 | 32.875 | NA |
| MAND 049 | MDG42410050 | NA | bovine | F | 6 | 0 | NA | NA | NA | NA | 0.120 | 0.423 | 0.228 | 0.014 | 32.875 | NA |
| MAND 098 | MDG42410050 | NA | bovine | M | 7 | 0 | NA | NA | NA | NA | 0.120 | 0.423 | 0.228 | 0.014 | 32.875 | NA |
| MAND 077 | MDG42410070 | NA | bovine | M | 3 | 0 | NA | NA | NA | NA | 0.158 | -0.134 | 0.345 | -0.603 | 33.5454545454545 | NA |
| MAND 083 | MDG42410111 | NA | bovine | M | 5 | 1 | NA | NA | NA | NA | 0.097 | 0.097 | 0.488 | -0.728 | 29.2307692307692 | NA |
| MAND 092 | MDG42410111 | NA | bovine | F | 1 | 0 | NA | NA | NA | NA | 0.097 | 0.097 | 0.488 | -0.728 | 29.2307692307692 | NA |
| MAND 091 | MDG42410111 | NA | bovine | M | 6 | 0 | NA | NA | NA | NA | 0.097 | 0.097 | 0.488 | -0.728 | 29.2307692307692 | NA |
| MAND 088 | MDG42410111 | NA | bovine | M | 8 | 0 | NA | NA | NA | NA | 0.097 | 0.097 | 0.488 | -0.728 | 29.2307692307692 | NA |
| MAND 087 | MDG42410111 | NA | bovine | M | 8 | 0 | NA | NA | NA | NA | 0.097 | 0.097 | 0.488 | -0.728 | 29.2307692307692 | NA |
| MAND 086 | MDG42410111 | NA | bovine | F | 7 | 0 | NA | NA | NA | NA | 0.097 | 0.097 | 0.488 | -0.728 | 29.2307692307692 | NA |
| MAND 026 | MDG42410111 | NA | bovine | M | 8 | 1 | NA | NA | NA | NA | 0.097 | 0.097 | 0.488 | -0.728 | 29.2307692307692 | NA |
| MAND 085 | MDG42410111 | NA | bovine | M | 3 | 0 | NA | NA | NA | NA | 0.097 | 0.097 | 0.488 | -0.728 | 29.2307692307692 | NA |
| MAND 084 | MDG42410111 | NA | bovine | M | 8 | 0 | NA | NA | NA | NA | 0.097 | 0.097 | 0.488 | -0.728 | 29.2307692307692 | NA |
| MAND 065 | MDG42410130 | NA | bovine | M | 5 | 0 | NA | NA | NA | NA | 0.303 | -0.266 | 0.307 | -0.481 | 34.1428571428571 | NA |
| MAND 082 | MDG42410130 | NA | bovine | M | 6 | 0 | NA | NA | NA | NA | 0.303 | -0.266 | 0.307 | -0.481 | 34.1428571428571 | NA |
| MAND 059 | MDG42410130 | NA | bovine | M | 5 | 1 | NA | NA | NA | NA | 0.303 | -0.266 | 0.307 | -0.481 | 34.1428571428571 | NA |
| MAND 030 | MDG42410171 | NA | bovine | M | 4 | 0 | NA | NA | NA | NA | 0.361 | 0.017 | 0.478 | -0.773 | 31.5555555555555 | NA |
| MAND 069 | MDG42410191 | NA | bovine | M | 4 | 0 | NA | NA | NA | NA | 0.150 | 0.011 | 0.731 | -0.709 | 19.4285714285714 | NA |
| MAND 097 | MDG42410192 | NA | bovine | M | 6 | 1 | NA | NA | NA | NA | -0.038 | 0.044 | 0.533 | -0.650 | 26.1428571428571 | NA |
| MAND 012 | MDG42410290 | NA | bovine | F | 4 | 0 | NA | NA | NA | NA | 0.169 | -0.316 | 0.790 | -0.407 | 32.7297297297297 | NA |
| MAIN _045 | MDG44421010 | NA | bovine | F | 6 | 0 | NA | NA | NA | NA | 1.004 | 0.372 | 1.630 | 0.228 | 12.375 | NA |
| MAIN _042 | MDG44421010 | NA | bovine | F | 1 | 0 | NA | NA | NA | NA | 1.004 | 0.372 | 1.630 | 0.228 | 12.375 | NA |
| MAIN _032 | MDG44421010 | NA | bovine | F | 7 | 0 | NA | NA | NA | NA | 1.004 | 0.372 | 1.630 | 0.228 | 12.375 | NA |
| MAIN _001 | MDG44421010 | NA | bovine | M | 1 | 0 | NA | NA | NA | NA | 1.004 | 0.372 | 1.630 | 0.228 | 12.375 | NA |
| MAIN _098 | MDG44421010 | NA | bovine | F | 2 | 0 | NA | NA | NA | NA | 1.004 | 0.372 | 1.630 | 0.228 | 12.375 | NA |
| MAIN _099 | MDG44421010 | NA | bovine | F | 9 | 0 | NA | NA | NA | NA | 1.004 | 0.372 | 1.630 | 0.228 | 12.375 | NA |
| MAIN _100 | MDG44421010 | NA | bovine | M | 1 | 0 | NA | NA | NA | NA | 1.004 | 0.372 | 1.630 | 0.228 | 12.375 | NA |
| MAIN _096 | MDG44421010 | NA | bovine | F | 7 | 1 | NA | NA | NA | NA | 1.004 | 0.372 | 1.630 | 0.228 | 12.375 | NA |
| MAIN _095 | MDG44421010 | NA | bovine | F | 9 | 0 | NA | NA | NA | NA | 1.004 | 0.372 | 1.630 | 0.228 | 12.375 | NA |
| MAIN _094 | MDG44421010 | NA | bovine | F | 7 | 0 | NA | NA | NA | NA | 1.004 | 0.372 | 1.630 | 0.228 | 12.375 | NA |
| MAIN _021 | MDG44421010 | NA | bovine | F | 1 | 0 | NA | NA | NA | NA | 1.004 | 0.372 | 1.630 | 0.228 | 12.375 | NA |
| MAIN _092 | MDG44421010 | NA | bovine | F | 3 | 0 | NA | NA | NA | NA | 1.004 | 0.372 | 1.630 | 0.228 | 12.375 | NA |
| MAIN _024 | MDG44421010 | NA | bovine | M | 4 | 0 | NA | NA | NA | NA | 1.004 | 0.372 | 1.630 | 0.228 | 12.375 | NA |
| MAIN _002 | MDG44421010 | NA | bovine | F | 8 | 0 | NA | NA | NA | NA | 1.004 | 0.372 | 1.630 | 0.228 | 12.375 | NA |
| MAIN _003 | MDG44421010 | NA | bovine | M | 3 | 0 | NA | NA | NA | NA | 1.004 | 0.372 | 1.630 | 0.228 | 12.375 | NA |
| MAIN _004 | MDG44421010 | NA | bovine | F | 2 | 0 | NA | NA | NA | NA | 1.004 | 0.372 | 1.630 | 0.228 | 12.375 | NA |
| MAIN _009 | MDG44421010 | NA | bovine | F | 8 | 1 | NA | NA | NA | NA | 1.004 | 0.372 | 1.630 | 0.228 | 12.375 | NA |
| MAIN _015 | MDG44421010 | NA | bovine | F | 9 | 1 | NA | NA | NA | NA | 1.004 | 0.372 | 1.630 | 0.228 | 12.375 | NA |
| MAIN _097 | MDG44421010 | NA | bovine | F | 10 | 1 | NA | NA | NA | NA | 1.004 | 0.372 | 1.630 | 0.228 | 12.375 | NA |
| MAIN _018 | MDG44421010 | NA | bovine | M | 6 | 0 | NA | NA | NA | NA | 1.004 | 0.372 | 1.630 | 0.228 | 12.375 | NA |
| MAIN _093 | MDG44421010 | NA | bovine | F | 1 | 0 | NA | NA | NA | NA | 1.004 | 0.372 | 1.630 | 0.228 | 12.375 | NA |
| MAIN _068 | MDG44421010 | NA | bovine | F | 8 | 0 | NA | NA | NA | NA | 1.004 | 0.372 | 1.630 | 0.228 | 12.375 | NA |
| MAIN _091 | MDG44421010 | NA | bovine | F | 7 | 0 | NA | NA | NA | NA | 1.004 | 0.372 | 1.630 | 0.228 | 12.375 | NA |
| MAIN _060 | MDG44421010 | NA | bovine | F | 8 | 1 | NA | NA | NA | NA | 1.004 | 0.372 | 1.630 | 0.228 | 12.375 | NA |
| MAIN _071 | MDG44421010 | NA | bovine | F | 9 | 1 | NA | NA | NA | NA | 1.004 | 0.372 | 1.630 | 0.228 | 12.375 | NA |
| MAIN _083 | MDG44421070 | NA | bovine | M | 7 | 0 | NA | NA | NA | NA | 1.295 | 0.224 | 2.318 | 0.163 | 17.3999999999999 | NA |
| MAIN _084 | MDG44421070 | NA | bovine | F | 8 | 0 | NA | NA | NA | NA | 1.295 | 0.224 | 2.318 | 0.163 | 17.3999999999999 | NA |
| MAIN _079 | MDG44421070 | NA | bovine | F | 1 | 0 | NA | NA | NA | NA | 1.295 | 0.224 | 2.318 | 0.163 | 17.3999999999999 | NA |
| MAIN _082 | MDG44421070 | NA | bovine | F | 4 | 1 | NA | NA | NA | NA | 1.295 | 0.224 | 2.318 | 0.163 | 17.3999999999999 | NA |
| MAIN _078 | MDG44421070 | NA | bovine | M | 2 | 0 | NA | NA | NA | NA | 1.295 | 0.224 | 2.318 | 0.163 | 17.3999999999999 | NA |
| MAIN _077 | MDG44421070 | NA | bovine | M | 3 | 1 | NA | NA | NA | NA | 1.295 | 0.224 | 2.318 | 0.163 | 17.3999999999999 | NA |
| MAIN _076 | MDG44421070 | NA | bovine | M | 7 | 1 | NA | NA | NA | NA | 1.295 | 0.224 | 2.318 | 0.163 | 17.3999999999999 | NA |
| MAIN _081 | MDG44421070 | NA | bovine | F | 9 | 1 | NA | NA | NA | NA | 1.295 | 0.224 | 2.318 | 0.163 | 17.3999999999999 | NA |
| MAIN _085 | MDG44421070 | NA | bovine | F | 7 | 1 | NA | NA | NA | NA | 1.295 | 0.224 | 2.318 | 0.163 | 17.3999999999999 | NA |
| MAIN _090 | MDG44421070 | NA | bovine | F | 1 | 0 | NA | NA | NA | NA | 1.295 | 0.224 | 2.318 | 0.163 | 17.3999999999999 | NA |

| ID | PCODE | site2 | sp | gender | age | IgG | contact_ruminant | contact_milk | contact_fresh_fluid | profession | fact1 | fact2 | fact3 | fact4 | cattle_density | habitat |
| --- | --- | --- | --- | --- | --- | --- | --- | --- | --- | --- | --- | --- | --- | --- | --- | --- |
| MAIN _086 | MDG44421070 | NA | bovine | F | 6 | 1 | NA | NA | NA | NA | 1.295 | 0.224 | 2.318 | 0.163 | 17.3999999999999 | NA |
| MAIN _087 | MDG44421070 | NA | bovine | F | 7 | 1 | NA | NA | NA | NA | 1.295 | 0.224 | 2.318 | 0.163 | 17.3999999999999 | NA |
| MAIN _088 | MDG44421070 | NA | bovine | M | 5 | 0 | NA | NA | NA | NA | 1.295 | 0.224 | 2.318 | 0.163 | 17.3999999999999 | NA |
| MAIN _089 | MDG44421070 | NA | bovine | F | 7 | 0 | NA | NA | NA | NA | 1.295 | 0.224 | 2.318 | 0.163 | 17.3999999999999 | NA |
| MAIN _080 | MDG44421070 | NA | bovine | F | 6 | 0 | NA | NA | NA | NA | 1.295 | 0.224 | 2.318 | 0.163 | 17.3999999999999 | NA |
| MAIN _075 | MDG44421070 | NA | bovine | F | 1 | 0 | NA | NA | NA | NA | 1.295 | 0.224 | 2.318 | 0.163 | 17.3999999999999 | NA |
| MAIN _074 | MDG44421070 | NA | bovine | M | 5 | 0 | NA | NA | NA | NA | 1.295 | 0.224 | 2.318 | 0.163 | 17.3999999999999 | NA |
| MAIN _073 | MDG44421070 | NA | bovine | M | 4 | 0 | NA | NA | NA | NA | 1.295 | 0.224 | 2.318 | 0.163 | 17.3999999999999 | NA |
| MAIN _072 | MDG44421070 | NA | bovine | M | 2 | 0 | NA | NA | NA | NA | 1.295 | 0.224 | 2.318 | 0.163 | 17.3999999999999 | NA |
| MAIN _013 | MDG44421110 | NA | bovine | F | 10 | 0 | NA | NA | NA | NA | 1.231 | -0.027 | 1.840 | -0.006 | 15.96 | NA |
| MAIN _014 | MDG44421110 | NA | bovine | M | 1 | 0 | NA | NA | NA | NA | 1.231 | -0.027 | 1.840 | -0.006 | 15.96 | NA |
| MAIN _058 | MDG44421110 | NA | bovine | M | 2 | 0 | NA | NA | NA | NA | 1.231 | -0.027 | 1.840 | -0.006 | 15.96 | NA |
| MAIN _061 | MDG44421110 | NA | bovine | F | 8 | 0 | NA | NA | NA | NA | 1.231 | -0.027 | 1.840 | -0.006 | 15.96 | NA |
| MAIN _059 | MDG44421110 | NA | bovine | F | 5 | 1 | NA | NA | NA | NA | 1.231 | -0.027 | 1.840 | -0.006 | 15.96 | NA |
| MAIN _057 | MDG44421110 | NA | bovine | F | 3 | 0 | NA | NA | NA | NA | 1.231 | -0.027 | 1.840 | -0.006 | 15.96 | NA |
| MAIN _062 | MDG44421110 | NA | bovine | F | 5 | 0 | NA | NA | NA | NA | 1.231 | -0.027 | 1.840 | -0.006 | 15.96 | NA |
| MAIN _063 | MDG44421110 | NA | bovine | F | 10 | 1 | NA | NA | NA | NA | 1.231 | -0.027 | 1.840 | -0.006 | 15.96 | NA |
| MAIN _065 | MDG44421110 | NA | bovine | F | 8 | 1 | NA | NA | NA | NA | 1.231 | -0.027 | 1.840 | -0.006 | 15.96 | NA |
| MAIN _012 | MDG44421110 | NA | bovine | M | 3 | 1 | NA | NA | NA | NA | 1.231 | -0.027 | 1.840 | -0.006 | 15.96 | NA |
| MAIN _010 | MDG44421110 | NA | bovine | F | 10 | 1 | NA | NA | NA | NA | 1.231 | -0.027 | 1.840 | -0.006 | 15.96 | NA |
| MAIN _016 | MDG44421110 | NA | bovine | M | 1 | 0 | NA | NA | NA | NA | 1.231 | -0.027 | 1.840 | -0.006 | 15.96 | NA |
| MAIN _007 | MDG44421110 | NA | bovine | M | 3 | 0 | NA | NA | NA | NA | 1.231 | -0.027 | 1.840 | -0.006 | 15.96 | NA |
| MAIN _064 | MDG44421110 | NA | bovine | F | 9 | 1 | NA | NA | NA | NA | 1.231 | -0.027 | 1.840 | -0.006 | 15.96 | NA |
| MAIN _067 | MDG44421110 | NA | bovine | F | 12 | 1 | NA | NA | NA | NA | 1.231 | -0.027 | 1.840 | -0.006 | 15.96 | NA |
| MAIN _069 | MDG44421110 | NA | bovine | F | 8 | 1 | NA | NA | NA | NA | 1.231 | -0.027 | 1.840 | -0.006 | 15.96 | NA |
| MAIN _070 | MDG44421110 | NA | bovine | F | 10 | 1 | NA | NA | NA | NA | 1.231 | -0.027 | 1.840 | -0.006 | 15.96 | NA |
| MAIN _008 | MDG44421110 | NA | bovine | F | 4 | 0 | NA | NA | NA | NA | 1.231 | -0.027 | 1.840 | -0.006 | 15.96 | NA |
| MAIN _050 | MDG44421110 | NA | bovine | F | 7 | 1 | NA | NA | NA | NA | 1.231 | -0.027 | 1.840 | -0.006 | 15.96 | NA |
| MAIN _039 | MDG44421110 | NA | bovine | M | 4 | 0 | NA | NA | NA | NA | 1.231 | -0.027 | 1.840 | -0.006 | 15.96 | NA |
| MAIN _040 | MDG44421110 | NA | bovine | F | 7 | 0 | NA | NA | NA | NA | 1.231 | -0.027 | 1.840 | -0.006 | 15.96 | NA |
| MAIN _041 | MDG44421110 | NA | bovine | M | 2 | 0 | NA | NA | NA | NA | 1.231 | -0.027 | 1.840 | -0.006 | 15.96 | NA |
| MAIN _043 | MDG44421110 | NA | bovine | M | 3 | 0 | NA | NA | NA | NA | 1.231 | -0.027 | 1.840 | -0.006 | 15.96 | NA |
| MAIN _044 | MDG44421110 | NA | bovine | M | 5 | 0 | NA | NA | NA | NA | 1.231 | -0.027 | 1.840 | -0.006 | 15.96 | NA |
| MAIN _046 | MDG44421110 | NA | bovine | F | 12 | 0 | NA | NA | NA | NA | 1.231 | -0.027 | 1.840 | -0.006 | 15.96 | NA |
| MAIN _047 | MDG44421110 | NA | bovine | F | 9 | 1 | NA | NA | NA | NA | 1.231 | -0.027 | 1.840 | -0.006 | 15.96 | NA |
| MAIN _038 | MDG44421110 | NA | bovine | M | 5 | 0 | NA | NA | NA | NA | 1.231 | -0.027 | 1.840 | -0.006 | 15.96 | NA |
| MAIN _017 | MDG44421110 | NA | bovine | F | 12 | 1 | NA | NA | NA | NA | 1.231 | -0.027 | 1.840 | -0.006 | 15.96 | NA |
| MAIN _049 | MDG44421110 | NA | bovine | M | 2 | 0 | NA | NA | NA | NA | 1.231 | -0.027 | 1.840 | -0.006 | 15.96 | NA |
| MAIN _066 | MDG44421110 | NA | bovine | F | 10 | 1 | NA | NA | NA | NA | 1.231 | -0.027 | 1.840 | -0.006 | 15.96 | NA |
| MAIN _051 | MDG44421110 | NA | bovine | F | 1 | 1 | NA | NA | NA | NA | 1.231 | -0.027 | 1.840 | -0.006 | 15.96 | NA |
| MAIN _052 | MDG44421110 | NA | bovine | F | 6 | 1 | NA | NA | NA | NA | 1.231 | -0.027 | 1.840 | -0.006 | 15.96 | NA |
| MAIN _053 | MDG44421110 | NA | bovine | F | 10 | 0 | NA | NA | NA | NA | 1.231 | -0.027 | 1.840 | -0.006 | 15.96 | NA |
| MAIN _054 | MDG44421110 | NA | bovine | F | 10 | 1 | NA | NA | NA | NA | 1.231 | -0.027 | 1.840 | -0.006 | 15.96 | NA |
| MAIN _055 | MDG44421110 | NA | bovine | M | 1 | 0 | NA | NA | NA | NA | 1.231 | -0.027 | 1.840 | -0.006 | 15.96 | NA |
| MAIN _056 | MDG44421110 | NA | bovine | F | 4 | 1 | NA | NA | NA | NA | 1.231 | -0.027 | 1.840 | -0.006 | 15.96 | NA |
| MAIN _048 | MDG44421110 | NA | bovine | F | 8 | 0 | NA | NA | NA | NA | 1.231 | -0.027 | 1.840 | -0.006 | 15.96 | NA |
| MAIN _027 | MDG44421110 | NA | bovine | M | 1 | 0 | NA | NA | NA | NA | 1.231 | -0.027 | 1.840 | -0.006 | 15.96 | NA |
| MAIN _037 | MDG44421110 | NA | bovine | F | 6 | 0 | NA | NA | NA | NA | 1.231 | -0.027 | 1.840 | -0.006 | 15.96 | NA |
| MAIN _019 | MDG44421110 | NA | bovine | M | 1 | 1 | NA | NA | NA | NA | 1.231 | -0.027 | 1.840 | -0.006 | 15.96 | NA |
| MAIN _020 | MDG44421110 | NA | bovine | F | 3 | 0 | NA | NA | NA | NA | 1.231 | -0.027 | 1.840 | -0.006 | 15.96 | NA |
| MAIN _022 | MDG44421110 | NA | bovine | F | 5 | 1 | NA | NA | NA | NA | 1.231 | -0.027 | 1.840 | -0.006 | 15.96 | NA |
| MAIN _023 | MDG44421110 | NA | bovine | M | 1 | 0 | NA | NA | NA | NA | 1.231 | -0.027 | 1.840 | -0.006 | 15.96 | NA |
| MAIN _026 | MDG44421110 | NA | bovine | F | 8 | 1 | NA | NA | NA | NA | 1.231 | -0.027 | 1.840 | -0.006 | 15.96 | NA |
| MAIN _028 | MDG44421110 | NA | bovine | F | 1 | 0 | NA | NA | NA | NA | 1.231 | -0.027 | 1.840 | -0.006 | 15.96 | NA |
| MAIN _029 | MDG44421110 | NA | bovine | M | 1 | 0 | NA | NA | NA | NA | 1.231 | -0.027 | 1.840 | -0.006 | 15.96 | NA |
| MAIN _030 | MDG44421110 | NA | bovine | M | 4 | 0 | NA | NA | NA | NA | 1.231 | -0.027 | 1.840 | -0.006 | 15.96 | NA |
| MAIN _035 | MDG44421110 | NA | bovine | M | 4 | 1 | NA | NA | NA | NA | 1.231 | -0.027 | 1.840 | -0.006 | 15.96 | NA |
| MAIN _031 | MDG44421110 | NA | bovine | F | 8 | 1 | NA | NA | NA | NA | 1.231 | -0.027 | 1.840 | -0.006 | 15.96 | NA |
| MAIN _033 | MDG44421110 | NA | bovine | M | 4 | 1 | NA | NA | NA | NA | 1.231 | -0.027 | 1.840 | -0.006 | 15.96 | NA |
| MAIN _034 | MDG44421110 | NA | bovine | M | 3 | 1 | NA | NA | NA | NA | 1.231 | -0.027 | 1.840 | -0.006 | 15.96 | NA |
| MAIN _036 | MDG44421110 | NA | bovine | F | 8 | 1 | NA | NA | NA | NA | 1.231 | -0.027 | 1.840 | -0.006 | 15.96 | NA |
| MAIN _025 | MDG44421110 | NA | bovine | F | 1 | 0 | NA | NA | NA | NA | 1.231 | -0.027 | 1.840 | -0.006 | 15.96 | NA |
| TU2 089 | MDG51501006 | NA | bovine | F | 4 | 0 | NA | NA | NA | NA | 2.431 | 0.247 | -1.138 | -0.121 | 118 | NA |

| ID | PCODE | site2 | sp | gender | age | IgG | contact_ruminant | contact_milk | contact_fresh_fluid | profession | fact1 | fact2 | fact3 | fact4 | cattle_density | habitat |
| --- | --- | --- | --- | --- | --- | --- | --- | --- | --- | --- | --- | --- | --- | --- | --- | --- |
| ANK_085 | MDG51505011 | NA | bovine | F | 6 | 0 | NA | NA | NA | NA | 2.026 | -0.581 | -0.335 | -0.607 | 6 | NA |
| ANK_090 | MDG51505011 | NA | bovine | F | 10 | 0 | NA | NA | NA | NA | 2.026 | -0.581 | -0.335 | -0.607 | 6 | NA |
| ANK_106 | MDG51505011 | NA | bovine | M | 8 | 0 | NA | NA | NA | NA | 2.026 | -0.581 | -0.335 | -0.607 | 6 | NA |
| ANK_038 | MDG51505011 | NA | bovine | F | 3 | 1 | NA | NA | NA | NA | 2.026 | -0.581 | -0.335 | -0.607 | 6 | NA |
| ANK_037 | MDG51505011 | NA | bovine | M | 8 | 1 | NA | NA | NA | NA | 2.026 | -0.581 | -0.335 | -0.607 | 6 | NA |
| ANK_118 | MDG51505011 | NA | bovine | F | 10 | 1 | NA | NA | NA | NA | 2.026 | -0.581 | -0.335 | -0.607 | 6 | NA |
| ANK_107 | MDG51505011 | NA | bovine | F | 10 | 0 | NA | NA | NA | NA | 2.026 | -0.581 | -0.335 | -0.607 | 6 | NA |
| ANK_119 | MDG51505012 | NA | bovine | M | 7 | 0 | NA | NA | NA | NA | 1.988 | -0.554 | -0.426 | -0.492 | 7.25 | NA |
| ANK_068 | MDG51505012 | NA | bovine | F | 7 | 0 | NA | NA | NA | NA | 1.988 | -0.554 | -0.426 | -0.492 | 7.25 | NA |
| ANK_052 | MDG51505012 | NA | bovine | F | 4 | 0 | NA | NA | NA | NA | 1.988 | -0.554 | -0.426 | -0.492 | 7.25 | NA |
| ANK_021 | MDG51505012 | NA | bovine | M | 8 | 0 | NA | NA | NA | NA | 1.988 | -0.554 | -0.426 | -0.492 | 7.25 | NA |
| ANK_022 | MDG51505012 | NA | bovine | M | 7 | 0 | NA | NA | NA | NA | 1.988 | -0.554 | -0.426 | -0.492 | 7.25 | NA |
| ANK_023 | MDG51505012 | NA | bovine | F | 4 | 0 | NA | NA | NA | NA | 1.988 | -0.554 | -0.426 | -0.492 | 7.25 | NA |
| ANK_053 | MDG51505012 | NA | bovine | F | 5 | 0 | NA | NA | NA | NA | 1.988 | -0.554 | -0.426 | -0.492 | 7.25 | NA |
| ANK_024 | MDG51505012 | NA | bovine | F | 4 | 0 | NA | NA | NA | NA | 1.988 | -0.554 | -0.426 | -0.492 | 7.25 | NA |
| ANK_016 | MDG51505030 | NA | bovine | M | 8 | 0 | NA | NA | NA | NA | 2.249 | -0.660 | -0.449 | -0.447 | 5.18421052631578 | NA |
| ANK_082 | MDG51505030 | NA | bovine | F | 9 | 0 | NA | NA | NA | NA | 2.249 | -0.660 | -0.449 | -0.447 | 5.18421052631578 | NA |
| ANK_084 | MDG51505030 | NA | bovine | M | 10 | 0 | NA | NA | NA | NA | 2.249 | -0.660 | -0.449 | -0.447 | 5.18421052631578 | NA |
| ANK_017 | MDG51505030 | NA | bovine | M | 2 | 0 | NA | NA | NA | NA | 2.249 | -0.660 | -0.449 | -0.447 | 5.18421052631578 | NA |
| ANK_130 | MDG51505030 | NA | bovine | F | 5 | 0 | NA | NA | NA | NA | 2.249 | -0.660 | -0.449 | -0.447 | 5.18421052631578 | NA |
| ANK_018 | MDG51505030 | NA | bovine | F | 3 | 0 | NA | NA | NA | NA | 2.249 | -0.660 | -0.449 | -0.447 | 5.18421052631578 | NA |
| ANK_030 | MDG51505050 | NA | bovine | F | 7 | 0 | NA | NA | NA | NA | 1.909 | -0.351 | 0.038 | -0.674 | 7.02857142857142 | NA |
| ANK_028 | MDG51505050 | NA | bovine | F | 4 | 0 | NA | NA | NA | NA | 1.909 | -0.351 | 0.038 | -0.674 | 7.02857142857142 | NA |
| ANK_029 | MDG51505050 | NA | bovine | F | 6 | 0 | NA | NA | NA | NA | 1.909 | -0.351 | 0.038 | -0.674 | 7.02857142857142 | NA |
| ANK_067 | MDG51505050 | NA | bovine | M | 6 | 0 | NA | NA | NA | NA | 1.909 | -0.351 | 0.038 | -0.674 | 7.02857142857142 | NA |
| ANK_027 | MDG51505050 | NA | bovine | M | 3 | 0 | NA | NA | NA | NA | 1.909 | -0.351 | 0.038 | -0.674 | 7.02857142857142 | NA |
| ANK_026 | MDG51505050 | NA | bovine | M | 6 | 0 | NA | NA | NA | NA | 1.909 | -0.351 | 0.038 | -0.674 | 7.02857142857142 | NA |
| ANK_025 | MDG51505050 | NA | bovine | F | 8 | 0 | NA | NA | NA | NA | 1.909 | -0.351 | 0.038 | -0.674 | 7.02857142857142 | NA |
| ANK_049 | MDG51505050 | NA | bovine | M | 4 | 0 | NA | NA | NA | NA | 1.909 | -0.351 | 0.038 | -0.674 | 7.02857142857142 | NA |
| ANK_012 | MDG51505071 | NA | bovine | F | 8 | 0 | NA | NA | NA | NA | 2.176 | -0.944 | -0.507 | -0.446 | 5.45454545454545 | NA |
| ANK_009 | MDG51505071 | NA | bovine | F | 4 | 0 | NA | NA | NA | NA | 2.176 | -0.944 | -0.507 | -0.446 | 5.45454545454545 | NA |
| ANK_117 | MDG51505071 | NA | bovine | M | 4 | 0 | NA | NA | NA | NA | 2.176 | -0.944 | -0.507 | -0.446 | 5.45454545454545 | NA |
| ANK_086 | MDG51505071 | NA | bovine | F | 7 | 0 | NA | NA | NA | NA | 2.176 | -0.944 | -0.507 | -0.446 | 5.45454545454545 | NA |
| ANK_069 | MDG51505071 | NA | bovine | M | 8 | 0 | NA | NA | NA | NA | 2.176 | -0.944 | -0.507 | -0.446 | 5.45454545454545 | NA |
| ANK_011 | MDG51505071 | NA | bovine | M | 7 | 0 | NA | NA | NA | NA | 2.176 | -0.944 | -0.507 | -0.446 | 5.45454545454545 | NA |
| ANK_010 | MDG51505071 | NA | bovine | F | 6 | 0 | NA | NA | NA | NA | 2.176 | -0.944 | -0.507 | -0.446 | 5.45454545454545 | NA |
| ANK_032 | MDG51505072 | NA | bovine | F | 2 | 0 | NA | NA | NA | NA | 1.896 | -0.695 | -0.507 | -0.412 | 6.47368421052631 | NA |
| ANK_033 | MDG51505072 | NA | bovine | F | 5 | 1 | NA | NA | NA | NA | 1.896 | -0.695 | -0.507 | -0.412 | 6.47368421052631 | NA |
| ANK_031 | MDG51505072 | NA | bovine | M | 4 | 0 | NA | NA | NA | NA | 1.896 | -0.695 | -0.507 | -0.412 | 6.47368421052631 | NA |
| ANK_060 | MDG51505072 | NA | bovine | F | 2 | 0 | NA | NA | NA | NA | 1.896 | -0.695 | -0.507 | -0.412 | 6.47368421052631 | NA |
| ANK_081 | MDG51505072 | NA | bovine | F | 8 | 0 | NA | NA | NA | NA | 1.896 | -0.695 | -0.507 | -0.412 | 6.47368421052631 | NA |
| ANK_034 | MDG51505072 | NA | bovine | F | 4 | 1 | NA | NA | NA | NA | 1.896 | -0.695 | -0.507 | -0.412 | 6.47368421052631 | NA |
| TU2 107 | MDG51506030 | NA | bovine | F | 4 | 1 | NA | NA | NA | NA | 2.244 | 0.059 | -0.762 | -0.213 | 19.6999999999999 | NA |
| AMP 143 | MDG51507010 | NA | bovine | F | 4 | 0 | NA | NA | NA | NA | 1.707 | -0.245 | -0.974 | -0.937 | 16.9629629629629 | NA |
| AMP 115 | MDG51507010 | NA | bovine | F | 4 | 0 | NA | NA | NA | NA | 1.707 | -0.245 | -0.974 | -0.937 | 16.9629629629629 | NA |
| AMP 114 | MDG51507010 | NA | bovine | F | 1 | 0 | NA | NA | NA | NA | 1.707 | -0.245 | -0.974 | -0.937 | 16.9629629629629 | NA |
| AMP 113 | MDG51507010 | NA | bovine | M | 2 | 0 | NA | NA | NA | NA | 1.707 | -0.245 | -0.974 | -0.937 | 16.9629629629629 | NA |
| AMP 112 | MDG51507010 | NA | bovine | M | 1 | 0 | NA | NA | NA | NA | 1.707 | -0.245 | -0.974 | -0.937 | 16.9629629629629 | NA |
| AMP 147 | MDG51507010 | NA | bovine | M | 6 | 0 | NA | NA | NA | NA | 1.707 | -0.245 | -0.974 | -0.937 | 16.9629629629629 | NA |
| AMP 033 | MDG51507010 | NA | bovine | F | 3 | 0 | NA | NA | NA | NA | 1.707 | -0.245 | -0.974 | -0.937 | 16.9629629629629 | NA |
| AMP 146 | MDG51507010 | NA | bovine | F | 2 | 0 | NA | NA | NA | NA | 1.707 | -0.245 | -0.974 | -0.937 | 16.9629629629629 | NA |
| AMP 145 | MDG51507010 | NA | bovine | M | 2 | 0 | NA | NA | NA | NA | 1.707 | -0.245 | -0.974 | -0.937 | 16.9629629629629 | NA |
| AMP 034 | MDG51507010 | NA | bovine | F | 4 | 0 | NA | NA | NA | NA | 1.707 | -0.245 | -0.974 | -0.937 | 16.9629629629629 | NA |
| AMP 035 | MDG51507010 | NA | bovine | F | 3 | 0 | NA | NA | NA | NA | 1.707 | -0.245 | -0.974 | -0.937 | 16.9629629629629 | NA |
| AMP 195 | MDG51507010 | NA | bovine | F | 2 | 0 | NA | NA | NA | NA | 1.707 | -0.245 | -0.974 | -0.937 | 16.9629629629629 | NA |
| AMP 142 | MDG51507010 | NA | bovine | M | 1 | 0 | NA | NA | NA | NA | 1.707 | -0.245 | -0.974 | -0.937 | 16.9629629629629 | NA |
| AMP 141 | MDG51507010 | NA | bovine | M | 2 | 0 | NA | NA | NA | NA | 1.707 | -0.245 | -0.974 | -0.937 | 16.9629629629629 | NA |
| AMP 116 | MDG51507010 | NA | bovine | M | 2 | 0 | NA | NA | NA | NA | 1.707 | -0.245 | -0.974 | -0.937 | 16.9629629629629 | NA |
| AMP 122 | MDG51507010 | NA | bovine | F | 2 | 0 | NA | NA | NA | NA | 1.707 | -0.245 | -0.974 | -0.937 | 16.9629629629629 | NA |
| AMP 139 | MDG51507010 | NA | bovine | M | 4 | 0 | NA | NA | NA | NA | 1.707 | -0.245 | -0.974 | -0.937 | 16.9629629629629 | NA |
| AMP 199 | MDG51507010 | NA | bovine | M | 1 | 0 | NA | NA | NA | NA | 1.707 | -0.245 | -0.974 | -0.937 | 16.9629629629629 | NA |
| AMP 031 | MDG51507010 | NA | bovine | F | 3 | 0 | NA | NA | NA | NA | 1.707 | -0.245 | -0.974 | -0.937 | 16.9629629629629 | NA |
| AMP 144 | MDG51507010 | NA | bovine | F | 2 | 0 | NA | NA | NA | NA | 1.707 | -0.245 | -0.974 | -0.937 | 16.9629629629629 | NA |

| ID | PCODE | site2 | sp | gender | age | IgG | contact_ruminant | contact_milk | contact_fresh_fluid | profession | fact1 | fact2 | fact3 | fact4 | cattle_density | habitat |
| --- | --- | --- | --- | --- | --- | --- | --- | --- | --- | --- | --- | --- | --- | --- | --- | --- |
| AMP 127 | MDG51507010 | NA | bovine | M | 4 | 0 | NA | NA | NA | NA | 1.707 | -0.245 | -0.974 | -0.937 | 16.9629629629629 | NA |
| AMP 138 | MDG51507010 | NA | bovine | M | 1 | 0 | NA | NA | NA | NA | 1.707 | -0.245 | -0.974 | -0.937 | 16.9629629629629 | NA |
| AMP 137 | MDG51507010 | NA | bovine | M | 1 | 0 | NA | NA | NA | NA | 1.707 | -0.245 | -0.974 | -0.937 | 16.9629629629629 | NA |
| AMP 136 | MDG51507010 | NA | bovine | M | 5 | 0 | NA | NA | NA | NA | 1.707 | -0.245 | -0.974 | -0.937 | 16.9629629629629 | NA |
| AMP 135 | MDG51507010 | NA | bovine | F | 2 | 0 | NA | NA | NA | NA | 1.707 | -0.245 | -0.974 | -0.937 | 16.9629629629629 | NA |
| AMP 134 | MDG51507010 | NA | bovine | F | 4 | 0 | NA | NA | NA | NA | 1.707 | -0.245 | -0.974 | -0.937 | 16.9629629629629 | NA |
| AMP 133 | MDG51507010 | NA | bovine | M | 5 | 0 | NA | NA | NA | NA | 1.707 | -0.245 | -0.974 | -0.937 | 16.9629629629629 | NA |
| AMP 132 | MDG51507010 | NA | bovine | F | 1 | 0 | NA | NA | NA | NA | 1.707 | -0.245 | -0.974 | -0.937 | 16.9629629629629 | NA |
| AMP 131 | MDG51507010 | NA | bovine | F | 3 | 0 | NA | NA | NA | NA | 1.707 | -0.245 | -0.974 | -0.937 | 16.9629629629629 | NA |
| AMP 130 | MDG51507010 | NA | bovine | F | 5 | 0 | NA | NA | NA | NA | 1.707 | -0.245 | -0.974 | -0.937 | 16.9629629629629 | NA |
| AMP 120 | MDG51507010 | NA | bovine | F | 12 | 0 | NA | NA | NA | NA | 1.707 | -0.245 | -0.974 | -0.937 | 16.9629629629629 | NA |
| AMP 128 | MDG51507010 | NA | bovine | F | 5 | 0 | NA | NA | NA | NA | 1.707 | -0.245 | -0.974 | -0.937 | 16.9629629629629 | NA |
| AMP 117 | MDG51507010 | NA | bovine | F | 3 | 0 | NA | NA | NA | NA | 1.707 | -0.245 | -0.974 | -0.937 | 16.9629629629629 | NA |
| AMP 126 | MDG51507010 | NA | bovine | F | 1 | 0 | NA | NA | NA | NA | 1.707 | -0.245 | -0.974 | -0.937 | 16.9629629629629 | NA |
| AMP 125 | MDG51507010 | NA | bovine | M | 1 | 0 | NA | NA | NA | NA | 1.707 | -0.245 | -0.974 | -0.937 | 16.9629629629629 | NA |
| AMP 124 | MDG51507010 | NA | bovine | M | 6 | 0 | NA | NA | NA | NA | 1.707 | -0.245 | -0.974 | -0.937 | 16.9629629629629 | NA |
| AMP 123 | MDG51507010 | NA | bovine | F | 6 | 0 | NA | NA | NA | NA | 1.707 | -0.245 | -0.974 | -0.937 | 16.9629629629629 | NA |
| AMP 042 | MDG51507010 | NA | bovine | F | 5 | 0 | NA | NA | NA | NA | 1.707 | -0.245 | -0.974 | -0.937 | 16.9629629629629 | NA |
| AMP 121 | MDG51507010 | NA | bovine | F | 8 | 0 | NA | NA | NA | NA | 1.707 | -0.245 | -0.974 | -0.937 | 16.9629629629629 | NA |
| AMP 036 | MDG51507010 | NA | bovine | M | 3 | 0 | NA | NA | NA | NA | 1.707 | -0.245 | -0.974 | -0.937 | 16.9629629629629 | NA |
| AMP 119 | MDG51507010 | NA | bovine | F | 4 | 0 | NA | NA | NA | NA | 1.707 | -0.245 | -0.974 | -0.937 | 16.9629629629629 | NA |
| AMP 118 | MDG51507010 | NA | bovine | F | 3 | 0 | NA | NA | NA | NA | 1.707 | -0.245 | -0.974 | -0.937 | 16.9629629629629 | NA |
| AMP 129 | MDG51507010 | NA | bovine | M | 6 | 0 | NA | NA | NA | NA | 1.707 | -0.245 | -0.974 | -0.937 | 16.9629629629629 | NA |
| AMP 182 | MDG51507010 | NA | bovine | M | 4 | 0 | NA | NA | NA | NA | 1.707 | -0.245 | -0.974 | -0.937 | 16.9629629629629 | NA |
| AMP 023 | MDG51507010 | NA | bovine | M | 2 | 0 | NA | NA | NA | NA | 1.707 | -0.245 | -0.974 | -0.937 | 16.9629629629629 | NA |
| AMP 140 | MDG51507010 | NA | bovine | F | 5 | 0 | NA | NA | NA | NA | 1.707 | -0.245 | -0.974 | -0.937 | 16.9629629629629 | NA |
| AMP 163 | MDG51507010 | NA | bovine | F | 5 | 0 | NA | NA | NA | NA | 1.707 | -0.245 | -0.974 | -0.937 | 16.9629629629629 | NA |
| AMP 164 | MDG51507010 | NA | bovine | M | 2 | 0 | NA | NA | NA | NA | 1.707 | -0.245 | -0.974 | -0.937 | 16.9629629629629 | NA |
| AMP 165 | MDG51507010 | NA | bovine | M | 3 | 0 | NA | NA | NA | NA | 1.707 | -0.245 | -0.974 | -0.937 | 16.9629629629629 | NA |
| AMP 166 | MDG51507010 | NA | bovine | F | 6 | 0 | NA | NA | NA | NA | 1.707 | -0.245 | -0.974 | -0.937 | 16.9629629629629 | NA |
| AMP 167 | MDG51507010 | NA | bovine | F | 4 | 0 | NA | NA | NA | NA | 1.707 | -0.245 | -0.974 | -0.937 | 16.9629629629629 | NA |
| AMP 168 | MDG51507010 | NA | bovine | F | 2 | 0 | NA | NA | NA | NA | 1.707 | -0.245 | -0.974 | -0.937 | 16.9629629629629 | NA |
| AMP 040 | MDG51507010 | NA | bovine | F | 2 | 0 | NA | NA | NA | NA | 1.707 | -0.245 | -0.974 | -0.937 | 16.9629629629629 | NA |
| AMP 170 | MDG51507010 | NA | bovine | M | 2 | 0 | NA | NA | NA | NA | 1.707 | -0.245 | -0.974 | -0.937 | 16.9629629629629 | NA |
| AMP 024 | MDG51507010 | NA | bovine | M | 3 | 0 | NA | NA | NA | NA | 1.707 | -0.245 | -0.974 | -0.937 | 16.9629629629629 | NA |
| AMP 111 | MDG51507010 | NA | bovine | F | 7 | 0 | NA | NA | NA | NA | 1.707 | -0.245 | -0.974 | -0.937 | 16.9629629629629 | NA |
| AMP 171 | MDG51507010 | NA | bovine | M | 1 | 0 | NA | NA | NA | NA | 1.707 | -0.245 | -0.974 | -0.937 | 16.9629629629629 | NA |
| AMP 172 | MDG51507010 | NA | bovine | F | 1 | 0 | NA | NA | NA | NA | 1.707 | -0.245 | -0.974 | -0.937 | 16.9629629629629 | NA |
| AMP 198 | MDG51507010 | NA | bovine | M | 2 | 0 | NA | NA | NA | NA | 1.707 | -0.245 | -0.974 | -0.937 | 16.9629629629629 | NA |
| AMP 107 | MDG51507010 | NA | bovine | M | 2 | 0 | NA | NA | NA | NA | 1.707 | -0.245 | -0.974 | -0.937 | 16.9629629629629 | NA |
| AMP 108 | MDG51507010 | NA | bovine | M | 2 | 0 | NA | NA | NA | NA | 1.707 | -0.245 | -0.974 | -0.937 | 16.9629629629629 | NA |
| AMP 109 | MDG51507010 | NA | bovine | F | 1 | 0 | NA | NA | NA | NA | 1.707 | -0.245 | -0.974 | -0.937 | 16.9629629629629 | NA |
| AMP 110 | MDG51507010 | NA | bovine | M | 4 | 0 | NA | NA | NA | NA | 1.707 | -0.245 | -0.974 | -0.937 | 16.9629629629629 | NA |
| AMP 032 | MDG51507010 | NA | bovine | F | 2 | 0 | NA | NA | NA | NA | 1.707 | -0.245 | -0.974 | -0.937 | 16.9629629629629 | NA |
| AMP 169 | MDG51507010 | NA | bovine | F | 8 | 0 | NA | NA | NA | NA | 1.707 | -0.245 | -0.974 | -0.937 | 16.9629629629629 | NA |
| AMP 049 | MDG51507010 | NA | bovine | F | 3 | 0 | NA | NA | NA | NA | 1.707 | -0.245 | -0.974 | -0.937 | 16.9629629629629 | NA |
| AMP 037 | MDG51507010 | NA | bovine | F | 4 | 0 | NA | NA | NA | NA | 1.707 | -0.245 | -0.974 | -0.937 | 16.9629629629629 | NA |
| AMP 038 | MDG51507010 | NA | bovine | F | 3 | 0 | NA | NA | NA | NA | 1.707 | -0.245 | -0.974 | -0.937 | 16.9629629629629 | NA |
| AMP 039 | MDG51507010 | NA | bovine | F | 5 | 0 | NA | NA | NA | NA | 1.707 | -0.245 | -0.974 | -0.937 | 16.9629629629629 | NA |
| AMP 041 | MDG51507010 | NA | bovine | M | 3 | 0 | NA | NA | NA | NA | 1.707 | -0.245 | -0.974 | -0.937 | 16.9629629629629 | NA |
| AMP 043 | MDG51507010 | NA | bovine | F | 4 | 0 | NA | NA | NA | NA | 1.707 | -0.245 | -0.974 | -0.937 | 16.9629629629629 | NA |
| AMP 044 | MDG51507010 | NA | bovine | F | 5 | 0 | NA | NA | NA | NA | 1.707 | -0.245 | -0.974 | -0.937 | 16.9629629629629 | NA |
| AMP 045 | MDG51507010 | NA | bovine | F | 2 | 0 | NA | NA | NA | NA | 1.707 | -0.245 | -0.974 | -0.937 | 16.9629629629629 | NA |
| AMP 046 | MDG51507010 | NA | bovine | F | 1 | 0 | NA | NA | NA | NA | 1.707 | -0.245 | -0.974 | -0.937 | 16.9629629629629 | NA |
| AMP 151 | MDG51507010 | NA | bovine | F | 5 | 0 | NA | NA | NA | NA | 1.707 | -0.245 | -0.974 | -0.937 | 16.9629629629629 | NA |
| AMP 048 | MDG51507010 | NA | bovine | F | 2 | 0 | NA | NA | NA | NA | 1.707 | -0.245 | -0.974 | -0.937 | 16.9629629629629 | NA |
| AMP 150 | MDG51507010 | NA | bovine | M | 1 | 0 | NA | NA | NA | NA | 1.707 | -0.245 | -0.974 | -0.937 | 16.9629629629629 | NA |
| AMP 029 | MDG51507010 | NA | bovine | F | 2 | 0 | NA | NA | NA | NA | 1.707 | -0.245 | -0.974 | -0.937 | 16.9629629629629 | NA |
| AMP 051 | MDG51507010 | NA | bovine | M | 1 | 0 | NA | NA | NA | NA | 1.707 | -0.245 | -0.974 | -0.937 | 16.9629629629629 | NA |
| AMP 148 | MDG51507010 | NA | bovine | M | 5 | 0 | NA | NA | NA | NA | 1.707 | -0.245 | -0.974 | -0.937 | 16.9629629629629 | NA |
| AMP 028 | MDG51507010 | NA | bovine | F | 2 | 0 | NA | NA | NA | NA | 1.707 | -0.245 | -0.974 | -0.937 | 16.9629629629629 | NA |
| AMP 027 | MDG51507010 | NA | bovine | F | 3 | 0 | NA | NA | NA | NA | 1.707 | -0.245 | -0.974 | -0.937 | 16.9629629629629 | NA |
| AMP 026 | MDG51507010 | NA | bovine | F | 2 | 0 | NA | NA | NA | NA | 1.707 | -0.245 | -0.974 | -0.937 | 16.9629629629629 | NA |

| ID | PCODE | site2 | sp | gender | age | IgG | contact_ruminant | contact_milk | contact_fresh_fluid | profession | fact1 | fact2 | fact3 | fact4 | cattle_density | habitat |
| --- | --- | --- | --- | --- | --- | --- | --- | --- | --- | --- | --- | --- | --- | --- | --- | --- |
| AMP 149 | MDG51507010 | NA | bovine | M | 3 | 0 | NA | NA | NA | NA | 1.707 | -0.245 | -0.974 | -0.937 | 16.9629629629629 | NA |
| AMP 025 | MDG51507010 | NA | bovine | F | 2 | 0 | NA | NA | NA | NA | 1.707 | -0.245 | -0.974 | -0.937 | 16.9629629629629 | NA |
| AMP 030 | MDG51507010 | NA | bovine | F | 2 | 0 | NA | NA | NA | NA | 1.707 | -0.245 | -0.974 | -0.937 | 16.9629629629629 | NA |
| AMP 047 | MDG51507010 | NA | bovine | M | 4 | 0 | NA | NA | NA | NA | 1.707 | -0.245 | -0.974 | -0.937 | 16.9629629629629 | NA |
| AMP 188 | MDG51507030 | NA | bovine | F | 8 | 0 | NA | NA | NA | NA | 2.077 | -0.444 | -0.611 | -0.691 | 19.0833333333333 | NA |
| AMP 190 | MDG51507030 | NA | bovine | M | 1 | 0 | NA | NA | NA | NA | 2.077 | -0.444 | -0.611 | -0.691 | 19.0833333333333 | NA |
| AMP 186 | MDG51507030 | NA | bovine | F | 4 | 0 | NA | NA | NA | NA | 2.077 | -0.444 | -0.611 | -0.691 | 19.0833333333333 | NA |
| AMP 181 | MDG51507030 | NA | bovine | F | 2 | 0 | NA | NA | NA | NA | 2.077 | -0.444 | -0.611 | -0.691 | 19.0833333333333 | NA |
| AMP 183 | MDG51507030 | NA | bovine | F | 5 | 0 | NA | NA | NA | NA | 2.077 | -0.444 | -0.611 | -0.691 | 19.0833333333333 | NA |
| AMP 184 | MDG51507030 | NA | bovine | F | 4 | 0 | NA | NA | NA | NA | 2.077 | -0.444 | -0.611 | -0.691 | 19.0833333333333 | NA |
| AMP 185 | MDG51507030 | NA | bovine | F | 1 | 0 | NA | NA | NA | NA | 2.077 | -0.444 | -0.611 | -0.691 | 19.0833333333333 | NA |
| AMP 187 | MDG51507030 | NA | bovine | F | 3 | 0 | NA | NA | NA | NA | 2.077 | -0.444 | -0.611 | -0.691 | 19.0833333333333 | NA |
| AMP 179 | MDG51507030 | NA | bovine | M | 1 | 0 | NA | NA | NA | NA | 2.077 | -0.444 | -0.611 | -0.691 | 19.0833333333333 | NA |
| AMP 189 | MDG51507030 | NA | bovine | M | 3 | 0 | NA | NA | NA | NA | 2.077 | -0.444 | -0.611 | -0.691 | 19.0833333333333 | NA |
| AMP 178 | MDG51507030 | NA | bovine | F | 3 | 0 | NA | NA | NA | NA | 2.077 | -0.444 | -0.611 | -0.691 | 19.0833333333333 | NA |
| AMP 191 | MDG51507030 | NA | bovine | F | 6 | 0 | NA | NA | NA | NA | 2.077 | -0.444 | -0.611 | -0.691 | 19.0833333333333 | NA |
| AMP 192 | MDG51507030 | NA | bovine | M | 5 | 0 | NA | NA | NA | NA | 2.077 | -0.444 | -0.611 | -0.691 | 19.0833333333333 | NA |
| AMP 193 | MDG51507030 | NA | bovine | F | 5 | 0 | NA | NA | NA | NA | 2.077 | -0.444 | -0.611 | -0.691 | 19.0833333333333 | NA |
| AMP 194 | MDG51507030 | NA | bovine | M | 4 | 0 | NA | NA | NA | NA | 2.077 | -0.444 | -0.611 | -0.691 | 19.0833333333333 | NA |
| AMP 196 | MDG51507030 | NA | bovine | F | 1 | 0 | NA | NA | NA | NA | 2.077 | -0.444 | -0.611 | -0.691 | 19.0833333333333 | NA |
| AMP 197 | MDG51507030 | NA | bovine | M | 8 | 0 | NA | NA | NA | NA | 2.077 | -0.444 | -0.611 | -0.691 | 19.0833333333333 | NA |
| AMP 065 | MDG51507030 | NA | bovine | F | 5 | 0 | NA | NA | NA | NA | 2.077 | -0.444 | -0.611 | -0.691 | 19.0833333333333 | NA |
| AMP 161 | MDG51507030 | NA | bovine | F | 3 | 0 | NA | NA | NA | NA | 2.077 | -0.444 | -0.611 | -0.691 | 19.0833333333333 | NA |
| AMP 153 | MDG51507030 | NA | bovine | F | 2 | 0 | NA | NA | NA | NA | 2.077 | -0.444 | -0.611 | -0.691 | 19.0833333333333 | NA |
| AMP 154 | MDG51507030 | NA | bovine | M | 2 | 0 | NA | NA | NA | NA | 2.077 | -0.444 | -0.611 | -0.691 | 19.0833333333333 | NA |
| AMP 155 | MDG51507030 | NA | bovine | F | 3 | 0 | NA | NA | NA | NA | 2.077 | -0.444 | -0.611 | -0.691 | 19.0833333333333 | NA |
| AMP 156 | MDG51507030 | NA | bovine | F | 1 | 0 | NA | NA | NA | NA | 2.077 | -0.444 | -0.611 | -0.691 | 19.0833333333333 | NA |
| AMP 157 | MDG51507030 | NA | bovine | F | 8 | 0 | NA | NA | NA | NA | 2.077 | -0.444 | -0.611 | -0.691 | 19.0833333333333 | NA |
| AMP 158 | MDG51507030 | NA | bovine | M | 3 | 0 | NA | NA | NA | NA | 2.077 | -0.444 | -0.611 | -0.691 | 19.0833333333333 | NA |
| AMP 180 | MDG51507030 | NA | bovine | M | 2 | 0 | NA | NA | NA | NA | 2.077 | -0.444 | -0.611 | -0.691 | 19.0833333333333 | NA |
| AMP 160 | MDG51507030 | NA | bovine | F | 3 | 0 | NA | NA | NA | NA | 2.077 | -0.444 | -0.611 | -0.691 | 19.0833333333333 | NA |
| AMP 152 | MDG51507030 | NA | bovine | F | 8 | 0 | NA | NA | NA | NA | 2.077 | -0.444 | -0.611 | -0.691 | 19.0833333333333 | NA |
| AMP 162 | MDG51507030 | NA | bovine | F | 2 | 0 | NA | NA | NA | NA | 2.077 | -0.444 | -0.611 | -0.691 | 19.0833333333333 | NA |
| AMP 173 | MDG51507030 | NA | bovine | M | 1 | 0 | NA | NA | NA | NA | 2.077 | -0.444 | -0.611 | -0.691 | 19.0833333333333 | NA |
| AMP 174 | MDG51507030 | NA | bovine | F | 8 | 0 | NA | NA | NA | NA | 2.077 | -0.444 | -0.611 | -0.691 | 19.0833333333333 | NA |
| AMP 175 | MDG51507030 | NA | bovine | F | 4 | 0 | NA | NA | NA | NA | 2.077 | -0.444 | -0.611 | -0.691 | 19.0833333333333 | NA |
| AMP 176 | MDG51507030 | NA | bovine | F | 1 | 0 | NA | NA | NA | NA | 2.077 | -0.444 | -0.611 | -0.691 | 19.0833333333333 | NA |
| AMP 177 | MDG51507030 | NA | bovine | F | 1 | 0 | NA | NA | NA | NA | 2.077 | -0.444 | -0.611 | -0.691 | 19.0833333333333 | NA |
| AMP 159 | MDG51507030 | NA | bovine | F | 8 | 0 | NA | NA | NA | NA | 2.077 | -0.444 | -0.611 | -0.691 | 19.0833333333333 | NA |
| AMP 055 | MDG51507030 | NA | bovine | F | 3 | 0 | NA | NA | NA | NA | 2.077 | -0.444 | -0.611 | -0.691 | 19.0833333333333 | NA |
| AMP 003 | MDG51507030 | NA | bovine | F | 7 | 0 | NA | NA | NA | NA | 2.077 | -0.444 | -0.611 | -0.691 | 19.0833333333333 | NA |
| AMP 002 | MDG51507030 | NA | bovine | F | 2 | 0 | NA | NA | NA | NA | 2.077 | -0.444 | -0.611 | -0.691 | 19.0833333333333 | NA |
| AMP 001 | MDG51507030 | NA | bovine | F | 2 | 0 | NA | NA | NA | NA | 2.077 | -0.444 | -0.611 | -0.691 | 19.0833333333333 | NA |
| AMP 016 | MDG51507030 | NA | bovine | F | 10 | 0 | NA | NA | NA | NA | 2.077 | -0.444 | -0.611 | -0.691 | 19.0833333333333 | NA |
| AMP 050 | MDG51507030 | NA | bovine | F | 8 | 0 | NA | NA | NA | NA | 2.077 | -0.444 | -0.611 | -0.691 | 19.0833333333333 | NA |
| AMP 052 | MDG51507030 | NA | bovine | F | 4 | 0 | NA | NA | NA | NA | 2.077 | -0.444 | -0.611 | -0.691 | 19.0833333333333 | NA |
| AMP 064 | MDG51507030 | NA | bovine | M | 4 | 0 | NA | NA | NA | NA | 2.077 | -0.444 | -0.611 | -0.691 | 19.0833333333333 | NA |
| AMP 054 | MDG51507030 | NA | bovine | F | 4 | 0 | NA | NA | NA | NA | 2.077 | -0.444 | -0.611 | -0.691 | 19.0833333333333 | NA |
| AMP 022 | MDG51507030 | NA | bovine | F | 2 | 0 | NA | NA | NA | NA | 2.077 | -0.444 | -0.611 | -0.691 | 19.0833333333333 | NA |
| AMP 056 | MDG51507030 | NA | bovine | M | 3 | 0 | NA | NA | NA | NA | 2.077 | -0.444 | -0.611 | -0.691 | 19.0833333333333 | NA |
| AMP 013 | MDG51507030 | NA | bovine | F | 2 | 0 | NA | NA | NA | NA | 2.077 | -0.444 | -0.611 | -0.691 | 19.0833333333333 | NA |
| AMP 058 | MDG51507030 | NA | bovine | F | 6 | 0 | NA | NA | NA | NA | 2.077 | -0.444 | -0.611 | -0.691 | 19.0833333333333 | NA |
| AMP 060 | MDG51507030 | NA | bovine | F | 1 | 0 | NA | NA | NA | NA | 2.077 | -0.444 | -0.611 | -0.691 | 19.0833333333333 | NA |
| AMP 061 | MDG51507030 | NA | bovine | F | 7 | 0 | NA | NA | NA | NA | 2.077 | -0.444 | -0.611 | -0.691 | 19.0833333333333 | NA |
| AMP 062 | MDG51507030 | NA | bovine | M | 2 | 0 | NA | NA | NA | NA | 2.077 | -0.444 | -0.611 | -0.691 | 19.0833333333333 | NA |
| AMP 063 | MDG51507030 | NA | bovine | F | 6 | 0 | NA | NA | NA | NA | 2.077 | -0.444 | -0.611 | -0.691 | 19.0833333333333 | NA |
| AMP 053 | MDG51507030 | NA | bovine | F | 1 | 0 | NA | NA | NA | NA | 2.077 | -0.444 | -0.611 | -0.691 | 19.0833333333333 | NA |
| AMP 018 | MDG51507030 | NA | bovine | F | 2 | 0 | NA | NA | NA | NA | 2.077 | -0.444 | -0.611 | -0.691 | 19.0833333333333 | NA |
| AMP 014 | MDG51507030 | NA | bovine | F | 6 | 0 | NA | NA | NA | NA | 2.077 | -0.444 | -0.611 | -0.691 | 19.0833333333333 | NA |
| AMP 012 | MDG51507030 | NA | bovine | M | 1 | 0 | NA | NA | NA | NA | 2.077 | -0.444 | -0.611 | -0.691 | 19.0833333333333 | NA |
| AMP 015 | MDG51507030 | NA | bovine | F | 5 | 0 | NA | NA | NA | NA | 2.077 | -0.444 | -0.611 | -0.691 | 19.0833333333333 | NA |
| AMP 011 | MDG51507030 | NA | bovine | F | 2 | 0 | NA | NA | NA | NA | 2.077 | -0.444 | -0.611 | -0.691 | 19.0833333333333 | NA |
| AMP 010 | MDG51507030 | NA | bovine | F | 8 | 0 | NA | NA | NA | NA | 2.077 | -0.444 | -0.611 | -0.691 | 19.0833333333333 | NA |

| ID | PCODE | site2 | sp | gender | age | IgG | contact_ruminant | contact_milk | contact_fresh_fluid | profession | fact1 | fact2 | fact3 | fact4 | cattle_density | habitat |
| --- | --- | --- | --- | --- | --- | --- | --- | --- | --- | --- | --- | --- | --- | --- | --- | --- |
| AMP 009 | MDG51507030 | NA | bovine | M | 2 | 0 | NA | NA | NA | NA | 2.077 | -0.444 | -0.611 | -0.691 | 19.0833333333333 | NA |
| AMP 008 | MDG51507030 | NA | bovine | F | 6 | 0 | NA | NA | NA | NA | 2.077 | -0.444 | -0.611 | -0.691 | 19.0833333333333 | NA |
| AMP 004 | MDG51507030 | NA | bovine | M | 1 | 0 | NA | NA | NA | NA | 2.077 | -0.444 | -0.611 | -0.691 | 19.0833333333333 | NA |
| AMP 017 | MDG51507030 | NA | bovine | F | 2 | 0 | NA | NA | NA | NA | 2.077 | -0.444 | -0.611 | -0.691 | 19.0833333333333 | NA |
| AMP 005 | MDG51507030 | NA | bovine | F | 5 | 0 | NA | NA | NA | NA | 2.077 | -0.444 | -0.611 | -0.691 | 19.0833333333333 | NA |
| AMP 019 | MDG51507030 | NA | bovine | F | 1 | 0 | NA | NA | NA | NA | 2.077 | -0.444 | -0.611 | -0.691 | 19.0833333333333 | NA |
| AMP 020 | MDG51507030 | NA | bovine | F | 1 | 0 | NA | NA | NA | NA | 2.077 | -0.444 | -0.611 | -0.691 | 19.0833333333333 | NA |
| AMP 106 | MDG51507030 | NA | bovine | M | 1 | 0 | NA | NA | NA | NA | 2.077 | -0.444 | -0.611 | -0.691 | 19.0833333333333 | NA |
| AMP 067 | MDG51507030 | NA | bovine | F | 8 | 0 | NA | NA | NA | NA | 2.077 | -0.444 | -0.611 | -0.691 | 19.0833333333333 | NA |
| AMP 021 | MDG51507030 | NA | bovine | F | 2 | 0 | NA | NA | NA | NA | 2.077 | -0.444 | -0.611 | -0.691 | 19.0833333333333 | NA |
| AMP 006 | MDG51507030 | NA | bovine | M | 2 | 0 | NA | NA | NA | NA | 2.077 | -0.444 | -0.611 | -0.691 | 19.0833333333333 | NA |
| AMP 059 | MDG51507030 | NA | bovine | F | 5 | 0 | NA | NA | NA | NA | 2.077 | -0.444 | -0.611 | -0.691 | 19.0833333333333 | NA |
| AMP 007 | MDG51507030 | NA | bovine | F | 7 | 0 | NA | NA | NA | NA | 2.077 | -0.444 | -0.611 | -0.691 | 19.0833333333333 | NA |
| AMP 096 | MDG51507030 | NA | bovine | F | 1 | 0 | NA | NA | NA | NA | 2.077 | -0.444 | -0.611 | -0.691 | 19.0833333333333 | NA |
| AMP 085 | MDG51507030 | NA | bovine | M | 2 | 0 | NA | NA | NA | NA | 2.077 | -0.444 | -0.611 | -0.691 | 19.0833333333333 | NA |
| AMP 057 | MDG51507030 | NA | bovine | F | 7 | 0 | NA | NA | NA | NA | 2.077 | -0.444 | -0.611 | -0.691 | 19.0833333333333 | NA |
| AMP 087 | MDG51507030 | NA | bovine | F | 5 | 0 | NA | NA | NA | NA | 2.077 | -0.444 | -0.611 | -0.691 | 19.0833333333333 | NA |
| AMP 088 | MDG51507030 | NA | bovine | M | 1 | 0 | NA | NA | NA | NA | 2.077 | -0.444 | -0.611 | -0.691 | 19.0833333333333 | NA |
| AMP 089 | MDG51507030 | NA | bovine | M | 1 | 0 | NA | NA | NA | NA | 2.077 | -0.444 | -0.611 | -0.691 | 19.0833333333333 | NA |
| AMP 090 | MDG51507030 | NA | bovine | F | 4 | 0 | NA | NA | NA | NA | 2.077 | -0.444 | -0.611 | -0.691 | 19.0833333333333 | NA |
| AMP 091 | MDG51507030 | NA | bovine | F | 3 | 0 | NA | NA | NA | NA | 2.077 | -0.444 | -0.611 | -0.691 | 19.0833333333333 | NA |
| AMP 092 | MDG51507030 | NA | bovine | F | 8 | 0 | NA | NA | NA | NA | 2.077 | -0.444 | -0.611 | -0.691 | 19.0833333333333 | NA |
| AMP 093 | MDG51507030 | NA | bovine | M | 2 | 0 | NA | NA | NA | NA | 2.077 | -0.444 | -0.611 | -0.691 | 19.0833333333333 | NA |
| AMP 066 | MDG51507030 | NA | bovine | F | 3 | 0 | NA | NA | NA | NA | 2.077 | -0.444 | -0.611 | -0.691 | 19.0833333333333 | NA |
| AMP 095 | MDG51507030 | NA | bovine | M | 2 | 0 | NA | NA | NA | NA | 2.077 | -0.444 | -0.611 | -0.691 | 19.0833333333333 | NA |
| AMP 084 | MDG51507030 | NA | bovine | M | 3 | 0 | NA | NA | NA | NA | 2.077 | -0.444 | -0.611 | -0.691 | 19.0833333333333 | NA |
| AMP 097 | MDG51507030 | NA | bovine | F | 2 | 0 | NA | NA | NA | NA | 2.077 | -0.444 | -0.611 | -0.691 | 19.0833333333333 | NA |
| AMP 098 | MDG51507030 | NA | bovine | F | 3 | 0 | NA | NA | NA | NA | 2.077 | -0.444 | -0.611 | -0.691 | 19.0833333333333 | NA |
| AMP 099 | MDG51507030 | NA | bovine | M | 2 | 0 | NA | NA | NA | NA | 2.077 | -0.444 | -0.611 | -0.691 | 19.0833333333333 | NA |
| AMP 100 | MDG51507030 | NA | bovine | F | 1 | 0 | NA | NA | NA | NA | 2.077 | -0.444 | -0.611 | -0.691 | 19.0833333333333 | NA |
| AMP 101 | MDG51507030 | NA | bovine | F | 4 | 0 | NA | NA | NA | NA | 2.077 | -0.444 | -0.611 | -0.691 | 19.0833333333333 | NA |
| AMP 102 | MDG51507030 | NA | bovine | F | 2 | 0 | NA | NA | NA | NA | 2.077 | -0.444 | -0.611 | -0.691 | 19.0833333333333 | NA |
| AMP 103 | MDG51507030 | NA | bovine | F | 3 | 0 | NA | NA | NA | NA | 2.077 | -0.444 | -0.611 | -0.691 | 19.0833333333333 | NA |
| AMP 104 | MDG51507030 | NA | bovine | F | 1 | 0 | NA | NA | NA | NA | 2.077 | -0.444 | -0.611 | -0.691 | 19.0833333333333 | NA |
| AMP 105 | MDG51507030 | NA | bovine | F | 2 | 0 | NA | NA | NA | NA | 2.077 | -0.444 | -0.611 | -0.691 | 19.0833333333333 | NA |
| AMP 094 | MDG51507030 | NA | bovine | F | 6 | 0 | NA | NA | NA | NA | 2.077 | -0.444 | -0.611 | -0.691 | 19.0833333333333 | NA |
| AMP 081 | MDG51507030 | NA | bovine | F | 9 | 0 | NA | NA | NA | NA | 2.077 | -0.444 | -0.611 | -0.691 | 19.0833333333333 | NA |
| AMP 068 | MDG51507030 | NA | bovine | M | 6 | 0 | NA | NA | NA | NA | 2.077 | -0.444 | -0.611 | -0.691 | 19.0833333333333 | NA |
| AMP 069 | MDG51507030 | NA | bovine | F | 2 | 0 | NA | NA | NA | NA | 2.077 | -0.444 | -0.611 | -0.691 | 19.0833333333333 | NA |
| AMP 070 | MDG51507030 | NA | bovine | F | 2 | 0 | NA | NA | NA | NA | 2.077 | -0.444 | -0.611 | -0.691 | 19.0833333333333 | NA |
| AMP 071 | MDG51507030 | NA | bovine | M | 2 | 0 | NA | NA | NA | NA | 2.077 | -0.444 | -0.611 | -0.691 | 19.0833333333333 | NA |
| AMP 072 | MDG51507030 | NA | bovine | F | 1 | 0 | NA | NA | NA | NA | 2.077 | -0.444 | -0.611 | -0.691 | 19.0833333333333 | NA |
| AMP 075 | MDG51507030 | NA | bovine | M | 4 | 0 | NA | NA | NA | NA | 2.077 | -0.444 | -0.611 | -0.691 | 19.0833333333333 | NA |
| AMP 076 | MDG51507030 | NA | bovine | M | 2 | 0 | NA | NA | NA | NA | 2.077 | -0.444 | -0.611 | -0.691 | 19.0833333333333 | NA |
| AMP 077 | MDG51507030 | NA | bovine | M | 1 | 0 | NA | NA | NA | NA | 2.077 | -0.444 | -0.611 | -0.691 | 19.0833333333333 | NA |
| AMP 078 | MDG51507030 | NA | bovine | F | 1 | 0 | NA | NA | NA | NA | 2.077 | -0.444 | -0.611 | -0.691 | 19.0833333333333 | NA |
| AMP 086 | MDG51507030 | NA | bovine | F | 4 | 0 | NA | NA | NA | NA | 2.077 | -0.444 | -0.611 | -0.691 | 19.0833333333333 | NA |
| AMP 080 | MDG51507030 | NA | bovine | F | 2 | 0 | NA | NA | NA | NA | 2.077 | -0.444 | -0.611 | -0.691 | 19.0833333333333 | NA |
| AMP 082 | MDG51507030 | NA | bovine | F | 1 | 0 | NA | NA | NA | NA | 2.077 | -0.444 | -0.611 | -0.691 | 19.0833333333333 | NA |
| AMP 083 | MDG51507030 | NA | bovine | M | 1 | 0 | NA | NA | NA | NA | 2.077 | -0.444 | -0.611 | -0.691 | 19.0833333333333 | NA |
| AMP 079 | MDG51507030 | NA | bovine | F | 7 | 0 | NA | NA | NA | NA | 2.077 | -0.444 | -0.611 | -0.691 | 19.0833333333333 | NA |
| SAK 342 | MDG51512011 | NA | bovine | F | 6 | 0 | NA | NA | NA | NA | 1.996 | -0.642 | -0.283 | -0.395 | 14.1818181818181 | NA |
| SAK 339 | MDG51512011 | NA | bovine | M | 5 | 0 | NA | NA | NA | NA | 1.996 | -0.642 | -0.283 | -0.395 | 14.1818181818181 | NA |
| SAK 340 | MDG51512011 | NA | bovine | F | 7 | 0 | NA | NA | NA | NA | 1.996 | -0.642 | -0.283 | -0.395 | 14.1818181818181 | NA |
| SAK 305 | MDG51512011 | NA | bovine | F | 5 | 0 | NA | NA | NA | NA | 1.996 | -0.642 | -0.283 | -0.395 | 14.1818181818181 | NA |
| SAK 341 | MDG51512011 | NA | bovine | F | 4 | 0 | NA | NA | NA | NA | 1.996 | -0.642 | -0.283 | -0.395 | 14.1818181818181 | NA |
| SAK 316 | MDG51512011 | NA | bovine | F | 4 | 0 | NA | NA | NA | NA | 1.996 | -0.642 | -0.283 | -0.395 | 14.1818181818181 | NA |
| SAK 336 | MDG51512011 | NA | bovine | M | 4 | 0 | NA | NA | NA | NA | 1.996 | -0.642 | -0.283 | -0.395 | 14.1818181818181 | NA |
| SAK 338 | MDG51512011 | NA | bovine | M | 4 | 0 | NA | NA | NA | NA | 1.996 | -0.642 | -0.283 | -0.395 | 14.1818181818181 | NA |
| SAK 337 | MDG51512011 | NA | bovine | M | 3 | 0 | NA | NA | NA | NA | 1.996 | -0.642 | -0.283 | -0.395 | 14.1818181818181 | NA |
| SAK 303 | MDG51512011 | NA | bovine | F | 4 | 0 | NA | NA | NA | NA | 1.996 | -0.642 | -0.283 | -0.395 | 14.1818181818181 | NA |
| SAK 302 | MDG51512011 | NA | bovine | M | 3 | 0 | NA | NA | NA | NA | 1.996 | -0.642 | -0.283 | -0.395 | 14.1818181818181 | NA |
| SAK 343 | MDG51512011 | NA | bovine | M | 4 | 0 | NA | NA | NA | NA | 1.996 | -0.642 | -0.283 | -0.395 | 14.1818181818181 | NA |

| ID | PCODE | site2 | sp | gender | age | IgG | contact_ruminant | contact_milk | contact_fresh_fluid | profession | fact1 | fact2 | fact3 | fact4 | cattle_density | habitat |
| --- | --- | --- | --- | --- | --- | --- | --- | --- | --- | --- | --- | --- | --- | --- | --- | --- |
| SAK 306 | MDG51512011 | NA | bovine | M | 2 | 0 | NA | NA | NA | NA | 1.996 | -0.642 | -0.283 | -0.395 | 14.1818181818181 | NA |
| SAK 307 | MDG51512011 | NA | bovine | F | 7 | 0 | NA | NA | NA | NA | 1.996 | -0.642 | -0.283 | -0.395 | 14.1818181818181 | NA |
| SAK 325 | MDG51512011 | NA | bovine | M | 6 | 0 | NA | NA | NA | NA | 1.996 | -0.642 | -0.283 | -0.395 | 14.1818181818181 | NA |
| SAK 311 | MDG51512011 | NA | bovine | F | 4 | 0 | NA | NA | NA | NA | 1.996 | -0.642 | -0.283 | -0.395 | 14.1818181818181 | NA |
| SAK 312 | MDG51512011 | NA | bovine | M | 5 | 0 | NA | NA | NA | NA | 1.996 | -0.642 | -0.283 | -0.395 | 14.1818181818181 | NA |
| SAK 313 | MDG51512011 | NA | bovine | F | 2 | 0 | NA | NA | NA | NA | 1.996 | -0.642 | -0.283 | -0.395 | 14.1818181818181 | NA |
| SAK 314 | MDG51512011 | NA | bovine | F | 3 | 0 | NA | NA | NA | NA | 1.996 | -0.642 | -0.283 | -0.395 | 14.1818181818181 | NA |
| SAK 315 | MDG51512011 | NA | bovine | M | 3 | 0 | NA | NA | NA | NA | 1.996 | -0.642 | -0.283 | -0.395 | 14.1818181818181 | NA |
| SAK 317 | MDG51512011 | NA | bovine | M | 3 | 0 | NA | NA | NA | NA | 1.996 | -0.642 | -0.283 | -0.395 | 14.1818181818181 | NA |
| SAK 344 | MDG51512011 | NA | bovine | M | 2 | 0 | NA | NA | NA | NA | 1.996 | -0.642 | -0.283 | -0.395 | 14.1818181818181 | NA |
| SAK 319 | MDG51512011 | NA | bovine | M | 6 | 0 | NA | NA | NA | NA | 1.996 | -0.642 | -0.283 | -0.395 | 14.1818181818181 | NA |
| SAK 320 | MDG51512011 | NA | bovine | M | 7 | 0 | NA | NA | NA | NA | 1.996 | -0.642 | -0.283 | -0.395 | 14.1818181818181 | NA |
| SAK 321 | MDG51512011 | NA | bovine | F | 7 | 0 | NA | NA | NA | NA | 1.996 | -0.642 | -0.283 | -0.395 | 14.1818181818181 | NA |
| SAK 322 | MDG51512011 | NA | bovine | M | 6 | 0 | NA | NA | NA | NA | 1.996 | -0.642 | -0.283 | -0.395 | 14.1818181818181 | NA |
| SAK 318 | MDG51512011 | NA | bovine | M | 4 | 0 | NA | NA | NA | NA | 1.996 | -0.642 | -0.283 | -0.395 | 14.1818181818181 | NA |
| SAK 324 | MDG51512011 | NA | bovine | F | 7 | 0 | NA | NA | NA | NA | 1.996 | -0.642 | -0.283 | -0.395 | 14.1818181818181 | NA |
| SAK 335 | MDG51512011 | NA | bovine | M | 3 | 0 | NA | NA | NA | NA | 1.996 | -0.642 | -0.283 | -0.395 | 14.1818181818181 | NA |
| SAK 326 | MDG51512011 | NA | bovine | F | 4 | 0 | NA | NA | NA | NA | 1.996 | -0.642 | -0.283 | -0.395 | 14.1818181818181 | NA |
| SAK 327 | MDG51512011 | NA | bovine | F | 4 | 0 | NA | NA | NA | NA | 1.996 | -0.642 | -0.283 | -0.395 | 14.1818181818181 | NA |
| SAK 328 | MDG51512011 | NA | bovine | F | 5 | 0 | NA | NA | NA | NA | 1.996 | -0.642 | -0.283 | -0.395 | 14.1818181818181 | NA |
| SAK 329 | MDG51512011 | NA | bovine | F | 3 | 0 | NA | NA | NA | NA | 1.996 | -0.642 | -0.283 | -0.395 | 14.1818181818181 | NA |
| SAK 330 | MDG51512011 | NA | bovine | F | 3 | 0 | NA | NA | NA | NA | 1.996 | -0.642 | -0.283 | -0.395 | 14.1818181818181 | NA |
| SAK 309 | MDG51512011 | NA | bovine | F | 5 | 0 | NA | NA | NA | NA | 1.996 | -0.642 | -0.283 | -0.395 | 14.1818181818181 | NA |
| SAK 331 | MDG51512011 | NA | bovine | F | 4 | 0 | NA | NA | NA | NA | 1.996 | -0.642 | -0.283 | -0.395 | 14.1818181818181 | NA |
| SAK 332 | MDG51512011 | NA | bovine | M | 3 | 0 | NA | NA | NA | NA | 1.996 | -0.642 | -0.283 | -0.395 | 14.1818181818181 | NA |
| SAK 333 | MDG51512011 | NA | bovine | M | 4 | 0 | NA | NA | NA | NA | 1.996 | -0.642 | -0.283 | -0.395 | 14.1818181818181 | NA |
| SAK 308 | MDG51512011 | NA | bovine | F | 3 | 0 | NA | NA | NA | NA | 1.996 | -0.642 | -0.283 | -0.395 | 14.1818181818181 | NA |
| SAK 334 | MDG51512011 | NA | bovine | M | 3 | 0 | NA | NA | NA | NA | 1.996 | -0.642 | -0.283 | -0.395 | 14.1818181818181 | NA |
| SAK 323 | MDG51512011 | NA | bovine | M | 6 | 0 | NA | NA | NA | NA | 1.996 | -0.642 | -0.283 | -0.395 | 14.1818181818181 | NA |
| SAK 061 | MDG51512011 | NA | bovine | F | 6 | 0 | NA | NA | NA | NA | 1.996 | -0.642 | -0.283 | -0.395 | 14.1818181818181 | NA |
| SAK 170 | MDG51512011 | NA | bovine | F | 4 | 0 | NA | NA | NA | NA | 1.996 | -0.642 | -0.283 | -0.395 | 14.1818181818181 | NA |
| SAK 162 | MDG51512011 | NA | bovine | M | 3 | 0 | NA | NA | NA | NA | 1.996 | -0.642 | -0.283 | -0.395 | 14.1818181818181 | NA |
| SAK 169 | MDG51512011 | NA | bovine | F | 5 | 0 | NA | NA | NA | NA | 1.996 | -0.642 | -0.283 | -0.395 | 14.1818181818181 | NA |
| SAK 310 | MDG51512011 | NA | bovine | M | 4 | 0 | NA | NA | NA | NA | 1.996 | -0.642 | -0.283 | -0.395 | 14.1818181818181 | NA |
| SAK 345 | MDG51512011 | NA | bovine | M | 3 | 0 | NA | NA | NA | NA | 1.996 | -0.642 | -0.283 | -0.395 | 14.1818181818181 | NA |
| SAK 168 | MDG51512011 | NA | bovine | F | 4 | 0 | NA | NA | NA | NA | 1.996 | -0.642 | -0.283 | -0.395 | 14.1818181818181 | NA |
| SAK 060 | MDG51512011 | NA | bovine | F | 5 | 0 | NA | NA | NA | NA | 1.996 | -0.642 | -0.283 | -0.395 | 14.1818181818181 | NA |
| SAK 171 | MDG51512011 | NA | bovine | F | 4 | 0 | NA | NA | NA | NA | 1.996 | -0.642 | -0.283 | -0.395 | 14.1818181818181 | NA |
| SAK 167 | MDG51512011 | NA | bovine | F | 5 | 0 | NA | NA | NA | NA | 1.996 | -0.642 | -0.283 | -0.395 | 14.1818181818181 | NA |
| SAK 161 | MDG51512011 | NA | bovine | F | 4 | 0 | NA | NA | NA | NA | 1.996 | -0.642 | -0.283 | -0.395 | 14.1818181818181 | NA |
| SAK 166 | MDG51512011 | NA | bovine | F | 12 | 0 | NA | NA | NA | NA | 1.996 | -0.642 | -0.283 | -0.395 | 14.1818181818181 | NA |
| SAK 165 | MDG51512011 | NA | bovine | F | 8 | 0 | NA | NA | NA | NA | 1.996 | -0.642 | -0.283 | -0.395 | 14.1818181818181 | NA |
| SAK 164 | MDG51512011 | NA | bovine | M | 3 | 0 | NA | NA | NA | NA | 1.996 | -0.642 | -0.283 | -0.395 | 14.1818181818181 | NA |
| SAK 163 | MDG51512011 | NA | bovine | M | 3 | 0 | NA | NA | NA | NA | 1.996 | -0.642 | -0.283 | -0.395 | 14.1818181818181 | NA |
| SAK 048 | MDG51512011 | NA | bovine | F | 5 | 0 | NA | NA | NA | NA | 1.996 | -0.642 | -0.283 | -0.395 | 14.1818181818181 | NA |
| SAK 348 | MDG51512011 | NA | bovine | F | 4 | 0 | NA | NA | NA | NA | 1.996 | -0.642 | -0.283 | -0.395 | 14.1818181818181 | NA |
| SAK 301 | MDG51512011 | NA | bovine | F | 3 | 0 | NA | NA | NA | NA | 1.996 | -0.642 | -0.283 | -0.395 | 14.1818181818181 | NA |
| SAK 346 | MDG51512011 | NA | bovine | M | 3 | 0 | NA | NA | NA | NA | 1.996 | -0.642 | -0.283 | -0.395 | 14.1818181818181 | NA |
| SAK 300 | MDG51512011 | NA | bovine | F | 2 | 0 | NA | NA | NA | NA | 1.996 | -0.642 | -0.283 | -0.395 | 14.1818181818181 | NA |
| SAK 299 | MDG51512011 | NA | bovine | F | 2 | 0 | NA | NA | NA | NA | 1.996 | -0.642 | -0.283 | -0.395 | 14.1818181818181 | NA |
| SAK 033 | MDG51512011 | NA | bovine | M | 3 | 0 | NA | NA | NA | NA | 1.996 | -0.642 | -0.283 | -0.395 | 14.1818181818181 | NA |
| SAK 037 | MDG51512011 | NA | bovine | F | 4 | 0 | NA | NA | NA | NA | 1.996 | -0.642 | -0.283 | -0.395 | 14.1818181818181 | NA |
| SAK 172 | MDG51512011 | NA | bovine | M | 3 | 0 | NA | NA | NA | NA | 1.996 | -0.642 | -0.283 | -0.395 | 14.1818181818181 | NA |
| SAK 177 | MDG51512011 | NA | bovine | M | 4 | 0 | NA | NA | NA | NA | 1.996 | -0.642 | -0.283 | -0.395 | 14.1818181818181 | NA |
| SAK 176 | MDG51512011 | NA | bovine | M | 4 | 0 | NA | NA | NA | NA | 1.996 | -0.642 | -0.283 | -0.395 | 14.1818181818181 | NA |
| SAK 175 | MDG51512011 | NA | bovine | F | 3 | 0 | NA | NA | NA | NA | 1.996 | -0.642 | -0.283 | -0.395 | 14.1818181818181 | NA |
| SAK 174 | MDG51512011 | NA | bovine | F | 3 | 0 | NA | NA | NA | NA | 1.996 | -0.642 | -0.283 | -0.395 | 14.1818181818181 | NA |
| SAK 173 | MDG51512011 | NA | bovine | M | 3 | 0 | NA | NA | NA | NA | 1.996 | -0.642 | -0.283 | -0.395 | 14.1818181818181 | NA |
| SAK 347 | MDG51512011 | NA | bovine | M | 3 | 0 | NA | NA | NA | NA | 1.996 | -0.642 | -0.283 | -0.395 | 14.1818181818181 | NA |
| TAO_083 | MDG53515033 | NA | bovine | F | 4 | 0 | NA | NA | NA | NA | -0.204 | -0.434 | -1.217 | -0.254 | 0.666666666666666 | NA |
| TAO_065 | MDG53515033 | NA | bovine | M | 5 | 0 | NA | NA | NA | NA | -0.204 | -0.434 | -1.217 | -0.254 | 0.666666666666666 | NA |
| TAO_087 | MDG53515033 | NA | bovine | M | 5 | 0 | NA | NA | NA | NA | -0.204 | -0.434 | -1.217 | -0.254 | 0.666666666666666 | NA |
| TAO_086 | MDG53515033 | NA | bovine | M | 6 | 1 | NA | NA | NA | NA | -0.204 | -0.434 | -1.217 | -0.254 | 0.666666666666666 | NA |

| ID | PCODE | site2 | sp | gender | age | IgG | contact_ruminant | contact_milk | contact_fresh_fluid | profession | fact1 | fact2 | fact3 | fact4 | cattle_density | habitat |
| --- | --- | --- | --- | --- | --- | --- | --- | --- | --- | --- | --- | --- | --- | --- | --- | --- |
| TAO_085 | MDG53515033 | NA | bovine | M | 6 | 1 | NA | NA | NA | NA | -0.204 | -0.434 | -1.217 | -0.254 | 0.666666666666666 | NA |
| TAO_084 | MDG53515033 | NA | bovine | M | 5 | 0 | NA | NA | NA | NA | -0.204 | -0.434 | -1.217 | -0.254 | 0.666666666666666 | NA |
| TAO_055 | MDG53515033 | NA | bovine | M | 5 | 0 | NA | NA | NA | NA | -0.204 | -0.434 | -1.217 | -0.254 | 0.666666666666666 | NA |
| TAO_067 | MDG53515033 | NA | bovine | F | 4 | 0 | NA | NA | NA | NA | -0.204 | -0.434 | -1.217 | -0.254 | 0.666666666666666 | NA |
| TAO_062 | MDG53515033 | NA | bovine | F | 2 | 1 | NA | NA | NA | NA | -0.204 | -0.434 | -1.217 | -0.254 | 0.666666666666666 | NA |
| TAO_061 | MDG53515033 | NA | bovine | F | 2 | 0 | NA | NA | NA | NA | -0.204 | -0.434 | -1.217 | -0.254 | 0.666666666666666 | NA |
| TAO_060 | MDG53515033 | NA | bovine | M | 5 | 1 | NA | NA | NA | NA | -0.204 | -0.434 | -1.217 | -0.254 | 0.666666666666666 | NA |
| TAO_059 | MDG53515033 | NA | bovine | M | 2 | 0 | NA | NA | NA | NA | -0.204 | -0.434 | -1.217 | -0.254 | 0.666666666666666 | NA |
| TAO_058 | MDG53515033 | NA | bovine | M | 1 | 0 | NA | NA | NA | NA | -0.204 | -0.434 | -1.217 | -0.254 | 0.666666666666666 | NA |
| TAO_063 | MDG53515033 | NA | bovine | F | 3 | 0 | NA | NA | NA | NA | -0.204 | -0.434 | -1.217 | -0.254 | 0.666666666666666 | NA |
| TAO_056 | MDG53515033 | NA | bovine | M | 5 | 0 | NA | NA | NA | NA | -0.204 | -0.434 | -1.217 | -0.254 | 0.666666666666666 | NA |
| TAO_064 | MDG53515033 | NA | bovine | M | 2 | 1 | NA | NA | NA | NA | -0.204 | -0.434 | -1.217 | -0.254 | 0.666666666666666 | NA |
| TAO_054 | MDG53515033 | NA | bovine | M | 5 | 0 | NA | NA | NA | NA | -0.204 | -0.434 | -1.217 | -0.254 | 0.666666666666666 | NA |
| TAO_053 | MDG53515033 | NA | bovine | M | 5 | 1 | NA | NA | NA | NA | -0.204 | -0.434 | -1.217 | -0.254 | 0.666666666666666 | NA |
| TAO_052 | MDG53515033 | NA | bovine | F | 2 | 1 | NA | NA | NA | NA | -0.204 | -0.434 | -1.217 | -0.254 | 0.666666666666666 | NA |
| TAO_051 | MDG53515033 | NA | bovine | M | 8 | 1 | NA | NA | NA | NA | -0.204 | -0.434 | -1.217 | -0.254 | 0.666666666666666 | NA |
| TAO_050 | MDG53515033 | NA | bovine | F | 3 | 1 | NA | NA | NA | NA | -0.204 | -0.434 | -1.217 | -0.254 | 0.666666666666666 | NA |
| TAO_049 | MDG53515033 | NA | bovine | M | 6 | 1 | NA | NA | NA | NA | -0.204 | -0.434 | -1.217 | -0.254 | 0.666666666666666 | NA |
| TAO_057 | MDG53515033 | NA | bovine | M | 4 | 0 | NA | NA | NA | NA | -0.204 | -0.434 | -1.217 | -0.254 | 0.666666666666666 | NA |
| TAO_074 | MDG53515033 | NA | bovine | F | 2 | 1 | NA | NA | NA | NA | -0.204 | -0.434 | -1.217 | -0.254 | 0.666666666666666 | NA |
| TAO_081 | MDG53515033 | NA | bovine | M | 5 | 1 | NA | NA | NA | NA | -0.204 | -0.434 | -1.217 | -0.254 | 0.666666666666666 | NA |
| TAO_080 | MDG53515033 | NA | bovine | F | 3 | 0 | NA | NA | NA | NA | -0.204 | -0.434 | -1.217 | -0.254 | 0.666666666666666 | NA |
| TAO_079 | MDG53515033 | NA | bovine | F | 2 | 1 | NA | NA | NA | NA | -0.204 | -0.434 | -1.217 | -0.254 | 0.666666666666666 | NA |
| TAO_078 | MDG53515033 | NA | bovine | M | 3 | 0 | NA | NA | NA | NA | -0.204 | -0.434 | -1.217 | -0.254 | 0.666666666666666 | NA |
| TAO_077 | MDG53515033 | NA | bovine | M | 5 | 0 | NA | NA | NA | NA | -0.204 | -0.434 | -1.217 | -0.254 | 0.666666666666666 | NA |
| TAO_075 | MDG53515033 | NA | bovine | F | 4 | 1 | NA | NA | NA | NA | -0.204 | -0.434 | -1.217 | -0.254 | 0.666666666666666 | NA |
| TAO_082 | MDG53515033 | NA | bovine | F | 3 | 0 | NA | NA | NA | NA | -0.204 | -0.434 | -1.217 | -0.254 | 0.666666666666666 | NA |
| TAO_073 | MDG53515033 | NA | bovine | F | 3 | 0 | NA | NA | NA | NA | -0.204 | -0.434 | -1.217 | -0.254 | 0.666666666666666 | NA |
| TAO_072 | MDG53515033 | NA | bovine | F | 2 | 0 | NA | NA | NA | NA | -0.204 | -0.434 | -1.217 | -0.254 | 0.666666666666666 | NA |
| TAO_071 | MDG53515033 | NA | bovine | F | 2 | 0 | NA | NA | NA | NA | -0.204 | -0.434 | -1.217 | -0.254 | 0.666666666666666 | NA |
| TAO_070 | MDG53515033 | NA | bovine | M | 2 | 0 | NA | NA | NA | NA | -0.204 | -0.434 | -1.217 | -0.254 | 0.666666666666666 | NA |
| TAO_069 | MDG53515033 | NA | bovine | M | 5 | 0 | NA | NA | NA | NA | -0.204 | -0.434 | -1.217 | -0.254 | 0.666666666666666 | NA |
| TAO_068 | MDG53515033 | NA | bovine | M | 4 | 0 | NA | NA | NA | NA | -0.204 | -0.434 | -1.217 | -0.254 | 0.666666666666666 | NA |
| TAO_066 | MDG53515033 | NA | bovine | M | 5 | 1 | NA | NA | NA | NA | -0.204 | -0.434 | -1.217 | -0.254 | 0.666666666666666 | NA |
| TAO_076 | MDG53515033 | NA | bovine | F | 3 | 0 | NA | NA | NA | NA | -0.204 | -0.434 | -1.217 | -0.254 | 0.666666666666666 | NA |
| TAO_103 | MDG53515111 | NA | bovine | M | 2 | 0 | NA | NA | NA | NA | 0.497 | -0.508 | -0.829 | -0.421 | 17.090909090909 | NA |
| TAO_110 | MDG53515111 | NA | bovine | M | 2 | 0 | NA | NA | NA | NA | 0.497 | -0.508 | -0.829 | -0.421 | 17.090909090909 | NA |
| TAO_025 | MDG53515111 | NA | bovine | M | 2 | 0 | NA | NA | NA | NA | 0.497 | -0.508 | -0.829 | -0.421 | 17.090909090909 | NA |
| TAO_105 | MDG53515111 | NA | bovine | M | 2 | 0 | NA | NA | NA | NA | 0.497 | -0.508 | -0.829 | -0.421 | 17.090909090909 | NA |
| TAO_022 | MDG53515111 | NA | bovine | M | 3 | 0 | NA | NA | NA | NA | 0.497 | -0.508 | -0.829 | -0.421 | 17.090909090909 | NA |
| TAO_024 | MDG53515111 | NA | bovine | M | 2 | 0 | NA | NA | NA | NA | 0.497 | -0.508 | -0.829 | -0.421 | 17.090909090909 | NA |
| TAO_101 | MDG53515111 | NA | bovine | M | 3 | 0 | NA | NA | NA | NA | 0.497 | -0.508 | -0.829 | -0.421 | 17.090909090909 | NA |
| TAO_102 | MDG53515111 | NA | bovine | M | 2 | 0 | NA | NA | NA | NA | 0.497 | -0.508 | -0.829 | -0.421 | 17.090909090909 | NA |
| TAO_104 | MDG53515111 | NA | bovine | M | 3 | 0 | NA | NA | NA | NA | 0.497 | -0.508 | -0.829 | -0.421 | 17.090909090909 | NA |
| TAO_106 | MDG53515111 | NA | bovine | M | 2 | 0 | NA | NA | NA | NA | 0.497 | -0.508 | -0.829 | -0.421 | 17.090909090909 | NA |
| TAO_107 | MDG53515111 | NA | bovine | M | 3 | 0 | NA | NA | NA | NA | 0.497 | -0.508 | -0.829 | -0.421 | 17.090909090909 | NA |
| TAO_021 | MDG53515111 | NA | bovine | M | 2 | 0 | NA | NA | NA | NA | 0.497 | -0.508 | -0.829 | -0.421 | 17.090909090909 | NA |
| TAO_109 | MDG53515111 | NA | bovine | M | 2 | 0 | NA | NA | NA | NA | 0.497 | -0.508 | -0.829 | -0.421 | 17.090909090909 | NA |
| TAO_108 | MDG53515111 | NA | bovine | M | 5 | 0 | NA | NA | NA | NA | 0.497 | -0.508 | -0.829 | -0.421 | 17.090909090909 | NA |
| TAO_023 | MDG53515111 | NA | bovine | M | 4 | 0 | NA | NA | NA | NA | 0.497 | -0.508 | -0.829 | -0.421 | 17.090909090909 | NA |
| BET 041 | MDG53517010 | NA | bovine | F | 10 | 0 | NA | NA | NA | NA | 1.774 | -0.208 | -0.339 | 0.092 | 13.4545454545454 | NA |
| BET 070 | MDG53517010 | NA | bovine | F | 1 | 0 | NA | NA | NA | NA | 1.774 | -0.208 | -0.339 | 0.092 | 13.4545454545454 | NA |
| BET 058 | MDG53517010 | NA | bovine | F | 4 | 0 | NA | NA | NA | NA | 1.774 | -0.208 | -0.339 | 0.092 | 13.4545454545454 | NA |
| BET 057 | MDG53517010 | NA | bovine | F | 6 | 0 | NA | NA | NA | NA | 1.774 | -0.208 | -0.339 | 0.092 | 13.4545454545454 | NA |
| BET 056 | MDG53517010 | NA | bovine | F | 2 | 1 | NA | NA | NA | NA | 1.774 | -0.208 | -0.339 | 0.092 | 13.4545454545454 | NA |
| BET 055 | MDG53517010 | NA | bovine | M | 6 | 0 | NA | NA | NA | NA | 1.774 | -0.208 | -0.339 | 0.092 | 13.4545454545454 | NA |
| BET 054 | MDG53517010 | NA | bovine | M | 2 | 0 | NA | NA | NA | NA | 1.774 | -0.208 | -0.339 | 0.092 | 13.4545454545454 | NA |
| BET 053 | MDG53517010 | NA | bovine | F | 10 | 0 | NA | NA | NA | NA | 1.774 | -0.208 | -0.339 | 0.092 | 13.4545454545454 | NA |
| BET 052 | MDG53517010 | NA | bovine | F | 1 | 0 | NA | NA | NA | NA | 1.774 | -0.208 | -0.339 | 0.092 | 13.4545454545454 | NA |
| BET 050 | MDG53517010 | NA | bovine | M | 2 | 0 | NA | NA | NA | NA | 1.774 | -0.208 | -0.339 | 0.092 | 13.4545454545454 | NA |
| BET 044 | MDG53517010 | NA | bovine | M | 2 | 0 | NA | NA | NA | NA | 1.774 | -0.208 | -0.339 | 0.092 | 13.4545454545454 | NA |
| BET 071 | MDG53517010 | NA | bovine | F | 8 | 0 | NA | NA | NA | NA | 1.774 | -0.208 | -0.339 | 0.092 | 13.4545454545454 | NA |
| BET 035 | MDG53517010 | NA | bovine | F | 2 | 0 | NA | NA | NA | NA | 1.774 | -0.208 | -0.339 | 0.092 | 13.4545454545454 | NA |

| ID | PCODE | site2 | sp | gender | age | IgG | contact_ruminant | contact_milk | contact_fresh_fluid | profession | fact1 | fact2 | fact3 | fact4 | cattle_density | habitat |
| --- | --- | --- | --- | --- | --- | --- | --- | --- | --- | --- | --- | --- | --- | --- | --- | --- |
| BET 047 | MDG53517010 | NA | bovine | F | 2 | 0 | NA | NA | NA | NA | 1.774 | -0.208 | -0.339 | 0.092 | 13.4545454545454 | NA |
| BET 046 | MDG53517010 | NA | bovine | M | 2 | 0 | NA | NA | NA | NA | 1.774 | -0.208 | -0.339 | 0.092 | 13.4545454545454 | NA |
| BET 045 | MDG53517010 | NA | bovine | M | 2 | 1 | NA | NA | NA | NA | 1.774 | -0.208 | -0.339 | 0.092 | 13.4545454545454 | NA |
| BET 051 | MDG53517010 | NA | bovine | F | 3 | 0 | NA | NA | NA | NA | 1.774 | -0.208 | -0.339 | 0.092 | 13.4545454545454 | NA |
| BET 005 | MDG53517010 | NA | bovine | F | 5 | 0 | NA | NA | NA | NA | 1.774 | -0.208 | -0.339 | 0.092 | 13.4545454545454 | NA |
| BET 102 | MDG53517010 | NA | bovine | F | 11 | 0 | NA | NA | NA | NA | 1.774 | -0.208 | -0.339 | 0.092 | 13.4545454545454 | NA |
| BET 100 | MDG53517010 | NA | bovine | F | 10 | 0 | NA | NA | NA | NA | 1.774 | -0.208 | -0.339 | 0.092 | 13.4545454545454 | NA |
| BET 040 | MDG53517010 | NA | bovine | M | 2 | 0 | NA | NA | NA | NA | 1.774 | -0.208 | -0.339 | 0.092 | 13.4545454545454 | NA |
| BET 078 | MDG53517010 | NA | bovine | F | 6 | 0 | NA | NA | NA | NA | 1.774 | -0.208 | -0.339 | 0.092 | 13.4545454545454 | NA |
| BET 077 | MDG53517010 | NA | bovine | F | 3 | 1 | NA | NA | NA | NA | 1.774 | -0.208 | -0.339 | 0.092 | 13.4545454545454 | NA |
| BET 037 | MDG53517010 | NA | bovine | F | 6 | 0 | NA | NA | NA | NA | 1.774 | -0.208 | -0.339 | 0.092 | 13.4545454545454 | NA |
| BET 004 | MDG53517010 | NA | bovine | F | 8 | 0 | NA | NA | NA | NA | 1.774 | -0.208 | -0.339 | 0.092 | 13.4545454545454 | NA |
| BET 072 | MDG53517010 | NA | bovine | F | 8 | 0 | NA | NA | NA | NA | 1.774 | -0.208 | -0.339 | 0.092 | 13.4545454545454 | NA |
| BET 015 | MDG53517010 | NA | bovine | M | 3 | 0 | NA | NA | NA | NA | 1.774 | -0.208 | -0.339 | 0.092 | 13.4545454545454 | NA |
| BET 028 | MDG53517010 | NA | bovine | F | 7 | 1 | NA | NA | NA | NA | 1.774 | -0.208 | -0.339 | 0.092 | 13.4545454545454 | NA |
| BET 105 | MDG53517010 | NA | bovine | M | 4 | 0 | NA | NA | NA | NA | 1.774 | -0.208 | -0.339 | 0.092 | 13.4545454545454 | NA |
| BET 036 | MDG53517010 | NA | bovine | F | 6 | 0 | NA | NA | NA | NA | 1.774 | -0.208 | -0.339 | 0.092 | 13.4545454545454 | NA |
| BET 038 | MDG53517010 | NA | bovine | M | 7 | 1 | NA | NA | NA | NA | 1.774 | -0.208 | -0.339 | 0.092 | 13.4545454545454 | NA |
| BET 074 | MDG53517010 | NA | bovine | M | 7 | 0 | NA | NA | NA | NA | 1.774 | -0.208 | -0.339 | 0.092 | 13.4545454545454 | NA |
| BET 073 | MDG53517010 | NA | bovine | M | 8 | 0 | NA | NA | NA | NA | 1.774 | -0.208 | -0.339 | 0.092 | 13.4545454545454 | NA |
| BET 008 | MDG53517050 | NA | bovine | F | 6 | 0 | NA | NA | NA | NA | 1.928 | -0.116 | -0.143 | 0.233 | 14.8333333333333 | NA |
| BET 094 | MDG53517050 | NA | bovine | F | 1 | 0 | NA | NA | NA | NA | 1.928 | -0.116 | -0.143 | 0.233 | 14.8333333333333 | NA |
| BET 039 | MDG53517050 | NA | bovine | M | 6 | 1 | NA | NA | NA | NA | 1.928 | -0.116 | -0.143 | 0.233 | 14.8333333333333 | NA |
| BET 007 | MDG53517050 | NA | bovine | F | 4 | 1 | NA | NA | NA | NA | 1.928 | -0.116 | -0.143 | 0.233 | 14.8333333333333 | NA |
| BET 115 | MDG53517050 | NA | bovine | F | 6 | 0 | NA | NA | NA | NA | 1.928 | -0.116 | -0.143 | 0.233 | 14.8333333333333 | NA |
| BET 104 | MDG53517050 | NA | bovine | F | 2 | 0 | NA | NA | NA | NA | 1.928 | -0.116 | -0.143 | 0.233 | 14.8333333333333 | NA |
| BET 106 | MDG53517050 | NA | bovine | F | 2 | 0 | NA | NA | NA | NA | 1.928 | -0.116 | -0.143 | 0.233 | 14.8333333333333 | NA |
| BET 107 | MDG53517050 | NA | bovine | M | 2 | 0 | NA | NA | NA | NA | 1.928 | -0.116 | -0.143 | 0.233 | 14.8333333333333 | NA |
| BET 108 | MDG53517050 | NA | bovine | M | 2 | 0 | NA | NA | NA | NA | 1.928 | -0.116 | -0.143 | 0.233 | 14.8333333333333 | NA |
| BET 109 | MDG53517050 | NA | bovine | F | 2 | 0 | NA | NA | NA | NA | 1.928 | -0.116 | -0.143 | 0.233 | 14.8333333333333 | NA |
| BET 110 | MDG53517050 | NA | bovine | F | 2 | 0 | NA | NA | NA | NA | 1.928 | -0.116 | -0.143 | 0.233 | 14.8333333333333 | NA |
| BET 111 | MDG53517050 | NA | bovine | F | 3 | 0 | NA | NA | NA | NA | 1.928 | -0.116 | -0.143 | 0.233 | 14.8333333333333 | NA |
| BET 112 | MDG53517050 | NA | bovine | M | 3 | 0 | NA | NA | NA | NA | 1.928 | -0.116 | -0.143 | 0.233 | 14.8333333333333 | NA |
| BET 103 | MDG53517050 | NA | bovine | F | 4 | 0 | NA | NA | NA | NA | 1.928 | -0.116 | -0.143 | 0.233 | 14.8333333333333 | NA |
| BET 114 | MDG53517050 | NA | bovine | F | 4 | 0 | NA | NA | NA | NA | 1.928 | -0.116 | -0.143 | 0.233 | 14.8333333333333 | NA |
| BET 116 | MDG53517050 | NA | bovine | F | 6 | 0 | NA | NA | NA | NA | 1.928 | -0.116 | -0.143 | 0.233 | 14.8333333333333 | NA |
| BET 117 | MDG53517050 | NA | bovine | F | 7 | 0 | NA | NA | NA | NA | 1.928 | -0.116 | -0.143 | 0.233 | 14.8333333333333 | NA |
| BET 092 | MDG53517050 | NA | bovine | M | 1 | 0 | NA | NA | NA | NA | 1.928 | -0.116 | -0.143 | 0.233 | 14.8333333333333 | NA |
| BET 119 | MDG53517050 | NA | bovine | F | 10 | 0 | NA | NA | NA | NA | 1.928 | -0.116 | -0.143 | 0.233 | 14.8333333333333 | NA |
| BET 120 | MDG53517050 | NA | bovine | M | 7 | 0 | NA | NA | NA | NA | 1.928 | -0.116 | -0.143 | 0.233 | 14.8333333333333 | NA |
| BET 121 | MDG53517050 | NA | bovine | M | 7 | 0 | NA | NA | NA | NA | 1.928 | -0.116 | -0.143 | 0.233 | 14.8333333333333 | NA |
| BET 123 | MDG53517050 | NA | bovine | F | 6 | 0 | NA | NA | NA | NA | 1.928 | -0.116 | -0.143 | 0.233 | 14.8333333333333 | NA |
| BET 124 | MDG53517050 | NA | bovine | F | 3 | 0 | NA | NA | NA | NA | 1.928 | -0.116 | -0.143 | 0.233 | 14.8333333333333 | NA |
| BET 125 | MDG53517050 | NA | bovine | M | 2 | 0 | NA | NA | NA | NA | 1.928 | -0.116 | -0.143 | 0.233 | 14.8333333333333 | NA |
| BET 113 | MDG53517050 | NA | bovine | M | 2 | 0 | NA | NA | NA | NA | 1.928 | -0.116 | -0.143 | 0.233 | 14.8333333333333 | NA |
| BET 101 | MDG53517050 | NA | bovine | M | 1 | 0 | NA | NA | NA | NA | 1.928 | -0.116 | -0.143 | 0.233 | 14.8333333333333 | NA |
| BET 098 | MDG53517050 | NA | bovine | F | 2 | 0 | NA | NA | NA | NA | 1.928 | -0.116 | -0.143 | 0.233 | 14.8333333333333 | NA |
| BET 097 | MDG53517050 | NA | bovine | F | 3 | 0 | NA | NA | NA | NA | 1.928 | -0.116 | -0.143 | 0.233 | 14.8333333333333 | NA |
| BET 096 | MDG53517050 | NA | bovine | F | 1 | 0 | NA | NA | NA | NA | 1.928 | -0.116 | -0.143 | 0.233 | 14.8333333333333 | NA |
| BET 095 | MDG53517050 | NA | bovine | M | 1 | 0 | NA | NA | NA | NA | 1.928 | -0.116 | -0.143 | 0.233 | 14.8333333333333 | NA |
| BET 118 | MDG53517050 | NA | bovine | F | 10 | 0 | NA | NA | NA | NA | 1.928 | -0.116 | -0.143 | 0.233 | 14.8333333333333 | NA |
| BET 093 | MDG53517050 | NA | bovine | F | 1 | 0 | NA | NA | NA | NA | 1.928 | -0.116 | -0.143 | 0.233 | 14.8333333333333 | NA |
| TU1 285 | MDG51520010 | NA | bovine | F | 4 | 1 | NA | NA | NA | NA | 2.610 | -0.570 | -0.950 | 0.137 | 105.5 | NA |
| TU1 282 | MDG51520010 | NA | bovine | F | 3 | 0 | NA | NA | NA | NA | 2.610 | -0.570 | -0.950 | 0.137 | 105.5 | NA |
| TU2 022 | MDG51520030 | NA | bovine | F | 7 | 0 | NA | NA | NA | NA | 1.877 | 0.497 | -0.538 | -0.407 | 45.6363636363636 | NA |
| TU2 103 | MDG51520050 | NA | bovine | F | 5 | 1 | NA | NA | NA | NA | 2.215 | -0.111 | -0.733 | -0.004 | 47.6 | NA |
| TU2 102 | MDG51520050 | NA | bovine | F | 3 | 0 | NA | NA | NA | NA | 2.215 | -0.111 | -0.733 | -0.004 | 47.6 | NA |
| TU12 142 | MDG51520071 | NA | bovine | F | 7 | 1 | NA | NA | NA | NA | 2.159 | -0.479 | -0.827 | 0.114 | 64.4285714285714 | NA |
| TU12 143 | MDG51520071 | NA | bovine | F | 6 | 1 | NA | NA | NA | NA | 2.159 | -0.479 | -0.827 | 0.114 | 64.4285714285714 | NA |
| TU12 176 | MDG51520071 | NA | bovine | F | 5 | 0 | NA | NA | NA | NA | 2.159 | -0.479 | -0.827 | 0.114 | 64.4285714285714 | NA |
| TU12 161 | MDG51520071 | NA | bovine | F | 5 | 0 | NA | NA | NA | NA | 2.159 | -0.479 | -0.827 | 0.114 | 64.4285714285714 | NA |
| TU2 036 | MDG51520090 | NA | bovine | F | 5 | 0 | NA | NA | NA | NA | 1.495 | 0.316 | -0.030 | -0.641 | 12.2962962962962 | NA |
| TU2 106 | MDG51520090 | NA | bovine | F | 5 | 1 | NA | NA | NA | NA | 1.495 | 0.316 | -0.030 | -0.641 | 12.2962962962962 | NA |

| ID | PCODE | site2 | sp | gender | age | IgG | contact_ruminant | contact_milk | contact_fresh_fluid | profession | fact1 | fact2 | fact3 | fact4 | cattle_density | habitat |
| --- | --- | --- | --- | --- | --- | --- | --- | --- | --- | --- | --- | --- | --- | --- | --- | --- |
| TU1 246 | MDG51520090 | NA | bovine | F | 4 | 0 | NA | NA | NA | NA | 1.495 | 0.316 | -0.030 | -0.641 | 12.2962962962962 | NA |
| TU1 249 | MDG51520090 | NA | bovine | F | 3 | 0 | NA | NA | NA | NA | 1.495 | 0.316 | -0.030 | -0.641 | 12.2962962962962 | NA |
| TU12 144 | MDG51520090 | NA | bovine | F | 5 | 0 | NA | NA | NA | NA | 1.495 | 0.316 | -0.030 | -0.641 | 12.2962962962962 | NA |
| TU2 014 | MDG51520111 | NA | bovine | F | 10 | 0 | NA | NA | NA | NA | 2.037 | 0.038 | -0.805 | -0.049 | 12.5833333333333 | NA |
| TU2 069 | MDG51520111 | NA | bovine | F | 6 | 0 | NA | NA | NA | NA | 2.037 | 0.038 | -0.805 | -0.049 | 12.5833333333333 | NA |
| TU1 313 | MDG51520111 | NA | bovine | F | 4 | 1 | NA | NA | NA | NA | 2.037 | 0.038 | -0.805 | -0.049 | 12.5833333333333 | NA |
| TU1 219 | MDG51520111 | NA | bovine | F | 4 | 0 | NA | NA | NA | NA | 2.037 | 0.038 | -0.805 | -0.049 | 12.5833333333333 | NA |
| TU1 220 | MDG51520111 | NA | bovine | F | 5 | 0 | NA | NA | NA | NA | 2.037 | 0.038 | -0.805 | -0.049 | 12.5833333333333 | NA |
| TU12 187 | MDG51520131 | NA | bovine | F | 4 | 0 | NA | NA | NA | NA | 1.809 | 0.245 | -0.980 | -0.111 | 14.6 | NA |
| TU12 186 | MDG51520131 | NA | bovine | F | 4 | 0 | NA | NA | NA | NA | 1.809 | 0.245 | -0.980 | -0.111 | 14.6 | NA |
| TU1 230 | MDG51520131 | NA | bovine | F | 4 | 0 | NA | NA | NA | NA | 1.809 | 0.245 | -0.980 | -0.111 | 14.6 | NA |
| TU1 276 | MDG51520131 | NA | bovine | F | 3 | 0 | NA | NA | NA | NA | 1.809 | 0.245 | -0.980 | -0.111 | 14.6 | NA |
| TU1 256 | MDG51520131 | NA | bovine | F | 5 | 0 | NA | NA | NA | NA | 1.809 | 0.245 | -0.980 | -0.111 | 14.6 | NA |
| TU2 031 | MDG51520150 | NA | bovine | M | 6 | 0 | NA | NA | NA | NA | 1.842 | 0.262 | -0.548 | -0.340 | 8.33333333333333 | NA |
| TU2 032 | MDG51520150 | NA | bovine | F | 5 | 0 | NA | NA | NA | NA | 1.842 | 0.262 | -0.548 | -0.340 | 8.33333333333333 | NA |
| TU1 296 | MDG51520170 | NA | bovine | F | 5 | 0 | NA | NA | NA | NA | 2.223 | -0.323 | -0.278 | -0.757 | 16.7777777777777 | NA |
| TU12 180 | MDG51520191 | NA | bovine | F | 5 | 0 | NA | NA | NA | NA | 1.925 | 0.486 | -0.604 | -0.310 | 12.3 | NA |
| TU1 261 | MDG51520191 | NA | bovine | M | 6 | 0 | NA | NA | NA | NA | 1.925 | 0.486 | -0.604 | -0.310 | 12.3 | NA |
| TU1 211 | MDG51520191 | NA | bovine | F | 5 | 0 | NA | NA | NA | NA | 1.925 | 0.486 | -0.604 | -0.310 | 12.3 | NA |
| TU12 190 | MDG51520191 | NA | bovine | F | 4 | 0 | NA | NA | NA | NA | 1.925 | 0.486 | -0.604 | -0.310 | 12.3 | NA |
| TU2 003 | MDG51520192 | NA | bovine | F | 6 | 0 | NA | NA | NA | NA | 1.658 | 0.428 | -0.464 | -0.294 | 11.5 | NA |
| TU2 011 | MDG51520192 | NA | bovine | F | 5 | 0 | NA | NA | NA | NA | 1.658 | 0.428 | -0.464 | -0.294 | 11.5 | NA |
| TU2 029 | MDG51520192 | NA | bovine | F | 9 | 0 | NA | NA | NA | NA | 1.658 | 0.428 | -0.464 | -0.294 | 11.5 | NA |
| TU2 045 | MDG51520192 | NA | bovine | F | 7 | 0 | NA | NA | NA | NA | 1.658 | 0.428 | -0.464 | -0.294 | 11.5 | NA |
| TU2 047 | MDG51520192 | NA | bovine | M | 6 | 0 | NA | NA | NA | NA | 1.658 | 0.428 | -0.464 | -0.294 | 11.5 | NA |
| TU2 052 | MDG51520192 | NA | bovine | F | 5 | 0 | NA | NA | NA | NA | 1.658 | 0.428 | -0.464 | -0.294 | 11.5 | NA |
| TU1 278 | MDG51520192 | NA | bovine | F | 4 | 0 | NA | NA | NA | NA | 1.658 | 0.428 | -0.464 | -0.294 | 11.5 | NA |
| TU2 127 | MDG51520192 | NA | bovine | F | 5 | 1 | NA | NA | NA | NA | 1.658 | 0.428 | -0.464 | -0.294 | 11.5 | NA |
| TU12 185 | MDG51520192 | NA | bovine | F | 4 | 0 | NA | NA | NA | NA | 1.658 | 0.428 | -0.464 | -0.294 | 11.5 | NA |
| TU1 222 | MDG51520192 | NA | bovine | F | 6 | 0 | NA | NA | NA | NA | 1.658 | 0.428 | -0.464 | -0.294 | 11.5 | NA |
| TU2 010 | MDG51520192 | NA | bovine | F | 7 | 1 | NA | NA | NA | NA | 1.658 | 0.428 | -0.464 | -0.294 | 11.5 | NA |
| TU1 274 | MDG51520192 | NA | bovine | F | 3 | 0 | NA | NA | NA | NA | 1.658 | 0.428 | -0.464 | -0.294 | 11.5 | NA |
[truncated: 25,936 more chars]
